# Dipolarophile-Controlled Regioselective 1,3-Dipolar Cycloaddition: A Switchable Divergent Access to Functionalized *N*-Fused Pyrrolidinyl Spirooxindoles

**DOI:** 10.3390/ijms24043771

**Published:** 2023-02-13

**Authors:** Yongchao Wang, Lijun Yan, Yuxin Yan, Sujin Li, Hongying Lu, Jia Liu, Jianwei Dong

**Affiliations:** 1 Colleage of Vocational and Technical Education, Yunnan Normal University, Kunming 650092, China; 2 Colleage of Chemistry and Environmental Science, Qujing Normal University, Qujing 655011, China

**Keywords:** dipolarophile-controlled, 1,3-dipolar cycloaddition, switchable divergent synthesis, spirooxindole, 1,4-enedione derivatives

## Abstract

*N*-fused pyrrolidinyl spirooxindole belongs to a class of privileged heterocyclic scaffolds and is prevalent in natural alkaloids and synthetic pharmaceutical molecules. To realize the switchable synthesis of divergent *N*-fused pyrrolidinyl spirooxindoles for further biological activity evaluation via a substrate-controlled strategy, a chemically sustainable, catalysis-free, and dipolarophile-controlled three-component 1,3-dipolar cycloaddition of isatin-derived azomethine ylides with diverse dipolarophiles is described in this work. A total of 40 functionalized *N*-fused pyrrolidinyl spirooxindoles were synthesized in 76–95% yields with excellent diastereoselectivities (up to >99:1 dr). The scaffolds of these products can be well-controlled by employing different 1,4-enedione derivatives as dipolarophiles in EtOH at room temperature. This study provides an efficient strategy to afford a spectrum of natural-like and potentially bioactive *N*-fused pyrrolidinyl spirooxindoles.

## 1. Introduction

**Figure 1:**
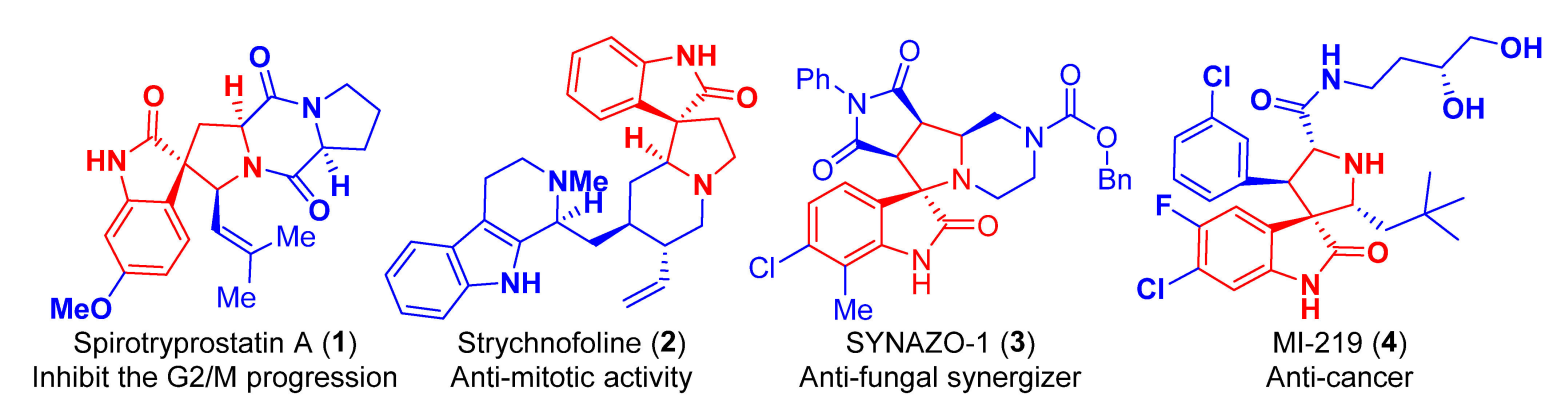
Representative natural and synthetic *N*-fused pyrrolidinyl spirooxindoles.

The spirooxindole system belongs to a class of privileged heterocyclic scaffolds and is prevalent in natural alkaloids and synthetic pharmaceutical molecules [1,2,3,4]. As a representative subclass of spirooxindoles, *N*-fused pyrrolidinyl spirooxindoles are gradually became appealing synthetic targets as the diverse biological activities [5,6,7,8] (Figure 1). Spirotryprostatin A (**1**) was first isolated from the fermentation broth of *Aspergillus fumigatu*, which can inhibit the G2/M progression of mammalian tsFT210 cells [9]. Strychnofoline (**2**) is a representative alkaloid and displays high antimitotic activity against cultures of mice melanoma and Eurlich tumor cells [10,11]. Synthetic *N*-fused pyrrolidinyl spirooxindoles SYNAZO-1 (**3**) [12] and MI-219 (**4**) [13] are promising antifungal synergizer and anticancer drug candidate, respectively. Other *N*-fused pyrrolidinyl spirooxindoles have also displayed a broad range of biological activities, such as antitumor, antiviral, anti-HIV activities, etc. [14,15,16,17].

**Scheme 1:**
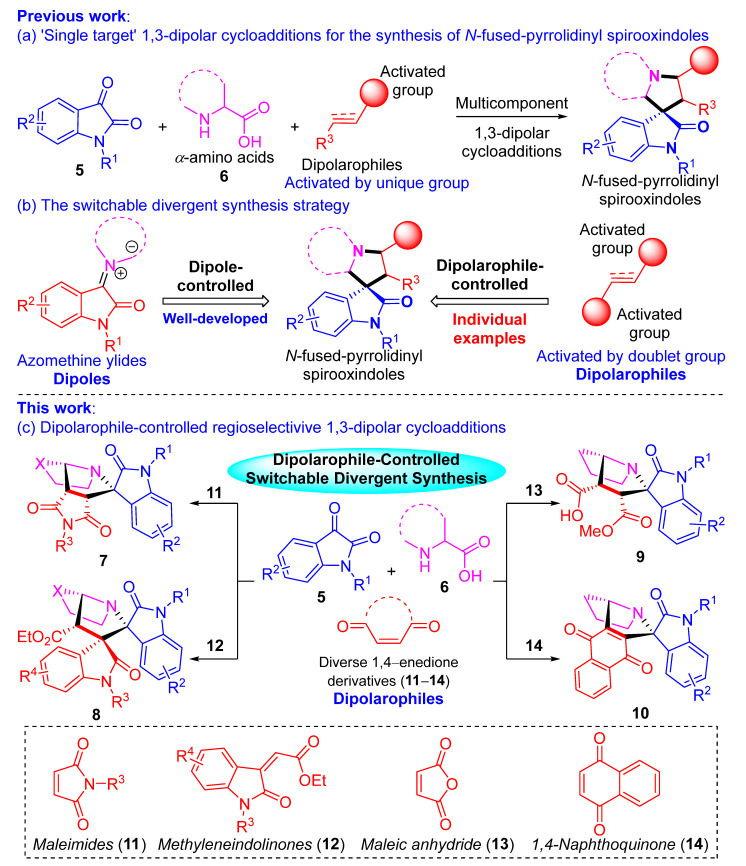
Three-component 1,3-dipolar cycloaddition reactions.

Due to the diverse biological activities and the challenge of the simultaneous creation of spiro-quaternary centers, the development of cyclizations, cycloadditions, and cascade reactions for the construction of *N*-fused pyrrolidinyl spirooxindoles has continuously attracted the attention of synthetic chemists [18,19,20,21,22,23]. Among all the reported synthetic methodologies, multicomponent 1,3-dipolar cycloaddition [24,25,26,27] has been proven to be one of the most efficient and straightforward pathways for the construction of these unique nitrogen-containing skeletons [28,29,30] (Scheme 1a). Isatin-derived azomethine ylides were recognized as typical dipoles and generally generated in situ by decarboxylative condensation of *α*-amino acids with isatins, which then react with dipolarophiles [31]. Significantly, diverse dipolarophiles have proven highly fruitful, including electron-deficient alkenes (activated by nitro [32,33,34,35,36], carbonyl [37,38,39,40], or other electron-withdrawing groups [41,42,43]), alkynes [44,45,46], arylacetylenes [47], allenes [48], *N*-heteroenes [49,50], and strained cyclopropenes [51]. However, previously reported methods mainly focused on the traditional ‘single target’ approach, and thus, only one type of spirooxindole skeleton can be prepared via the 1,3-dipolar cycloaddition of isatin-derived azomethine ylides to monogroup-activated dipolarophiles. Fortunately, the switchable divergent synthesis strategy provides a straightforward method whereby the products can be switched by changing the reaction conditions or using common pluripotent substrates [52,53,54,55]. In this context, the scaffolds of *N*-fused pyrrolidinyl spirooxindoles can be regulated and switched via the dipole-controlled or dipolarophile-controlled strategy. To date, several powerful dipole-controlled multicomponent 1,3-dipolar cycloadditions have been explored selectively to access *N*-fused pyrrolidinyl spirooxindoles by using diverse *α*-amino acids as precursors to furnish isatin-derived azomethine ylides [40,56,57,58,59] (Scheme 1b). Noticeably, 1,4-enedione derivatives [60,61,62], such as maleimides [63,64], methylene indolinones [65,66,67], 1,4-naphthoquinone [68,69,70,71], maleic anhydride, etc., can be activated by two carbonyl groups, which can enhance the flexibility and diversity of cyclization in synthetic chemistry. To the best of our knowledge, only individual multicomponent dipolarophile-controlled methods have been elegantly developed to synthesize *N*-fused pyrrolidinyl spirooxindoles, while designated catalysts are necessary for this synthetic strategy [72]. Therefore, to fully take advantage of the library of 1,4-enedione derivatives and fill the gap of structural diversity, the development of efficient reactions to access biologically important *N*-fused pyrrolidinyl spirooxindoles is urgent in the field of chemical synthesis and drug discovery. Herein, we disclose a dipolarophile-controlled, catalysis-free, and eco-friendly 1,3-dipolar cycloaddition of in situ generated isatin-derived azomethine ylides with 1,4-enedione derivatives in EtOH at room temperature (Scheme 1c). An array of 1,4-enedione derivatives (including maleimides, methylene indolinones, maleic anhydride, and 1,4-naphthoquinone) were successfully utilized in the assembly of diversely substituted and functionalized *N*-fused pyrrolidinyl spirooxindoles. This reaction not only realizes a concise dipolarophile-controlled, catalysis-free 1,3-dipolar cycloaddition under green conditions but also provides a practical strategy for the construction of functionalized *N*-fused pyrrolidinyl spirooxindoles.

## 2. Results and Discussion

In this study, we describe a substrate-controlled 1,3-dipolar cycloaddition reaction of isatins **5**, *α*-amino acids (**6**), and 1,4-enedione derivatives to access divergent *N*-fused pyrrolidinyl spirooxindoles. To establish the feasibility of this strategy, as well as to optimize the reaction conditions, the three-component reaction of isatin **5a** (0.5 mmol), *L*-proline (**6a**, 0.6 mmol), and *N*-ethylmaleimide (**11a**, 0.6 mmol) was selected as the model reaction (Table 1). Initially, the model reaction was performed in a range of organic solvents (including proton solvents and aprotic solvents) at room temperature for 4 h (entries 1–16). The model reaction proceeded smoothly in the screened solvents with excellent diastereoselectivity (all >20:1 dr), with yields ranging from 18 to 81% (entries 1-14). Pleasingly, the 1,3-dipolar cycloaddition reaction produced the desired product in excellent yield in the presence of MeOH or EtOH (entries 15 and 16). Taking the toxicity and green synthesis into consideration, we chose EtOH as the optimal solvent to further optimize the reaction temperature and time. When the reaction temperature rose from room temperature to 40 °C or reflux, the product yields were not significantly changed (entries 16–18). In addition, there was no significant increase in yield when the reaction time was extended from 4 h to 8 h or 24 h (entries 16, 20 and 21), while the product yield declined to 81% when the reaction time was reduced to 2 h. The target product, *N*-fused-pyrrolidinyl spirooxindole (**7a**)**,** could be easily precipitated from the reaction solution and purified by filtering and recrystallization or column chromatography separation. Overall, the optimal reaction conditions for synthesizes of *N*-fused-pyrrolidinyl spirooxindoles were achieved via a ‘one-pot’ reaction of isatins (**5**), *α*-amino acids (**6**), and 1,4-enedione derivatives in EtOH at room temperature for 4 h.

**Scheme 2:**
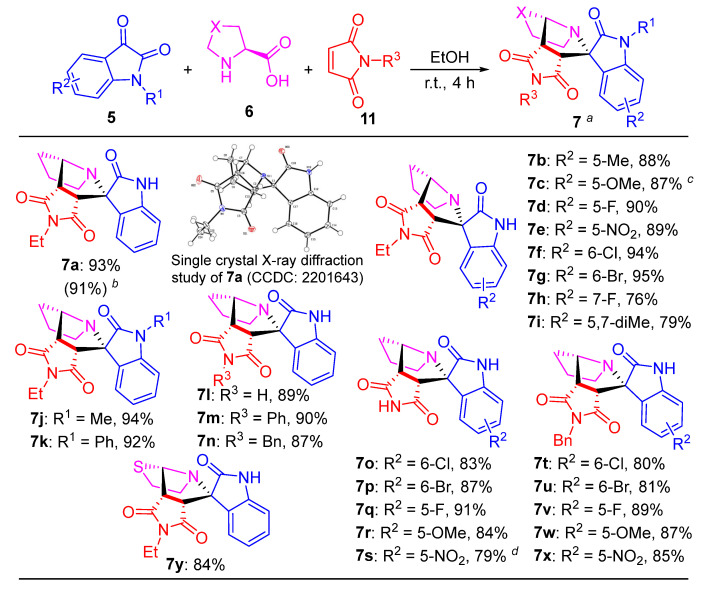
The synthesis of product **7**: *^a^* All reactions were carried out with isatins **5** (0.5 mmol), *a*-amino acids (**6**, 0.6 mmol) and maleimides **11** (0.6 mmol) in EtOH (3 mL) at room temperatures for 4 h. Isolated or recrystallizated yields based on isatin **5a** and all >25:1 dr (except for **7c** and **7s**) by ^1^H NMR analysis. *^b^* D-proline was used instead of **6a**. *^c^* 20:1 dr. *^d^* 17:1 dr.

With the optimal reaction conditions in hand, we then explored the scope of this three-component 1,3-dipolar cycloaddition reaction. Initially, an array of maleimides (**11**) were fixed as dipolarophiles to test a variety of isatin-derived azomethine ylides generated in situ from isatins (**5)** and *α*-amino acids (**6**), the results of which are presented in Scheme 2. Under the optimized conditions, this three-component 1,3-dipolar cycloaddition reaction was tolerated by all the tested isatins (**5**), *α*-amino acids (**6**)**,** and maleimides (**11**) bearing various different electron properties and substitution patterns, affording the corresponding products (**7**) in 76–95% yields with excellent diastereoselectivities (17:1-> 99:1 dr). For the isatins (**5**), the aromatic ring bearing either electron-donating (Me, OMe) or electron-withdrawing functional groups (F, Cl, Br, NO_2_) and the various *N*-substituted groups (R^2^) could form the target products in high yields with excellent diastereoselectivities (up to >99:1 dr) (**7a–7k**). For the substitution patterns of isatins (**5**), 5-substitution and 6-substitution were proven to be the preferred substitution position (87–95% yields) compared with 7-substitution (76–79% yields). The results clearly show that the types of *N*-hydrocarbyl of maleimides (**11**) and *α*-amino acids (**6**) have no obvious effect on the reaction efficiency and diastereoselectivities (**7l–7y**). *D*-proline was also employed and led to high-purity **7a** in 91% yield. Furthermore, the structure and relative configuration of product **7a** (CCDC 2201643) were unequivocally established by X-ray crystallographic analysis.

**Scheme 3:**
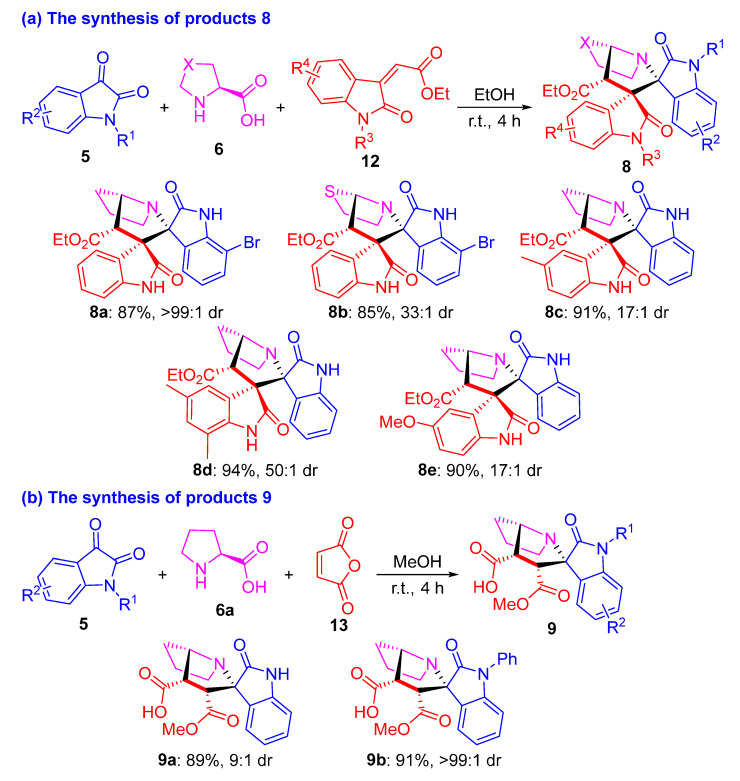
The synthesis of products **8** and **9**.

Encouraged by the above results, we further screened different types of 1,4-enedione derivatives as dipolarophiles. When various methylene indolinones (**12**) were employed, this three-component 1,3-dipolar cycloaddition reaction occurred smoothly and afforded the corresponding products (**8**) in 85–94% yields with excellent diastereoselectivities (17:1->99:1 dr) (Scheme 3a). It is worth noting that the 7-substitution pattern and steric hindrance of isatins (**5**) had no significant effect on the reaction efficiency and diastereoselectivities. Based on this 1,3-dipolar cycloaddition reaction, a series of complex *N*-fused pyrrolidinyl-dispirooxindoles (**8**) bearing two spiro-quaternary centers were obtained. Subsequently, maleic anhydride (**13**) was employed as the dipolarophile under the optimal reaction conditions. However, the use of EtOH as the solvent does not seem to favor the formation of the products (**9**), and only moderate yields (67% and 72%) were obtained. When MeOH was employed as the reaction solvent, the decyclization products **9a** and **9b** could be obtained in 89% and 91% yield, respectively (Scheme 3b), which may undergo a dehydration–decarboxylation–cycloaddition–hydrolysis–methylesterification sequence reaction [69]. In addition, we noticed that the diastereoselectivities of product **9** were tightly affected by the *N* group of isatins (**5**) (9:1 vs. >99:1 dr), which may be related to the hydrogen bonding between *NH* and the hydroxyl group. 

**Scheme 4:**
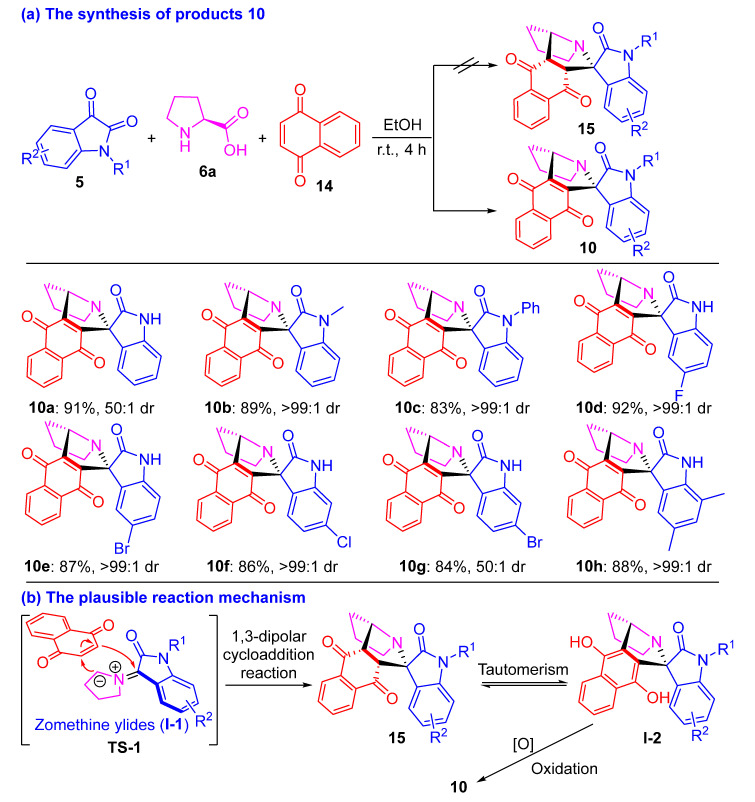
The synthesis of product **10**.

Subsequently, we investigated the substrate scopes by choosing 1,4-naphthoquinone (**14**) as the dipolarophile (Scheme 4a). Surprisingly, we did not observe the expected products (**15**). Instead, polycyclic *N-*fused-pyrrolidinyl spirooxindoles (**10**) bearing an unsaturated structure unit were obtained in 83–92% yields with excellent diastereoselectivities (50:1->99:1 dr), regardless of the positions and electronic properties of the substituents on the phenyl ring of the R^1^ group and *N*-hydrocarbyl group (R^2^) of isatins (**5**). Based on our previous work [73,74] and other studies [34,40,66,70], a plausible mechanism was proposed to explain the reaction process (Scheme 4b). The two nucleophilic carbons of the mentioned isatin-derived azomethine ylides (**I-1**) add to the corresponding electron-deficient carbons of the dipolarophile during the cycloadditions via 1,3-cycloaddition reactions (**TS-1**), leading to the formation of compound **15**. Then, the intermediates **I-2** were formed via the tautomerization of compounds **15**, which subsequently underwent rapid oxidation under air conditions to afford the product **10**. 

**Scheme 5:**
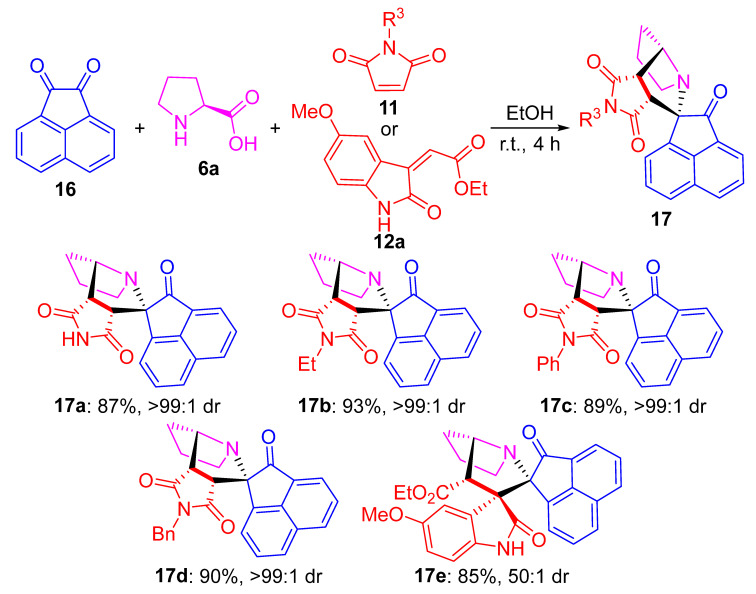
The synthesis of product **17**.

We also explored the 1,3-dipolar cycloaddition reactions by employing acenaphthenequinone-derived azomethine ylide as dipole, which was generated in situ from acenaphthenequinone (**16**) and *L*-proline (**6a**) (Scheme 5). Under reaction conditions similar to those described above, the reactions of 1,4-enedione derivatives (**11)** with azomethine ylide proceeded smoothly, and the corresponding products (**17a–17d**) were furnished in 87–93% yields with all >99:1 dr levels. This 1,3-dipolar cycloaddition reaction also occurred smoothly when methylene indolinone (**12a**) was used as the dipolarophile, affording product **17e** with an 85% yield with 50:1 dr.

**Scheme 6:**
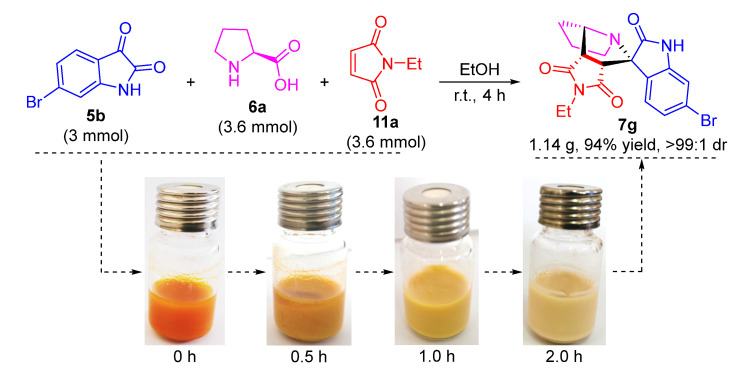
Scale-up of the 1,3-dipolar cycloaddition reaction.

To further demonstrate the synthetic utility of the 1,3-dipolar cycloaddition reaction, a gram-scale synthesis of **7g** was carried out (Scheme 6). Under the optimized conditions, this three-component reaction of 6-bromoisatin (**5b**, 3.0 mmol), *L*-proline (**6a**, 3.6 mmol), and *N*-ethylmaleimide (**11a**, 3.6 mmol) proceeded smoothly and led to the desired product (**7g**) in 94% yield with excellent diastereoselectivity (>99:1 dr). It is worth noting that visible color changes were observed during the reaction process, which were and accompanied by precipitation. Finally, the high-purity target compound (**7g**) was obtained by simple filtration and recrystallization.

## 3. Materials and Methods

### 3.1. General Information 

Reagents and materials (Adamas, Shanghai, China) were of the highest commercial grade and were used without further purification. NMR spectra were recorded on a Bruker DRX 400 (^1^H: 400 MHz, ^13^C: 100 MHz) with TMS as the internal standard. Chemical shifts (*δ*) were expressed in ppm, *J* values were given in Hz, and deuterated DMSO-*d_6_* was used as a solvent. IR spectra were recorded by FT-IR on a Thermo Nicolet Avatar 360 using a KBr pellet. The mass spectroscopic data were obtained from an Agilent 6550 Q-TOF and a Thermo Fisher-QE spectrometer. Melting points were determined with an SGWX-4 melting-point apparatus. The reactions were monitored by thin-layer chromatography (TLC) with silica gel GF_254_, and all compounds were visualized by UV and sprayed with H_2_SO_4_ (10%) in ethanol, followed by heating. A suitable single crystal was selected and analyzed with Rigaku XtaLab Synergy. Supplementary Materials: ^1^H-NMR, ^13^C-NMR spectra and HRMS of compounds **7**, **8**, **9**,**10**, **17**; Single crystal X-ray diffraction study data of compound **7a**.

### 3.2. General Procedure for the Synthesis of Compounds ***7***–***10***

The target compounds were obtained by a ‘one-pot’ reaction of isatins (5, 0.5 mmol), *α*-amino acids (6, 0.6 mmol) and 1,4-enedione derivatives (11–14, 0.6 mmol) in EtOH or MeOH (3 mL) at room temperature for 4 h. The resulting precipitate was collected by filtration and washed with cold EtOH 2–3 times. The crude product was further purified by recrystallization (in EtOH or MeOH) or column chromatography (the mixtures of petroleum ether and ethyl acetate were used as eluents) to afford the pure corresponding compound.

**2′-Ethyl-3*a*′,6′,7′,8′,8*a*′,8*b*′-hexahydro-1′*H*-spiro[indoline-3,4′-pyrrolo [3,4-*a*]pyrrolizine]-1′,2,3′(2′*H*)-trione (7a).** White solid; 93% yield; >99:1 dr; mp 231–232 °C; IR (KBr) 750, 1138, 1350, 1375, 1474, 1618, 1690, 1717, 2365, 2884, 2978, 3088, 3148 cm^−1^; HRMS (ESI) calcd for C_18_H_19_N_3_O_3_ [M + H]^+^ 326.1499, found 326.1508; ^1^H-NMR (400 MHz, DMSO-*d*_6_): *δ* 10.55 (s, 1H, NH), 7.25 (t, *J* = 14.8 Hz, 1H, ArH), 6.94 (t, *J* = 15.2 Hz, 1H, ArH), 6.89 (d, *J* = 7.6 Hz, 1H, ArH), 6.84 (d, *J* = 7.6 Hz, 1H, ArH), 4.26–4.21 (m, 1H, CH), 3.58 (d, *J* = 7.6 Hz, 1H, CH), 3.49 (t, *J* = 16.0 Hz, 1H, CH), 3.45–3.43 (m, 2H, CH_2_), 2.41–2.29 (m, 2H, CH_2_), 1.94–1.75 (m, 4H, CH_2_), 1.11 (t, *J* = 14.4 Hz, 3H, CH_3_); ^13^C-NMR (400 MHz, DMSO-*d*_6_): *δ* 178.2, 177.1, 175.4, 143.0, 129.9, 127.4, 125.4, 121.6, 110.1, 69.1, 64.4, 55.5, 46.2, 45.3, 33.6, 25.9, 23.8, 13.0.

**2′-Ethyl-5-methyl-3*a*′,6′,7′,8′,8*a*′,8*b*′-hexahydro-1′*H*-spiro[indoline-3,4′-pyrrolo[3,4-*a*]pyrrolizine]-1′,2,3′(2′*H*)-trione (7b).** Brown solid; 88% yield; >99:1 dr; mp 240–241 °C; IR (KBr) 1219, 1339, 1491, 1624, 1695, 1711, 2995, 3177 cm^−1^; HRMS (ESI) calcd for C_19_H_21_N_3_O_3_ [M + H]^+^ 340.1656, found 340.1662; ^1^H-NMR (400 MHz, DMSO-*d*_6_): *δ* 10.61 (s, 1H, NH), 7.06–7.04 (m, 1H, ArH), 6.74 (d, *J* = 8.0 Hz, 1H, ArH), 6.66 (s, 1H, ArH), 4.22–4.17 (m, 1H, CH), 3.57 (d, *J* = 8.0 Hz, 1H, CH), 3.48 (d, *J* = 8.0 Hz, 1H, CH), 3.45–3.41 (m, 2H, CH_2_), 2.39–2.25 (m, 2H, CH_2_), 2.21 (m, 3H, CH_3_), 1.90–1.79 (m, 4H, CH_2_), 1.12 (t, *J* = 14.4 Hz, 3H, CH_3_); ^13^C-NMR (400 MHz, DMSO-*d*_6_): *δ* 178.3, 177.0, 175.4, 140.6, 130.2, 130.1, 127.7, 125.6, 109.9, 68.8, 64.5, 56.1, 46.0, 44.7, 33.6, 26.1, 23.8, 21.2, 13.1.

**2′-Ethyl-5-methoxy-3*a*′,6′,7′,8′,8*a*′,8*b*′-hexahydro-1′*H*-spiro[indoline-3,4′-pyrrolo[3,4-*a*]pyrrolizine]-1′,2,3′(2′*H*)-trione (7c).** White solid; 87% yield; 20:1 dr; mp 241–242 °C; IR (KBr) 1207, 1224, 1348, 1400, 1491, 1690, 1701, 2957 cm^−1^; HRMS (ESI) calcd for C_19_H_21_N_3_O_4_ [M + H]^+^ 356.1605, found 356.1604; ^1^H-NMR (400 MHz, DMSO-*d*_6_): *δ* 10.39 (s, 1H, NH), 6.86–6.83 (m, 1H, ArH), 6.76 (d, *J* = 8.8 Hz, 1H, ArH), 6.41 (d, *J* = 2.4 Hz, 1H, ArH), 4.24–4.19 (m, 1H, CH), 3.68 (s, 3H, OCH_3_), 3.59 (d, *J* = 7.6 Hz, 1H, CH), 3.48 (d, *J* = 8.0 Hz, 1H, CH), 3.47–3.42 (m, 2H, CH_2_), 2.38–2.27 (m, 2H, CH_2_), 1.86–1.80 (m, 4H, CH_2_), 1.11 (t, *J* = 14.4 Hz, 3H, CH_3_); ^13^C-NMR (400 MHz, DMSO-*d*_6_): *δ* 178.1, 177.0, 175.4, 154.7, 136.2, 126.8, 114.4, 114.3, 110.3, 69.0, 64.5, 56.0, 55.8, 45.9, 44.6, 33.6, 26.1, 23.7, 13.1.

**2′-Ethyl-5-fluoro-3*a*′,6′,7′,8′,8*a*′,8*b*′-hexahydro-1′*H*-spiro[indoline-3,4′-pyrrolo[3,4-*a*]pyrrolizine]-1′,2,3′(2′*H*)-trione (7d).** Orange solid; 90% yield; >99:1 dr; mp 225–226 °C; IR (KBr) 768, 1173, 1221, 1462, 1487, 1695, 1730, 1770, 2704, 2821, 2976 cm^−1^; HRMS (ESI) calcd for C_18_H_18_FN_3_O_3_ [M + H]^+^ 344.1405, found 344.1414; ^1^H-NMR (400 MHz, DMSO-*d*_6_): *δ* 10.62 (s, 1H, NH), 7.15–7.10 (m, 1H, ArH), 6.86–6.82 (m, 1H, ArH), 6.68–6.66 (m, 1H, ArH), 4.25–4.20 (m, 1H, CH), 3.62 (d, *J* = 7.6 Hz, 1H, CH), 3.49 (d, *J* = 8.0 Hz, 1H, CH), 3.47–3.42 (m, 2H, CH_2_), 2.40–2.27 (m, 2H, CH_2_), 1.88–1.81 (m, 4H, CH_2_), 1.10 (t, *J* = 14.4 Hz, 3H, CH_3_); ^13^C-NMR (400 MHz, DMSO-*d*_6_): *δ* 178.1, 176.9, 175.4, 159.0, 156.6, 139.2, 139.2, 127.2, 127.1, 116.6, 116.4, 115.0, 114.8, 110.9, 110.8, 68.9, 64.5, 55.8, 45.9, 44.9, 33.7, 25.9, 23.7, 13.1.

**2′-Ethyl-5-nitro-3*a*′,6′,7′,8′,8*a*′,8*b*′-hexahydro-1′*H*-spiro[indoline-3,4′-pyrrolo[3,4-*a*]pyrrolizine]-1′,2,3′(2′*H*)-trione (7e).** Khaki solid; 89% yield; >99:1 dr; mp 267–268 °C; IR (KBr) 1099, 1217, 1339, 1406, 1628, 1680, 1736, 2965, 3233 cm^−1^; HRMS (ESI) calcd for C_18_H_18_N_4_O_5_ [M + H]^+^ 371.1350, found 371.1356; ^1^H-NMR (400 MHz, DMSO-*d*_6_): *δ* 11.24 (s, 1H, NH), 8.20–8.18 (m, 1H, ArH), 7.60 (d, *J* = 2.4 Hz, 1H, ArH), 7.00 (d, *J* = 8.8 Hz, 1H, ArH), 4.16–4.11 (m, 1H, CH), 3.67 (d, *J* = 7.6 Hz, 1H, CH), 3.46 (d, *J* = 8.0 Hz, 1H, CH), 3.43–3.37 (m, 2H, CH_2_), 2.37–2.19 (m, 2H, CH_2_), 1.87–1.75 (m, 4H, CH_2_), 1.11 (t, *J* = 14.4 Hz, 3H, CH_3_); ^13^C-NMR (400 MHz, DMSO-*d*_6_): *δ* 178.4, 176.6, 175.4, 149.5, 142.2, 127.3, 126.5, 122.5, 110.6, 67.8, 64.8, 56.2, 45.5, 44.3, 33.7, 26.1, 23.6, 13.1.

**6-Chloro-2′-ethyl-3*a*′,6′,7′,8′,8*a*′,8*b*′-hexahydro-1′*H*-spiro[indoline-3,4′-pyrrolo[3,4-*a*]pyrrolizine]-1′,2,3′(2′*H*)-trione (7f).** White solid; 94% yield; 25:1 dr; mp 270–271 °C; IR (KBr) 806, 1219, 1346, 1400, 1466, 1616, 1694, 1719, 2932, 2982 cm^−1^; HRMS (ESI) calcd for C_18_H_18_ClN_3_O_3_ [M + H]^+^ 360.1109, found 360.1113; ^1^H-NMR (400 MHz, DMSO-*d*_6_): *δ* 10.72 (s, 1H, NH), 7.02–6.99 (m, 1H, ArH), 6.87 (t, *J* = 10.4 Hz, 2H, ArH), 4.24–4.19 (m, 1H, CH), 3.60 (d, *J* = 8.0 Hz, 1H, CH), 3.49 (d, *J* = 8.0 Hz, 1H, CH), 3.47–3.40 (m, 2H, CH_2_), 2.40–2.27 (m, 2H, CH_2_), 1.88–1.79 (m, 4H, CH_2_), 1.10 (t, *J* = 14.4 Hz, 3H, CH_3_); ^13^C-NMR (400 MHz, DMSO-*d*_6_): *δ* 178.1, 177.0, 175.4, 144.5, 134.3, 128.9, 124.3, 121.4, 110.2, 68.6, 64.5, 55.6, 46.1, 45.2, 33.6, 25.9, 23.8, 13.1.

**6-Bromo-2′-ethyl-3*a*′,6′,7′,8′,8*a*′,8*b*′-hexahydro-1′*H*-spiro[indoline-3,4′-pyrrolo[3,4-*a*]pyrrolizine]-1′,2,3′(2′*H*)-trione (7g).** White solid; 95% yield; >99:1 dr; mp 200–201 °C; IR (KBr) 758, 804, 1219, 1398, 1466, 1589, 1701, 1722, 2928, 2947, 2984, 3030, 3122 cm^−1^; HRMS (ESI) calcd for C_18_H_18_BrN_3_O_3_ [M + H]^+^ 404.0604, found 404.0625; ^1^H-NMR (400 MHz, DMSO-*d*_6_): *δ* 10.74 (s, 1H, NH), 7.14 (d, *J* = 8.4 Hz, 1H, ArH), 6.99 (s, 1H, ArH), 6.81 (d, *J* = 8.0 Hz, 1H, ArH), 4.23–4.18 (m, 1H, CH), 3.60 (d, *J* = 7.6 Hz, 1H, CH), 3.49 (d, *J* = 8.0 Hz, 1H, CH), 3.46–3.42 (m, 2H, CH_2_), 2.39–2.29 (m, 2H, CH_2_), 1.86–1.80 (m, 4H, CH_2_), 1.09 (t, *J* = 13.6 Hz, 3H, CH_3_); ^13^C-NMR (400 MHz, DMSO-*d*_6_): *δ* 178.0, 177.0, 175.4, 144.7, 129.2, 124.7, 124.3, 122.8, 113.0, 68.6, 64.5, 55.6, 46.0, 45.1, 33.7, 25.9, 23.8, 13.1.

**2′-Ethyl-7-fluoro-3*a*′,6′,7′,8′,8*a*′,8*b*′-hexahydro-1′*H*-spiro[indoline-3,4′-pyrrolo[3,4-*a*]pyrrolizine]-1′,2,3′(2′*H*)-trione (7h).** White solid; 76% yield; >99:1 dr; mp 210–211 °C; IR (KBr) 729, 1206, 1225, 1341, 1686, 1701, 2363, 2799, 2968, 2974 cm^−1^; HRMS (ESI) calcd for C_18_H_18_FN_3_O_3_ [M + H]^+^ 344.1405, found 344.1413; ^1^H-NMR (400 MHz, DMSO-*d*_6_): *δ* 11.09 (s, 1H, NH), 7.19 (t, *J* = 18.0 Hz, 1H, ArH), 6.99–6.94 (m, 1H, ArH), 6.72 (d, *J* = 7.2 Hz, 1H, ArH), 4.25–4.20 (m, 1H, CH), 3.64 (d, *J* = 8.0 Hz, 1H, CH), 3.51 (d, *J* = 7.6 Hz, 1H, CH), 3.48–3.41 (m, 2H, CH_2_), 2.40–2.28 (m, 2H, CH_2_), 1.86–1.79 (m, 4H, CH_2_), 1.10 (t, *J* = 14.0 Hz, 3H, CH_3_); ^13^C-NMR (400 MHz, DMSO-*d*_6_): *δ* 178.0, 176.9, 175.2, 148.0, 145.6, 130.2, 130.1, 128.4, 128.3, 123.4, 123.3, 122.4, 122.4, 117.0, 116.8, 69.0, 69.0, 64.5, 56.0, 46.0, 45.0, 33.6, 26.0, 23.9, 13.1.

**2′-Ethyl-5,7-dimethyl-3*a*′,6′,7′,8′,8*a*′,8*b*′-hexahydro-1′*H*-spiro[indoline-3,4′-pyrrolo[3,4-*a*]pyrrolizine]-1′,2,3′(2′*H*)-trione (7i).** Yellow solid; 79% yield; >99:1 dr; mp 227–228 °C; IR (KBr) 1221, 1342, 1400, 1624, 1697, 1719, 2851, 2918, 2957, 3198 cm^−1^; HRMS (ESI) calcd for C_20_H_23_N_3_O_3_ [M + H]^+^ 354.1812, found 354.1823; ^1^H-NMR (400 MHz, DMSO-*d*_6_): *δ* 10.40 (s, 1H, NH), 6.79 (s, 1H, ArH), 6.40 (s, 1H, ArH), 4.13 (t, *J* = 12.8 Hz, 1H, CH), 3.49 (d, *J* = 8.0 Hz, 1H, CH), 3.39 (d, *J* = 7.6 Hz, 1H, CH), 3.36–3.33 (m, 2H, CH_2_), 2.30–2.16 (m, 2H, CH_2_), 2.10 (s, 3H, CH_3_), 2.08 (s, 3H, CH_3_), 1.78–1.72 (m, 4H, CH_2_), 1.04 (t, *J* = 14.4 Hz, 3H, CH_3_); ^13^C-NMR (400 MHz, DMSO-*d*_6_): *δ* 178.8, 177.0, 175.4, 139.0, 131.6, 130.1, 125.3, 124.9, 119.0, 68.8, 64.5, 56.3, 45.9, 44.5, 33.6, 26.1, 23.7, 21.1, 16.7, 13.1.

**2′-Ethyl-1-methyl-3*a*′,6′,7′,8′,8*a*′,8*b*′-hexahydro-1′*H*-spiro[indoline-3,4’-pyrrolo[3,4-*a*]pyrrolizine]-1′,2,3′(2′*H*)-trione (7j).** White solid; 94% yield; >99:1 dr; mp 247–249 °C; IR (KBr) 758, 1341, 1398, 1612, 1697, 2365, 2822, 2963 cm^−1^; HRMS (ESI) calcd for C_19_H_21_N_3_O_3_ [M + H]^+^ 340.1656, found 340.1664; ^1^H-NMR (400 MHz, DMSO-*d*_6_): *δ* 7.39–7.35 (m, 1H, ArH), 7.05–7.01 (m, 2H, ArH), 6.96 (t, *J* = 7.6 Hz, 1H, ArH), 4.29–4.24 (m, 1H, CH), 3.58 (d, *J* = 8.0 Hz, 1H, CH), 3.51 (t, *J* = 15.6 Hz, 1H, CH), 3.47–42 (m, 2H, CH), 3.36 (s, 3H, NCH_3_), 2.40–2.23 (m, 2H, CH_2_), 1.95–1.75 (m, 4H, CH_2_), 1.11 (t, *J* = 14.4 Hz, 3H, CH_3_); ^13^C-NMR (400 MHz, DMSO-*d*_6_): *δ* 177.1, 176.2, 175.4, 144.5, 130.1, 127.2, 124.6, 122.3, 109.1, 68.8, 64.5, 55.5, 46.3, 45.5, 33.6, 26.3, 25.8, 23.8, 13.0.

**2′-Ethyl-1-phenyl-3*a*′,6′,7′,8′,8*a*′,8*b*′-hexahydro-1′*H*-spiro[indoline-3,4′-pyrrolo[3,4-*a*]pyrrolizine]-1′,2,3′(2′*H*)-trione (7k).** Bright-yellow solid; 92% yield; >99:1 dr; mp 239–241 °C; IR (KBr) 750, 1225, 1373, 1400, 1499, 1611, 1699, 2940, 2955 cm^−1^; HRMS (ESI) calcd for C_24_H_23_N_3_O_3_ [M + H]^+^ 402.1812, found 402.1810; ^1^H-NMR (400 MHz, DMSO-*d*_6_): *δ* 7.61–7.51 (m, 2H, ArH), 7.49–7.45 (m, 3H, ArH), 7.33–7.29 (m, 1H, ArH), 7.11–7.04 (m, 2H, ArH), 6.74 (d, *J* = 8.0 Hz, 1H, ArH), 4.30–4.25 (m, 1H, CH), 3.80 (d, *J* = 7.6 Hz, 1H, CH), 3.45 (d, *J* = 8.0 Hz, 1H, CH), 3.52–3.46 (m, 2H, CH_2_), 2.48–2.39 (m, 2H, CH_2_), 1.93–1.83 (m, 4H, CH_2_), 1.17 (t, *J* = 14.4 Hz, 3H, CH_3_); ^13^C-NMR (400 MHz, DMSO-*d*_6_): *δ* 177.0, 175.6, 175.4, 144.3, 134.2, 130.1, 130.1, 130.1, 128.8, 127.8, 127.4, 127.4, 124.5, 122.8, 109.4, 68.7, 64.5, 55.7, 46.1, 45.1, 33.7, 25.9, 23.8, 13.1.

**3*a*′,6′,7′,8′,8*a*′,8*b*′-Hexahydro-1′*H*-spiro[indoline-3,4′-pyrrolo[3,4-*a*]pyrrolizine]-1′,2,3′(2′*H*)-trione (7l).** Light-yellow solid; 89% yield; >99:1 dr; mp >300 °C; IR (KBr) 751, 1194, 1474, 1701, 2345, 2365, 2965, 3086, 3186 cm^−1^; HRMS (ESI) calcd for C_16_H_15_N_3_O_3_ [M + H]^+^ 298.1186, found 298.1187; ^1^H-NMR (400 MHz, DMSO-*d*_6_): *δ* 11.33 (s, 1H, NH), 10.52 (s, 1H, NH), 7.26–7.22 (m, 1H, ArH), 6.98–6.91 (m, 2H, ArH), 6.83 (d, *J* = 7.6 Hz, 1H, ArH), 4.19–4.14 (m, 1H, CH), 3.53 (d, *J* = 8.0 Hz, 1H, CH), 3.45 (t, *J* = 15.6 Hz, 1H, CH), 2.46–2.26 (m, 2H, CH_2_), 1.99–1.81 (m, 4H, CH_2_); ^13^C-NMR (400 MHz, DMSO-*d*_6_): *δ* 178.9, 178.3, 177.1, 142.9, 129.9, 127.2, 125.7, 121.6, 110.0, 68.5, 64.4, 57.2, 47.2, 44.7, 26.1, 23.7.

**2′-Phenyl-3*a*′,6′,7′,8′,8*a*′,8*b*′-hexahydro-1′*H*-spiro[indoline-3,4′-pyrrolo[3,4-*a*]pyrrolizine]-1′,2,3′(2′*H*)-trione (7m).** Light-yellow solid; 90% yield; >99:1 dr; mp 230–232 °C; IR (KBr) 748, 1194, 1219, 1387, 1474, 1707, 1740, 2363, 2828, 2957, 3065 cm^−1^; HRMS (ESI) calcd for C_22_H_19_N_3_O_3_ [M + H]^+^ 374.1499, found 374.1502; ^1^H-NMR (400 MHz, DMSO-*d*_6_): *δ* 10.60 (s, 1H, NH), 7.54 (t, *J* = 15.2 Hz, 2H, ArH), 7.46 (t, *J* = 14.8 Hz, 1H, ArH), 7.30–7.24 (m, 3H, ArH), 7.02 (d, *J* = 7.2 Hz, 1H, ArH), 6.95 (t, *J* = 14.8 Hz, 1H, ArH), 6.86 (d, *J* = 8.0 Hz, 1H, ArH), 4.38–4.32 (m, 1H, CH), 3.74 (t, *J* = 18.0 Hz, 1H, CH), 3.69 (d, *J* = 8.0 Hz, 1H, CH), 2.56–2.39 (m, 2H, CH_2_), 1.96–1.82 (m, 4H, CH_2_); ^13^C-NMR (400 MHz, DMSO-*d*_6_): *δ* 178.2, 176.5, 174.7, 143.1, 132.8, 130.0, 129.6, 128.9, 128.9, 127.5, 127.4, 127.4, 125.3, 121.7, 110.2, 69.7, 64.8, 55.8, 46.7, 45.9, 25.9, 24.1.

**2′-Benzyl-3*a*′,6′,7′,8′,8*a*′,8*b*′-hexahydro-1′*H*-spiro[indoline-3,4′-pyrrolo[3,4-*a*]pyrrolizine]-1′,2,3′(2′*H*)-trione (7n).** Light-yellow solid; 87% yield; >99:1 dr; mp 269–270 °C; IR (KBr) 758, 1344, 1400, 1476, 1701, 1728, 2363, 2681, 2828, 2940, 3065 cm^−1^; HRMS (ESI) calcd for C_23_H_21_N_3_O_3_ [M + H]^+^ 388.1656, found 388.1660; ^1^H-NMR (400 MHz, DMSO-*d*_6_): *δ* 10.53 (s, 1H, NH), 7.42–7.32 (m, 5H, ArH), 7.22–7.18 (m, 1H, ArH), 6.81 (d, *J* = 8.0 Hz, 1H, ArH), 6.74–6.70 (m, 1H, ArH), 6.40 (d, *J* = 7.6 Hz, 1H, ArH), 4.66–4.53 (m, 2H, CH_2_), 4.28–4.23 (m, 1H, CH), 3.62 (d, *J* = 7.6 Hz, 1H, CH), 3.56 (t, *J* = 15.6 Hz, 1H, CH), 2.18–2.14 (m, 2H, CH_2_), 1.94–1.89 (m, 2H, CH_2_), 1.88–1.83 (m, 2H, CH_2_); ^13^C-NMR (400 MHz, DMSO-*d*_6_): *δ* 178.1, 177.1, 175.3, 143.0, 136.1, 129.8, 129.0, 129.0, 128.8, 128.8, 128.3, 127.6, 125.2, 121.4, 110.0, 69.2, 64.4, 55.4, 46.5, 45.4, 42.2, 25.6, 23.6.

**6-Chloro-3*a*′,6′,7′,8′,8*a*′,8*b*′-hexahydro-1′*H*-spiro[indoline-3,4′-pyrrolo[3,4-*a*]pyrrolizine]-1′,2,3′(2′*H*)-trione (7o).** White solid; 83% yield; >99:1 dr; mp 279–281 °C; IR (KBr) 761, 1192, 1327, 1458, 1618, 1717, 2710, 2922, 3134, 3192 cm^−1^; HRMS (ESI) calcd for C_16_H_14_ClN_3_O_3_ [M + H]^+^ 332.0796, found 332.0796; ^1^H-NMR (400 MHz, DMSO-*d*_6_): *δ* 11.36 (s, 1H, NH), 10.68 (s, 1H, NH), 7.02–6.99 (m, 1H, ArH), 6.96 (d, *J* = 8.0 Hz, 1H, ArH), 6.86 (d, *J* = 1.6 Hz, 1H, ArH), 4.17–4.12 (m, 1H, CH), 3.56 (d, *J* = 7.6 Hz, 1H, CH), 3.45 (t, *J* = 15.6 Hz, 1H, CH), 2.45–2.24 (m, 2H, CH_2_), 1.89–1.80 (m, 4H, CH_2_); ^13^C-NMR (400 MHz, DMSO-*d*_6_): *δ* 178.7, 178.2, 177.0, 144.4, 134.3, 128.6, 124.6, 121.4, 110.1, 68.1, 64.5, 57.3, 47.0, 44.5, 26.2, 23.7.

**6-Bromo-3*a*′,6′,7′,8′,8*a*′,8*b*′-hexahydro-1′*H*-spiro[indoline-3,4′-pyrrolo[3,4-*a*]pyrrolizine]-1′,2,3′(2′*H*)-trione (7p).** Light-pink solid; 87% yield; >99:1 dr; mp >300 °C; IR (KBr) 1120, 1321, 1449, 1609, 1709, 2828, 3063, 3132, 3163 cm^−1^; HRMS (ESI) calcd for C_16_H_14_BrN_3_O_3_ [M - H^+^]^-^ 374.0146, found 374.0138; ^1^H-NMR (400 MHz, DMSO-*d*_6_): *δ* 11.39 (s, 1H, NH), 10.70 (s, 1H, NH), 7.16–7.13 (m, 1H, ArH), 6.98 (d, *J* = 1.6 Hz, 1H, ArH), 6.89 (d, *J* = 8.0 Hz, 1H, ArH), 4.16–4.11 (m, 1H, CH), 3.56 (d, *J* = 8.0 Hz, 1H, CH), 3.44 (t, *J* = 15.6 Hz, 1H, CH), 2.44–2.25 (m, 2H, CH_2_), 1.91–1.81 (m, 4H, CH_2_); ^13^C-NMR (400 MHz, DMSO-*d*_6_): *δ* 178.8, 178.1, 177.0, 144.6, 128.9, 125.0, 124.4, 122.7, 112.9, 68.1, 64.5, 57.3, 47.0, 44.5, 26.2, 23.7.

**5-Fluoro-3*a*′,6′,7′,8′,8*a*′,8*b*′-hexahydro-1′*H*-spiro[indoline-3,4′-pyrrolo[3,4-*a*]pyrrolizine]-1′,2,3′(2′*H*)-trione (7q).** White solid; 91% yield; >99:1 dr; mp 254–256 °C; IR (KBr) 814, 899, 1194, 1321, 1489, 1717, 2706, 2967, 3088, 3184 cm^−1^; HRMS (ESI) calcd for C_16_H_14_FN_3_O_3_ [M - H]^-^ 314.0946, found 314.0945; ^1^H-NMR (400 MHz, DMSO-*d*_6_): *δ* 11.38 (s, 1H, NH), 10.57 (s, 1H, NH), 7.13–7.08 (m, 1H, ArH), 6.85–6.81 (m, 1H, ArH), 6.76–6.73 (m, 1H, ArH), 4.18–4.13 (m, 1H, CH), 3.57 (d, *J* = 7.6 Hz, 1H, CH), 3.44 (t, *J* = 15.6 Hz, 1H, CH), 2.46–2.42 (m, 1H, CH_2_), 2.28–2.24 (m, 1H, CH_2_), 1.93–1.79 (m, 4H, CH_2_); ^13^C-NMR (400 MHz, DMSO-*d*_6_): *δ* 178.7, 178.2, 177.0, 159.1, 156.7, 139.1, 139.1, 127.6, 127.5, 116.5, 116.3, 114.7, 114.4, 110.8, 110.8, 68.4, 68.4, 64.5, 57.5, 46.8, 44.2, 26.2, 23.5.

**5-Methoxy-3*a*′,6′,7′,8′,8*a*′,8*b*′-hexahydro-1′*H*-spiro[indoline-3,4′-pyrrolo[3,4-*a*]pyrrolizine]-1′,2,3′(2′*H*)-trione (7r).** White solid; 84% yield; 50:1 dr; mp 291–292 °C; IR (KBr) 1209, 1491, 1717, 2365, 2959, 3254 cm^−1^; HRMS (ESI) calcd for C_17_H_17_N_3_O_4_ [M - H]^-^ 326.1146, found 326.1158; ^1^H-NMR (400 MHz, DMSO-*d*_6_): *δ* 11.34 (s, 1H, NH), 10.35 (s, 1H, NH), 6.85–6.82 (m, 1H, ArH), 6.74 (d, *J* = 8.4 Hz, 1H, ArH), 6.53 (d, *J* = 2.4 Hz, 1H, ArH), 4.17–4.12 (m, 1H, CH), 3.68 (s, 3H, OCH_3_), 3.54 (d, *J* = 7.6 Hz, 1H, CH), 3.44 (t, *J* = 15.2 Hz, 1H, CH), 2.44–2.24 (m, 2H, CH_2_), 1.92–1.78 (m, 4H, CH_2_); ^13^C-NMR (400 MHz, DMSO-*d*_6_): *δ* 178.8, 178.2, 177.0, 154.7, 136.2, 127.1, 114.2, 114.2, 110.2, 68.5, 64.5, 57.7, 55.9, 46.9, 44.2, 26.3, 23.7.

**5-Nitro-3*a*′,6′,7′,8′,8*a*′,8*b*′-hexahydro-1′*H*-spiro[indoline-3,4′-pyrrolo[3,4-*a*]pyrrolizine]-1′,2,3′(2′*H*)-trione (7s).** Light-yellow solid; 79% yield; 17:1 dr; mp 255–256 °C; IR (KBr) 1194, 1341, 1522, 1624, 1717, 2365, 2967, 3231 cm^−1^; HRMS (ESI) calcd for C_16_H_14_N_4_O_5_ [M - H]^-^ 341.0891, found 341.0898; ^1^H-NMR (400 MHz, DMSO-*d*_6_): *δ* 11.55 (s, 1H, NH), 11.27 (s, 1H, NH), 8.27–8.24 (m, 1H, ArH), 7.77 (d, *J* = 2.0 Hz, 1H, ArH), 7.06 (d, *J* = 8.8 Hz, 1H, ArH), 4.19–4.13 (m, 1H, CH), 3.68 (d, *J* = 7.6 Hz, 1H, CH), 3.48 (t, *J* = 15.6 Hz, 1H, CH), 2.49–2.24 (m, 2H, CH_2_), 1.97–1.79 (m, 4H, CH_2_); ^13^C-NMR (400 MHz, DMSO-*d*_6_): *δ* 178.5, 178.4, 177.1, 149.5, 142.2, 127.2, 126.6, 122.5, 110.5, 67.6, 64.7, 57.5, 46.6, 44.0, 26.2, 23.5.

**2′-Benzyl-6-chloro-3*a*′,6′,7′,8′,8*a*′,8*b*′-hexahydro-1′*H*-spiro[indoline-3,4′-pyrrolo[3,4-*a*]pyrrolizine]-1′,2,3′(2′*H*)-trione (7t).** White solid; 80% yield; >99:1 dr; mp 211–213 °C; IR (KBr) 704, 1167, 1339, 1395, 1616, 1697, 1740, 2363, 2693, 2808, 2965 cm^−1^; HRMS (ESI) calcd for C_23_H_20_ClN_3_O_3_ [M - H]^-^ 420.1120, found 420.1125; ^1^H-NMR (400 MHz, DMSO-*d*_6_): *δ* 10.71 (s, 1H, NH), 7.42–7.38 (m, 2H, ArH), 7.35–7.32 (m, 3H, ArH), 6.83 (d, *J* = 2.0 Hz, 1H, ArH), 6.76–6.74 (m, 1H, ArH), 6.33 (d, *J* = 8.0 Hz, 1H, ArH), 4.64 (d, *J* = 14.4 Hz, 1H, CH), 4.54 (d, *J* = 14.4 Hz, 1H, CH_2_), 4.25–4.20 (m, 1H, CH), 3.65 (d, *J* = 7.6 Hz, 1H, CH), 3.56 (t, *J* = 15.6 Hz, 1H, CH), 2.18–2.13 (m, 2H, CH_2_), 1.90–1.86 (m, 2H, CH_2_), 1.84–1.73 (m, 2H, CH_2_); ^13^C-NMR (400 MHz, DMSO-*d*_6_): *δ* 178.0, 176.9, 175.2, 144.5, 136.1, 134.3, 129.0, 129.0, 128.9, 128.9, 128.3, 128.3, 124.1, 121.1, 110.2, 68.7, 64.6, 55.5, 46.2, 45.2, 42.3, 25.7, 23.6.

**2′-Benzyl-6-bromo-3*a*′,6′,7′,8′,8*a*′,8*b*′-hexahydro-1′*H*-spiro[indoline-3,4′-pyrrolo[3,4-*a*]pyrrolizine]-1′,2,3′(2′*H*)-trione (7u).** White solid; 81% yield; >99:1 dr; mp 270–272 °C; IR (KBr) 704, 1167, 1339, 1395, 1612, 1697, 1736, 2363, 2965 cm^−1^; HRMS (ESI) calcd for C_23_H_20_BrN_3_O_3_ [M + H]^+^ 466.0761, found 466.0766; ^1^H-NMR (400 MHz, DMSO-*d*_6_): *δ* 10.69 (s, 1H, NH), 7.40 (t, *J* = 14.8 Hz, 2H, ArH), 7.35 (t, *J* = 10.8 Hz, 3H, ArH), 6.96 (d, *J* = 1.6 Hz, 1H, ArH), 6.90–6.88 (m, 1H, ArH), 6.26 (d, *J* = 8.0 Hz, 1H, ArH), 4.64 (d, *J* = 14.8 Hz, 1H, CH_2_), 4.54 (d, *J* = 14.4 Hz, 1H, CH_2_), 4.25–4.19 (m, 1H, CH), 3.65 (d, *J* = 7.6 Hz, 1H, CH), 3.56 (t, *J* = 15.6 Hz, 1H, CH), 2.17–2.13 (m, 2H, CH_2_), 1.89–1.83 (m, 2H, CH_2_), 1.82–1.73 (m, 2H, CH_2_); ^13^C-NMR (400 MHz, DMSO-*d*_6_): *δ* 177.9, 176.9, 175.2, 144.7, 136.1, 129.2, 129.2, 129.0, 129.0, 128.9, 128.3, 124.6, 124.0, 122.7, 112.9, 68.8, 64.6, 55.5, 46.2, 45.1, 42.3, 25.7, 23.6.

**2′-Benzyl-5-fluoro-3*a*′,6′,7′,8′,8*a*′,8*b*′-hexahydro-1′*H*-spiro[indoline-3,4′-pyrrolo[3,4-*a*]pyrrolizine]-1′,2,3′(2′*H*)-trione (7v).** White solid; 89% yield; >99:1 dr; mp >300 °C; IR (KBr) 707, 1159, 1202, 1391, 1489, 1701, 1730, 2818, 2856, 2967, 3032 cm^−1^; HRMS (ESI) calcd for C_23_H_20_FN_3_O_3_ [M + H]^+^ 406.1561, found 406.1564; ^1^H-NMR (400 MHz, DMSO-*d*_6_): *δ* 10.62 (s, 1H, NH), 7.40–7.31 (m, 5H, ArH), 7.08–7.03 (m, 1H, ArH), 6.83–6.79 (m, 1H, ArH), 6.19–6.16 (m, 1H, ArH), 4.67 (d, *J* = 14.8 Hz, 1H, CH_2_), 4.55 (d, *J* = 14.4 Hz, 1H, CH_2_), 4.28–4.23 (m, 1H, CH), 3.67 (d, *J* = 8.0 Hz, 1H, CH), 3.55 (t, *J* = 15.6 Hz, 1H, CH), 2.16 (t, *J* = 14.8 Hz, 2H, CH_2_), 1.88–1.82 (m, 2H, CH_2_), 1.80–1.73 (m, 2H, CH_2_); ^13^C-NMR (400 MHz, DMSO-*d*_6_): *δ* 177.9, 176.9, 175.3, 158.8, 156.5, 139.2, 136.2, 129.1, 129.1, 128.6, 128.6, 128.3, 127.0, 126.9, 116.5, 116.3, 115.2, 115.0, 110.8, 110.7, 69.1, 64.5, 55.6, 46.0, 44.9, 42.3, 25.7, 23.4.

**2′-Benzyl-5-methoxy-3*a*′,6′,7′,8′,8*a*′,8*b*′-hexahydro-1′*H*-spiro[indoline-3,4′-pyrrolo[3,4-*a*]pyrrolizine]-1′,2,3′(2′*H*)-trione (7w).** White solid; 87% yield; >99:1 dr; mp 211–212 °C; IR (KBr) 742, 1217, 1395, 1491, 1688, 1717, 2857, 2967, 3032, 3177 cm^−1^; HRMS (ESI) calcd for C_24_H_23_N_3_O_4_ [M + H]^+^ 418.1761, 418.1757; ^1^H-NMR (400 MHz, DMSO-*d*_6_): *δ* 10.39 (s, 1H, NH), 7.39–7.28 (m, 5H, ArH), 6.81–6.78 (m, 1H, ArH), 6.73 (d, *J* = 8.8 Hz, 1H, ArH), 6.18 (d, *J* = 2.4 Hz, 1H, ArH), 4.62 (d, *J* = 4.4 Hz, 2H, CH_2_), 4.31–4.26 (m, 1H, CH), 3.64 (d, *J* = 7.6 Hz, 1H, CH_2_), 3.55 (t, *J* = 16.0 Hz, 1H, CH_2_), 3.45 (s, 3H, OCH_3_), 2.19 (t, *J* = 13.2 Hz, 2H, CH_2_), 1.88–1.84 (m, 2H, CH_2_), 1.80–1.72 (m, 2H, CH_2_); ^13^C-NMR (400 MHz, DMSO-*d*_6_): *δ* 177.9, 177.1, 175.3, 154.5, 136.2, 136.2, 129.1, 129.1, 128.2, 128.2, 128.1, 126.4, 114.9, 114.3, 110.3, 69.3, 64.3, 55.6, 55.6, 46.1, 44.9, 42.1, 25.7, 23.6.

**2′-Benzyl-5-nitro-3*a*′,6′,7′,8′,8*a*′,8*b*′-hexahydro-1′*H*-spiro[indoline-3,4′-pyrrolo[3,4-*a*]pyrrolizine]-1′,2,3′(2′*H*)-trione (7x).** White solid; 85% yield; >99:1 dr; mp 201–202 °C; IR (KBr) 739, 1339, 1398, 1522, 1624, 1686, 1734, 2372, 2947, 3128 cm^−1^; HRMS (ESI) calcd for C_23_H_20_N_4_O_5_ [M - H]^-^ 431.1361, found 431.1379; ^1^H-NMR (400 MHz, DMSO-*d*_6_): *δ* 11.33 (s, 1H, NH), 8.25 (d, *J* = 2.4 Hz, 1H, ArH), 8.23 (d, *J* = 2.4 Hz, 1H, ArH), 7.58–7.38 (m, 4H, ArH), 7.35–7.31 (m, 1H, ArH), 7.05 (d, *J* = 8.4 Hz, 1H, ArH), 4.67–4.57 (m, 2H, CH_2_), 4.31–4.25 (m, 1H, CH), 3.78 (d, *J* = 8.0 Hz, 1H, CH), 3.58 (t, *J* = 16.0 Hz, 1H, CH), 2.22–2.07 (m, 2H, CH_2_), 1.86–1.79 (m, 2H, CH_2_), 1.69–1.57 (m, 2H, CH_2_); ^13^C-NMR (400 MHz, DMSO-*d*_6_): *δ* 178.1, 176.8, 175.4, 149.7, 142.0, 135.9, 129.1, 129.1, 128.6, 128.6, 128.3, 127.3, 125.8, 123.4, 110.5, 68.9, 64.6, 54.9, 46.3, 45.7, 42.4, 25.3, 23.6.

**7′-Ethyl-2′,3′,8*a*′,8*b*′-tetrahydrospiro[indoline-3,5′-pyrrolo[3′,4′:3,4]pyrrolo[2,1-*b*]thiazole]-2,6′,8′(5*a*′*H*,7′*H*)-trione (7y).** Yellow solid; 84% yield; >99:1 dr; mp 199–201 °C; IR (KBr) 752, 1227, 1686, 1701, 2365, 2976, 3265 cm^−1^; HRMS (ESI) calcd for C_17_H_17_N_3_O_3_S [M + H]^+^ 344.1063, found 344.1069; ^1^H-NMR (400 MHz, DMSO-*d*_6_): *δ* 10.69 (s, 1H, NH), 7.32–7.24 (m, 1H, ArH), 6.98–6.94 (m, 1H, ArH), 6.86–6.80 (m, 2H, ArH), 4.44–4.38 (m, 1H, CH), 3.76 (d, *J* = 8.0 Hz, 1H, CH), 3.68–3.64 (m, 1H, CH_2_), 3.48–3.42 (m, 2H, CH_2_), 3.38–3.36 (m, 1H, CH_2_), 3.19 (d, *J* = 6.0 Hz, 1H, CH), 3.02–2.98 (m, 1H, CH_2_), 2.77–2.72 (m, 1H, CH_2_), 1.12 (t, *J* = 14.4 Hz, 3H, CH_3_); ^13^C-NMR (400 MHz, DMSO-*d*_6_): *δ* 177.6, 175.9, 174.6, 142.8, 130.3, 126.5, 124.6, 122.2, 110.4, 68.0, 67.3, 56.9, 45.8, 44.2, 33.9, 29.7, 13.4.

**Ethyl 7″ -bromo-2,2″-dioxo-5′,6′,7′,7*a*′-tetrahydro-1′*H*-dispiro[indoline-3,2′-pyrrolizine-3′,3″-indoline]-1′-carboxylate (8a).** White solid; 87% yield; >99:1 dr; mp >300 °C; IR (KBr) 1234, 1479, 1631, 1724, 2378, 3279 cm^−1^; HRMS (ESI) calcd for C_24_H_22_BrN_3_O_4_ [M + H]^+^ 496.0866, found 496.0869; ^1^H-NMR (400 MHz, DMSO-*d*_6_): *δ* 10.77 (s, 1H, NH), 10.30 (s, 1H, NH), 7.63 (d, *J* = 7.2 Hz, 1H, ArH), 7.24–7.21 (m, 2H, ArH), 7.07 (t, *J* = 14.8 Hz, 1H, ArH), 6.66 (d, *J* = 7.6 Hz, 1H, ArH), 6.45 (t, *J* = 15.6 Hz, 1H, ArH), 6.22 (d, *J* = 7.6 Hz, 1H, ArH), 4.37 (s, 2H, CH_2_), 3.84–3.76 (m, 1H, CH_2_), 3.68–3.60 (m, 1H, CH_2_), 2.62 (t, *J* = 15.2 Hz, 1H, CH), 2.46 (d, *J* = 6.8 Hz, 1H, CH), 2.21–2.15 (m, 2H, CH_2_), 2.13–2.03 (m, 2H, CH_2_), 0.68 (t, *J* = 14.4 Hz, 3H, CH_3_); ^13^C-NMR (100 MHz, DMSO-*d*_6_): *δ* 177.9, 173.5, 169.9, 142.9, 142.2, 132.7, 129.6, 127.8, 127.3, 126.7, 124.7, 122.6, 121.5, 109.7, 102.0, 77.7, 66.5, 66.1, 60.3, 51.1, 47.2, 31.4, 30.5, 13.7.

**Ethyl 7-bromo-2,2″-dioxo-2′,3′,7′,7*a*′-tetrahydrodispiro[indoline-3,5′-pyrrolo[2,1-*b*]thiazole-6′,3″-indoline]-7′-carboxylate (8b).** White solid; 85% yield; 33:1 dr; mp >300 °C; IR (KBr) 1221, 1465, 1644, 1718, 2385, 3274 cm^−1^; HRMS (ESI) calcd for C_23_H_20_BrN_3_O_4_S [M + H]^+^ 514.0431, found 514.0435; ^1^H-NMR (400 MHz, DMSO-*d*_6_): *δ* 10.41 (s, 1H, NH), 10.31 (s, 1H, NH), 7.59 (d, *J* = 7.6 Hz, 1H, ArH), 7.44–7.39 (m, 2H, ArH), 7.18–7.14 (m, 1H, ArH), 6.95–6.89 (m, 2H, ArH), 6.69 (d, *J* = 7.6 Hz, 1H, ArH), 4.94–4.89 (m, 1H, CH), 3.87 (d, *J* = 8.8 Hz, 1H, CH), 3.77–3.71 (m, 2H, CH_2_), 3.69–3.53 (m, 2H, CH_2_), 3.24–3.11 (m, 2H, CH_2_), 0.60 (t, *J* = 15.0 Hz, 3H, CH_3_); ^13^C-NMR (100 MHz, DMSO-*d*_6_): *δ* 174.9, 174.8, 169.0, 142.2, 141.6, 132.9, 129.3, 128.6, 128.0, 126.2, 126.2, 122.6, 121.1, 109.4, 102.2, 76.5, 68.5, 66.0, 60.6, 54.2, 49.8, 35.2, 13.6.

**Ethyl 5-methyl-2,2″-dioxo-5′,6′,7′,7*a*′-tetrahydro-1′*H*-dispiro[indoline-3,2′-pyrrolizine-3′,3″-indoline]-1′-carboxylate (8c).** White solid; 91% yield; 17:1 dr; mp >300 °C; IR (KBr) 1200, 1342, 1474, 1618, 1719, 2345, 3119, 3277 cm^−1^; HRMS (ESI) calcd for C_25_H_25_N_3_O_4_ [M + H]^+^ 432.1918, found 432.1924; ^1^H-NMR (400 MHz, DMSO-*d*_6_): *δ* 10.18 (s, 1H, NH), 10.11 (s, 1H, NH), 7.53 (d, *J* = 7.6 Hz, 1H, ArH), 7.19–7.15 (m, 2H, ArH), 6.95–6.88 (m, 2H, ArH), 6.61 (d, *J* = 7.6 Hz, 1H, ArH), 6.49 (d, *J* = 8.0 Hz, 1H, ArH), 4.94–4.89 (m, 1H, CH), 3.74–3.69 (m, 2H, CH_2_), 3.68–3.58 (m, 1H, CH_2_), 3.43–3.39 (m, 1H, CH_2_), 2.59 (d, *J* = 6.8 Hz, 1H, CH), 2.18 (s, 3H, CH_3_), 2.16–2.01 (m, 2H, CH_2_), 1.84–1.76 (m, 2H, CH_2_), 0.57 (t, *J* = 14.0 Hz, 3H, CH_3_); ^13^C-NMR (100 MHz, DMSO-*d*_6_): *δ* 178.6, 176.2, 170.1, 143.0, 140.2, 129.9, 129.6, 129.0, 128.7, 128.5, 127.0, 125.2, 121.3, 109.6, 108.7, 77.6, 66.8, 65.7, 60.2, 57.7, 47.6, 30.0, 26.3, 21.4, 13.7.

**Ethyl 5,7-dimethyl-2,2″-dioxo-5′,6′,7′,7*a*′-tetrahydro-1′*H*-dispiro[indoline-3,2′-pyrrolizine-3′,3″-indoline]-1′-carboxylate (8d).** White solid; 94% yield; 50:1 dr; mp >300 °C; IR (KBr) 679, 750, 1115, 1202, 1325, 1474, 1616, 1715, 2961, 3092, 3157, 3308 cm^−1^; HRMS (ESI) calcd for C_26_H_27_N_3_O_4_ [M + H]^+^ 446.2074, found 446.2095; ^1^H-NMR (400 MHz, DMSO-*d*_6_): *δ* 10.24 (s, 1H, NH), 10.11 (s, 1H, NH), 7.54 (d, *J* = 7.6 Hz, 1H, ArH), 7.17 (t, *J* = 16.8 Hz, 1H, ArH), 7.00 (s, 1H, ArH), 6.93 (t, *J* = 14.8 Hz, 1H, ArH), 6.72 (s, 1H, ArH), 6.62 (d, *J* = 7.6 Hz, 1H, ArH), 4.87 (s, 1H, CH), 3.73–3.67 (m, 2H, CH_2_), 3.60 (d, *J* = 8.8 Hz, 2H, CH_2_), 3.35 (d, *J* = 7.6 Hz, 2H, CH_2_), 2.54 (d, *J* = 22.8 Hz, 1H, CH), 2.15 (s, 3H, CH_3_), 1.99 (s, 3H, CH_3_), 1.81 (s, 2H, CH_2_), 0.55 (t, *J* = 13.2 Hz, 3H, CH_3_); ^13^C-NMR (100 MHz, DMSO-*d*_6_): *δ* 178.7, 176.2, 170.1, 142.9, 138.8, 130.5, 129.8, 129.4, 128.9, 126.6, 125.9, 125.3, 121.2, 117.7, 109.7, 77.4, 66.9, 65.6, 60.1, 57.7, 47.5, 30.1, 26.6, 21.3, 16.7, 13.6.

**Ethyl 5-methoxy-2,2″-dioxo-5′,6′,7′,7*a*′-tetrahydro-1′*H*-dispiro[indoline-3,2′-pyrrolizine-3′,3″-indoline]-1′-carboxylate (8e).** White solid; 90% yield; 17:1 dr; mp >300 °C; IR (KBr) 812, 1209, 1474, 1709, 1719, 2345, 3275 cm^−1^; HRMS (ESI) calcd for C_25_H_25_N_3_O_5_ [M + H]^+^ 448.1867, found 448.1879; ^1^H-NMR (400 MHz, DMSO-*d*_6_): *δ* 10.18 (s, 1H, NH), 10.17 (s, 1H, NH), 7.54 (d, *J* = 7.6 Hz, 1H, ArH), 7.17 (t, *J* = 14.8 Hz, 1H, ArH), 6.95–6.68 (m, 2H, ArH), 6.66 (d, *J* = 1.2 Hz, 1H, ArH), 6.61 (d, *J* = 7.6 Hz, 1H, ArH), 6.50 (d, *J* = 8.4 Hz, 1H, ArH), 4.96–4.91 (m, 1H, CH), 3.76–3.64 (m, 2H, CH_2_), 3.59 (s, 3H, OCH_3_), 3.50–3.43 (m, 2H, CH_2_), 2.60 (d, *J* = 6.8 Hz, 1H, CH), 2.18–2.11 (m, 2H, CH_2_), 2.09–1.74 (m, 2H, CH_2_), 0.60 (t, *J* = 14.4 Hz, 3H, CH_3_); ^13^C-NMR (100 MHz, DMSO-*d*_6_): *δ* 178.8, 176.2, 170.2, 154.4, 143.0, 136.0, 130.0, 128.7, 128.1, 125.1, 121.4, 115.4, 113.0, 109.6, 109.1, 77.8, 67.0, 65.8, 60.2, 57.9, 55.8, 47.7, 29.8, 26.0, 13.7.

**2′-(Methoxycarbonyl)-2-*oxo*-1′,2′,5′,6′,7′,7*a*′-hexahydrospiro[indoline-3,3′-pyrrolizine]-1′-carboxylic acid (9a).** White solid; 89% yield; 9:1 dr; mp 225–226 °C; IR (KBr) 764, 1182, 1202, 1381, 1595, 1740, 2365, 2986, 3370 cm^−1^; HRMS (ESI) calcd for C_17_H_18_N_2_O_5_ [M + H]^+^ 331.1288, found 331.1281; ^1^H-NMR (400 MHz, DMSO-*d*_6_): *δ* 12.45 (s, 1H, CO_2_H), 10.43 (s, 1H, NH), 7.35 (d, *J* = 7.6 Hz, 1H, ArH), 7.26–7.22 (m, 1H, ArH), 6.94 (t, *J* = 15.2 Hz, 1H, ArH), 6.82 (t, *J* = 11.2 Hz, 1H, ArH), 4.05–4.00 (m, 2H, CH_2_), 3.49 (d, *J* = 8.0 Hz, 1H, CH), 3.17 (s, 3H, CO_2_CH_3_), 3.12–3.06 (m, 1H, CH), 2.28 (t, *J* = 14.0 Hz, 1H, CH), 1.94–1.87 (m, 2H, CH_2_), 1.86–1.83 (m, 1H, CH_2_), 1.71–1.66 (m, 1H, CH_2_); ^13^C-NMR (100 MHz, DMSO-*d*_6_): *δ* 179.5, 173.1, 171.6, 143.4, 129.9, 127.7, 125.6, 121.5, 110.2, 70.6, 65.9, 54.4, 51.6, 49.1, 48.1, 48.0, 28.4, 27.2.

**2′-(Methoxycarbonyl)-2-*oxo*-1-phenyl-1′,2′,5′,6′,7′,7*a*′-hexahydrospiro[indoline-3,3′-pyrrolizine]-1′-carboxylic acid (9b).** White solid; 91% yield; >99:1 dr; mp 163–165 °C; IR (KBr) 750, 1177, 1204, 1375, 1499, 1609, 1717, 1734, 2365, 2951, 3391 cm^−1^; HRMS (ESI) calcd for C_23_H_22_N_2_O_5_ [M + H]^+^ 407.1601, found 407.1615; ^1^H-NMR (400 MHz, DMSO-*d*_6_): *δ* 12.43 (s, 1H, CO_2_H), 7.60–7.55 (m, 3H, ArH), 7.49 (d, *J* = 1.2 Hz, 1H, ArH), 7.48–7.41 (m, 2H, ArH), 7.31–7.27 (m, 1H, ArH), 7.10–7.07 (m, 1H, ArH), 7.11 (d, *J* = 7.2 Hz, 1H, ArH), 4.12–3.96 (m, 2H, CH_2_), 3.77 (d, *J* = 8.0 Hz, 1H, CH), 3.41 (s, 3H, CO_2_CH_3_), 3.18–3.13 (m, 1H, CH), 2.40 (t, *J* = 14.4 Hz, 1H, CH), 1.96–1.94 (m, 2H, CH_2_), 1.93–1.92 (m, 1H, CH_2_), 1.78–1.75 (m, 1H, CH_2_); ^13^C-NMR (100 MHz, DMSO-*d*_6_): *δ* 177.3, 173.1, 171.3, 144.6, 134.5, 130.1, 130.1, 130.0, 128.7, 128.1, 127.4, 127.4, 125.0, 122.8, 109.5, 70.5, 66.1, 55.3, 51.7, 47.9, 47.7, 28.4, 27.4.

**1,2,3,11*b*-Tetrahydrospiro[benzo[*f*]pyrrolo[2,1-*a*]isoindole-5,3′-indoline]-2′,6,11-trione (10a).** Reddish-brown solid; 91% yield; 50:1 dr; mp 265–267 °C; IR (KBr) 762, 1177, 1348, 1472, 1589, 1665, 1740, 2345, 2967, 3349 cm^−1^; HRMS (ESI) calcd for C_22_H_16_N_2_O_3_ [M + H]^+^ 357.1234, found 357.1249; ^1^H-NMR (400 MHz, DMSO-*d*_6_): *δ* 10.53 (s, 1H, NH), 8.06 (d, *J* = 7.6 Hz, 1H, ArH), 7.91–7.83 (m, 3H, ArH), 7.31–7.27 (m, 1H, ArH), 7.15 (d, *J* = 7.6 Hz, 1H, ArH), 6.95–6.91 (m, 2H, ArH), 4.78 (t, *J* = 14.8 Hz, 1H, CH), 2.67–2.61 (m, 1H, CH_2_), 2.55–2.51 (m, 1H, CH_2_), 2.25–2.20 (m, 1H, CH_2_), 1.91–1.88 (m, 1H, CH_2_), 1.87–1.78 (m, 2H, CH_2_); ^13^C-NMR (100 MHz, DMSO-*d*_6_): *δ* 182.6, 181.5, 177.7, 152.5, 146.1, 143.3, 134.8, 134.7, 133.1, 132.7, 130.4, 127.1, 126.5, 126.3, 126.1, 121.7, 110.6, 77.0, 70.7, 47.8, 30.9, 27.7.

**1′-Methyl-1,2,3,11*b*-tetrahydrospiro[benzo[*f*]pyrrolo[2,1-*a*]isoindole-5,3′-indoline]-2′,6,11-trione (10b).** Orange solid; 89% yield; >99:1 dr; mp 265–266 °C; IR (KBr) 1474, 1560, 1653, 1719, 2345, 3366 cm^−1^; HRMS (ESI) calcd for C_23_H_18_N_2_O_3_ [M + H]^+^ 371.1390, found 371.1396; ^1^H-NMR (400 MHz, DMSO-*d*_6_): *δ* 8.07 (t, *J* = 8.4 Hz, 1H, ArH), 7.91 (t, *J* = 6.8 Hz, 1H, ArH), 7.89–7.80 (m, 2H, ArH), 7.42–7.38 (m, 1H, ArH), 7.21 (d, *J* = 6.8 Hz, 1H, ArH), 7.12 (d, *J* = 7.6 Hz, 1H, ArH), 7.04–7.00 (m, 1H, ArH), 4.81 (t, *J* = 14.8 Hz, 1H, CH), 3.19 (s, 3H, NCH_3_), 2.66–2.60 (m, 1H, CH_2_), 2.52–2.51 (m, 1H, CH_2_), 2.27–2.21 (m, 1H, CH_2_), 1.94–1.90 (m, 1H, CH_2_), 1.89–1.77 (m, 2H, CH_2_); ^13^C-NMR (100 MHz, DMSO-*d*_6_): *δ* 182.6, 181.4, 176.0, 152.5, 145.9, 144.6, 134.9, 134.7, 133.1, 132.6, 130.6, 126.8, 126.5, 126.3, 125.3, 122.4, 109.6, 76.6, 70.8, 47.8, 30.9, 27.8, 26.8.

**1′-Phenyl-1,2,3,11*b*-tetrahydrospiro[benzo[*f*]pyrrolo[2,1-*a*]isoindole-5,3′-indoline]-2′,6,11-trione (10c).** Reddish-brown solid; 83% yield; >99:1 dr; mp 275–276 °C; IR (KBr) 698, 756, 1375, 1458, 1670, 1734, 2345, 2877, 3266 cm^−1^; HRMS (ESI) calcd for C_28_H_20_N_2_O_3_ [M + H]^+^ 433.1547, found 433.1558; ^1^H-NMR (400 MHz, DMSO-*d*_6_): *δ* 8.07 (d, *J* = 7.2 Hz, 1H, ArH), 7.91–7.84 (m, 3H, ArH), 7.65–7.61 (m, 2H, ArH), 7.52–7.47 (m, 3H, ArH), 7.36–7.30 (m, 2H, ArH), 7.10–7.01 (m, 1H, ArH), 6.82 (d, *J* = 7.6 Hz, 1H, ArH), 4.86 (t, *J* = 15.2 Hz, 1H, CH), 2.77–2.74 (m, 1H, CH), 2.68–2.64 (m, 1H, CH_2_), 2.28–2.26 (m, 1H, CH_2_), 2.01–1.96 (m, 1H, CH_2_), 1.94–1.88 (m, 2H, CH_2_); ^13^C-NMR (100 MHz, DMSO-*d*_6_): *δ* 182.5, 181.6, 175.7, 152.4, 146.1, 144.4, 134.9, 134.8, 134.8, 133.2, 132.6, 130.7, 130.3, 130.3, 128.7, 127.4, 127.1, 126.6, 126.6, 126.3, 125.0, 123.1, 110.1, 76.9, 71.2, 47.6, 31.0, 28.1.

**5′-Fluoro-1,2,3,11*b*-tetrahydrospiro[benzo[*f*]pyrrolo[2,1-*a*]isoindole-5,3′-indoline]-2′,6,11-trione (10d).** Light-yellow solid; 92% yield; >99:1 dr; mp 277–278 °C; IR (KBr) 791, 1184, 1341, 1489, 1668, 1744, 2345, 3431 cm^−1^; HRMS (ESI) calcd for C_22_H_15_FN_2_O_3_ [M + H]^+^ 375.1139, found 375.1143; ^1^H-NMR (400 MHz, DMSO-*d*_6_): *δ* 10.57 (s, 1H, NH), 8.05 (t, *J* = 7.2 Hz, 1H, ArH), 7.91–7.84 (m, 3H, ArH), 7.16–7.11 (m, 2H, ArH), 6.93–6.90 (m, 1H, ArH), 4.78 (t, *J* = 15.2 Hz, 1H, CH), 2.67–2.61 (m, 1H, CH_2_), 2.58–2.53 (m, 1H, CH_2_), 2.24–2.19 (m, 1H, CH_2_), 1.94–1.91 (m, 1H, CH_2_), 1.90–1.86 (m, 2H, CH_2_); ^13^C-NMR (100 MHz, DMSO-*d*_6_): *δ* 182.6, 181.5, 177.7, 159.2, 156.9, 152.7, 145.5, 139.5, 134.8, 134.7, 133.2, 132.7, 127.7, 127.6, 126.4, 126.3, 116.9, 116.7, 115.2, 114.9, 111.3, 111.2, 77.1, 70.8, 47.8, 30.7, 27.8.

**5′-Bromo-1,2,3,11*b*-tetrahydrospiro[benzo[*f*]pyrrolo[2,1-*a*]isoindole-5,3′-indoline]-2′,6,11-trione (10e).** Brown solid; 87% yield; >99:1 dr; mp 274–275 °C; IR (KBr) 814, 1288, 1344, 1474, 1667, 1748, 2345, 3431 cm^−1^; HRMS (ESI) calcd for C_22_H_15_BrN_2_O_3_ [M + H]^+^ 435.0339, found 435.0339; ^1^H-NMR (400 MHz, DMSO-*d*_6_): *δ* 10.69 (s, 1H, NH), 8.07–8.05 (m, 1H, ArH), 7.91–7.88 (m, 1H, ArH), 7.87–7.84 (m, 2H, ArH), 7.49–7.43 (m, 1H, ArH), 7.43 (s, 1H, ArH), 6.90 (d, *J* = 8.8 Hz, 1H, ArH), 4.80–4.76 (m, 1H, CH), 2.65–2.54 (m, 2H, CH), 2.24–2.18 (m, 1H, CH_2_), 1.95–1.93 (m, 1H, CH_2_), 1.91–1.89 (m, 2H, CH_2_); ^13^C-NMR (100 MHz, DMSO-*d*_6_): *δ* 182.6, 181.6, 177.3, 152.8, 145.3, 142.6, 134.8, 134.7, 133.3, 133.2, 132.7, 129.9, 128.4, 126.4, 126.3, 113.6, 112.5, 76.8, 70.9, 47.9, 30.6, 27.8.

**6′-Chloro-1,2,3,11*b*-tetrahydrospiro[benzo[*f*]pyrrolo[2,1-*a*]isoindole-5,3′-indoline]-2′,6,11-trione (10f).** Orange solid; 86% yield; >99:1 dr; mp 289–190 °C; IR (KBr) 1130, 1319, 1449, 1663, 1726, 2345, 3385 cm^−1^; HRMS (ESI) calcd for C_22_H_15_ClN_2_O_3_ [M + H]^+^ 391.0844, found 391.0847; ^1^H-NMR (400 MHz, DMSO-*d*_6_): *δ* 10.73 (s, 1H, NH), 8.04 (t, *J* = 7.2 Hz, 1H, ArH), 7.90–7.86 (m, 1H, ArH), 7.84–7.82 (m, 2H, ArH), 7.19 (d, *J* = 8.0 Hz, 1H, ArH), 7.01–6.95 (m, 1H, ArH), 6.94 (s, 1H, ArH), 4.78 (t, *J* = 14.8 Hz, 1H, CH), 2.65–2.53 (m, 2H, CH_2_), 2.24–2.20 (m, 1H, CH_2_), 1.92–1.90 (m, 1H, CH_2_), 1.89–1.83 (m, 2H, CH_2_); ^13^C-NMR (100 MHz, DMSO-*d*_6_): *δ* 182.5, 181.5, 177.6, 152.7, 145.5, 144.8, 134.9, 134.7, 134.7, 133.1, 132.6, 128.7, 126.5, 126.3, 124.9, 121.4, 110.7, 76.5, 70.8, 47.8, 30.8, 27.7.

**6′-Bromo-1,2,3,11*b*-tetrahydrospiro[benzo[*f*]pyrrolo[2,1-*a*]isoindole-5,3′-indoline]-2′,6,11-trione (10g).** Bright-yellow solid; 84% yield; 50:1 dr; mp 273–274 °C; IR (KBr) 1132, 1458, 1560, 1663, 1724, 2345, 3422 cm^−1^; HRMS (ESI) calcd for C_22_H_15_BrN_2_O_3_ [M + H]^+^ 435.0339, found 435.0341; ^1^H-NMR (400 MHz, DMSO-*d*_6_): *δ* 10.70 (s, 1H, NH), 8.05 (d, *J* = 7.2 Hz, 1H, ArH), 7.89–7.83 (m, 3H, ArH), 7.13 (s, 2H, ArH), 7.08 (s, 1H, ArH), 4.78 (t, *J* = 14.8 Hz, 1H, CH), 2.66–2.53 (m, 2H, CH_2_), 2.24–2.19 (m, 1H, CH_2_), 1.91–1.77 (m, 3H, CH_2_); ^13^C-NMR (100 MHz, DMSO-*d*_6_): *δ* 182.5, 181.5, 177.5, 152.7, 145.5, 144.9, 134.8, 134.7, 133.1, 132.6, 129.0, 126.5, 126.3, 125.4, 124.3, 123.2, 113.5, 76.5, 70.8, 47.9, 30.8, 27.7.

**5′,7′-Dimethyl-1,2,3,11*b*-tetrahydrospiro[benzo[*f*]pyrrolo[2,1-*a*]isoindole-5,3′-indoline]-2′,6,11-trione (10h).** Reddish-brown solid; 88% yield; >99:1 dr; mp 276–278 °C; IR (KBr) 708, 1296, 1341, 1481, 1670, 1719, 2345, 3387, 3399 cm^−1^; HRMS (ESI) calcd for C_24_H_20_N_2_O_3_ [M + H]^+^ 385.1547, found 385.1556; ^1^H-NMR (400 MHz, DMSO-*d*_6_): *δ* 10.46 (s, 1H, NH), 8.04 (d, *J* = 7.2 Hz, 1H, ArH), 7.90–7.83 (m, 3H, ArH), 6.91 (s, 1H, ArH), 6.78 (s, 1H, ArH), 4.77 (t, *J* = 14.8 Hz, 1H, CH), 2.69–2.63 (m, 1H, CH_2_), 2.53–2.48 (m, 1H, CH_2_), 2.24 (s, 3H, CH_3_), 2.16 (s, 3H, CH_3_), 1.91–1.84 (m, 2H, CH_2_), 1.83–1.76 (m, 2H, CH_2_); ^13^C-NMR (100 MHz, DMSO-*d*_6_): *δ* 182.7, 181.5, 178.1, 152.3, 146.3, 139.4, 134.8, 134.7, 133.1, 132.7, 132.2, 130.4, 126.4, 126.3, 125.8, 124.9, 119.5, 77.3, 70.7, 47.7, 31.0, 27.8, 21.0, 16.8.

### 3.3. General Procedure for the Synthesis of Compound ***17***

The reaction was performed by a mixture of acenaphthenequinone (**16**, 0.5 mmol), *L*-proline (**6a**, 0.6 mmol), and maleimides (**11**, 0.6 mmol) or methylene indolinone (**12**) in EtOH (3 mL) at room temperature for 4 h. The resulting precipitate was collected by filtration and washed with cold EtOH 2–3 times. The crude product was purified by recrystallization (in EtOH or MeOH) or column chromatography (the mixtures of petroleum ether and ethyl acetate were used as eluents) to afford the pure corresponding compounds (**17**).

**3*a*′,6′,7′,8′,8*a*′,8*b*′-Hexahydro-1′*H*,2*H*-spiro[acenaphthylene-1,4′-pyrrolo[3,4-*a*]pyrrolizine]-1′,2,3′(2′*H*)-trione (17a).** Reddish-brown solid; 87% yield; >99:1 dr; mp 178–179 °C; IR (KBr) 768, 918, 1177, 1325, 1595, 1695, 1732, 2737, 2949, 3462 cm^−1^; HRMS (ESI) calcd for C_20_H_16_N_2_O_3_ [M + H]^+^ 333.1234, found 333.1240; ^1^H-NMR (400 MHz, DMSO-*d*_6_): *δ* 11.40 (s, 1H, NH), 8.32 (d, *J* = 8.0 Hz, 1H, ArH), 8.06 (d, *J* = 8.8 Hz, 1H, ArH), 7.95 (t, *J* = 6.8 Hz, 1H, ArH), 7.87–7.83 (m, 1H, ArH), 7.75–7.71 (m, 1H, ArH), 7.46 (d, *J* = 6.8 Hz, 1H, ArH), 4.26–4.20 (m, 1H, CH), 3.68–3.62 (m, 2H, CH_2_), 2.56–2.53 (m, 1H, CH), 2.31–2.26 (m, 1H, CH), 2.11–1.70 (m, 4H, CH_2_); ^13^C-NMR (400 MHz, DMSO-*d*_6_): *δ* 203.7, 179.2, 177.8, 142.1, 134.9, 132.7, 130.6, 130.2, 128.9, 128.7, 126.0, 124.5, 122.4, 73.3, 65.2, 56.0, 48.7, 46.9, 25.9, 24.1.

**2′-Ethyl-3*a*′,6′,7′,8′,8*a*′,8*b*′-hexahydro-1′*H*,2*H*-spiro[acenaphthylene-1,4′-pyrrolo[3,4-*a*]pyrrolizine]-1′,2,3′(2′*H*)-trione (17b).** Yellow solid; 93% yield; >99:1 dr; mp 248–249 °C; IR (KBr) 792, 1215, 1354, 1686, 1724, 2361, 2978 cm^−1^; HRMS (ESI) calcd for C_22_H_20_N_2_O_3_ [M + H]^+^ 361.1547, found 361.1551; ^1^H-NMR (400 MHz, DMSO-*d*_6_): *δ* 8.34 (d, *J* = 8.0 Hz, 1H, ArH), 8.07 (d, *J* = 8.8 Hz, 1H, ArH), 7.98 (d, *J* = 6.8 Hz, 1H, ArH), 7.86 (t, *J* = 15.2 Hz, 1H, ArH), 7.73 (t, *J* = 15.6 Hz, 1H, ArH), 7.38 (d, *J* = 7.2 Hz, 1H, ArH), 4.31–4.26 (m, 1H, CH), 3.72 (d, *J* = 8.0 Hz, 1H, CH), 3.65 (t, *J* = 16.4 Hz, 1H, CH), 3.63–3.45 (m, 2H, CH_2_), 2.50–2.46 (m, 1H, CH_2_), 2.35–2.30 (m, 1H, CH_2_), 2.12–2.06 (m, 1H, CH_2_), 1.98–1.90 (m, 1H, CH_2_), 1.80–1.69 (m, 2H, CH_2_), 1.15 (t, *J* = 14.4 Hz, 3H, CH_3_); ^13^C-NMR (400 MHz, DMSO-*d*_6_): *δ* 203.2, 177.4, 176.1, 142.1, 134.6, 132.7, 130.6, 130.2, 129.0, 128.7, 126.1, 124.9, 122.5, 73.8, 65.2, 54.3, 47.7, 47.5, 33.7, 25.6, 24.2, 13.0.

**2′-Phenyl-3*a*′,6′,7′,8′,8*a*′,8*b*′-hexahydro-1′*H*,2*H*-spiro[acenaphthylene-1,4′-pyrrolo[3,4-*a*]pyrrolizine]-1′,2,3′(2′*H*)-trione (17c).** Yellow solid; 89% yield; >99:1 dr; mp 201–203 °C; IR (KBr) 1188, 1389, 1700, 1717, 2367, 2957 cm^−1^; HRMS (ESI) calcd for C_26_H_20_N_2_O_3_ [M + H]^+^ 409.1547, found 409.1540; ^1^H-NMR (400 MHz, DMSO-*d*_6_): *δ* 8.34 (d, *J* = 8.0 Hz, 1H, ArH), 8.07 (d, *J* = 8.0 Hz, 1H, ArH), 8.01 (d, *J* = 6.8 Hz, 1H, ArH), 7.87 (t, *J* = 8.0 Hz, 1H, ArH), 7.74 (t, *J* = 8.4 Hz, 1H, ArH), 7.71–7.54 (m, 2H, ArH), 7.51–7.46 (m, 2H, ArH), 7.36 (t, *J* = 8.8 Hz, 2H, ArH), 4.42–4.37 (m, 1H, CH), 3.90 (d, *J* = 8.0 Hz, 1H, CH), 3.86 (t, *J* = 16.0 Hz, 1H, CH), 2.67–2.51 (m, 1H, CH_2_), 2.47–2.41 (m, 1H, CH_2_), 2.18–2.15 (m, 1H, CH_2_), 2.01–1.98 (m, 1H, CH_2_), 1.88–1.86 (m, 1H, CH_2_), 1.79–1.74 (m, 1H, CH_2_); ^13^C-NMR (400 MHz, DMSO-*d*_6_): *δ* 203.1, 176.8, 175.4, 142.2, 134.5, 132.8, 132.8, 130.7, 130.2, 129.6, 129.6, 129.0, 129.0, 128.8, 127.4, 127.4, 126.2, 125.0, 122.7, 74.4, 65.5, 54.6, 48.2, 48.1, 25.6, 24.5.

**2′-Benzyl-3*a*′,6′,7′,8′,8*a*′,8*b*′-hexahydro-1′*H*,2*H*-spiro[acenaphthylene-1,4′-pyrrolo[3,4-*a*]pyrrolizine]-1′,2,3′(2′*H*)-trione (17d).** Yellow solid; 90% yield; >99:1 dr; mp 207–209 °C; IR (KBr) 702, 779, 1165, 1346, 1395, 1694, 1701, 1712, 2828, 2933, 2951 cm^−1^; HRMS (ESI) calcd for C_27_H_22_N_2_O_3_ [M + H]^+^ 423.1703, found 423.1713; ^1^H-NMR (400 MHz, DMSO-*d*_6_): *δ* 8.30 (d, *J* = 8.0 Hz, 1H, ArH), 8.01 (d, *J* = 8.0 Hz, 1H, ArH), 7.95 (t, *J* = 6.8 Hz, 1H, ArH), 7.84 (t, *J* = 8.0 Hz, 1H, ArH), 7.50 (t, *J* = 8.8 Hz, 1H, ArH), 7.48–7.36 (m, 5H, ArH), 6.82 (d, *J* = 7.2 Hz, 1H, ArH), 4.69 (d, *J* = 14.4 Hz, 1H, CH_2_), 4.58 (d, *J* = 14.4 Hz, 1H, CH_2_), 4.31–4.26 (m, 1H, CH), 3.76 (d, *J* = 8.0 Hz, 1H, CH), 3.72 (t, *J* = 16.0 Hz, 1H, CH), 2.20–2.13 (m, 2H, CH_2_), 2.11–2.08 (m, 1H, CH_2_), 1.93–1.88 (m, 1H, CH_2_), 1.65–1.59 (m, 2H, CH_2_); ^13^C-NMR (400 MHz, DMSO-*d*_6_): *δ* 203.0, 177.3, 175.9, 142.1, 136.1, 134.5, 132.7, 130.6, 130.2, 129.1, 129.1, 129.0, 129.0, 129.0, 128.5, 128.4, 126.0, 124.9, 122.6, 74.0, 65.2, 54.2, 47.9, 47.6, 42.4, 25.2, 23.9.

**Ethyl 5″-methoxy-2,2″-dioxo-5′,6′,7′,7a′-tetrahydro-1′*H*,2*H*-dispiro[acenaphthylene-1,3′-pyrrolizine-2′,3″-indoline]-1′-carboxylate (17e).** Yellow solid; 85% yield; 50:1 dr; mp 277–279 °C; IR (KBr) 743, 1212, 1347, 1434, 1700, 1756, 2825, 2998 cm^−1^; HRMS (ESI) calcd for C_29_H_26_N_2_O_5_ [M + H]^+^ 483.1914, found 483.1921; ^1^H-NMR (400 MHz, DMSO-*d*_6_): *δ* 9.91 (s, 1H, NH), 8.16 (d, *J* = 8.0 Hz, 1H, ArH), 7.84–7.75 (m, 3H, ArH), 7.34 (t, *J* = 15.2 Hz, 2H, ArH), 6.84–6.76 (m, 2H, ArH), 6.49 (d, *J* = 8.4 Hz, 1H, ArH), 4.48–4.42 (m, 1H, CH), 4.22 (d, *J* = 8.8 Hz, 1H, CH), 3.81 (s, 3H, OCH_3_), 3.69–3.63 (m, 2H, CH_2_), 2.59–2.55 (m, 2H, CH_2_), 2.38–2.23 (m, 2H, CH_2_), 2.14–2.10 (m, 2H, CH_2_), 0.69 (t, *J* = 14.0 Hz, 3H, CH_3_); ^13^C-NMR (400 MHz, DMSO-*d*_6_): *δ* 207.2, 173.6, 169.8, 154.8, 141.0, 136.4, 135.9, 132.1, 131.8, 130.3, 129.3, 128.7, 128.5, 125.7, 122.2, 120.4, 114.0, 113.6, 109.9, 79.5, 68.2, 66.2, 60.4, 56.2, 52.2, 46.5, 31.4, 30.4, 13.8.

## 4. Conclusions

In conclusion, we developed a chemically sustainable and dipolarophile-controlled three-component 1,3-dipolar cycloaddition reaction to construct a broad range of (40 examples) functionalized *N*-fused pyrrolidinyl spirooxindoles in 76–95% yields with excellent diastereoselectivities (up to >99:1 dr). The scaffolds of these products can be well-controlled by employing different 1,4-enedione derivatives as dipolarophiles in EtOH at room temperature. This reaction can be scaled-up to the gram level, and the products can be purified by filtering and recrystallization without compromising the chemical outcome, inferring an efficient synthetic method for achieving diverse, complex natural spirooxindole alkaloids and biologically active spirooxindole derivatives. This reaction not only realizes a concise dipolarophile-controlled, catalysis-free 1,3-dipolar cycloaddition under green conditions but also provides a practical strategy for the construction of functionalized *N*-fused pyrrolidinyl spirooxindoles. Further investigations of the synthesized *N*-fused pyrrolidinyl spirooxindoles are ongoing in our laboratory.

## Figures and Tables

**Table 1:** Optimization of reaction conditions for the model reaction *^a^*.

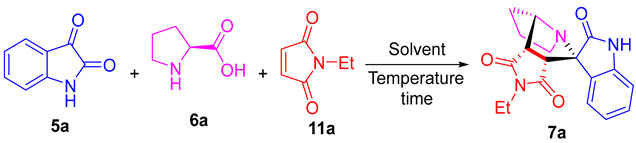				
Entry	Solvent	Temperature (°C)	Time (h)	Yield *^b^* (%)
1	Toluene	r.t. *^c^*	4	72
2	Benzene	r.t.	4	57
3	Acetonitrile	r.t.	4	49
4	Ether	r.t.	4	32
5	DCE	r.t.	4	67
6	DCM	r.t.	4	61
7	CHCl_3_	r.t.	4	58
8	THF	r.t.	4	45
9	Acetone	r.t.	4	24
10	1,4-Dioxane	r.t.	4	64
11	DMSO	r.t.	4	38
12	DMF	r.t.	4	25
13	H_2_O	r.t.	4	18
14	*^i^*PrOH	r.t.	4	81
15	MeOH	r.t.	4	94
16	EtOH	r.t.	4	93
17	EtOH	40	4	93
18	EtOH	reflux	4	94
19	EtOH	r.t.	2	78
20	EtOH	r.t.	8	93
21	EtOH	r.t.	24	94

*^a^* All reactions were carried out with isatin **5a** (0.5 mmol), *L*-proline (**6a**, 0.6 mmol), and *N*-ethylmaleimide (**11a**, 0.6 mmol) in corresponding solvents (3 mL) at corresponding temperatures. *^b^* Isolated or recrystallized yields based on isatin (**5a)**; >20:1 dr by ^1^H NMR analysis. *^c^* Room temperature.

## Data Availability

Data are contained within the article.

## Supplementary Materials

The following supporting information can be downloaded at: https://www.mdpi.com/article/10.3390/ijms24043771/s1.
