# Supplementary material for: Dipolarophile-Controlled Regioselective 1,3-Dipolar Cycloaddition: A Switchable Divergent Access to Functionalized N-Fused Pyrrolidinyl Spirooxindoles
Source: Int J Mol Sci. 2023 Feb 13;24(4):3771. doi: 10.3390/ijms24043771 (PMC9966135; doi:10.3390/ijms24043771)

# *Supplementary Materials*

# Dipolarophile-Controlled Regioselective 1,3-Dipolar Cycloaddition：A Switchable Divergent Access to Functionalized *N*-Fused-Pyrrolidinyl Spirooxindoles

Yongchao Wang [1,*], Lijun Yan [1], Yuxin Yan [1], Sujin Li [1], Hongying Lu [1], Jia Liu [1], and Jianwei Dong [2,*]

[1] Colleage of Vocational and Technical Education, Yunnan Normal University, Kunming 650092, China.

[2] College of Chemistry and Environmental Science, Qujing Normal University, Qujing, 655011, China

[*] Correspondence:

Yongchao Wang (ycwang@ynnu.edu.cn; yongchaowang126@126.com)

Jianwei Dong (jwdongyn@mail.qjnu.edu.cn)

# *Table of contents*

**1. $^{1}H$-NMR, $^{13}C$-NMR and HRMS spectra for compounds 7 ……………………S3**

**2. $^{1}H$-NMR, $^{13}C$-NMR and HRMS spectra for compounds 8 …………………S53**

**3. $^{1}H$-NMR, $^{13}C$-NMR and HRMS spectra for compounds 9 …………………S63**

**4. $^{1}H$-NMR, $^{13}C$-NMR and HRMS spectra for compounds 10 …………………S67**

**5. $^{1}H$-NMR, $^{13}C$-NMR and HRMS spectra for compounds 17 …………………S83**

**6. Single crystal X-ray diffraction study data of compound 7a ………………S93**

## 1. $^1$H and $^{13}$C NMR spectra for compounds 7

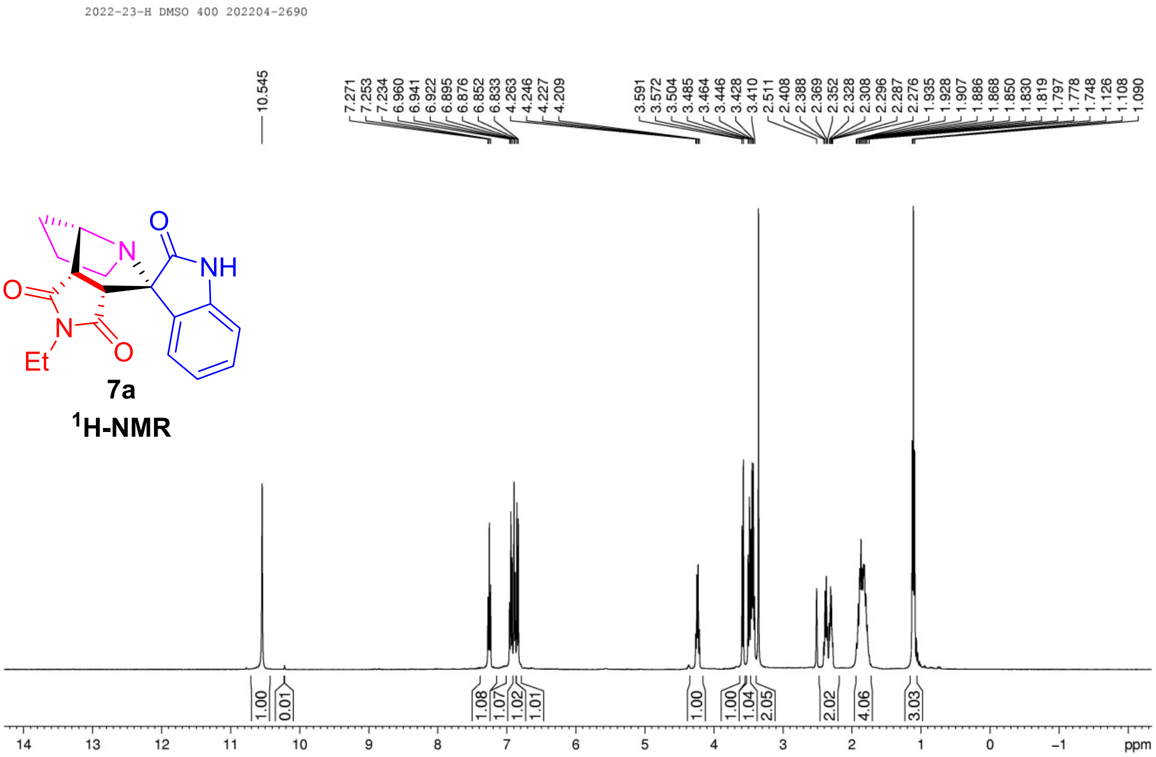


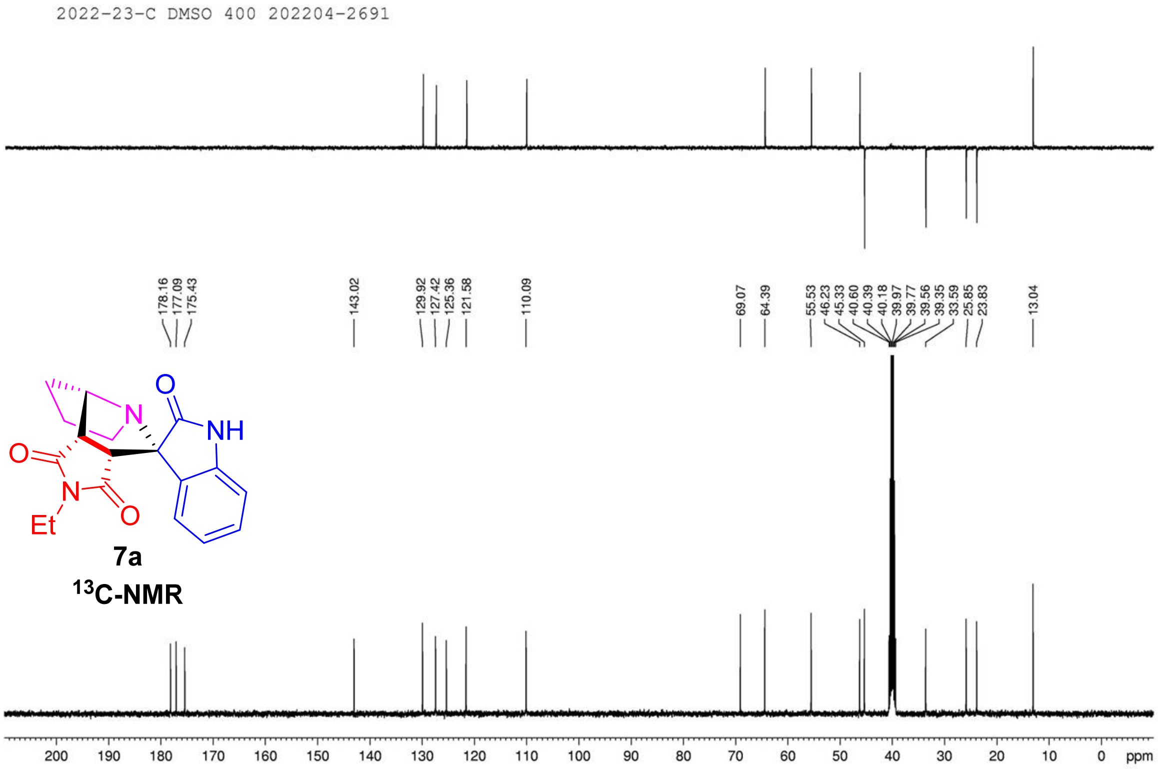

## Qualitative Compound Report

| | | | |
|---|---|---|---|
| Data File | 8036980-22-23.d | Sample Name | 8036980-22-23 |
| Sample Type | Sample | Position | P1-D8 |
| Instrument Name | Instrument 1 | User Name | |
| Acq Method | pos-1min.m | Acquired Time | 8/8/2022 5:45:42 PM |
| IRM Calibration Status | Success | DA Method | default.m |
| Comment | | | |
| Sample Group | | Info. | |
| Stream Name | LC 1 | Acquisition SW Version | 6200 series TOF/6500 series Q-TOF B.08.00 (B8058.0) |

**Compound Table**

| Compound Label | RT | Mass | Abund | Formula | Tgt Mass | Diff (ppm) |
|---|---|---|---|---|---|---|
| Cpd 1: C18 H19 N3 O3 | 0.087 | 325.1436 | 91791 | C18 H19 N3 O3 | 325.1426 | 2.87 |

MS Zoomed Spectrum

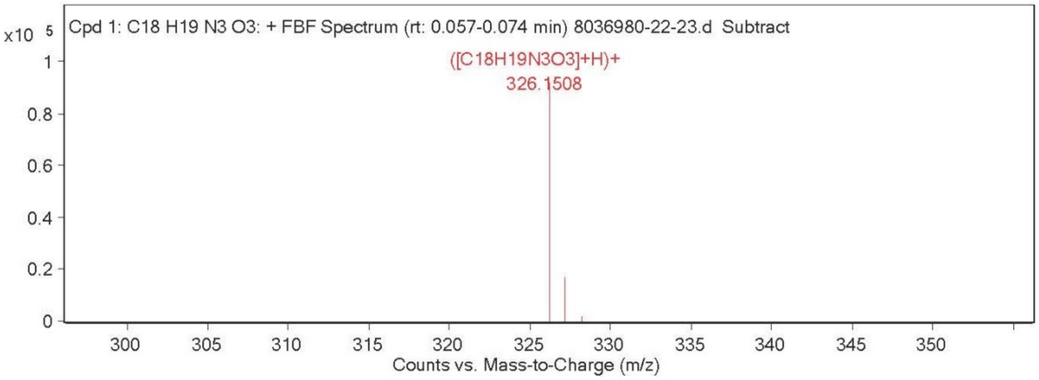


--- End Of Report ---

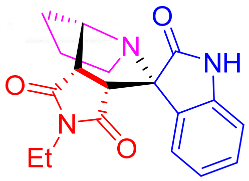


**7a-MS**

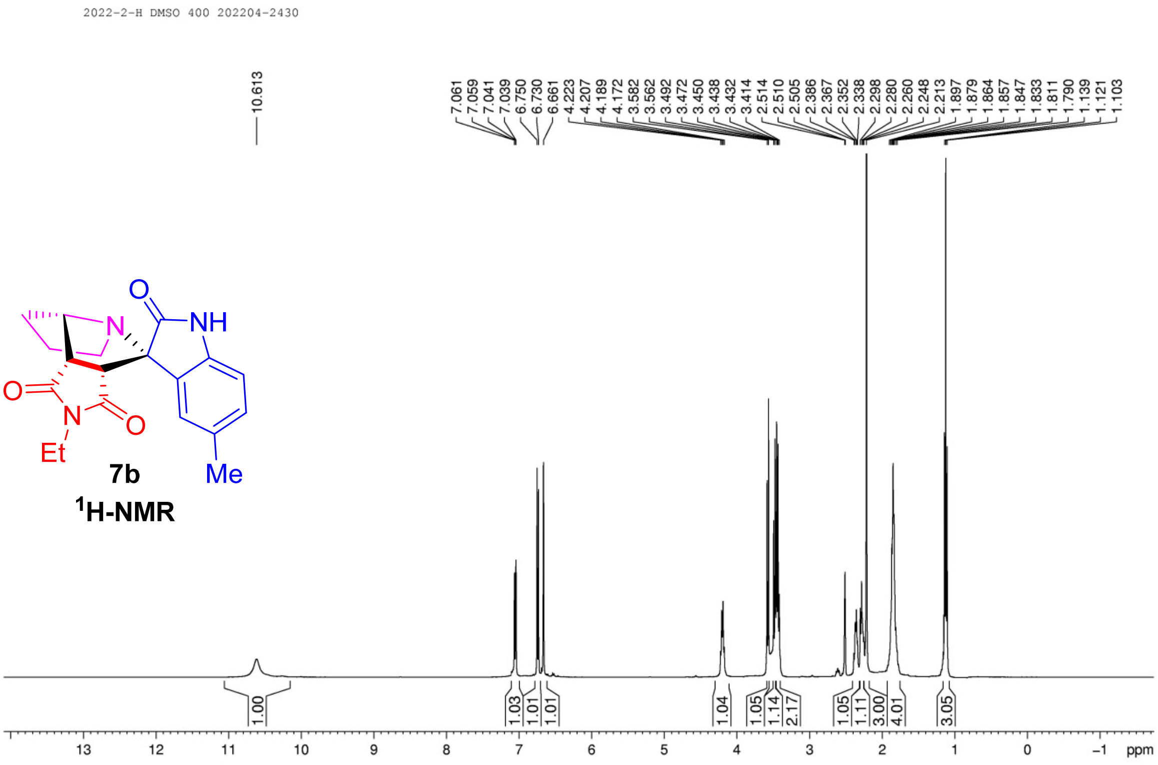
2022-2-H DMSO 400 202204-2430
10.613
7.061
7.059
7.041
7.039
6.750
6.730
6.661
4.223
4.207
4.189
4.172
3.582
3.562
3.492
3.472
3.450
3.438
3.432
3.414
2.514
2.510
2.505
2.386
2.367
2.352
2.338
2.298
2.280
2.260
2.248
2.213
1.897
1.879
1.864
1.857
1.847
1.833
1.811
1.790
1.139
1.121
1.103
O
NH
N
O
N
O
Et
Me
7b
1H-NMR
1.00
1.03
1.01
1.01
1.04
1.05
1.14
2.17
1.05
1.11
3.00
4.01
3.05
13
12
11
10
9
8
7
6
5
4
3
2
1
0
–1
ppm

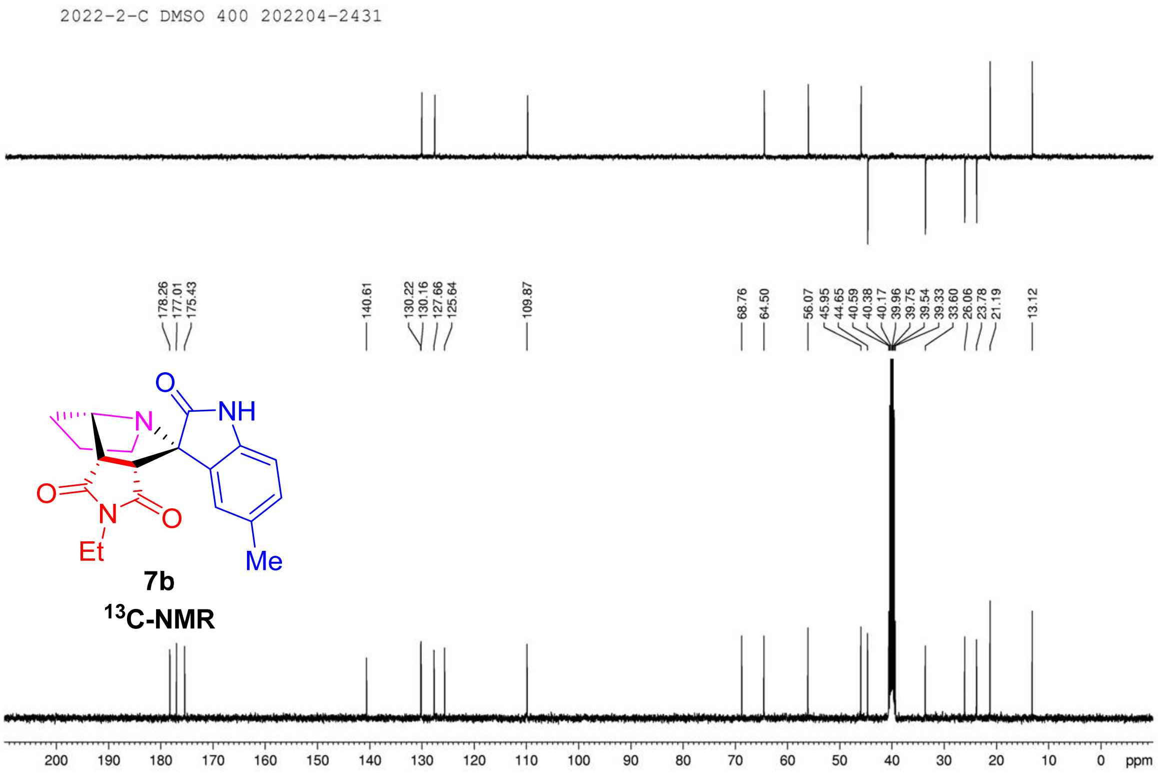
2022-2-C DMSO 400 202204-2431
178.26
177.01
175.43
140.61
130.22
130.16
127.66
125.64
109.87
68.76
64.50
56.07
45.95
44.65
40.59
40.38
40.17
39.96
39.75
39.54
39.33
33.60
26.06
23.78
21.19
13.12
O
NH
N
O
N
O
Et
Me
7b
13C-NMR
200
190
180
170
160
150
140
130
120
110
100
90
80
70
60
50
40
30
20
10
0
ppm

## Qualitative Compound Report

| | | | |
|---|---|---|---|
| **Data File** | 2033368-22-2.d | **Sample Name** | 2033368-22-2 |
| **Sample Type** | Sample | **Position** | P1-A3 |
| **Instrument Name** | Instrument 1 | **User Name** | |
| **Acq Method** | pos-1min.m | **Acquired Time** | 2/6/2023 5:06:50 PM |
| **IRM Calibration Status** | Success | **DA Method** | QG-907.m |
| **Comment** | | | |
| **Sample Group** | | **Info.** | |
| **Stream Name** | LC 1 | **Acquisition SW Version** | 6200 series TOF/6500 series Q-TOF B.08.00 (B8058.0) |

**Compound Table**

| Compound Label | RT | Mass | Abund | Formula | Tgt Mass | Diff (ppm) |
|---|---|---|---|---|---|---|
| Cpd 1: C19 H21 N3 O3 | 0.131 | 339.159 | 469321 | C19 H21 N3 O3 | 339.1583 | 2.14 |

MS Zoomed Spectrum

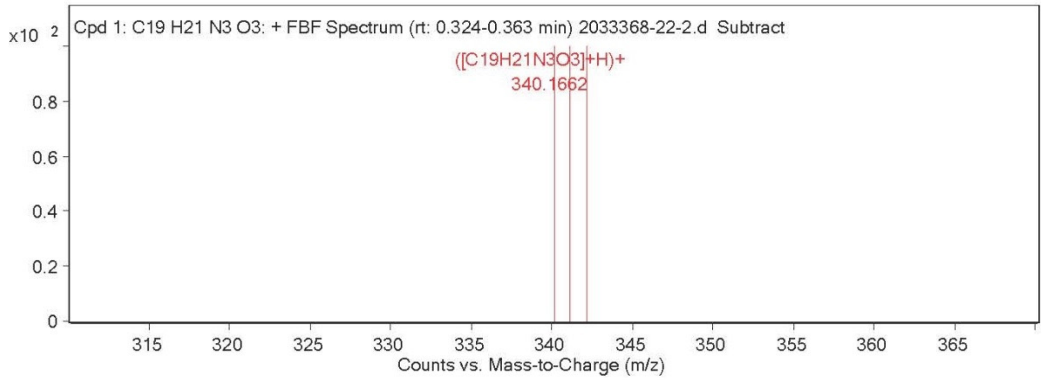


--- End Of Report ---

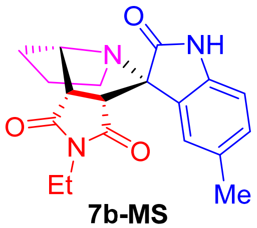


**7b-MS**

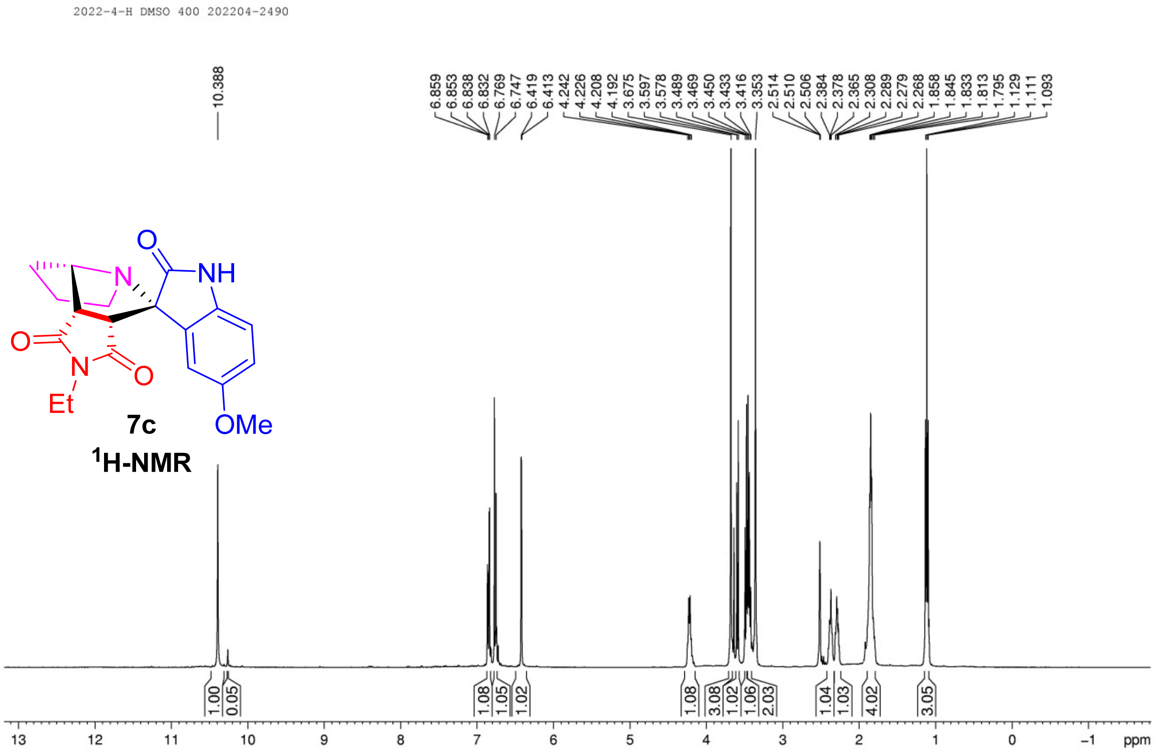
2022-4-H DMSO 400 202204-2490
10.388
6.859 6.853 6.838 6.832 6.769 6.747 6.419 6.413 4.242 4.226 4.208 4.192 3.675 3.597 3.578 3.489 3.469 3.450 3.433 3.416 3.353 2.514 2.510 2.506 2.384 2.378 2.365 2.308 2.289 2.279 2.268 1.858 1.845 1.833 1.813 1.795 1.129 1.111 1.093
O NH N O N O Et OMe
7c
1H-NMR
1.00 0.05 1.08 1.05 1.02 1.08 3.08 1.02 1.06 2.03 1.04 1.03 4.02 3.05
ppm

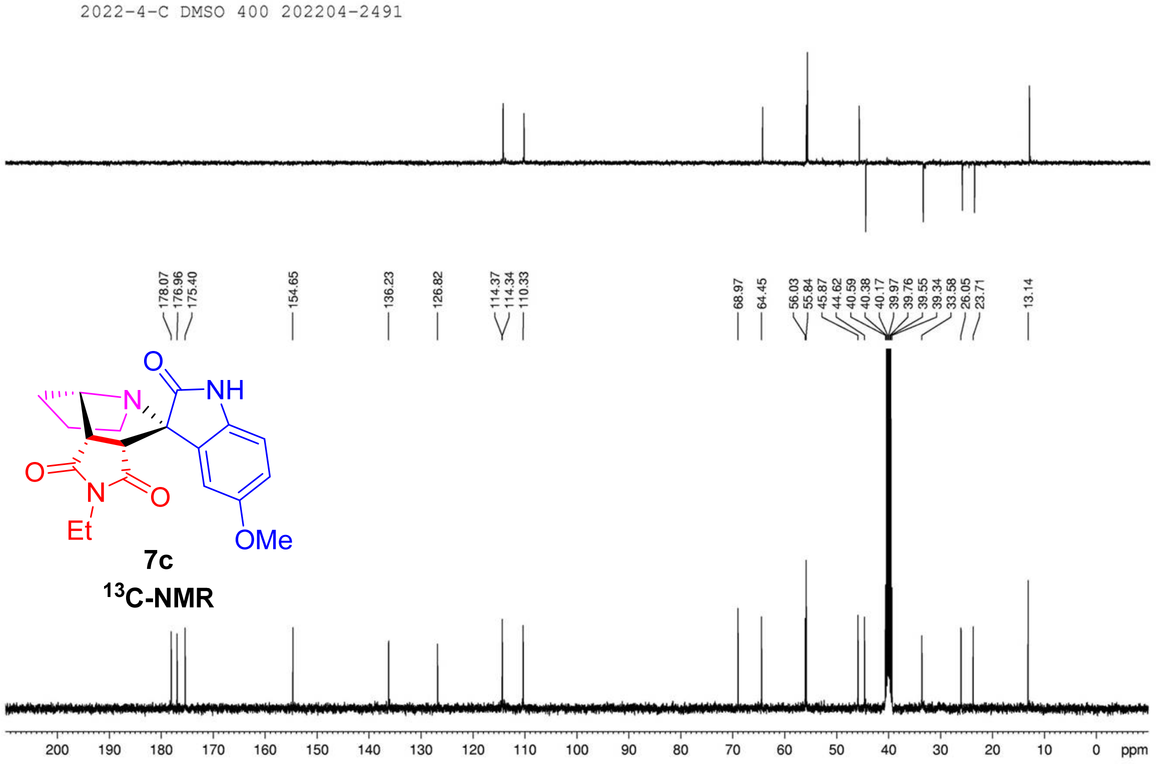
2022-4-C DMSO 400 202204-2491
178.07 176.96 175.40 154.65 136.23 126.82 114.37 114.34 110.33 68.97 64.45 56.03 55.84 45.87 44.62 40.59 40.38 40.17 39.97 39.76 39.55 39.34 33.58 26.05 23.71 13.14
O NH N O N O Et OMe
7c
13C-NMR
ppm

## Qualitative Compound Report

| | | | |
|---|---|---|---|
| Data File | 2033368-22-4.d | Sample Name | 2033368-22-4 |
| Sample Type | Sample | Position | P1-A4 |
| Instrument Name | Instrument 1 | User Name | |
| Acq Method | pos-1min.m | Acquired Time | 2/6/2023 5:08:41 PM |
| IRM Calibration Status | Success | DA Method | QG-907.m |
| Comment | | | |
| Sample Group | | Info. | |
| Stream Name | LC 1 | Acquisition SW Version | 6200 series TOF/6500 series Q-TOF B.08.00 (B8058.0) |

**Compound Table**

| Compound Label | RT | Mass | Abund | Formula | Tgt Mass | Diff (ppm) |
|---|---|---|---|---|---|---|
| Cpd 1: C19 H21 N3 O4 | 0.216 | 355.1533 | 6162 | C19 H21 N3 O4 | 355.1532 | 0.28 |

MS Zoomed Spectrum

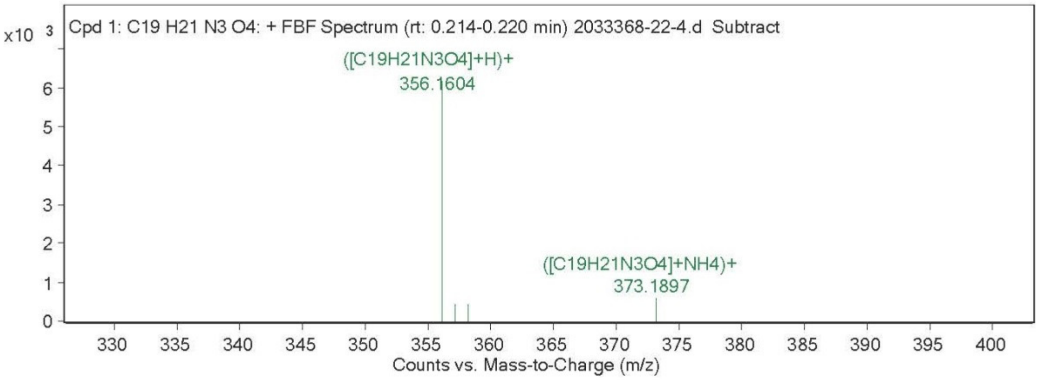


--- End Of Report ---

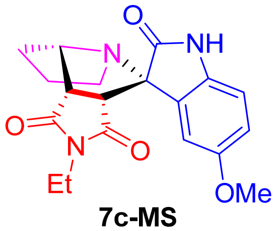


**7c-MS**

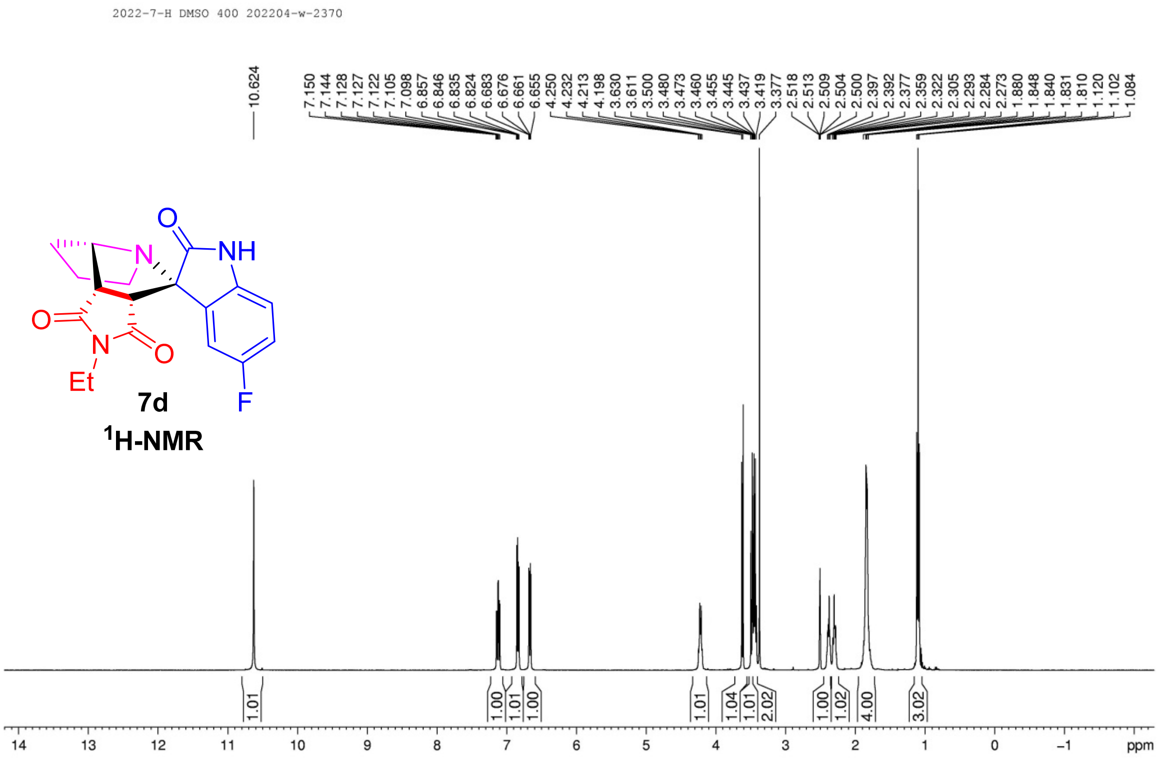
2022-7-H DMSO 400 202204-w-2370
7d
1H-NMR
ppm

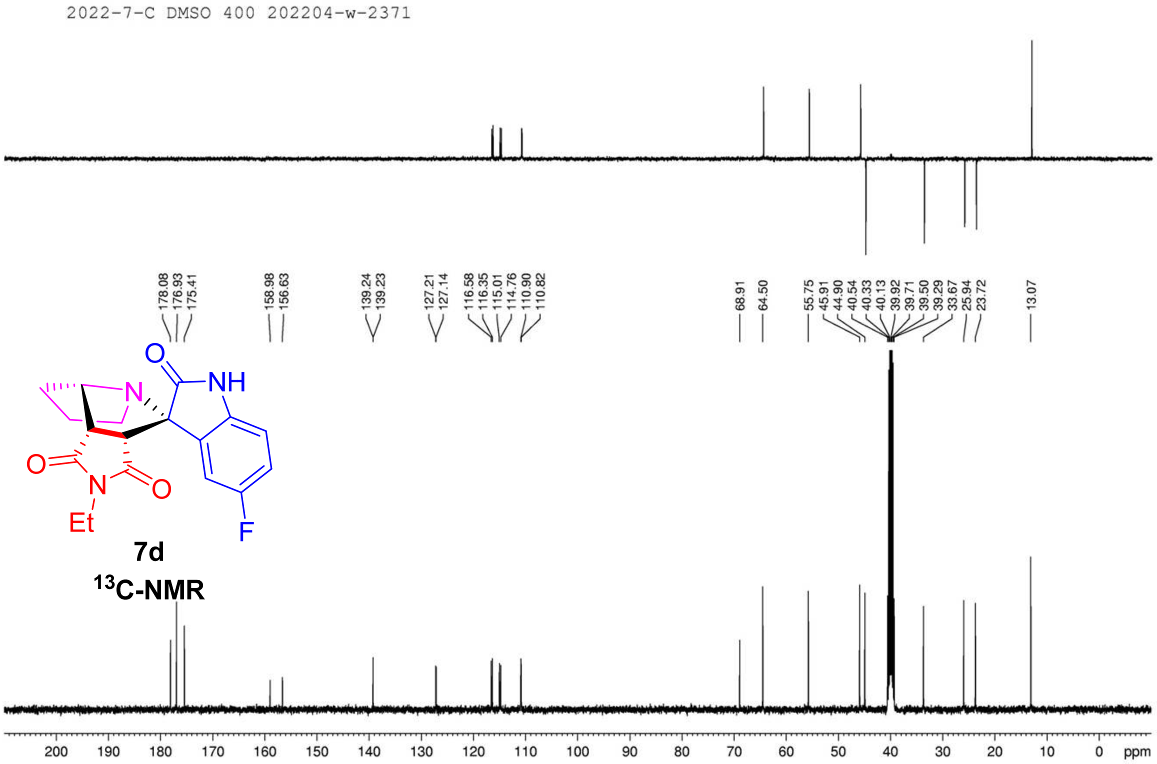
2022-7-C DMSO 400 202204-w-2371
7d
13C-NMR
ppm

## Qualitative Compound Report

| | | | |
|---|---|---|---|
| Data File | 2033368-22-2.d | Sample Name | 2033368-22-2 |
| Sample Type | Sample | Position | |
| Instrument Name | Instrument 1 | User Name | |
| Acq Method | pos-1min.m | Acquired Time | 2/6/2023 5:14:12 PM |
| IRM Calibration Status | Success | DA Method | QG-907.m |
| Comment | | | |
| Sample Group | | Info. | |
| Stream Name | LC 1 | Acquisition SW Version | 6200 series TOF/6500 series Q-TOF B.08.00 (B8058.0) |

**Compound Table**

| Compound Label | RT | Mass | Abund | Formula | Tgt Mass | Diff (ppm) |
|---|---|---|---|---|---|---|
| Cpd 1: C18 H18 F N3 O3 | 0.178 | 343.134 | 301648 | C18 H18 F N3 O3 | 343.1332 | 2.25 |

MS Zoomed Spectrum

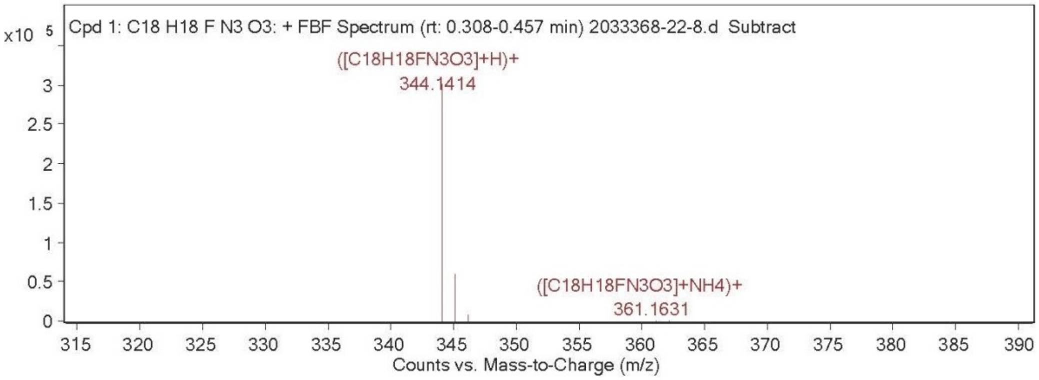


--- End Of Report ---

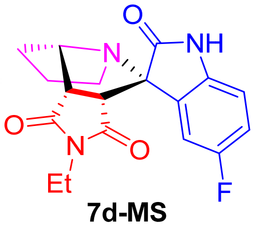


**7d-MS**

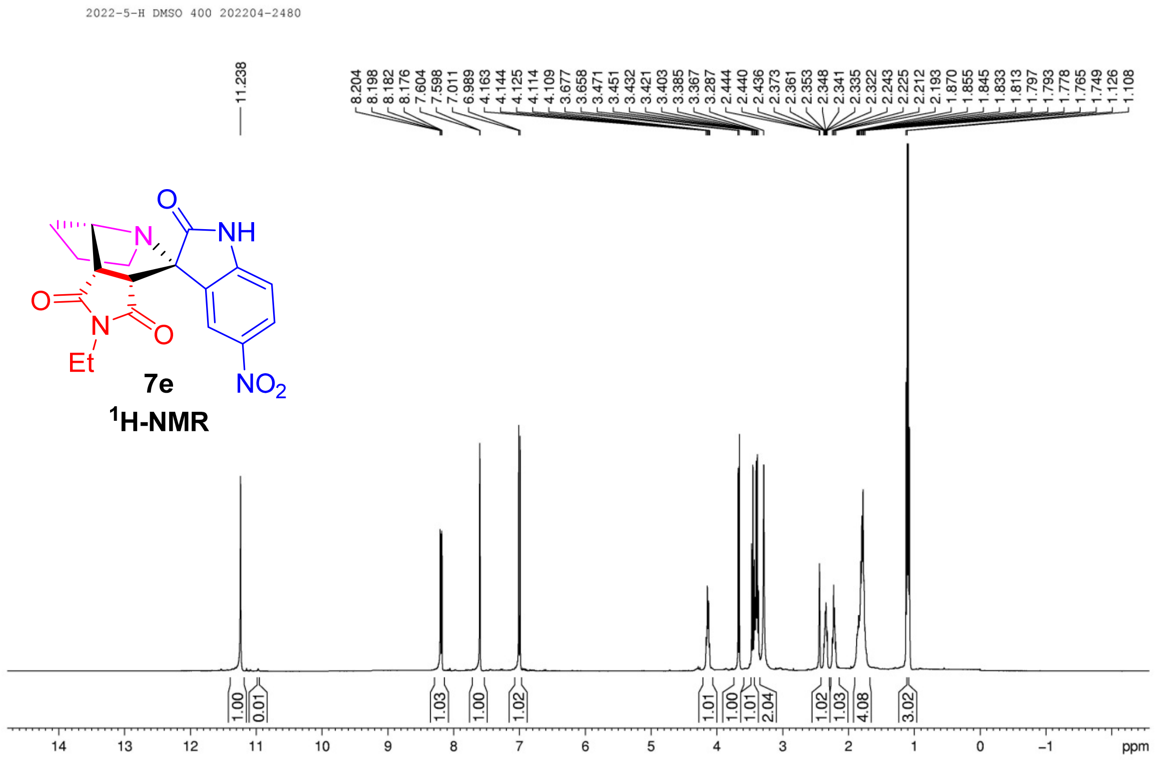
2022-5-H DMSO 400 202204-2480
11.238
8.204
8.198
8.182
8.176
7.604
7.598
7.011
6.989
4.163
4.144
4.125
4.114
4.109
3.677
3.658
3.471
3.451
3.432
3.421
3.403
3.385
3.367
3.287
2.444
2.440
2.436
2.373
2.361
2.353
2.348
2.341
2.335
2.322
2.243
2.225
2.212
2.193
1.870
1.855
1.845
1.833
1.813
1.797
1.793
1.778
1.765
1.749
1.126
1.108
O
NH
N
O
O
N
Et
$NO_2$
7e
$^1$H-NMR
1.00
0.01
1.03
1.00
1.02
1.01
1.00
1.01
2.04
1.02
1.03
4.08
3.02
14
13
12
11
10
9
8
7
6
5
4
3
2
1
0
–1
ppm

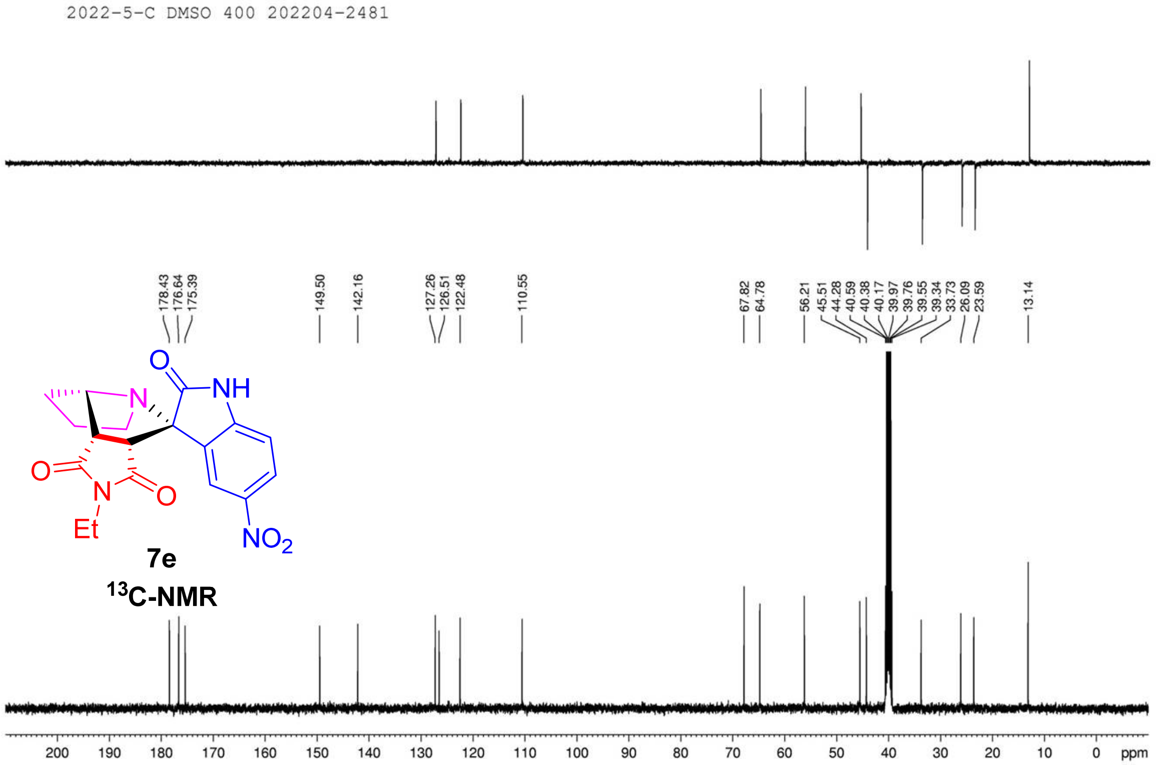
2022-5-C DMSO 400 202204-2481
178.43
176.64
175.39
149.50
142.16
127.26
126.51
122.48
110.55
67.82
64.78
56.21
45.51
44.28
40.59
40.38
40.17
39.97
39.76
39.55
39.34
33.73
26.09
23.59
13.14
O
NH
N
O
O
N
Et
$NO_2$
7e
$^{13}$C-NMR
200
190
180
170
160
150
140
130
120
110
100
90
80
70
60
50
40
30
20
10
0
ppm

## Qualitative Compound Report

| | | | |
|---|---|---|---|
| Data File | 2033368-22-21.d | Sample Name | 2033368-22-21 |
| Sample Type | Sample | Position | |
| Instrument Name | Instrument 1 | User Name | |
| Acq Method | pos-1min.m | Acquired Time | 2/6/2023 5:42:25 PM |
| IRM Calibration Status | Success | DA Method | QG-907.m |
| Comment | | | |
| Sample Group | | Info. | |
| Stream Name | LC 1 | Acquisition SW Version | 6200 series TOF/6500 series Q-TOF B.08.00 (B8058.0) |

**Compound Table**

| Compound Label | RT | Mass | Abund | Formula | Tgt Mass | Diff (ppm) |
|---|---|---|---|---|---|---|
| Cpd 1: C18 H18 N4O5 | 0.447 | 370.1327 | 213394 | C18 H18 N4O5 | 370.1317 | 2.55 |

MS Zoomed Spectrum

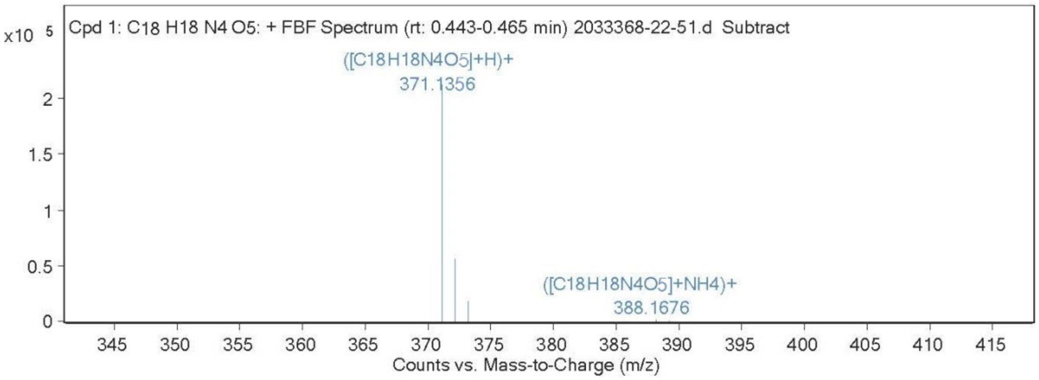


--- End Of Report ---

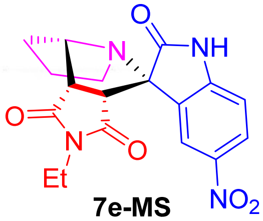


**7e-MS**

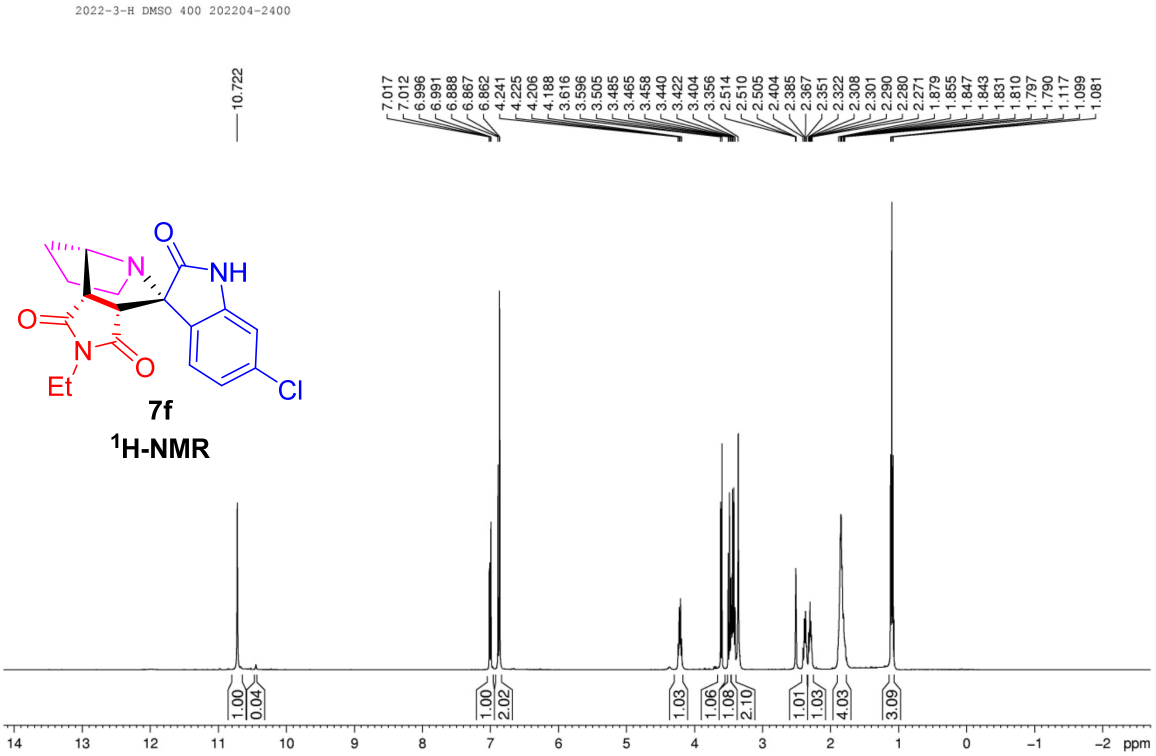
2022-3-H DMSO 400 202204-2400
10.722
7.017 7.012 6.996 6.991 6.888 6.867 6.862 4.241 4.225 4.206 4.188 3.616 3.596 3.505 3.485 3.465 3.458 3.440 3.422 3.404 3.356 2.514 2.510 2.505 2.404 2.385 2.367 2.351 2.322 2.308 2.301 2.290 2.280 2.271 1.879 1.855 1.847 1.843 1.831 1.810 1.797 1.790 1.117 1.099 1.081
O
NH
N
O
N
O
Et
Cl
7f
1H-NMR
1.00
0.04
1.00
2.02
1.03
1.06
1.08
2.10
1.01
1.03
4.03
3.09
14 13 12 11 10 9 8 7 6 5 4 3 2 1 0 −1 −2 ppm

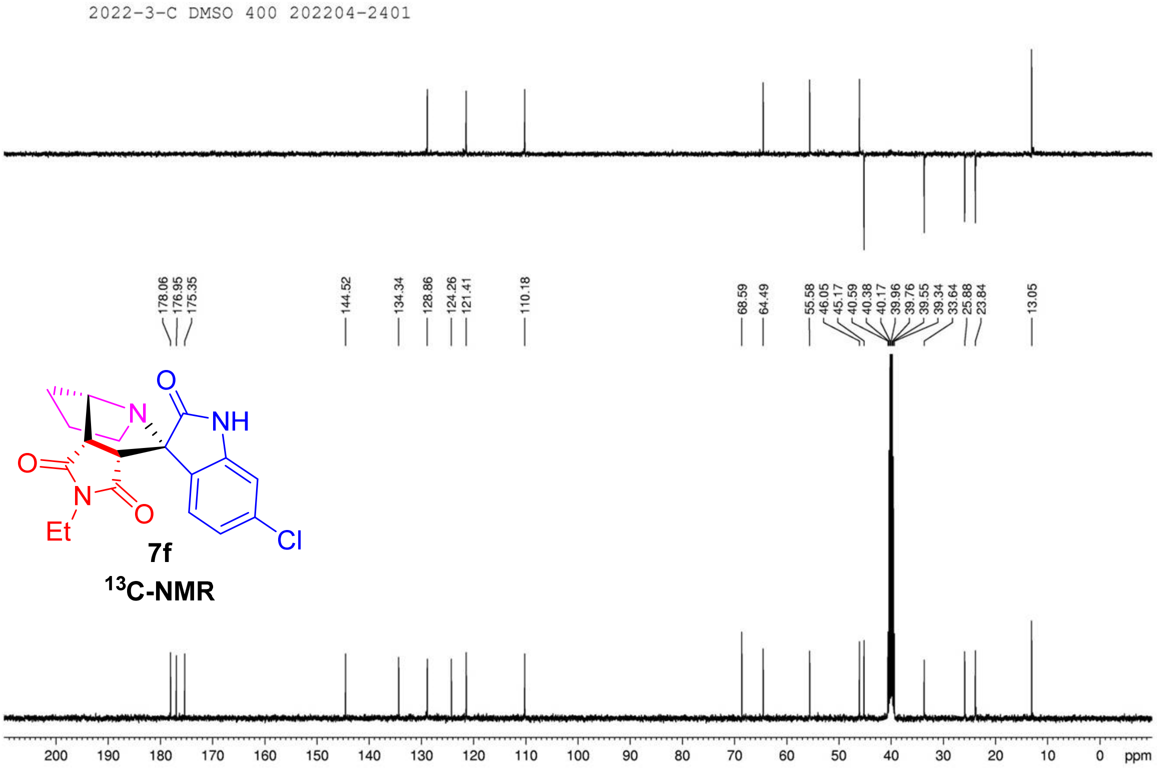
2022-3-C DMSO 400 202204-2401
178.06 176.95 175.35
144.52
134.34
128.86
124.26
121.41
110.18
68.59
64.49
55.58 46.05 45.17 40.59 40.38 40.17 39.96 39.76 39.55 39.34 33.64 25.88 23.84
13.05
O
NH
N
O
N
O
Et
Cl
7f
13C-NMR
200 190 180 170 160 150 140 130 120 110 100 90 80 70 60 50 40 30 20 10 0 ppm

## Qualitative Compound Report

| | | | |
|---|---|---|---|
| Data File | 2033368-22-44.d | Sample Name | 2033368-22-44 |
| Sample Type | Sample | Position | |
| Instrument Name | Instrument 1 | User Name | |
| Acq Method | pos-1min.m | Acquired Time | 2/6/2023 5:47:55 PM |
| IRM Calibration Status | Success | DA Method | QG-907.m |
| Comment | | | |
| Sample Group | | Info. | |
| Stream Name | LC 1 | Acquisition SW Version | 6200 series TOF/6500 series Q-TOF B.08.00 (B8058.0) |

**Compound Table**

| Compound Label | RT | Mass | Abund | Formula | Tgt Mass | Diff (ppm) |
|---|---|---|---|---|---|---|
| Cpd 1: C18 H18 Cl N3 O3 | 0.112 | 360.0779 | 85267 | C18 H18 Cl N3 O3 | 360.0771 | 2.07 |

MS Zoomed Spectrum

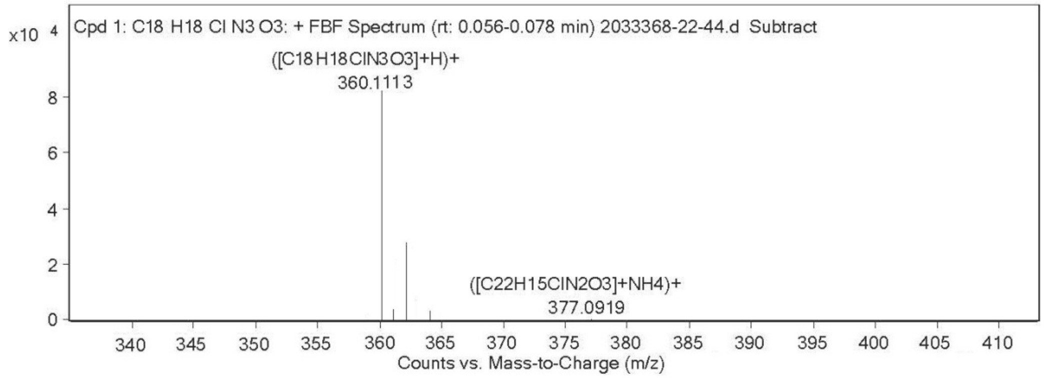


--- End Of Report ---

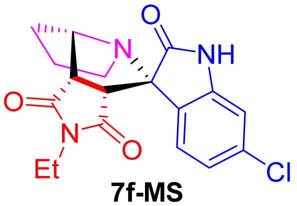

**7f-MS**

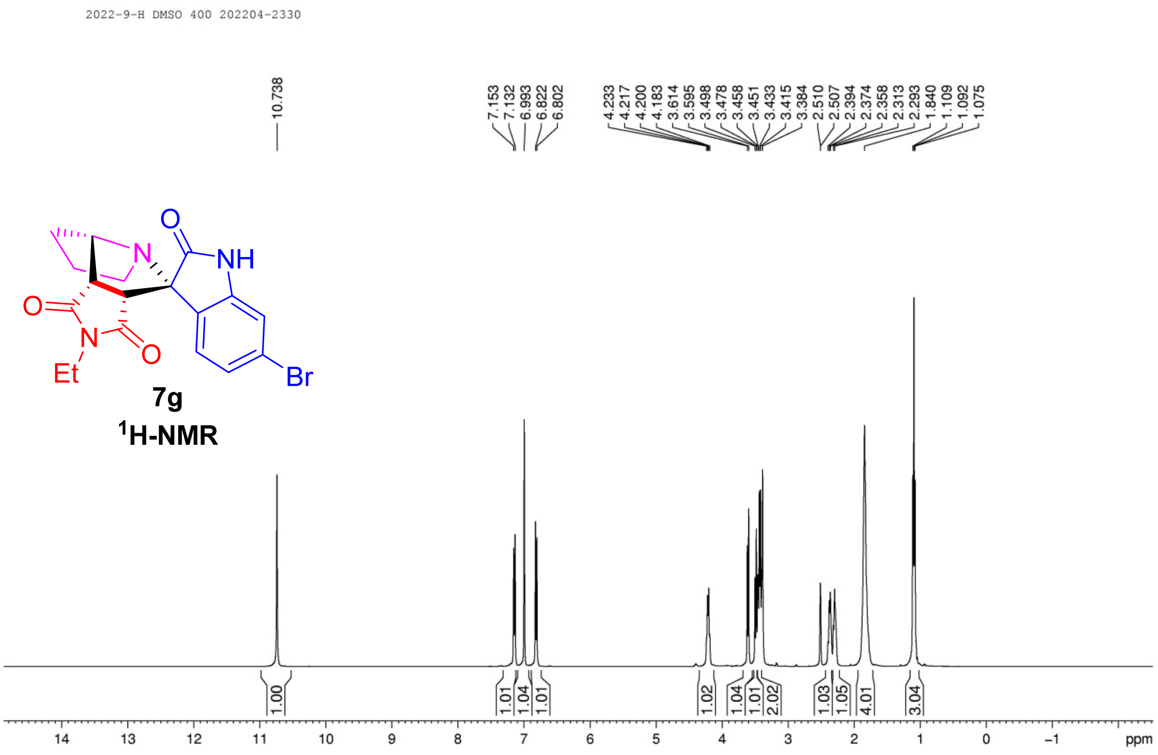
2022-9-H DMSO 400 202204-2330
10.738
7.153
7.132
6.993
6.822
6.802
4.233
4.217
4.200
4.183
3.614
3.595
3.498
3.478
3.458
3.451
3.433
3.415
3.384
2.510
2.507
2.394
2.374
2.358
2.313
2.293
1.840
1.109
1.092
1.075
O
NH
N
O
N
O
Et
Br
7g
$^1$H-NMR
1.00
1.01
1.04
1.01
1.02
1.04
1.01
2.02
1.03
1.05
4.01
3.04
14
13
12
11
10
9
8
7
6
5
4
3
2
1
0
–1
ppm

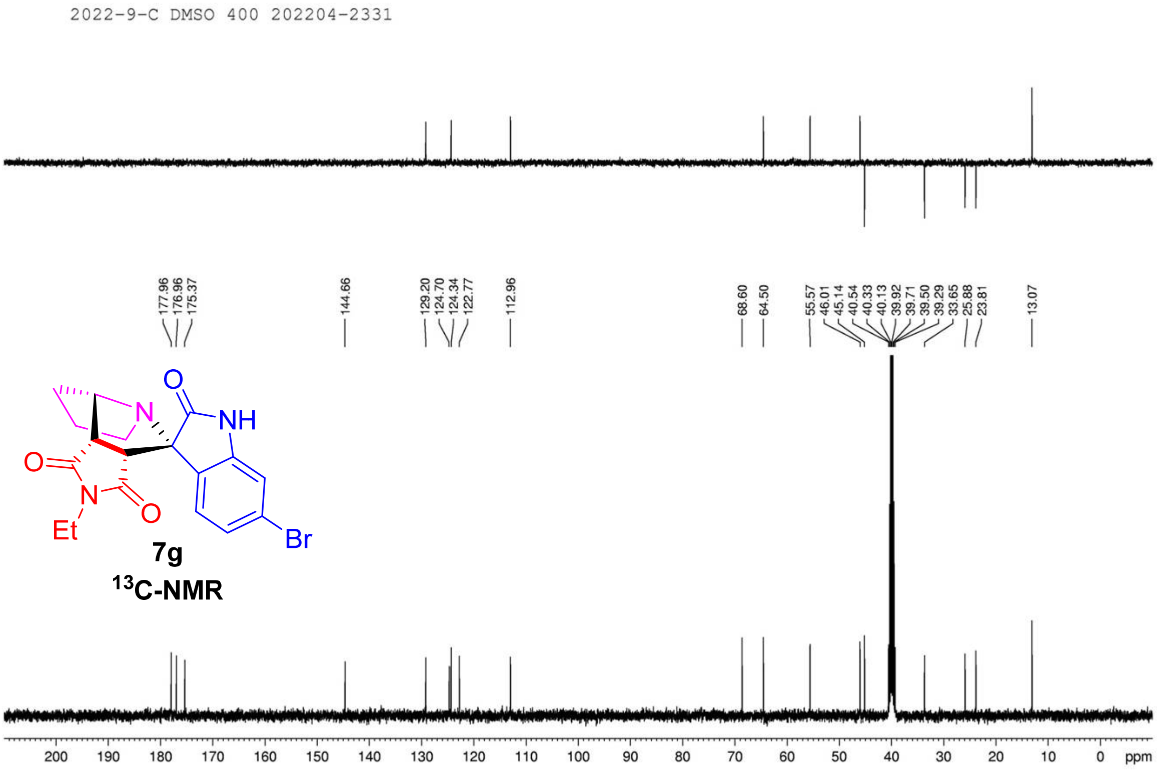
2022-9-C DMSO 400 202204-2331
177.96
176.96
175.37
144.66
129.20
124.70
124.34
122.77
112.96
68.60
64.50
55.57
46.01
45.14
40.54
40.33
40.13
39.92
39.71
39.50
39.29
33.65
25.88
23.81
13.07
O
NH
N
O
N
O
Et
Br
7g
$^{13}$C-NMR
200
190
180
170
160
150
140
130
120
110
100
90
80
70
60
50
40
30
20
10
0
ppm

## Qualitative Compound Report

| | | | |
|---|---|---|---|
| Data File | 8036980-22-9.d | Sample Name | 8036980-22-9 |
| Sample Type | Sample | Position | P1-D5 |
| Instrument Name | Instrument 1 | User Name | |
| Acq Method | pos-1min.m | Acquired Time | 8/8/2022 5:40:06 PM |
| IRM Calibration Status | Success | DA Method | default.m |
| Comment | | | |
| Sample Group | | Info. | |
| Stream Name | LC 1 | Acquisition SW Version | 6200 series TOF/6500 series Q-TOF B.08.00 (B8058.0) |

**Compound Table**

| Compound Label | RT | Mass | Abund | Formula | Tgt Mass | Diff (ppm) |
|---|---|---|---|---|---|---|
| Cpd 1: C18 H18 Br N3 O3 | 0.255 | 403.0545 | 273854 | C18 H18 Br N3 O3 | 403.0532 | 3.32 |

MS Zoomed Spectrum

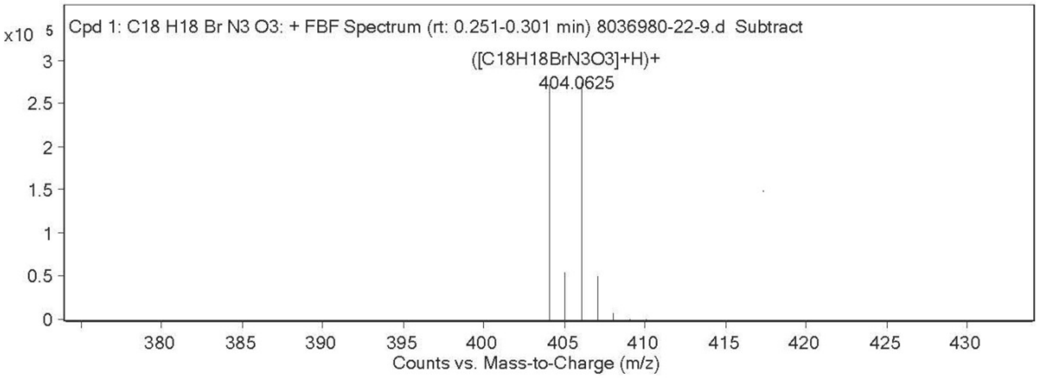


--- End Of Report ---

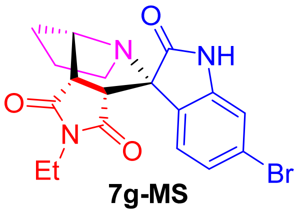


**7g-MS**

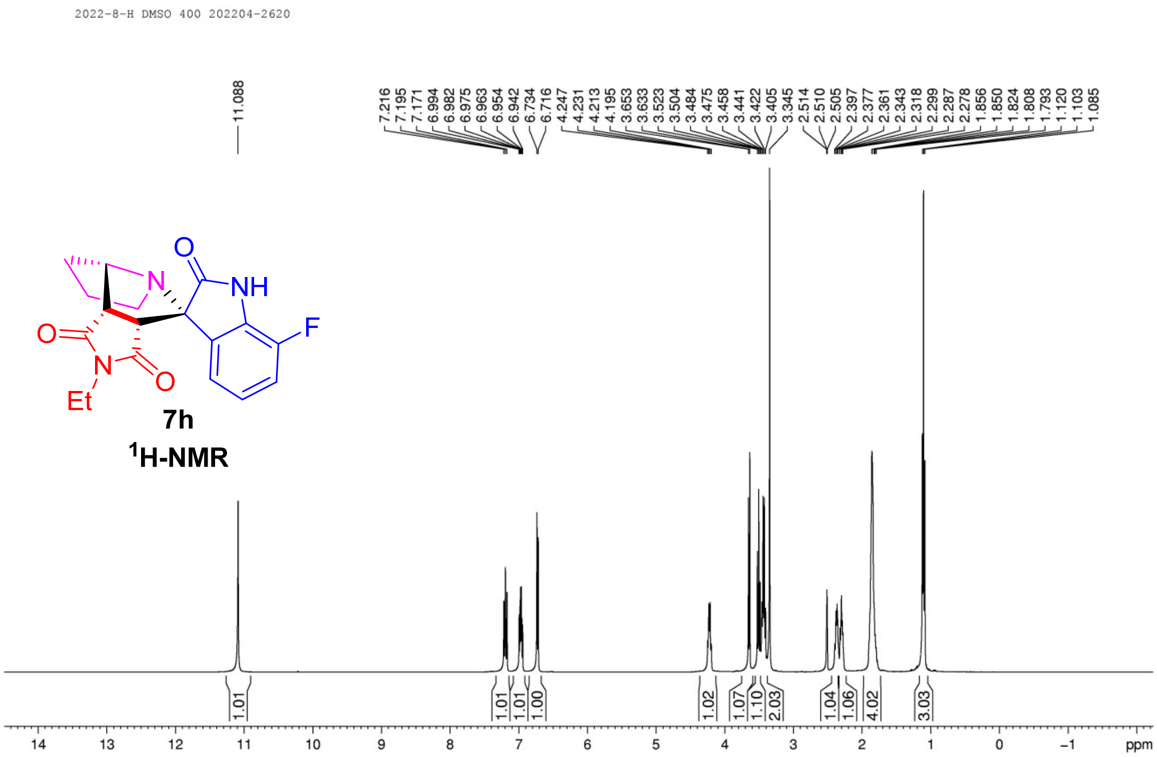
2022-8-H DMSO 400 202204-2620
11.088
7.216 7.195 7.171 6.994 6.982 6.975 6.963 6.954 6.942 6.734 6.716 4.247 4.231 4.213 4.195 3.653 3.633 3.523 3.504 3.484 3.475 3.458 3.441 3.422 3.405 3.345 2.514 2.510 2.505 2.397 2.377 2.361 2.343 2.318 2.299 2.287 2.278 1.856 1.850 1.824 1.808 1.793 1.120 1.103 1.085
O
NH
N
F
O
O
N
Et
7h
1H-NMR
1.01 1.01 1.01 1.00 1.02 1.07 1.10 2.03 1.04 1.06 4.02 3.03
14 13 12 11 10 9 8 7 6 5 4 3 2 1 0 −1 ppm

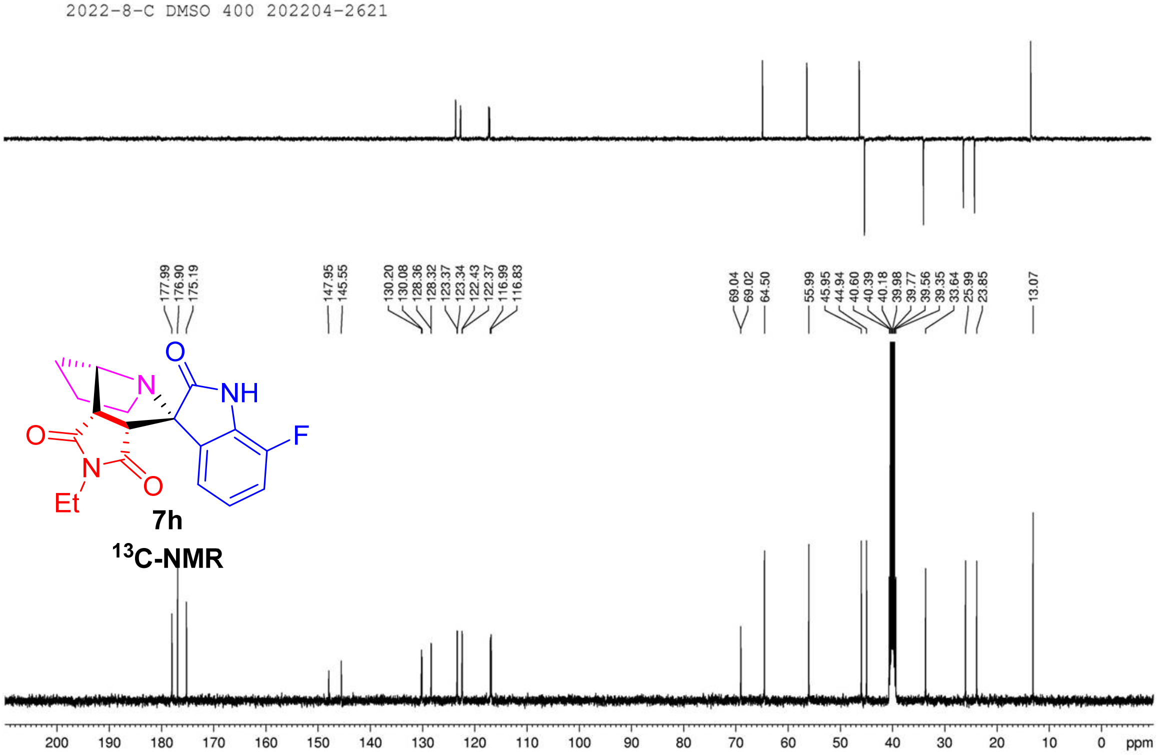
2022-8-C DMSO 400 202204-2621
177.99 176.90 175.19
147.95 145.55
130.20 130.08 128.36 128.32 123.37 123.34 122.43 122.37 116.99 116.83
69.04 69.02 64.50
55.99 45.95 44.94 40.60 40.39 40.18 39.98 39.77 39.56 39.35 33.64 25.99 23.85
13.07
O
NH
N
F
O
O
N
Et
7h
13C-NMR
200 190 180 170 160 150 140 130 120 110 100 90 80 70 60 50 40 30 20 10 0 ppm

## Qualitative Compound Report

| | | | |
|---|---|---|---|
| Data File | 2033368-22-8.d | Sample Name | 2033368-22-8 |
| Sample Type | Sample | Position | P1-A7 |
| Instrument Name | Instrument 1 | User Name | |
| Acq Method | pos-1min.m | Acquired Time | 2/6/2023 5:14:14 PM |
| IRM Calibration Status | Success | DA Method | QG-907.m |
| Comment | | | |
| Sample Group | | Info. | |
| Stream Name | LC 1 | Acquisition SW Version | 6200 series TOF/6500 series Q-TOF B.08.00 (B8058.0) |

**Compound Table**

| Compound Label | RT | Mass | Abund | Formula | Tgt Mass | Diff (ppm) |
|---|---|---|---|---|---|---|
| Cpd 1: C18 H18 F N3 O3 | 0.178 | 343.134 | 301648 | C18 H18 F N3 O3 | 343.1332 | 2.25 |

MS Zoomed Spectrum

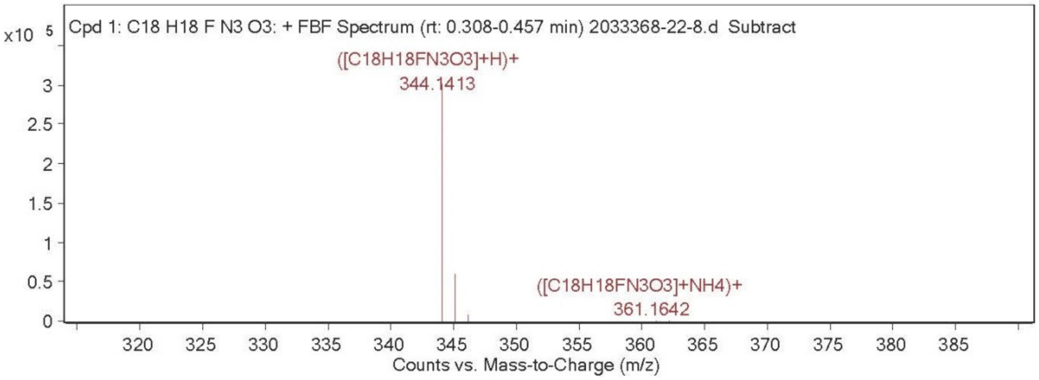


--- End Of Report ---

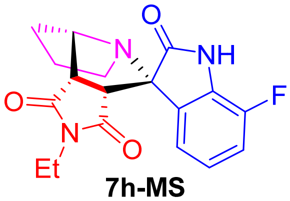


**7h-MS**

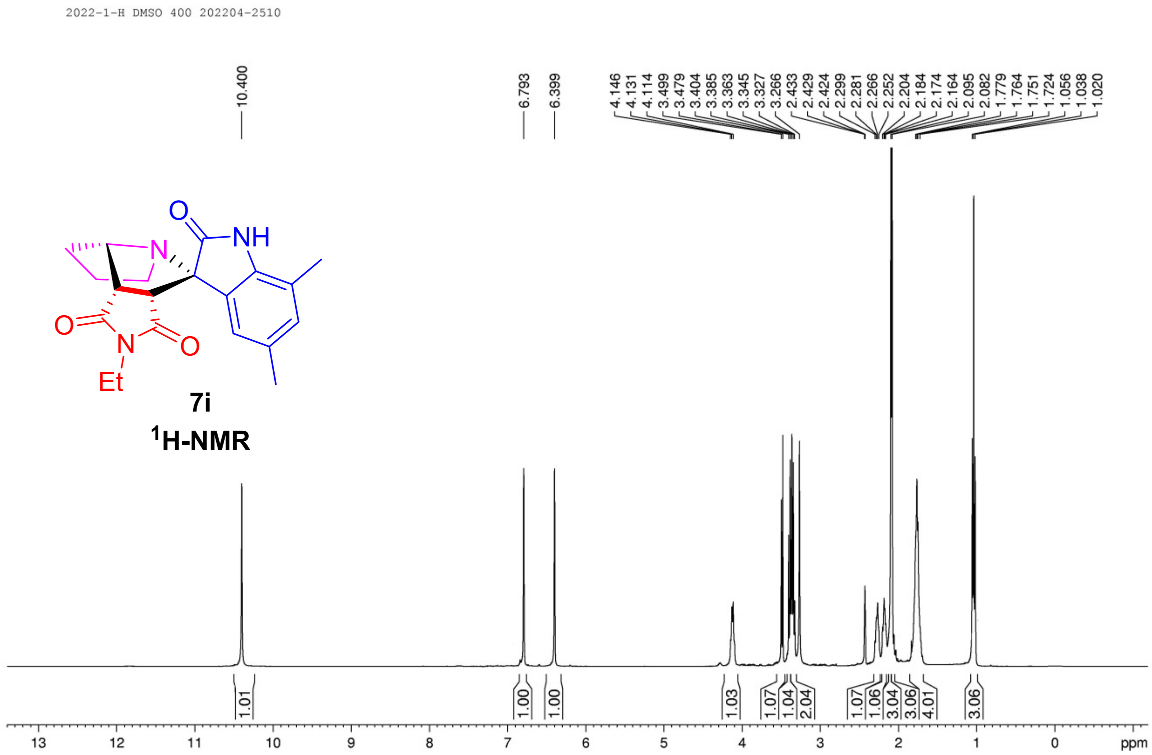
2022-1-H DMSO 400 202204-2510
10.400
6.793
6.399
4.146
4.131
4.114
3.499
3.479
3.404
3.385
3.363
3.345
3.327
3.266
2.433
2.429
2.424
2.299
2.281
2.266
2.252
2.204
2.184
2.174
2.164
2.095
2.082
1.779
1.764
1.751
1.724
1.056
1.038
1.020
O
NH
N
O
N
O
Et
7i
1H-NMR
1.01
1.00
1.00
1.03
1.07
1.04
2.04
1.07
1.06
3.04
3.06
4.01
3.06
13
12
11
10
9
8
7
6
5
4
3
2
1
0
ppm

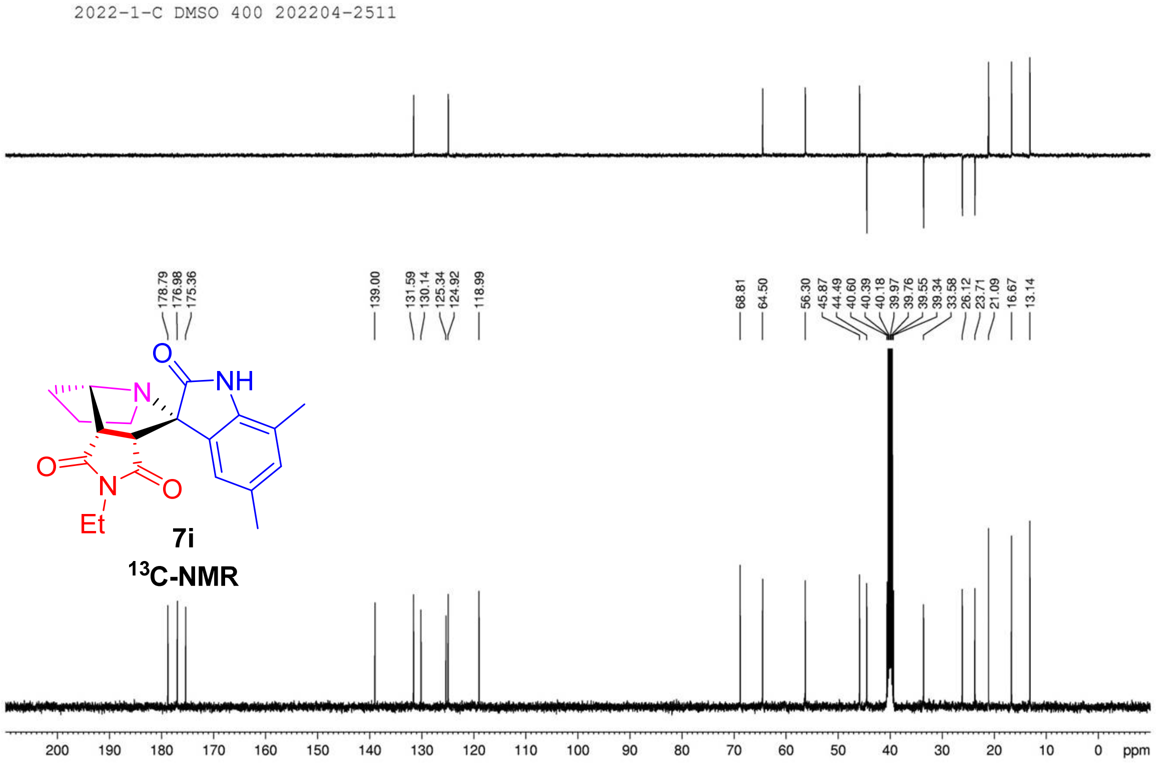
2022-1-C DMSO 400 202204-2511
178.79
176.98
175.36
139.00
131.59
130.14
125.34
124.92
118.99
68.81
64.50
56.30
45.87
44.49
40.60
40.39
40.18
39.97
39.76
39.55
39.34
33.58
26.12
23.71
21.09
16.67
13.14
O
NH
N
O
N
O
Et
7i
13C-NMR
200
190
180
170
160
150
140
130
120
110
100
90
80
70
60
50
40
30
20
10
0
ppm

## Qualitative Compound Report

| | | | |
|---|---|---|---|
| Data File | 2033368-22-1.d | Sample Name | 2033368-22-1 |
| Sample Type | Sample | Position | P1-A2 |
| Instrument Name | Instrument 1 | User Name | |
| Acq Method | pos-1min.m | Acquired Time | 2/6/2023 5:04:59 PM |
| IRM Calibration Status | Success | DA Method | QG-907.m |
| Comment | | | |
| Sample Group | | Info. | |
| Stream Name | LC 1 | Acquisition SW Version | 6200 series TOF/6500 series Q-TOF B.08.00 (B8058.0) |

**Compound Table**

| Compound Label | RT | Mass | Abund | Formula | Tgt Mass | Diff (ppm) |
|---|---|---|---|---|---|---|
| Cpd 1: C20 H23 N3 O3 | 0.497 | 353.1751 | 1250732 | C20 H23 N3 O3 | 353.1739 | 3.33 |

MS Zoomed Spectrum

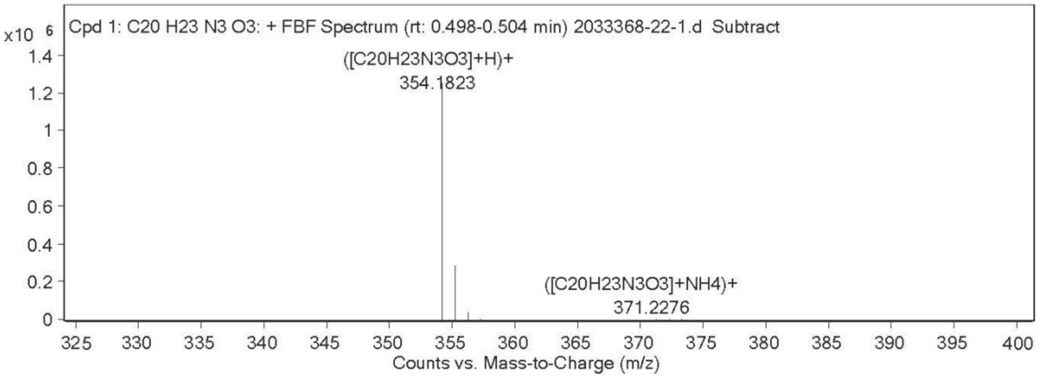


--- End Of Report ---

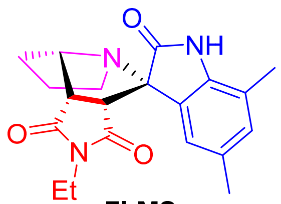


**7i-MS**

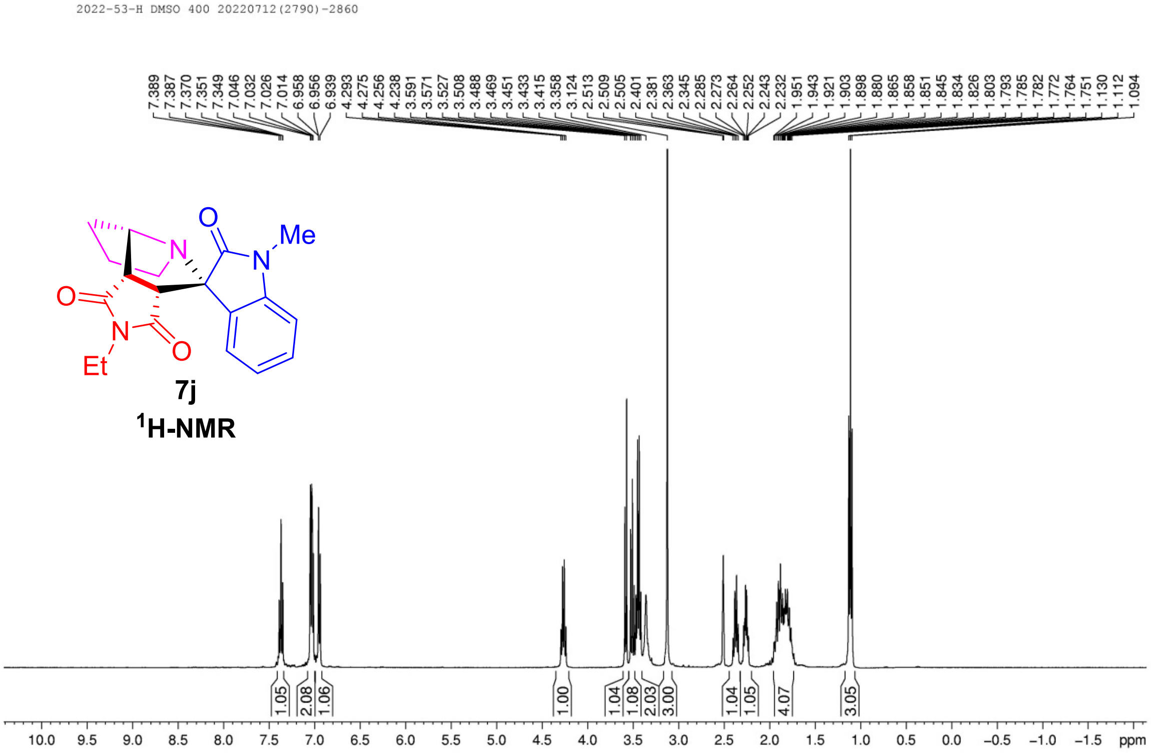
2022-53-H DMSO 400 20220712(2790)-2860
7.389 7.387 7.370 7.351 7.349 7.046 7.032 7.026 7.014 6.958 6.956 6.939 4.293 4.275 4.256 4.238 3.591 3.571 3.527 3.508 3.488 3.469 3.451 3.433 3.415 3.358 3.124 2.513 2.509 2.505 2.401 2.381 2.363 2.345 2.285 2.273 2.264 2.252 2.243 2.232 1.951 1.943 1.921 1.903 1.898 1.880 1.865 1.858 1.851 1.845 1.834 1.826 1.803 1.793 1.785 1.782 1.772 1.764 1.751 1.130 1.112 1.094
O
N
Me
O
N
Et
O
N
7j
1H-NMR
1.05 2.08 1.06 1.00 1.04 1.08 2.03 3.00 1.04 1.05 4.07 3.05
ppm

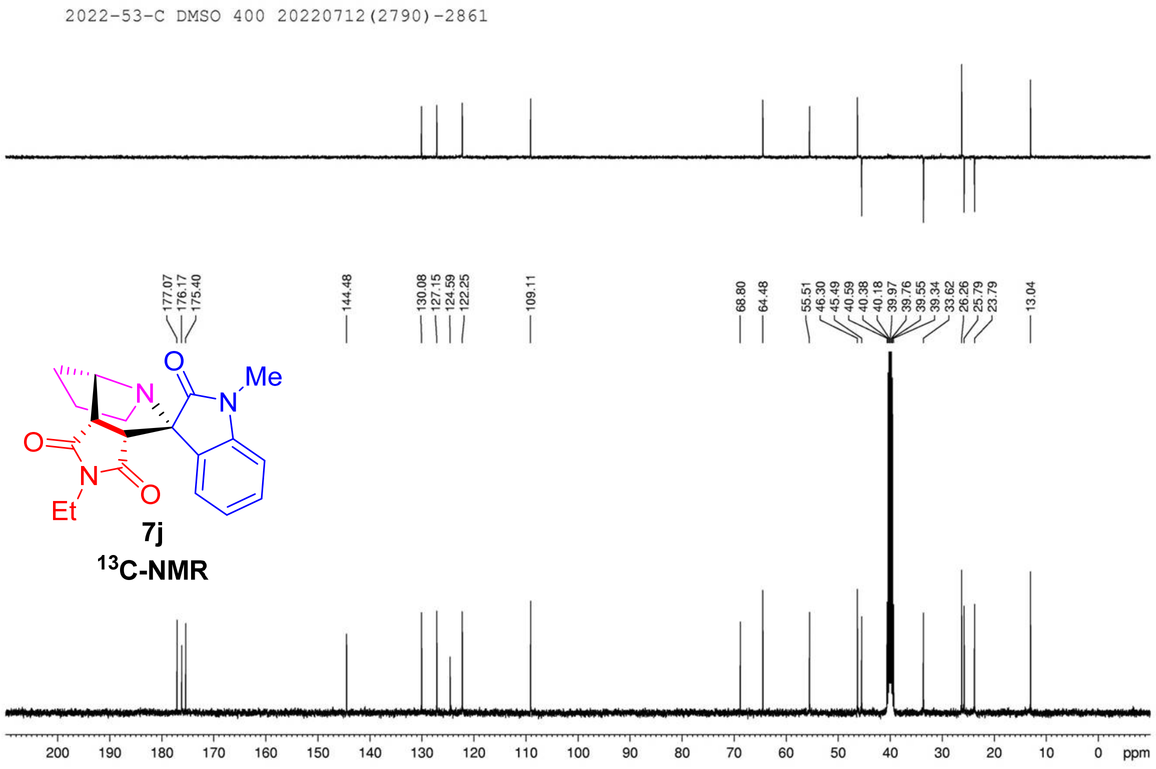
2022-53-C DMSO 400 20220712(2790)-2861
177.07 176.17 175.40 144.48 130.08 127.15 124.59 122.25 109.11 68.80 64.48 55.51 46.30 45.49 40.59 40.38 40.18 39.97 39.76 39.55 39.34 33.62 26.26 25.79 23.79 13.04
O
N
Me
O
N
Et
O
N
7j
13C-NMR
ppm

## Qualitative Compound Report

| | | | |
|---|---|---|---|
| **Data File** | 2033368-22-53.d | **Sample Name** | 2033368-22-53 |
| **Sample Type** | Sample | **Position** | P1-C6 |
| **Instrument Name** | Instrument 1 | **User Name** | |
| **Acq Method** | pos-1min.m | **Acquired Time** | 2/6/2023 5:46:00 PM |
| **IRM Calibration Status** | Success | **DA Method** | QG-907.m |
| **Comment** | | | |
| **Sample Group** | | **Info.** | |
| **Stream Name** | LC 1 | **Acquisition SW Version** | 6200 series TOF/6500 series Q-TOF B.08.00 (B8058.0) |

**Compound Table**

| Compound Label | RT | Mass | Abund | Formula | Tgt Mass | Diff (ppm) |
|---|---|---|---|---|---|---|
| Cpd 1: C19 H21 N3 O3 | 0.533 | 339.1592 | 795267 | C19 H21 N3 O3 | 339.1583 | 2.57 |

MS Zoomed Spectrum

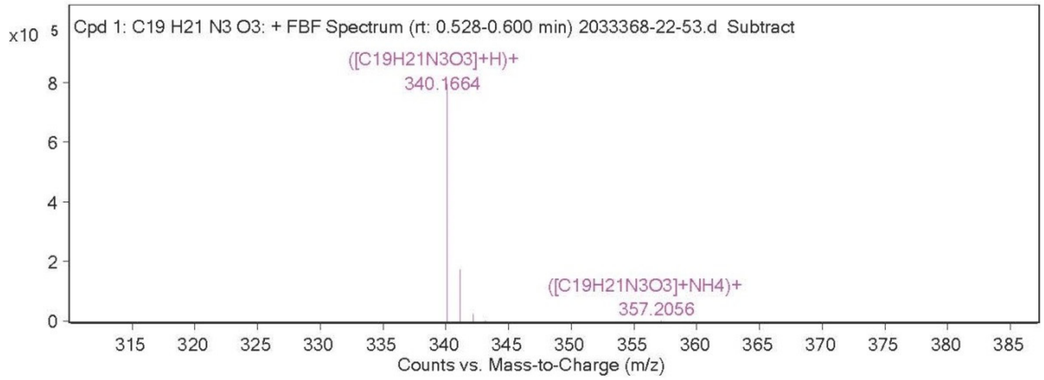


--- End Of Report ---

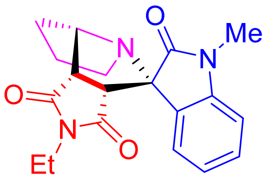


**7j-MS**

2022-6-H DMSO 400 202204-2660

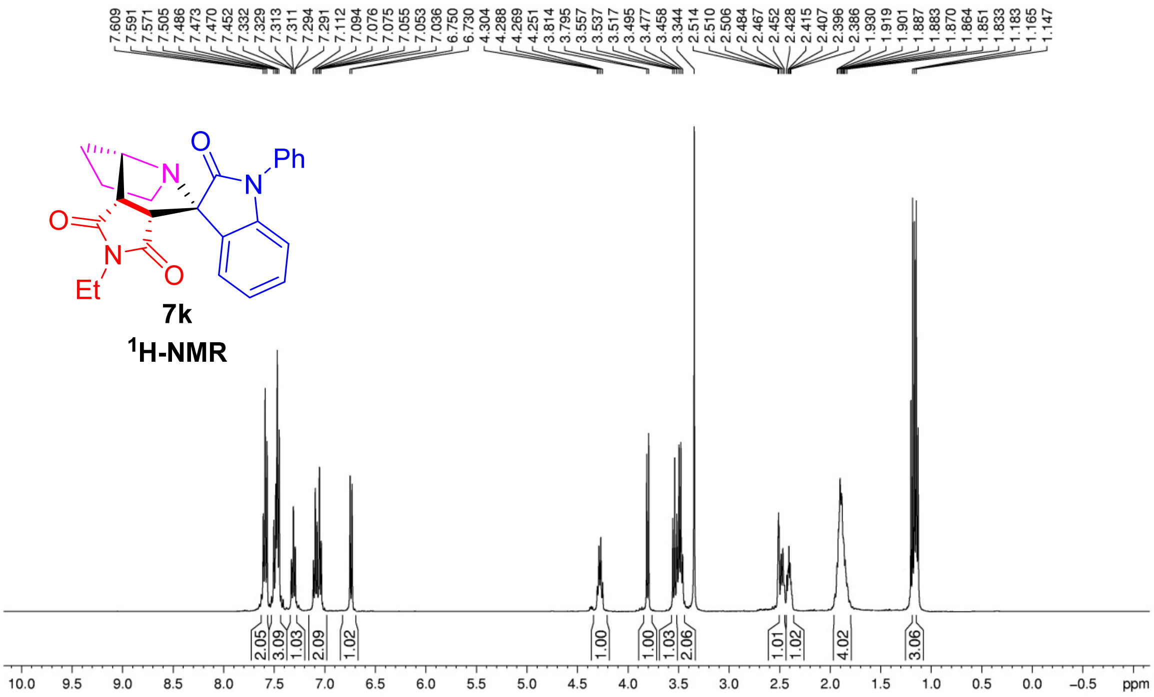


2022-6-C DMSO 400 202204-2661

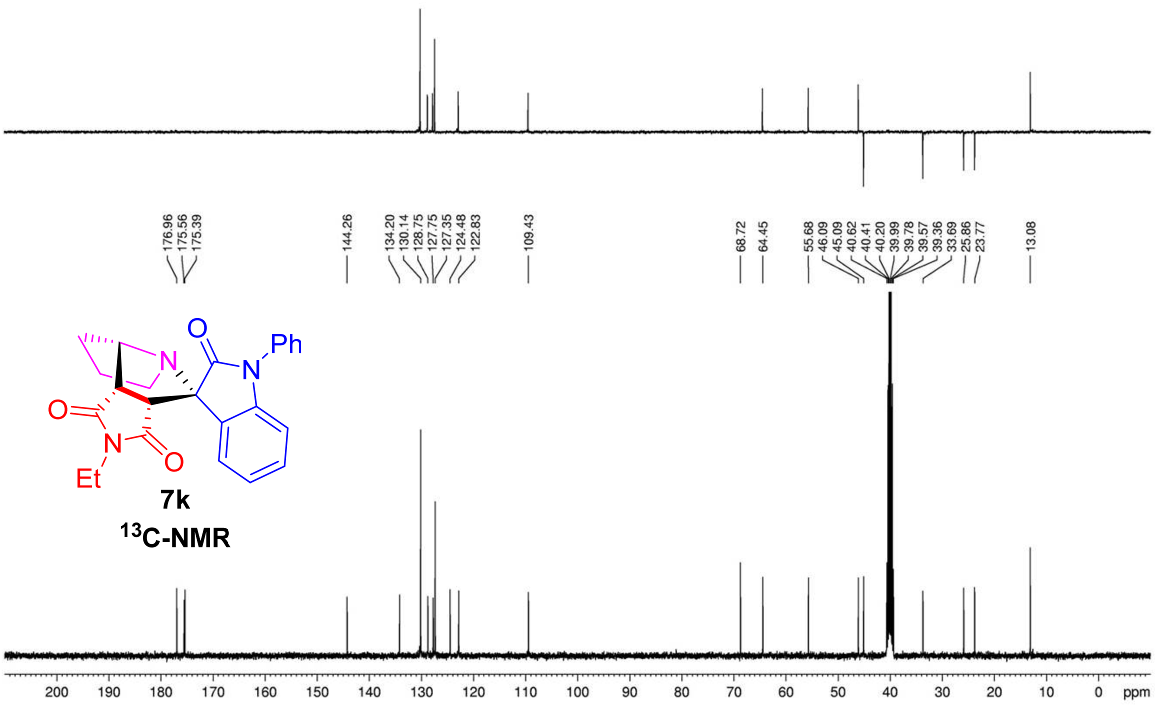

## Qualitative Compound Report

| | | | |
|---|---|---|---|
| Data File | 8036980-22-36.d | Sample Name | 8036980-22-36 |
| Sample Type | Sample | Position | P1-D5 |
| Instrument Name | Instrument 1 | User Name | |
| Acq Method | pos-1min.m | Acquired Time | 8/8/2022 5:40:40 PM |
| IRM Calibration Status | Success | DA Method | default.m |
| Comment | | | |
| Sample Group | | Info. | |
| Stream Name | LC 1 | Acquisition SW Version | 6200 series TOF/6500 series Q-TOF B.08.00 (B8058.0) |

**Compound Table**

| Compound Label | RT | Mass | Abund | Formula | Tgt Mass | Diff (ppm) |
|---|---|---|---|---|---|---|
| Cpd 1: C24 H23 N3 O3 | 0.255 | 401.0545 | 273254 | C24 H23 N3 O3 | 401.0532 | 2.32 |

MS Zoomed Spectrum

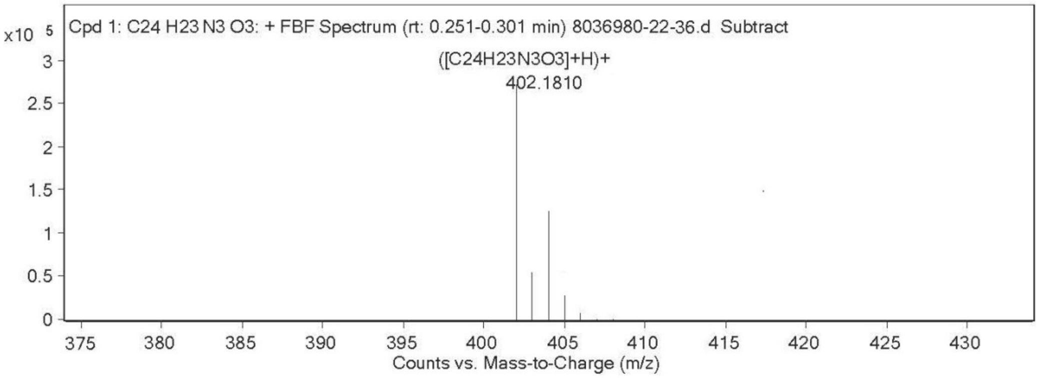


--- End Of Report ---

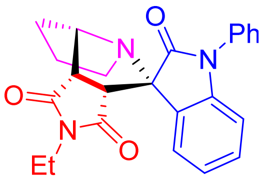


**7k-MS**

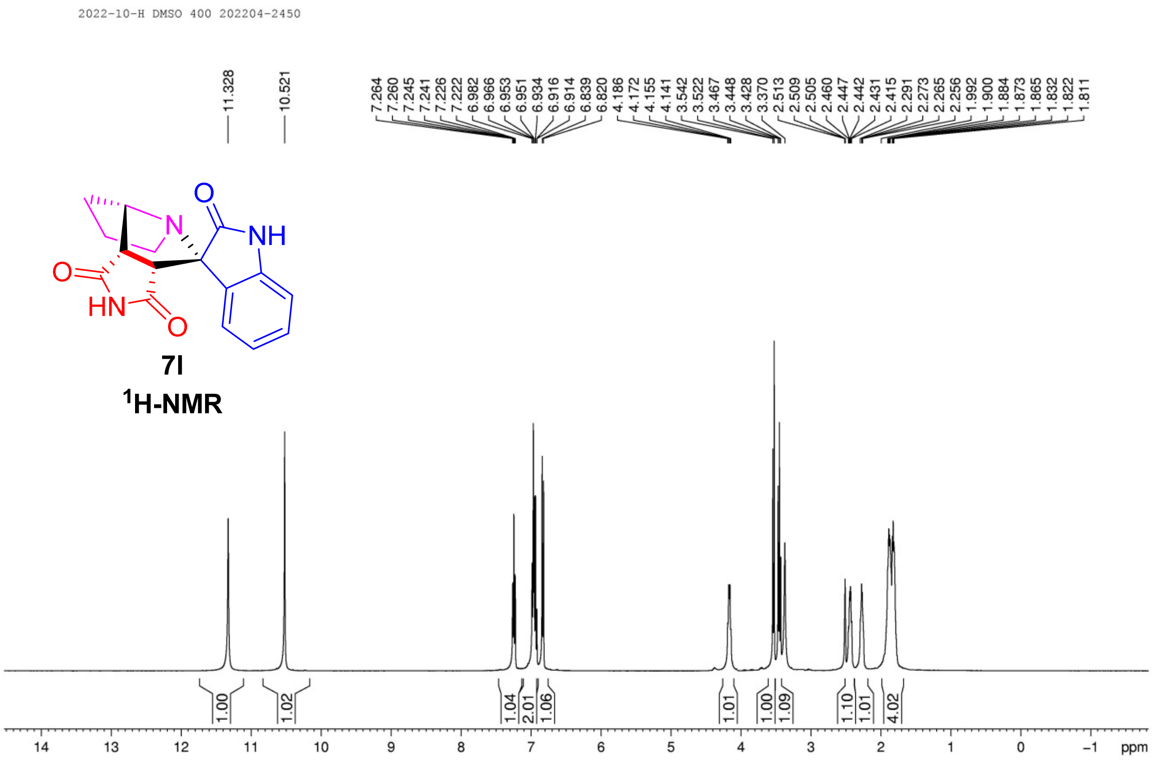
2022-10-H DMSO 400 202204-2450
11.328
10.521
7.264 7.260 7.245 7.241 7.226 7.222 6.982 6.966 6.953 6.951 6.934 6.916 6.914 6.839 6.820 4.186 4.172 4.155 4.141 3.542 3.522 3.467 3.448 3.428 3.370 2.513 2.509 2.505 2.460 2.447 2.442 2.431 2.415 2.291 2.273 2.265 2.256 1.992 1.900 1.884 1.873 1.865 1.832 1.822 1.811
O
N
NH
O
HN
O
7l
1H-NMR
1.00 1.02 1.04 2.01 1.06 1.01 1.00 1.09 1.10 1.01 4.02
14 13 12 11 10 9 8 7 6 5 4 3 2 1 0 –1 ppm

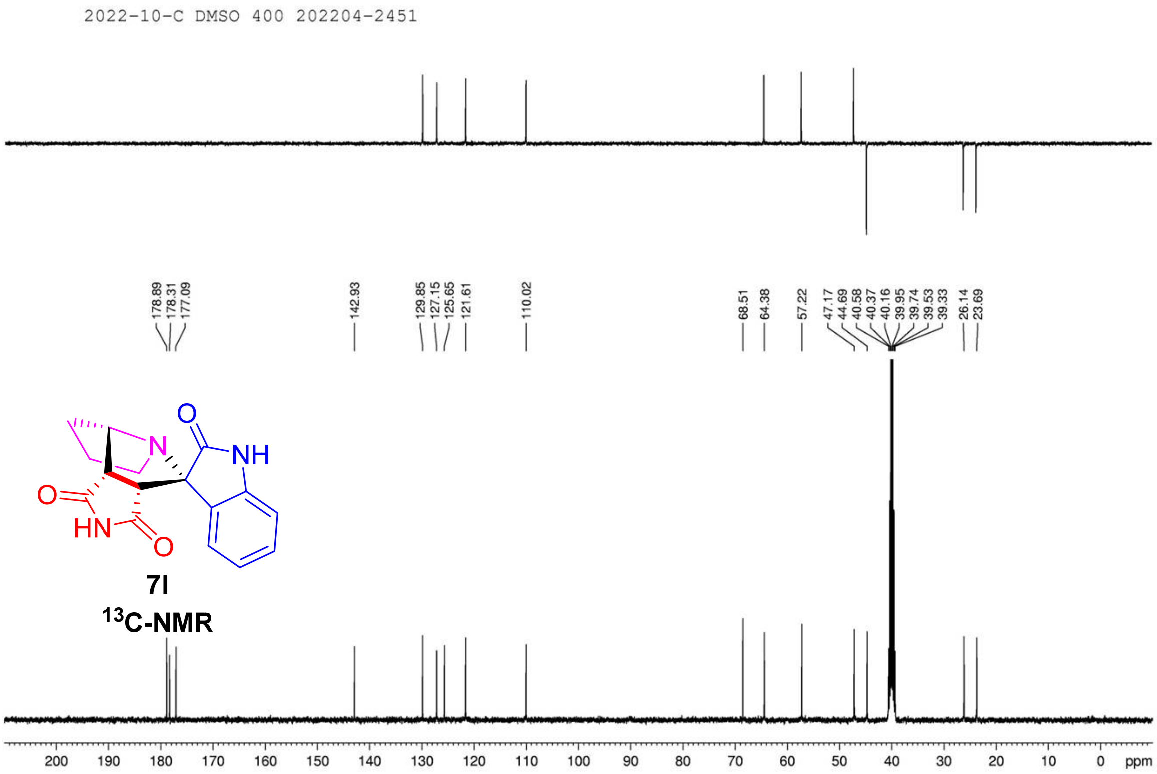
2022-10-C DMSO 400 202204-2451
178.89 178.31 177.09
142.93
129.85 127.15 125.65 121.61
110.02
68.51 64.38
57.22
47.17 44.69 40.58 40.37 40.16 39.95 39.74 39.53 39.33
26.14 23.69
O
N
NH
O
HN
O
7l
13C-NMR
200 190 180 170 160 150 140 130 120 110 100 90 80 70 60 50 40 30 20 10 0 ppm

# Qualitative Compound Report

| | | | |
|---|---|---|---|
| Data File | 2033368-22-44.d | Sample Name | 2033368-22-44 |
| Sample Type | Sample | Position | P1-C7 |
| Instrument Name | Instrument 1 | User Name | |
| Acq Method | pos-1min.m | Acquired Time | 2/6/2023 5:47:47 PM |
| IRM Calibration Status | Success | DA Method | QG-907.m |
| Comment | | | |
| Sample Group | | Info. | |
| Stream Name | LC 1 | Acquisition SW Version | 6200 series TOF/6500 series Q-TOF B.08.00 (B8058.0) |

**Compound Table**

| Compound Label | RT | Mass | Abund | Formula | Tgt Mass | Diff (ppm) |
|---|---|---|---|---|---|---|
| Cpd 1: C16 H15 N3 O3 | 0.112 | 297.0779 | 85285 | C16 H15 N3 O3 | 297.0771 | 2.71 |

MS Zoomed Spectrum

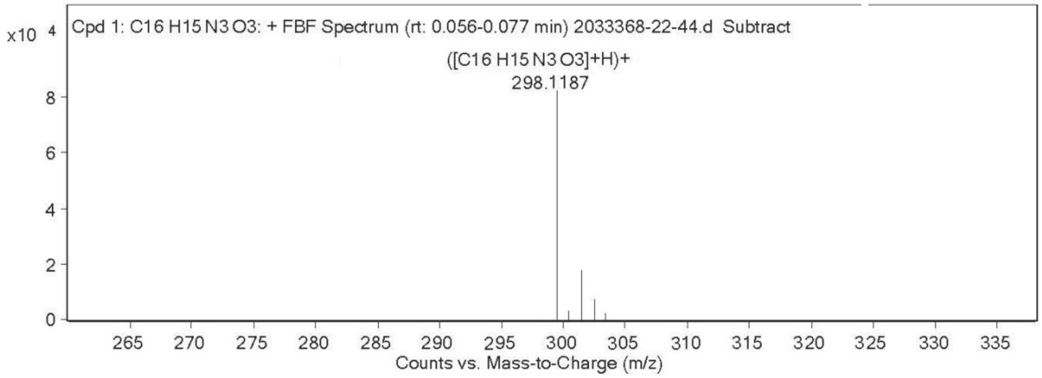


--- End Of Report ---

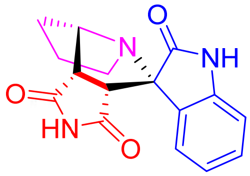


**7l-MS**

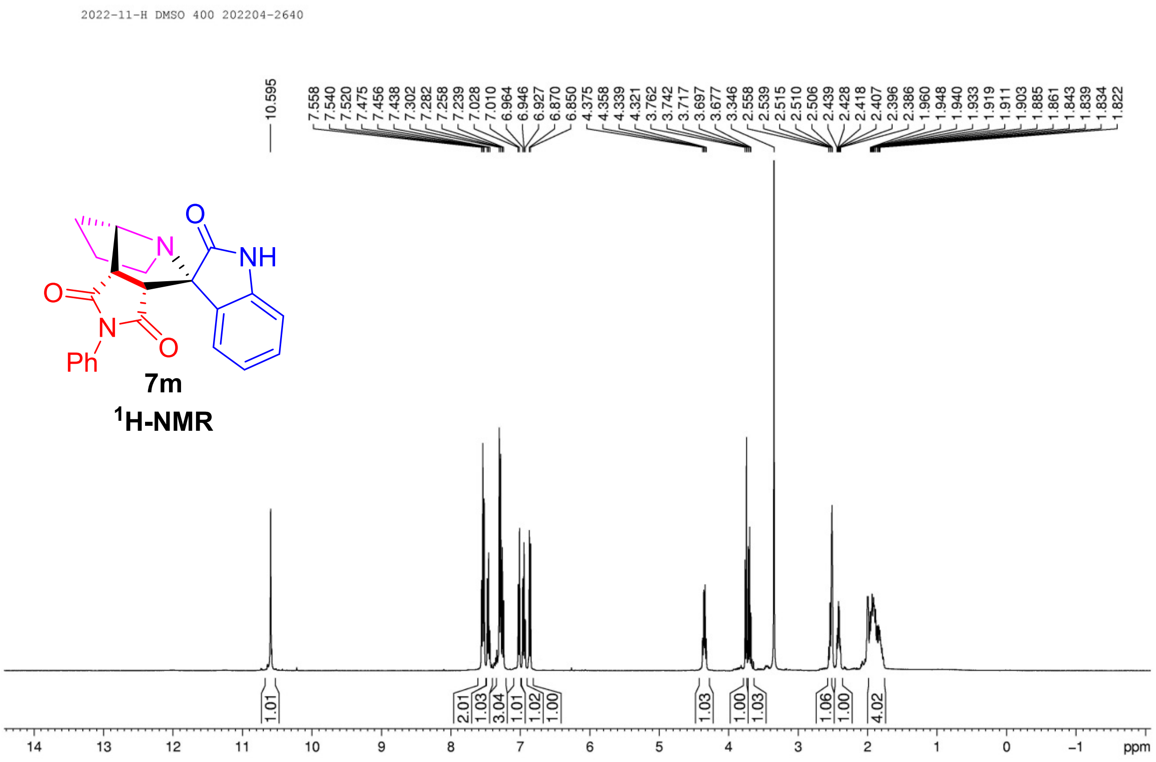

2022-11-H DMSO 400 202204-2640
10.595
7.558 7.540 7.520 7.475 7.456 7.438 7.302 7.282 7.258 7.239 7.028 7.010 6.964 6.946 6.927 6.870 6.850 4.375 4.358 4.339 4.321 3.762 3.742 3.717 3.697 3.677 3.346 2.558 2.539 2.515 2.510 2.506 2.439 2.428 2.418 2.407 2.396 2.386 1.960 1.948 1.940 1.933 1.919 1.911 1.903 1.885 1.861 1.843 1.839 1.834 1.822
O
NH
N
O
N
O
Ph
7m
1H-NMR
1.01
2.01 1.03 3.04 1.01 1.02 1.00
1.03
1.00 1.03
1.06 1.00
4.02
14 13 12 11 10 9 8 7 6 5 4 3 2 1 0 –1 ppm


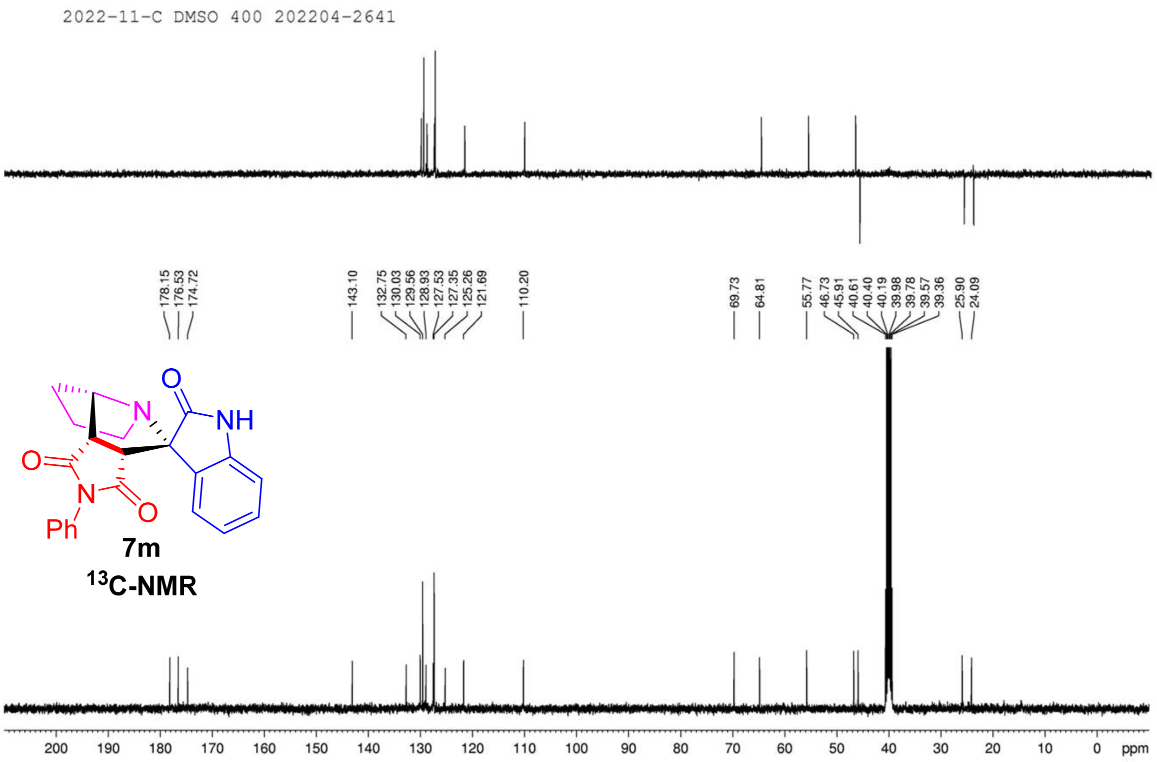

2022-11-C DMSO 400 202204-2641
178.15 176.53 174.72
143.10
132.75 130.03 129.56 128.93 127.53 127.35 125.26 121.69
110.20
69.73 64.81
55.77 46.73 45.91 40.61 40.40 40.19 39.98 39.78 39.57 39.36 25.90 24.09
O
NH
N
O
N
O
Ph
7m
13C-NMR
200 190 180 170 160 150 140 130 120 110 100 90 80 70 60 50 40 30 20 10 0 ppm

## Qualitative Compound Report

| | | | |
|---|---|---|---|
| Data File | 2033368-22-11.d | Sample Name | 2033368-22-11 |
| Sample Type | Sample | Position | P1-A2 |
| Instrument Name | Instrument 1 | User Name | |
| Acq Method | pos-1min.m | Acquired Time | 2/6/2023 5:34:23 PM |
| IRM Calibration Status | Success | DA Method | QG-907.m |
| Comment | | | |
| Sample Group | | Info. | |
| Stream Name | LC 1 | Acquisition SW Version | 6200 series TOF/6500 series Q-TOF B.08.00 (B8058.0) |

**Compound Table**

| Compound Label | RT | Mass | Abund | Formula | Tgt Mass | Diff (ppm) |
|---|---|---|---|---|---|---|
| Cpd 1: C22 H19 N3 O3 | 0.475 | 373.1737 | 1250725 | C22 H19 N3 O3 | 373.1751 | 3.17 |

MS Zoomed Spectrum

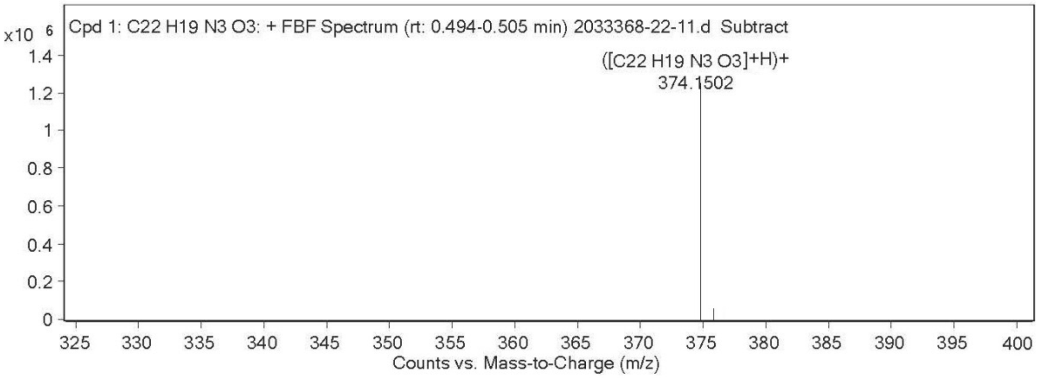


--- End Of Report ---

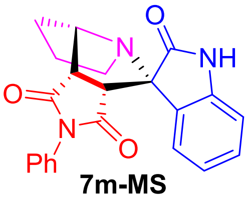


**7m-MS**

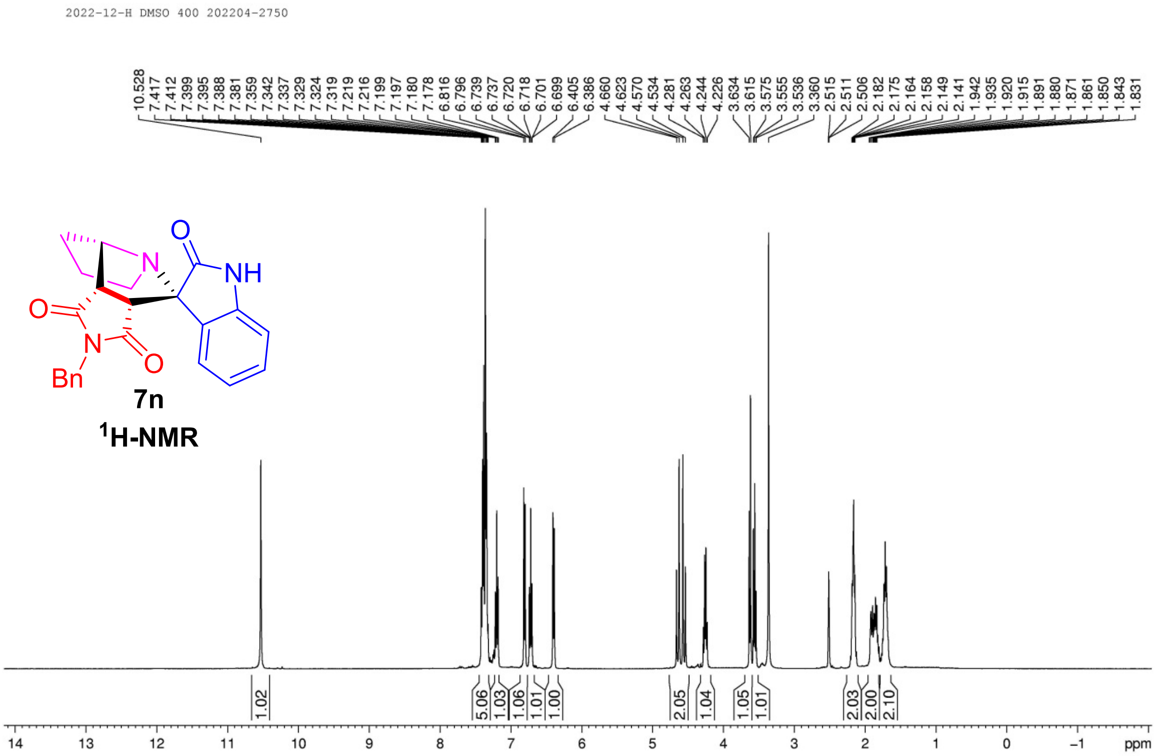
2022-12-H DMSO 400 202204-2750
10.528 7.417 7.412 7.399 7.395 7.388 7.381 7.359 7.342 7.337 7.329 7.324 7.319 7.219 7.216 7.199 7.197 7.180 7.178 6.816 6.796 6.739 6.737 6.720 6.718 6.701 6.699 6.405 6.386 4.660 4.623 4.570 4.534 4.281 4.263 4.244 4.226 3.634 3.615 3.575 3.555 3.536 3.360 2.515 2.511 2.506 2.182 2.175 2.164 2.158 2.149 2.141 1.942 1.935 1.920 1.915 1.891 1.880 1.871 1.861 1.850 1.843 1.831
O
N
NH
O
N
O
Bn
7n
1H-NMR
1.02
5.06
1.03
1.06
1.01
1.00
2.05
1.04
1.05
1.01
2.03
2.00
2.10
14 13 12 11 10 9 8 7 6 5 4 3 2 1 0 −1 ppm

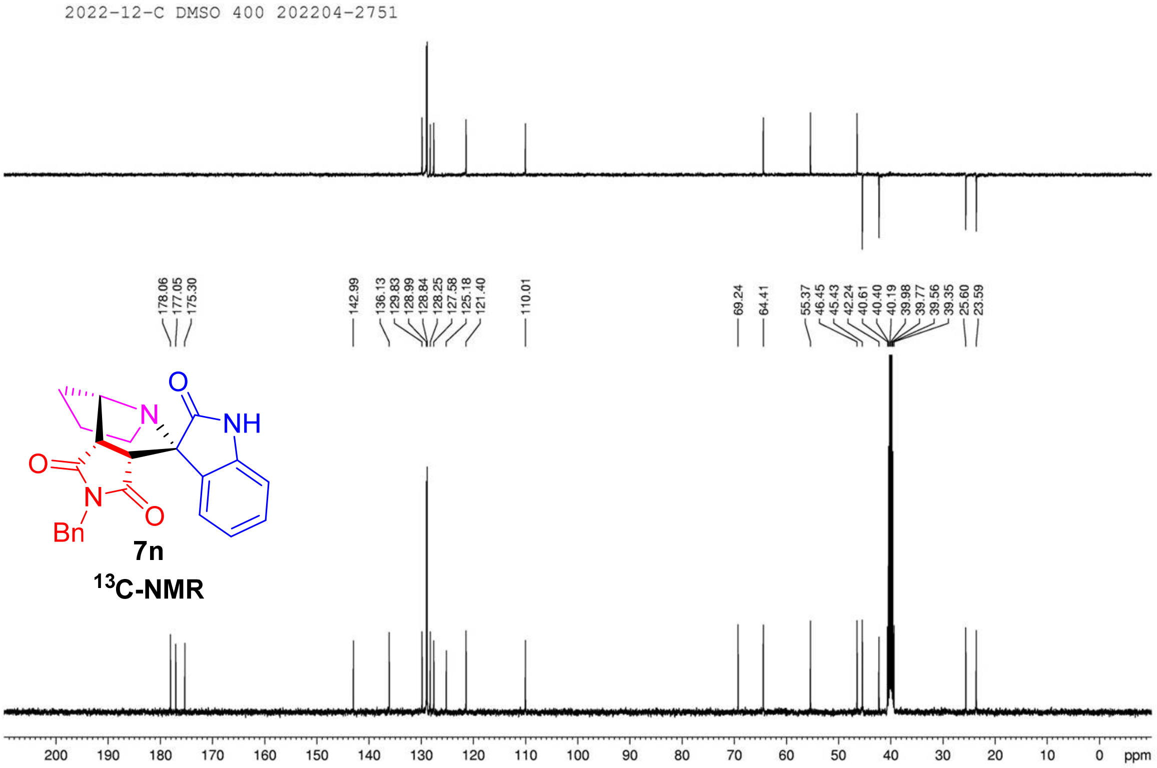
2022-12-C DMSO 400 202204-2751
178.06 177.05 175.30
142.99
136.13 129.83 128.99 128.84 128.25 127.58 125.18 121.40
110.01
69.24
64.41
55.37 46.45 45.43 42.24 40.61 40.40 40.19 39.98 39.77 39.56 39.35 25.60 23.59
O
N
NH
O
N
O
Bn
7n
13C-NMR
200 190 180 170 160 150 140 130 120 110 100 90 80 70 60 50 40 30 20 10 0 ppm

## Qualitative Compound Report

| | | | |
|---|---|---|---|
| Data File | 2033368-22-111.d | Sample Name | 2033368-22-111 |
| Sample Type | Sample | Position | P1-A2 |
| Instrument Name | Instrument 1 | User Name | |
| Acq Method | pos-1min.m | Acquired Time | 2/6/2023 5:23:20 PM |
| IRM Calibration Status | Success | DA Method | QG-907.m |
| Comment | | | |
| Sample Group | | Info. | |
| Stream Name | LC 1 | Acquisition SW Version | 6200 series TOF/6500 series Q-TOF B.08.00 (B8058.0) |

**Compound Table**

| Compound Label | RT | Mass | Abund | Formula | Tgt Mass | Diff (ppm) |
|---|---|---|---|---|---|---|
| Cpd 1: C23 H21 N3 O3 | 0.453 | 387.1739 | 1250125 | C23 H21 N3 O3 | 387.1751 | 3.12 |

MS Zoomed Spectrum

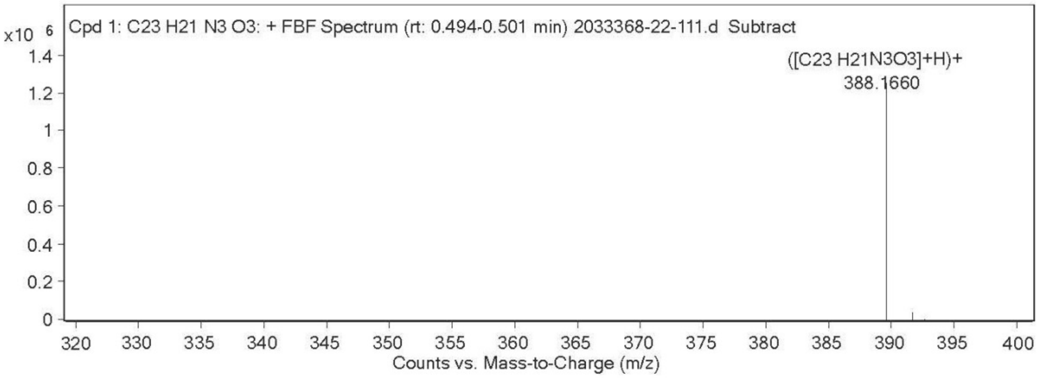


--- End Of Report ---

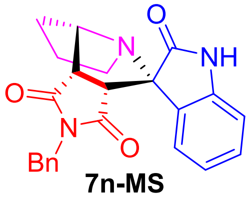


**7n-MS**

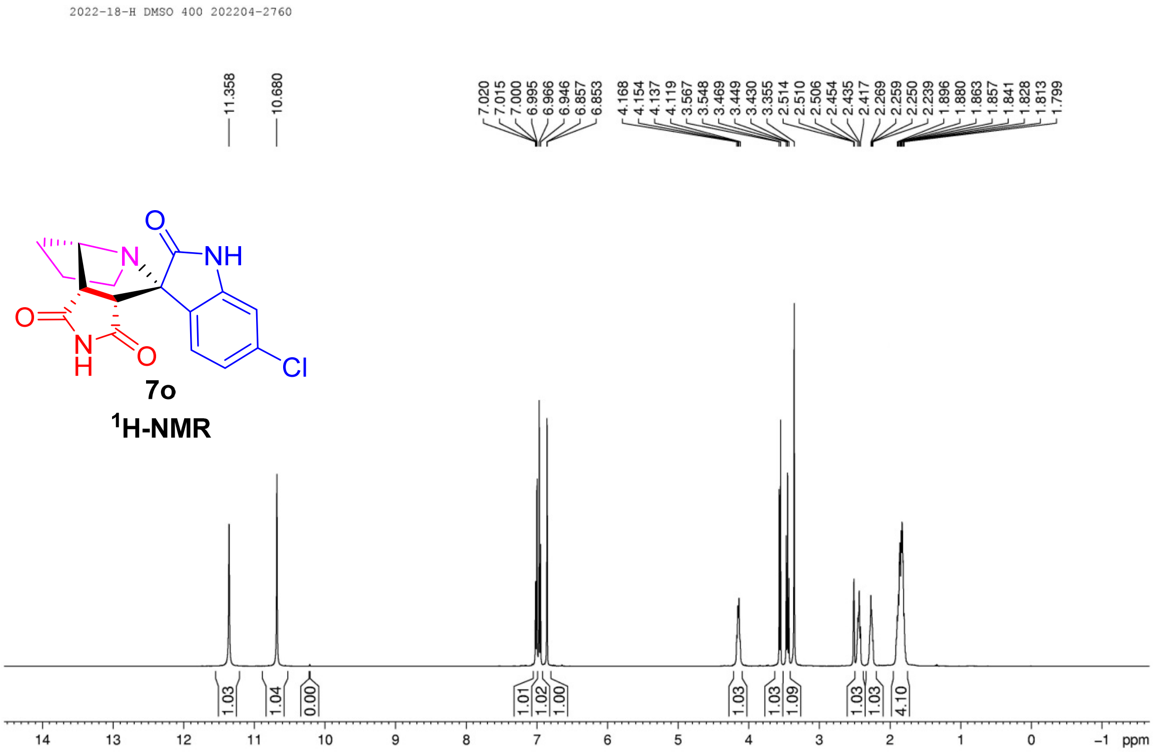
2022-18-H DMSO 400 202204-2760
11.358
10.680
7.020 7.015 7.000 6.995 6.966 6.946 6.857 6.853
4.168 4.154 4.137 4.119 3.567 3.548 3.469 3.449 3.430 3.355 2.514 2.510 2.506 2.454 2.435 2.417 2.269 2.259 2.250 2.239 1.896 1.880 1.863 1.857 1.841 1.828 1.813 1.799
O
NH
N
O
N
H
O
Cl
7o
1H-NMR
1.03 1.04 0.00 1.01 1.02 1.00 1.03 1.03 1.09 1.03 1.03 4.10
14 13 12 11 10 9 8 7 6 5 4 3 2 1 0 −1 ppm

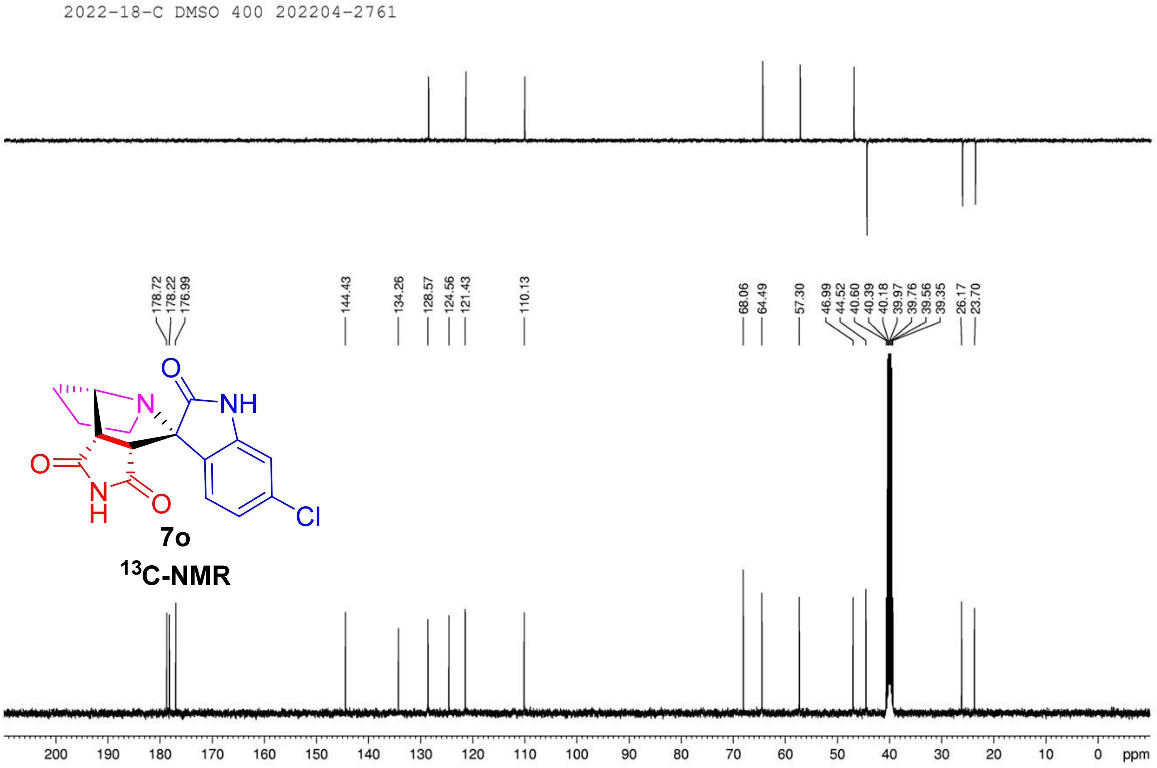
2022-18-C DMSO 400 202204-2761
178.72 178.22 176.99
144.43
134.26 128.57 124.56 121.43
110.13
68.06 64.49
57.30
46.99 44.52 40.60 40.39 40.18 39.97 39.76 39.56 39.35
26.17 23.70
O
NH
N
O
N
H
O
Cl
7o
13C-NMR
200 190 180 170 160 150 140 130 120 110 100 90 80 70 60 50 40 30 20 10 0 ppm

## Qualitative Compound Report

| | | | |
|---|---|---|---|
| Data File | 2033368-22-64.d | Sample Name | 2033368-22-64 |
| Sample Type | Sample | Position | P1-C2 |
| Instrument Name | Instrument 1 | User Name | |
| Acq Method | pos-1min.m | Acquired Time | 2/6/2023 5:23:47 PM |
| IRM Calibration Status | Success | DA Method | QG-905.m |
| Comment | | | |
| Sample Group | | Info. | |
| Stream Name | LC 1 | Acquisition SW Version | 6200 series TOF/6500 series Q-TOF B.08.00 (B8058.0) |

**Compound Table**

| Compound Label | RT | Mass | Abund | Formula | Tgt Mass | Diff (ppm) |
|---|---|---|---|---|---|---|
| Cpd 1: C16 H14 Cl N3 O3 | 0.132 | 331.0778 | 85779 | C16 H14 Cl N3 O3 | 331.0773 | 2.71 |

MS Zoomed Spectrum

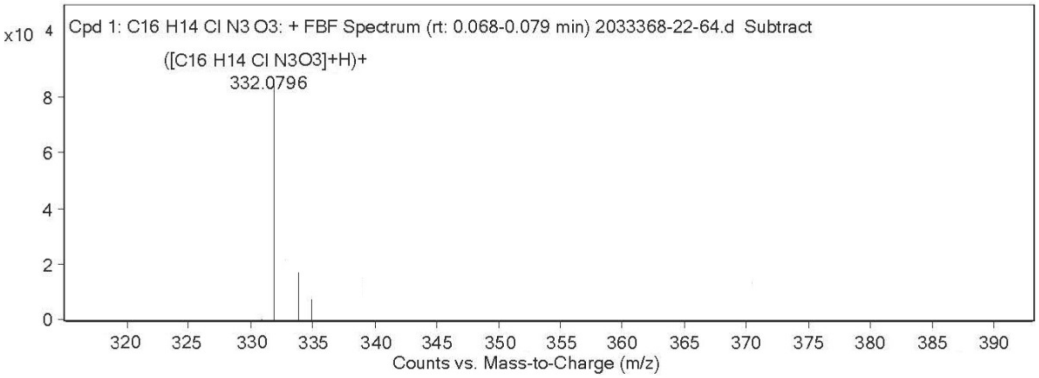


--- End Of Report ---

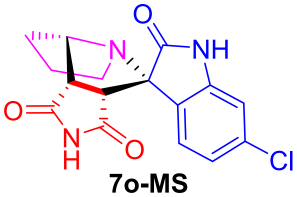


**7o-MS**

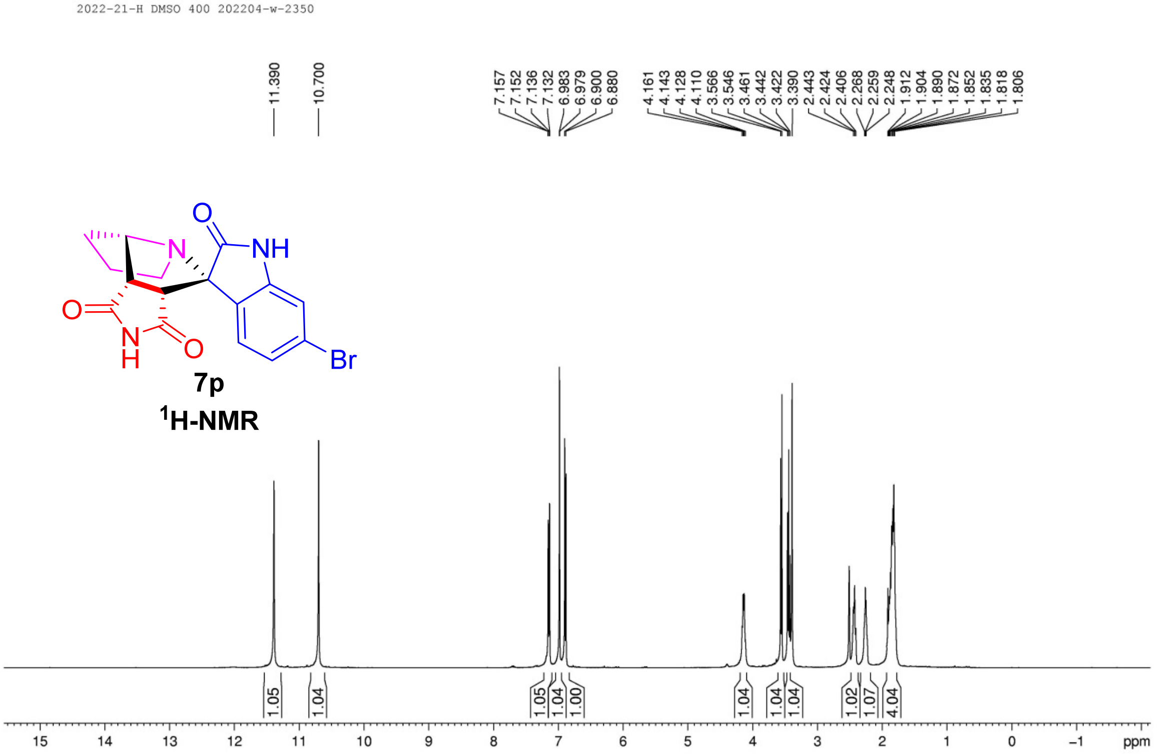
2022-21-H DMSO 400 202204-w-2350
11.390
10.700
7.157 7.152 7.136 7.132 6.983 6.979 6.900 6.880
4.161 4.143 4.128 4.110 3.566 3.546 3.461 3.442 3.422 3.390 2.443 2.424 2.406 2.268 2.259 2.248 1.912 1.904 1.890 1.872 1.852 1.835 1.818 1.806
O
NH
N
O
N
H
O
Br
7p
1H-NMR
1.05 1.04 1.05 1.04 1.00 1.04 1.04 1.04 1.02 1.07 4.04
15 14 13 12 11 10 9 8 7 6 5 4 3 2 1 0 −1 ppm

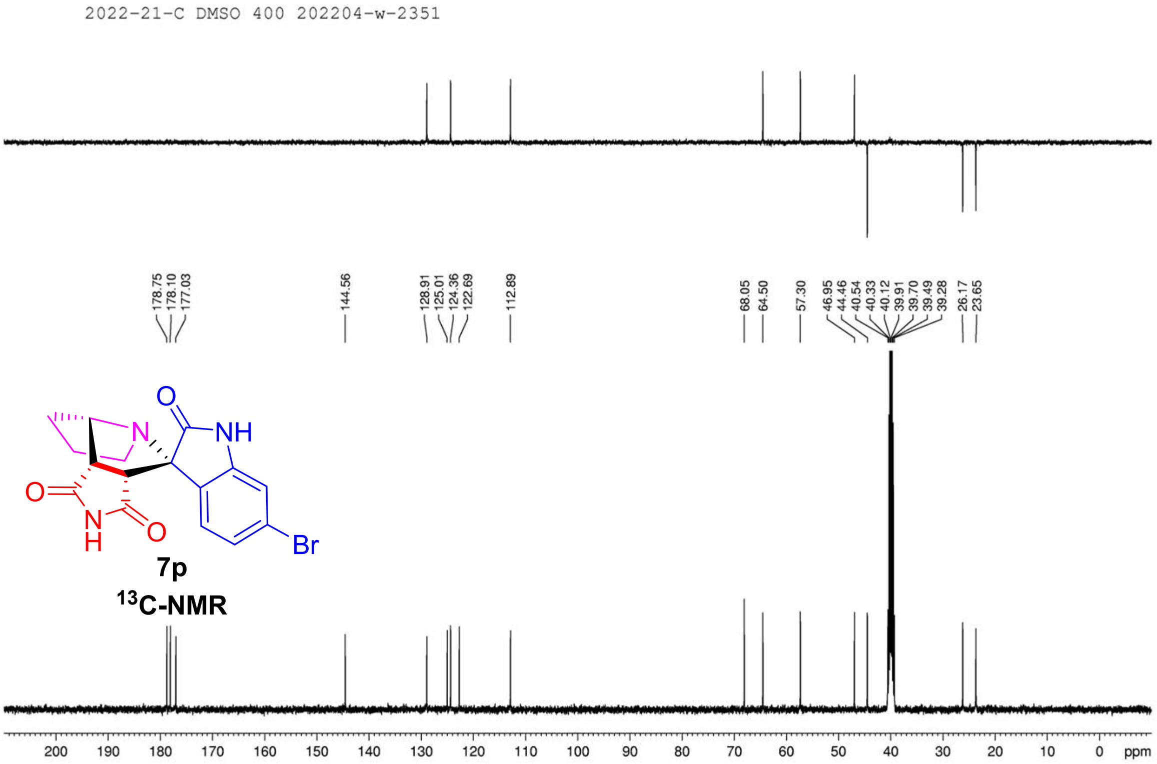
2022-21-C DMSO 400 202204-w-2351
178.75 178.10 177.03
144.56
128.91 125.01 124.36 122.69
112.89
68.05 64.50 57.30 46.95 44.46 40.54 40.33 40.12 39.91 39.70 39.49 39.28 26.17 23.65
O
NH
N
O
N
H
O
Br
7p
13C-NMR
200 190 180 170 160 150 140 130 120 110 100 90 80 70 60 50 40 30 20 10 0 ppm

# Qualitative Compound Report

| | | | |
|---|---|---|---|
| Data File | 2033368-22-21-NEG.d | Sample Name | 2033368-22-21 |
| Sample Type | Sample | Position | P1-B4 |
| Instrument Name | Instrument 1 | User Name | |
| Acq Method | neg-1min.m | Acquired Time | 2/8/2023 10:30:34 AM |
| IRM Calibration Status | Success | DA Method | QG-907.m |
| Comment | | | |
| Sample Group | | Info. | |
| Stream Name | LC 1 | Acquisition SW Version | 6200 series TOF/6500 series Q-TOF B.08.00 (B8058.0) |

**Compound Table**

| Compound Label | RT | Mass | Abund | Formula | Tgt Mass | Diff (ppm) |
|---|---|---|---|---|---|---|
| Cpd 1: C16 H14 Br N3 O3 | 0.628 | 375.0211 | 155 | C16 H14 Br N3 O3 | 375.0219 | -2.01 |

MS Zoomed Spectrum

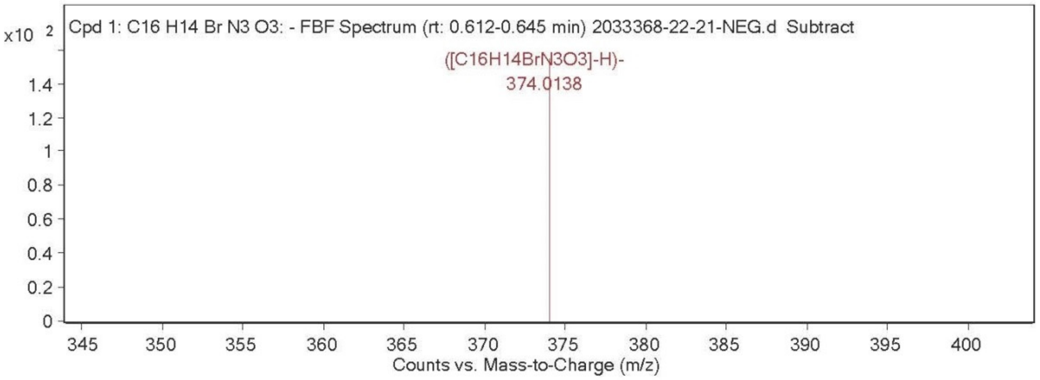


--- End Of Report ---

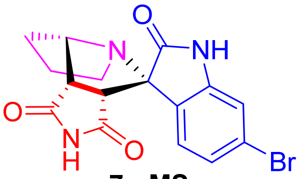


**7p-MS**

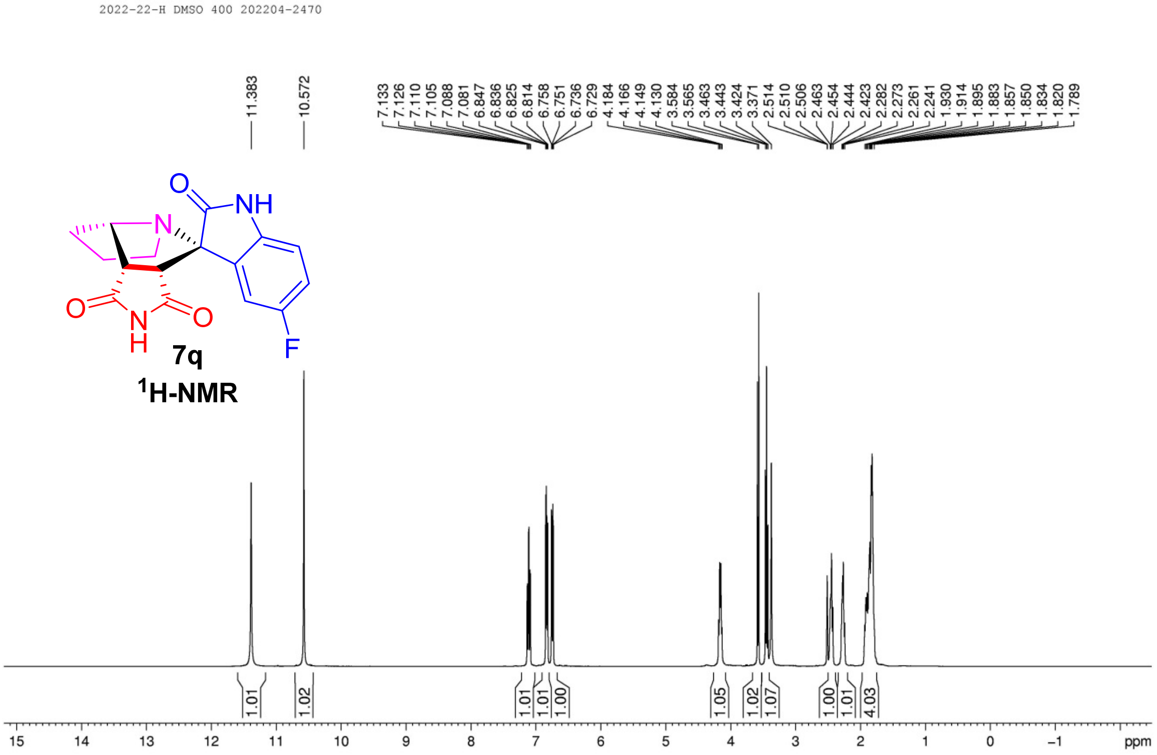
2022-22-H DMSO 400 202204-2470
11.383
10.572
7.133
7.126
7.110
7.105
7.088
7.081
6.847
6.836
6.825
6.814
6.758
6.751
6.736
6.729
4.184
4.166
4.149
4.130
3.584
3.565
3.463
3.443
3.424
3.371
2.514
2.510
2.506
2.463
2.454
2.444
2.423
2.282
2.273
2.261
2.241
1.930
1.914
1.895
1.883
1.857
1.850
1.834
1.820
1.789
O
NH
N
O
N
H
O
F
7q
1H-NMR
1.01
1.02
1.01
1.01
1.00
1.05
1.02
1.07
1.00
1.01
4.03
15
14
13
12
11
10
9
8
7
6
5
4
3
2
1
0
–1
ppm

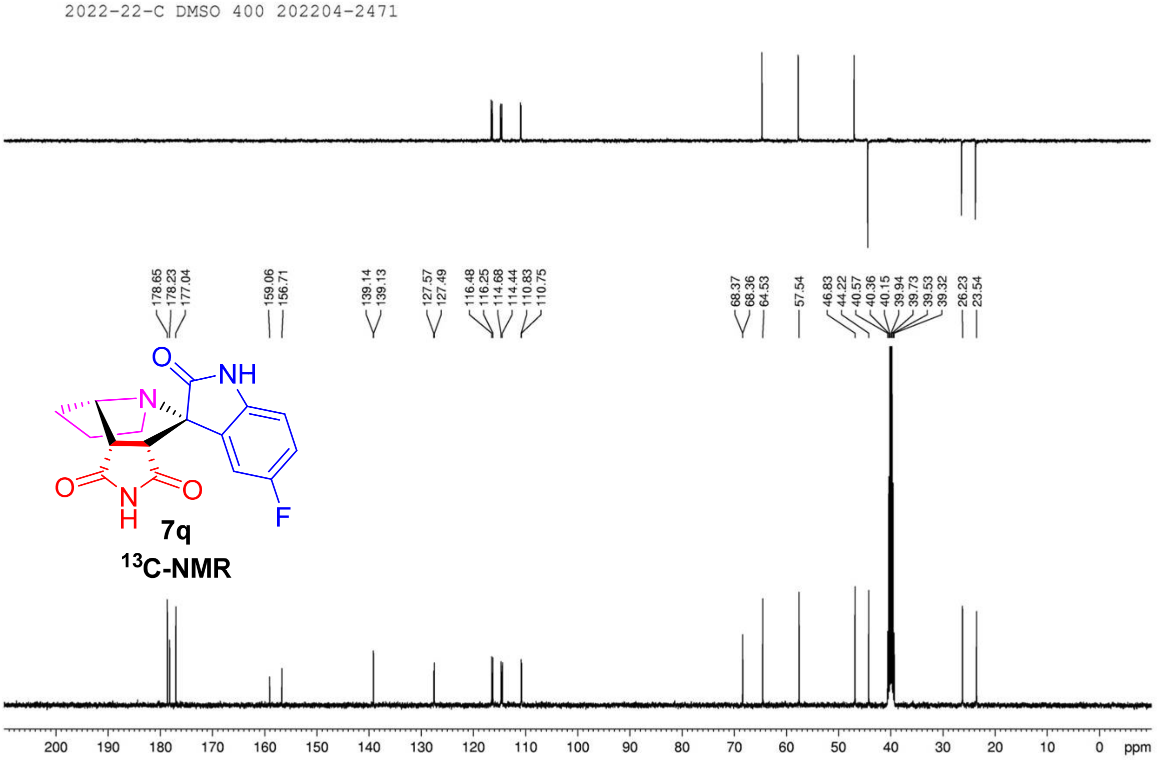
2022-22-C DMSO 400 202204-2471
178.65
178.23
177.04
159.06
156.71
139.14
139.13
127.57
127.49
116.48
116.25
114.68
114.44
110.83
110.75
68.37
68.36
64.53
57.54
46.83
44.22
40.57
40.36
40.15
39.94
39.73
39.53
39.32
26.23
23.54
O
NH
N
O
N
H
O
F
7q
13C-NMR
200
190
180
170
160
150
140
130
120
110
100
90
80
70
60
50
40
30
20
10
0
ppm

## Qualitative Compound Report

| | | | |
|---|---|---|---|
| Data File | 2033368-22-211-NEG.d | Sample Name | 2033368-22-211 |
| Sample Type | Sample | Position | P1-B3 |
| Instrument Name | Instrument 1 | User Name | |
| Acq Method | neg-1min.m | Acquired Time | 2/8/2023 10:23:20 AM |
| IRM Calibration Status | Success | DA Method | QG-903.m |
| Comment | | | |
| Sample Group | | Info. | |
| Stream Name | LC 1 | Acquisition SW Version | 6200 series TOF/6500 series Q-TOF B.08.00 (B8058.0) |

F

**Compound Table**

| Compound Label | RT | Mass | Abund | Formula | Tgt Mass | Diff (ppm) |
|---|---|---|---|---|---|---|
| Cpd 1: C16 H14 F N3 O3 | 0.637 | 315.0911 | 155 | C16 H14 F N3 O3 | 315.0919 | -2.03 |

MS Zoomed Spectrum

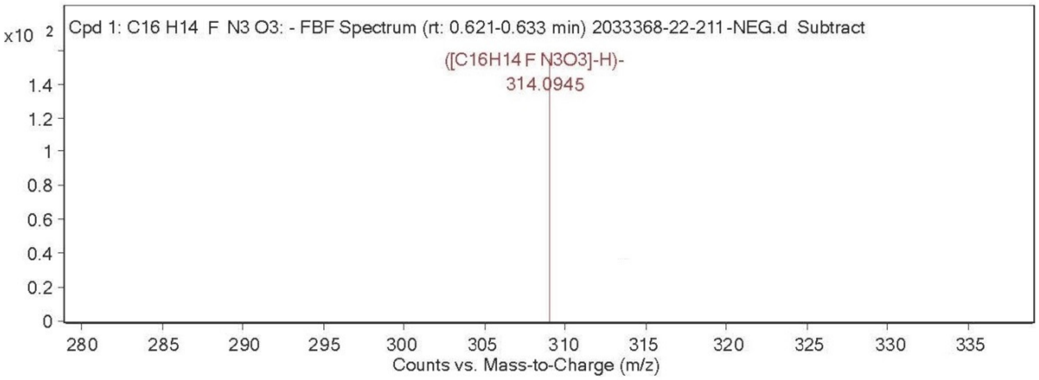


--- End Of Report ---

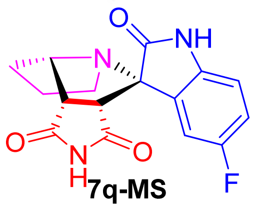


7q-MS

2022-19-H DMSO 400 202204-2560

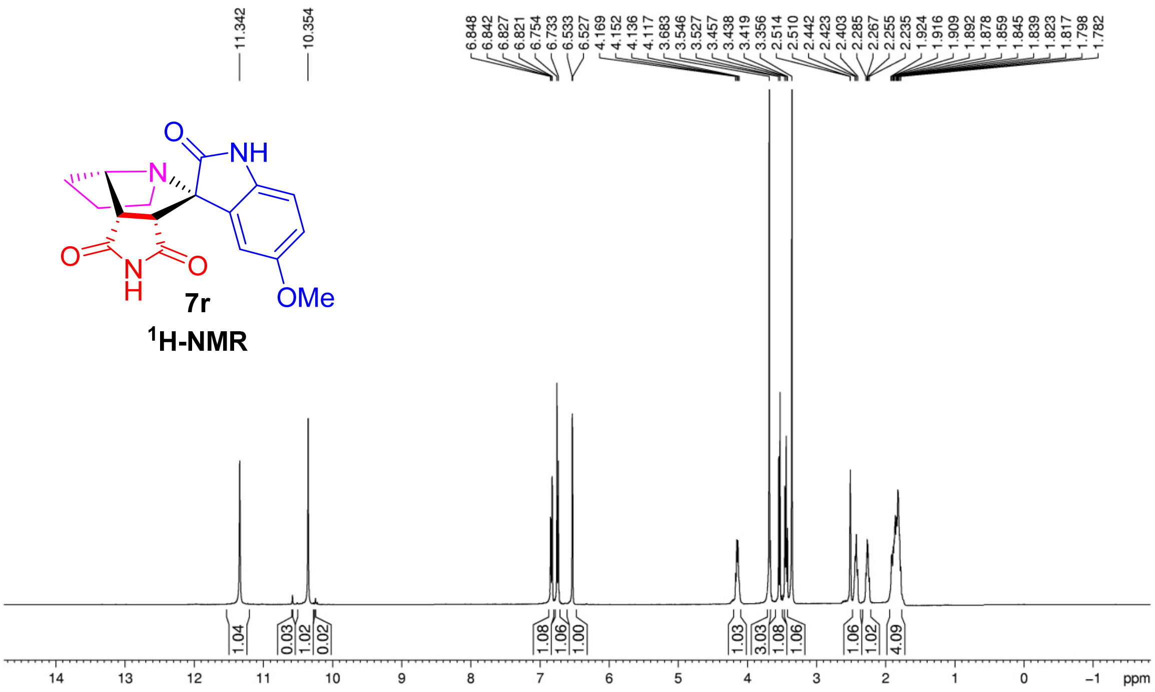


2022-19-C DMSO 400 202204-2561

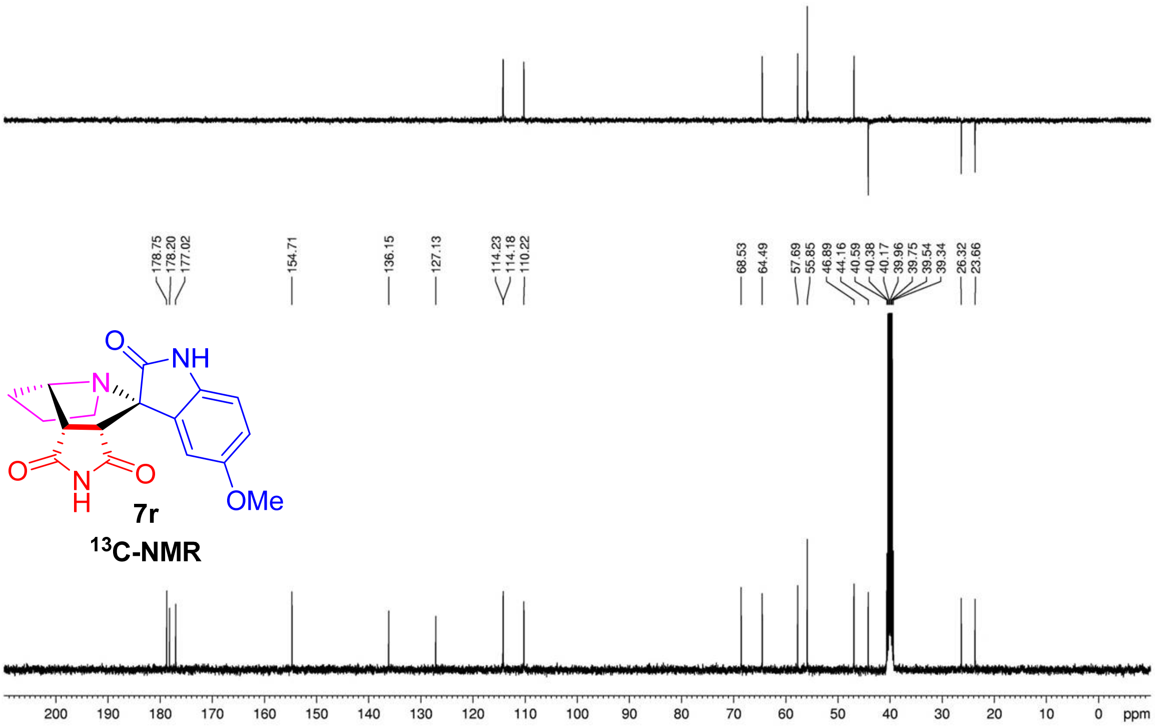

## Qualitative Compound Report

| | | | |
|---|---|---|---|
| Data File | 2033368-22-19-NEG.d | Sample Name | 2033368-22-19 |
| Sample Type | Sample | Position | P1-B2 |
| Instrument Name | Instrument 1 | User Name | |
| Acq Method | neg-1min.m | Acquired Time | 2/8/2023 10:42:25 AM |
| IRM Calibration Status | Success | DA Method | QG-907.m |
| Comment | | | |
| Sample Group | | Info. | |
| Stream Name | LC 1 | Acquisition SW Version | 6200 series TOF/6500 series Q-TOF B.08.00 (B8058.0) |

**Compound Table**

| Compound Label | RT | Mass | Abund | Formula | Tgt Mass | Diff (ppm) |
|---|---|---|---|---|---|---|
| Cpd 1: C17 H17 N3 O4 | 0.314 | 327.122 | 583 | C17 H17 N3 O4 | 327.1219 | 0.16 |

MS Zoomed Spectrum

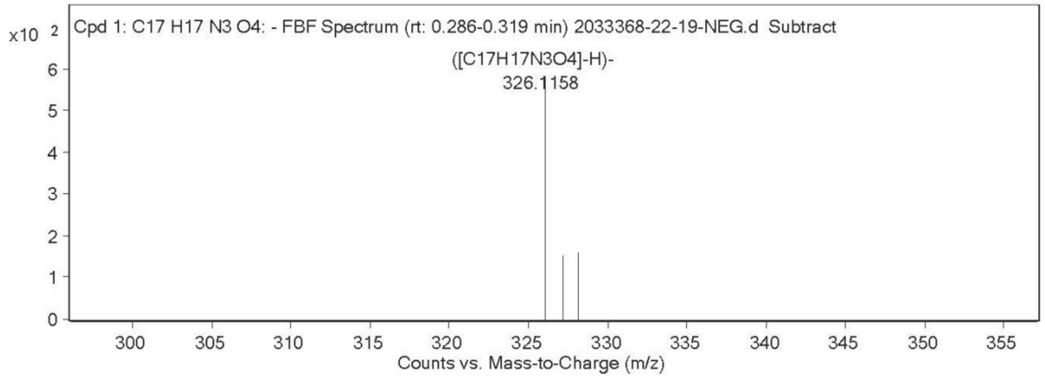


--- End Of Report ---

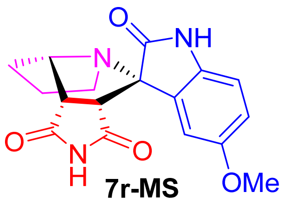


**7r-MS**

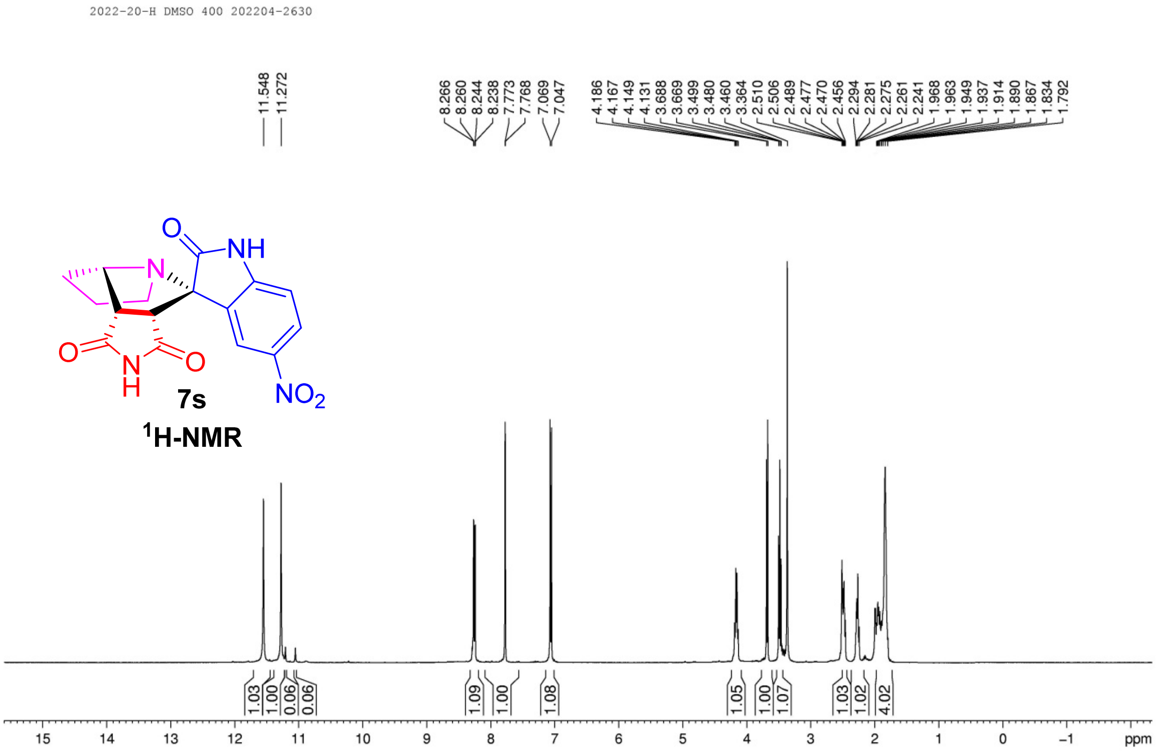
2022-20-H DMSO 400 202204-2630
11.548
11.272
8.266
8.260
8.244
8.238
7.773
7.768
7.069
7.047
4.186
4.167
4.149
4.131
3.688
3.669
3.499
3.480
3.460
3.364
2.510
2.506
2.489
2.477
2.470
2.456
2.294
2.281
2.275
2.261
2.241
1.968
1.963
1.949
1.937
1.914
1.890
1.867
1.834
1.792
O
NH
N
O
N
H
O
NO2
7s
1H-NMR
1.03
1.00
0.06
0.06
1.09
1.00
1.08
1.05
1.00
1.07
1.03
1.02
4.02
ppm

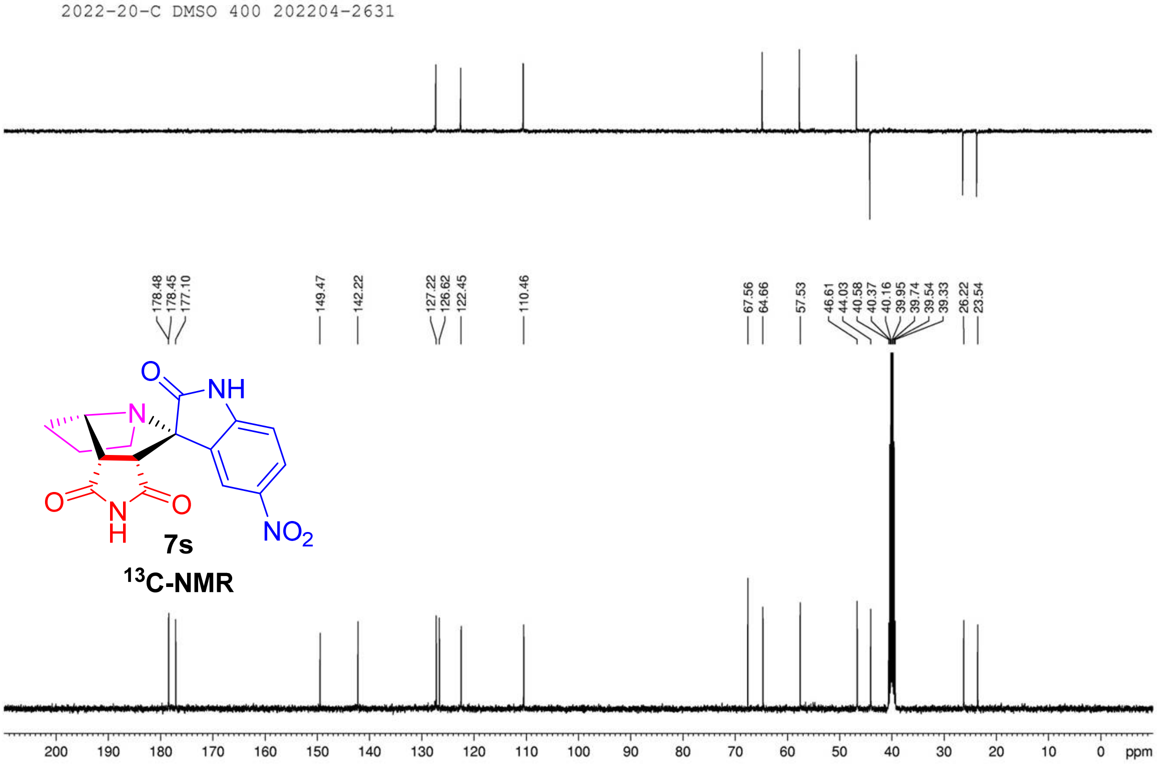
2022-20-C DMSO 400 202204-2631
178.48
178.45
177.10
149.47
142.22
127.22
126.62
122.45
110.46
67.56
64.66
57.53
46.61
44.03
40.58
40.37
40.16
39.95
39.74
39.54
39.33
26.22
23.54
O
NH
N
O
N
H
O
NO2
7s
13C-NMR
ppm

## Qualitative Compound Report

| | | | |
|---|---|---|---|
| Data File | 2033368-22-191-NEG.d | Sample Name | 2033368-22-191 |
| Sample Type | Sample | Position | P1-B3 |
| Instrument Name | Instrument 1 | User Name | |
| Acq Method | neg-1min.m | Acquired Time | 2/8/2023 10:19:23 AM |
| IRM Calibration Status | Success | DA Method | QG-903.m |
| Comment | | | |
| Sample Group | | Info. | |
| Stream Name | LC 1 | Acquisition SW Version | 6200 series TOF/6500 series Q-TOF B.08.00 (B8058.0) |

**Compound Table**

| Compound Label | RT | Mass | Abund | Formula | Tgt Mass | Diff (ppm) |
|---|---|---|---|---|---|---|
| Cpd 1: C16 H14 N4 O5 | 0.327 | 341.1222 | 584 | C16 H14 N4 O5 | 341.1229 | 0.19 |

MS Zoomed Spectrum

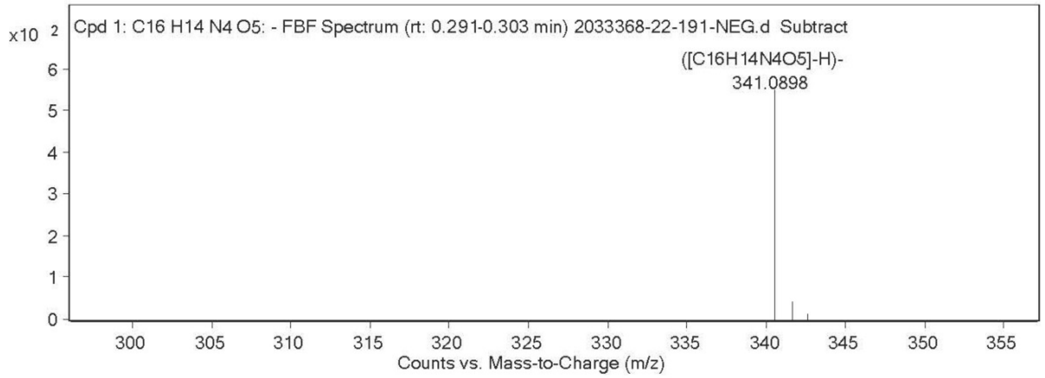


--- End Of Report ---

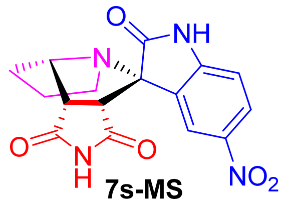


**7s-MS**



Agilent Technologies

Printed at 5:39 PM on 8-Feb-2023

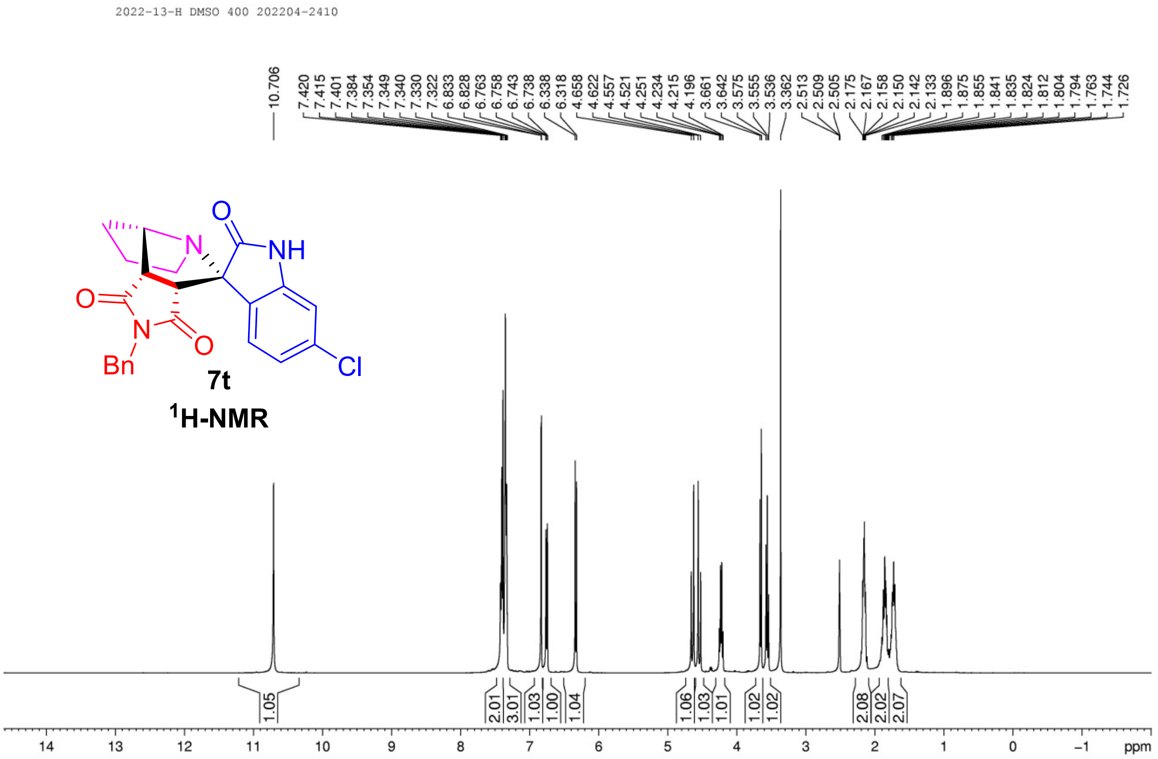
2022-13-H DMSO 400 202204-2410
10.706 7.420 7.415 7.401 7.384 7.354 7.349 7.340 7.330 7.322 6.833 6.828 6.763 6.758 6.743 6.738 6.338 6.318 4.658 4.622 4.557 4.521 4.251 4.234 4.215 4.196 3.661 3.642 3.575 3.555 3.536 3.362 2.513 2.509 2.505 2.175 2.167 2.158 2.150 2.142 2.133 1.896 1.875 1.855 1.841 1.835 1.824 1.812 1.804 1.794 1.763 1.744 1.726
O
N
NH
O
N
O
Bn
Cl
7t
1H-NMR
1.05 2.01 3.01 1.03 1.00 1.04 1.06 1.03 1.01 1.02 1.02 2.08 2.02 2.07
14 13 12 11 10 9 8 7 6 5 4 3 2 1 0 −1 ppm

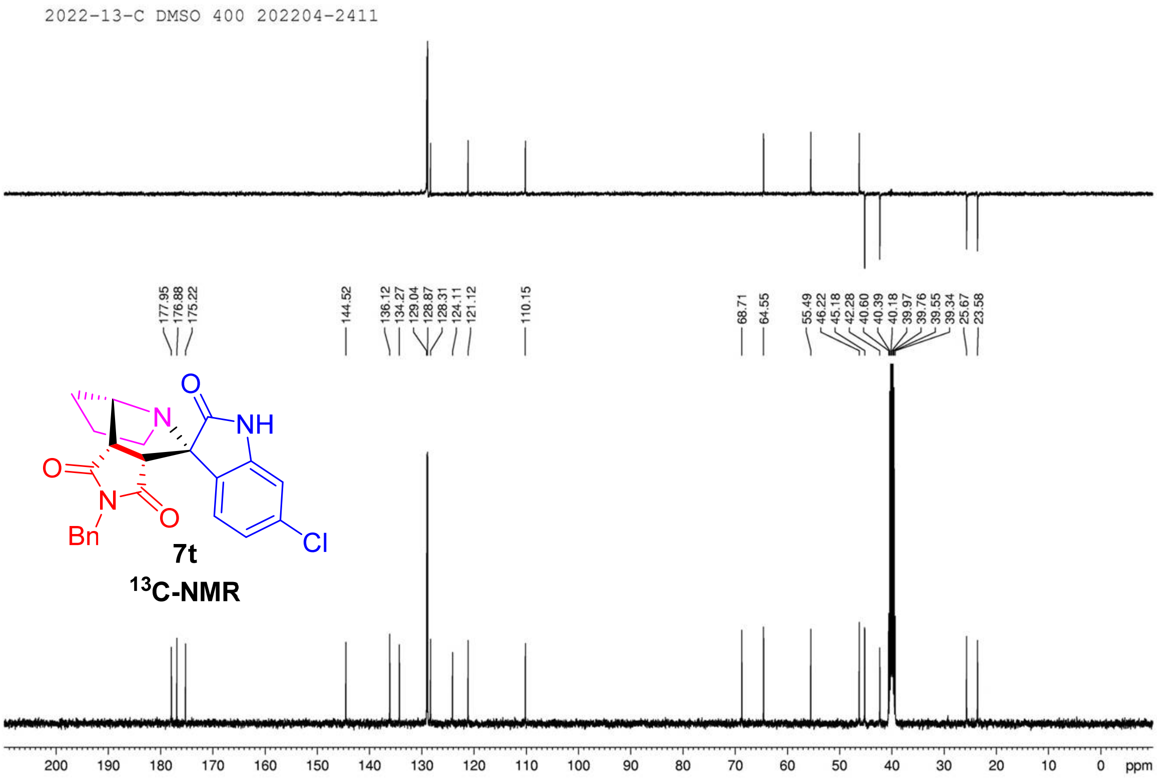
2022-13-C DMSO 400 202204-2411
177.95 176.88 175.22 144.52 136.12 134.27 129.04 128.87 128.31 124.11 121.12 110.15 68.71 64.55 55.49 46.22 45.18 42.28 40.60 40.39 40.18 39.97 39.76 39.55 39.34 25.67 23.58
O
N
NH
O
N
O
Bn
Cl
7t
13C-NMR
200 190 180 170 160 150 140 130 120 110 100 90 80 70 60 50 40 30 20 10 0 ppm

## Qualitative Compound Report

| | | | |
|---|---|---|---|
| Data File | 2033368-22-13-NEG.d | Sample Name | 2033368-22-13 |
| Sample Type | Sample | Position | P1-A8 |
| Instrument Name | Instrument 1 | User Name | |
| Acq Method | neg-1min.m | Acquired Time | 2/8/2023 10:36:46 AM |
| IRM Calibration Status | Success | DA Method | QG-907.m |
| Comment | | | |
| Sample Group | | Info. | |
| Stream Name | LC 1 | Acquisition SW Version | 6200 series TOF/6500 series Q-TOF B.08.00 (B8058.0) |

**Compound Table**

| Compound Label | RT | Mass | Abund | Formula | Tgt Mass | Diff (ppm) |
|---|---|---|---|---|---|---|
| Cpd 1: C23 H20 Cl N3 O3 | 0.294 | 421.1198 | 99605 | C23 H20 Cl N3 O3 | 421.1193 | 1.08 |

MS Zoomed Spectrum

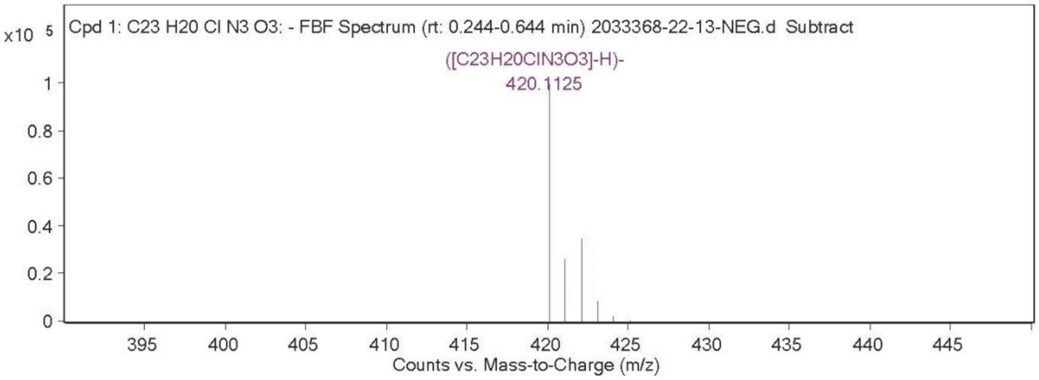


--- End Of Report ---

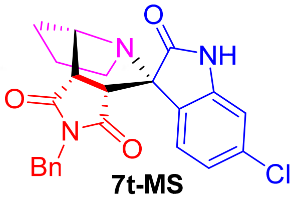


**7t-MS**

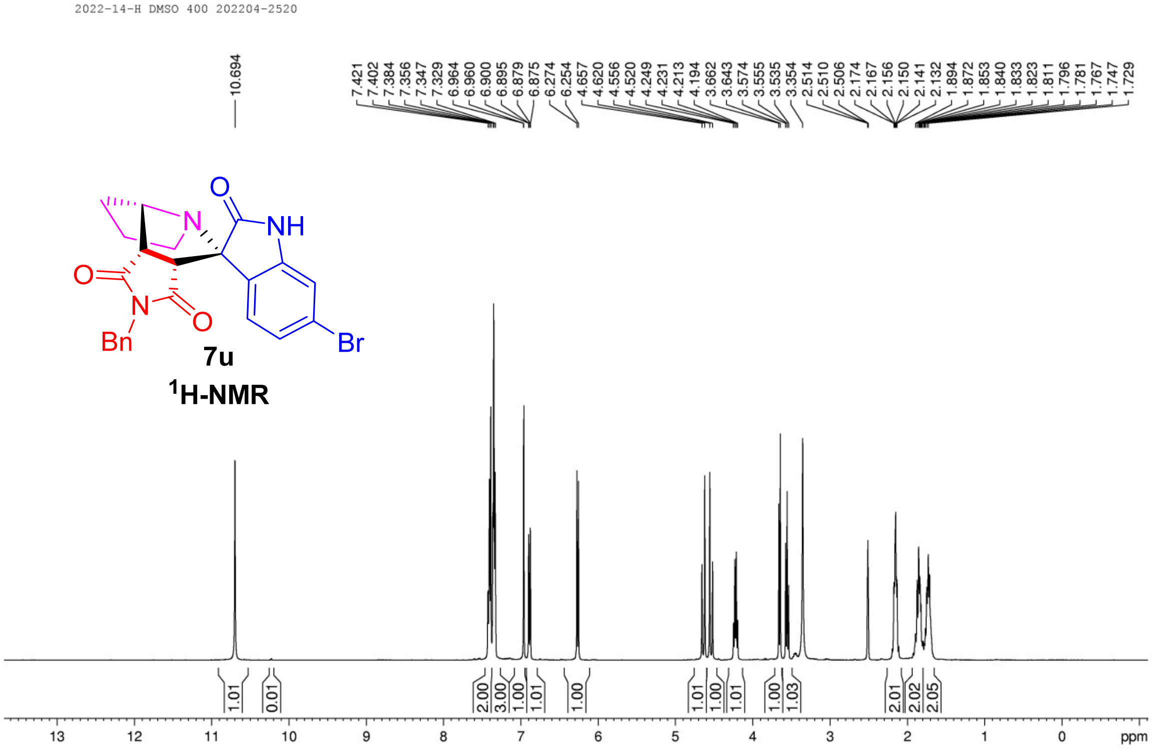
2022-14-H DMSO 400 202204-2520
7u
1H-NMR
Br
NH
Bn

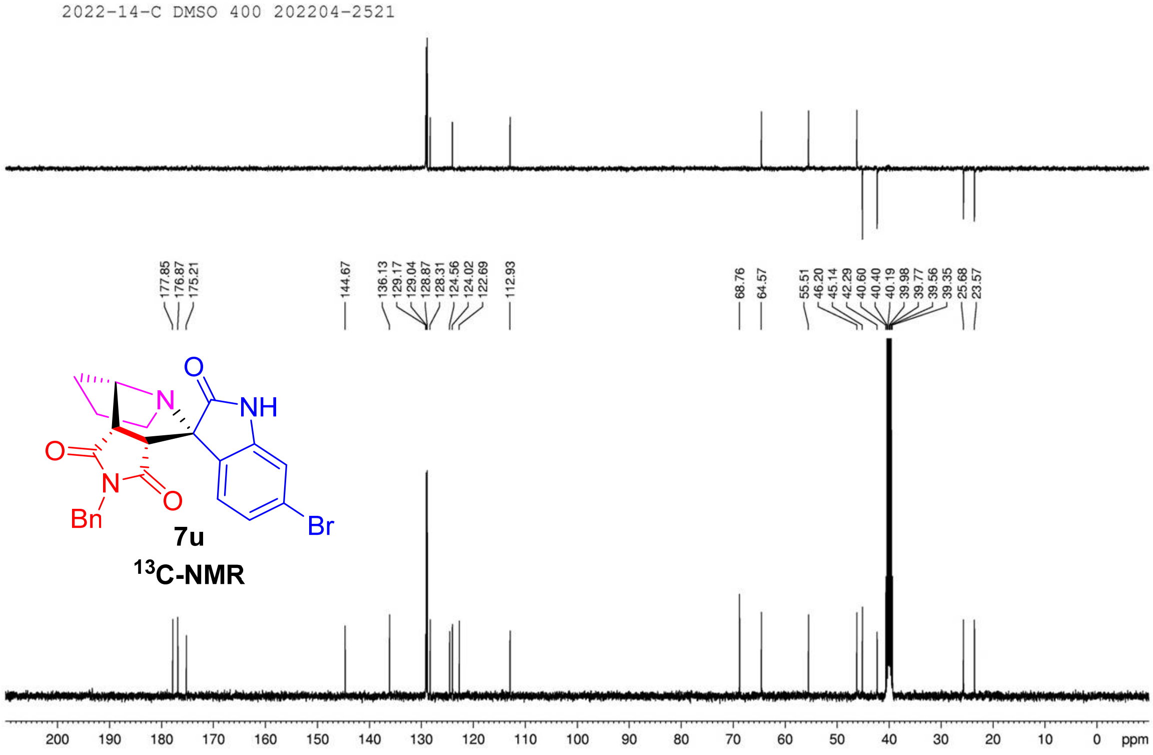
2022-14-C DMSO 400 202204-2521
7u
13C-NMR
Br
NH
Bn

## Qualitative Compound Report

| | | | |
|---|---|---|---|
| Data File | 2033368-22-33.d | Sample Name | 2033368-22-33 |
| Sample Type | Sample | Position | P1-B3 |
| Instrument Name | Instrument 1 | User Name | |
| Acq Method | pos-1min.m | Acquired Time | 2/6/2023 5:29:36 PM |
| IRM Calibration Status | Success | DA Method | QG-905.m |
| Comment | | | |
| Sample Group | | Info. | |
| Stream Name | LC 1 | Acquisition SW Version | 6200 series TOF/6500 series Q-TOF B.08.00 (B8058.0) |

**Compound Table**

| Compound Label | RT | Mass | Abund | Formula | Tgt Mass | Diff (ppm) |
|---|---|---|---|---|---|---|
| Cpd 1: C23 H20 Br N3 O3 | 0.127 | 466.0866 | 194434 | C23 H20 Br N3 O3 | 466.0662 | 2.26 |

MS Zoomed Spectrum

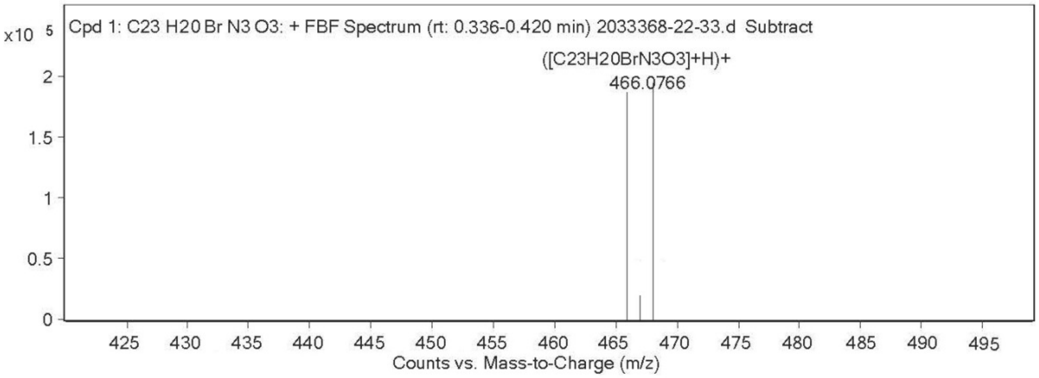


--- End Of Report ---

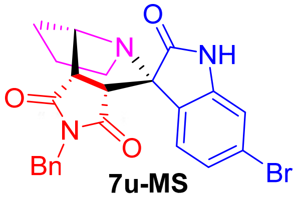


**7u-MS**

2022-15-H DMSO 400 202204-2460

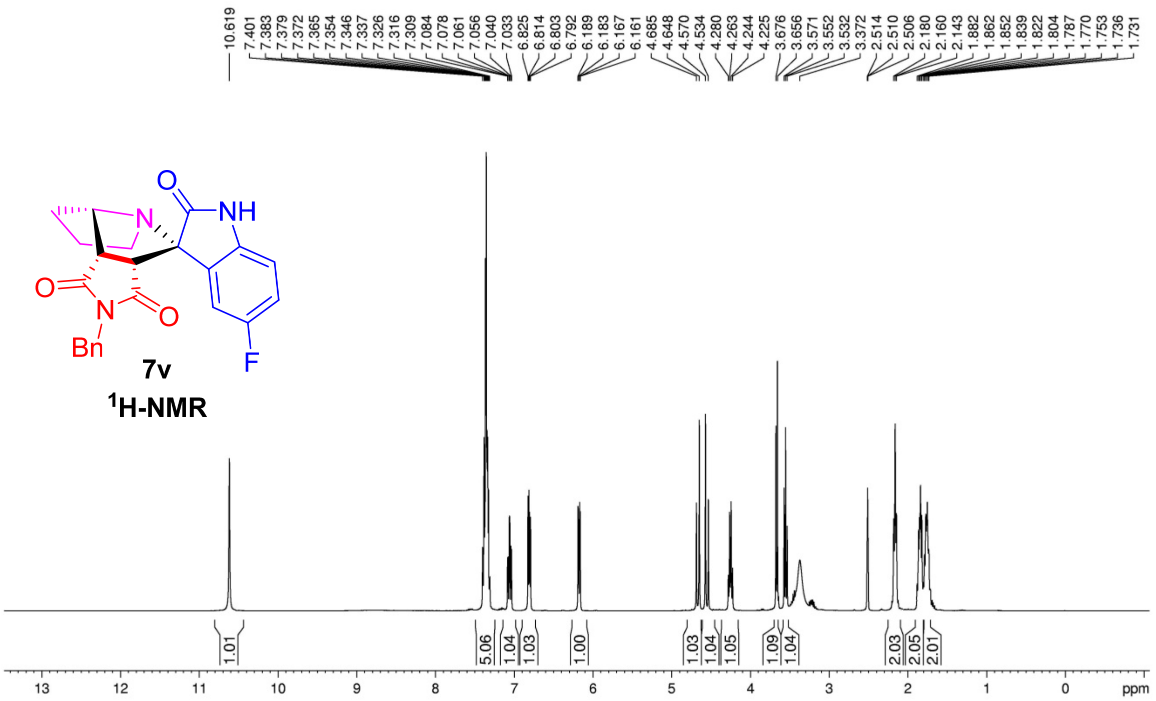


2022-15-C DMSO 400 202204-2461

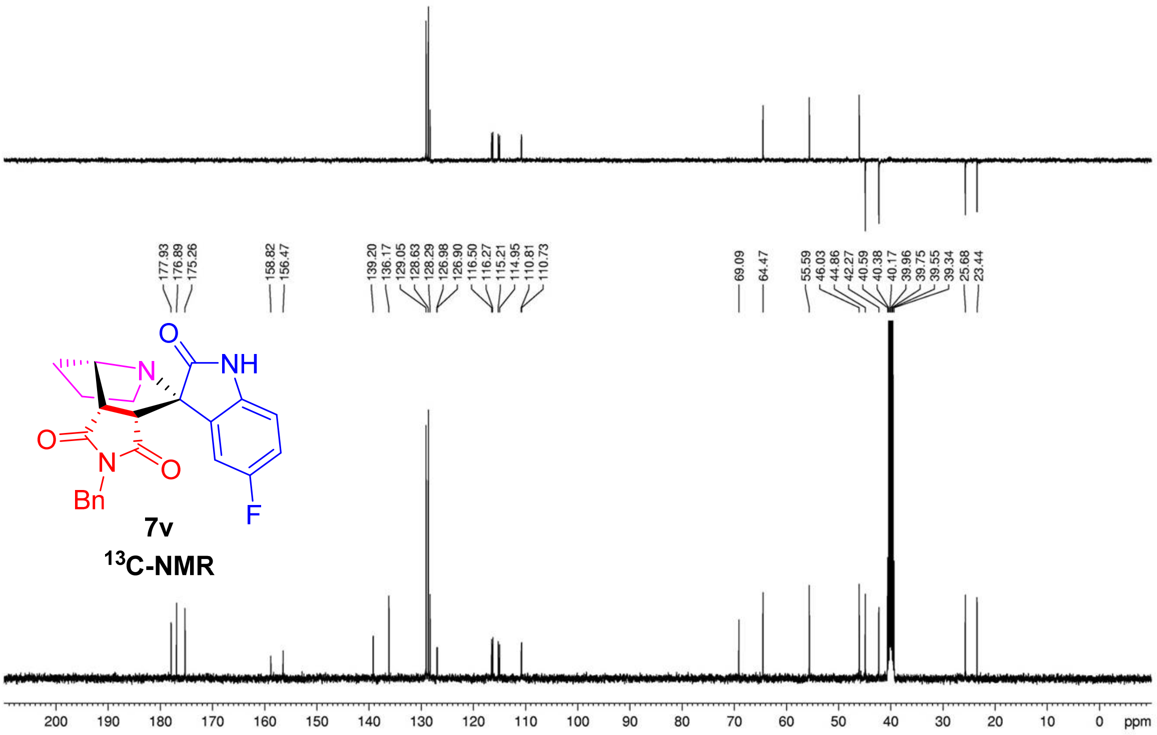

## Qualitative Compound Report

| | | | |
|---|---|---|---|
| Data File | 2033368-22-64.d | Sample Name | 2033368-22-64 |
| Sample Type | Sample | Position | P1-C6 |
| Instrument Name | Instrument 1 | User Name | |
| Acq Method | pos-1min.m | Acquired Time | 2/6/2023 5:47:20 PM |
| IRM Calibration Status | Success | DA Method | QG-906.m |
| Comment | | | |
| Sample Group | | Info. | |
| Stream Name | LC 1 | Acquisition SW Version | 6200 series TOF/6500 series Q-TOF B.08.00 (B8058.0) |

**Compound Table**

| Compound Label | RT | Mass | Abund | Formula | Tgt Mass | Diff (ppm) |
|---|---|---|---|---|---|---|
| Cpd 1: C23H20 F N3 O3 | 0.185 | 406.1579 | 85271 | C23H20 F N3 O3 | 406.1571 | 2.26 |

MS Zoomed Spectrum

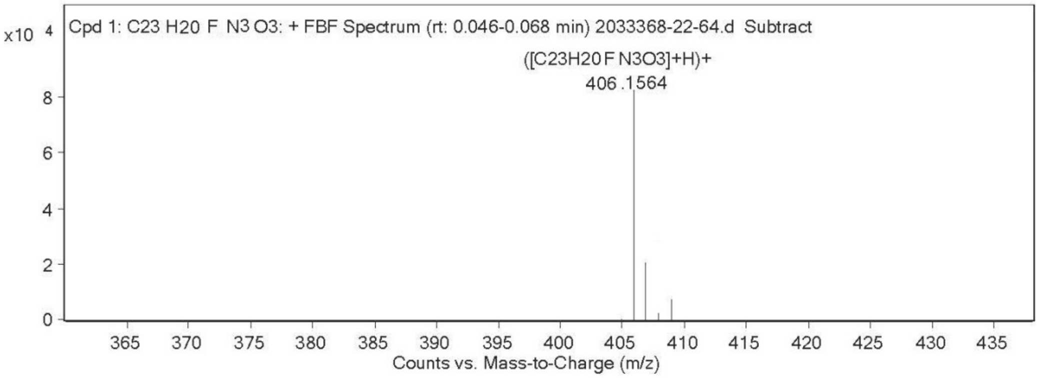


--- End Of Report ---

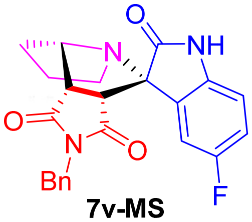


**7v-MS**

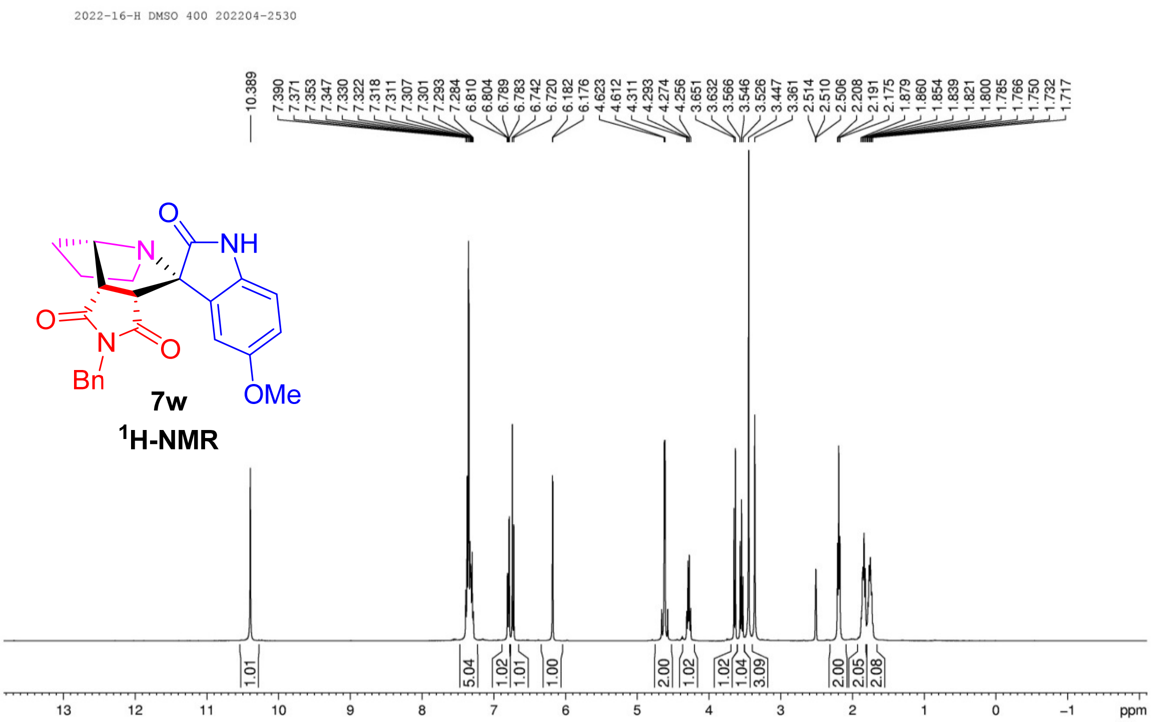
2022-16-H DMSO 400 202204-2530
10.389
7.390
7.371
7.353
7.347
7.330
7.322
7.318
7.311
7.307
7.301
7.293
7.284
6.810
6.804
6.789
6.783
6.742
6.720
6.182
6.176
4.623
4.612
4.311
4.293
4.274
4.256
3.651
3.632
3.566
3.546
3.526
3.447
3.361
2.514
2.510
2.506
2.208
2.191
2.175
1.879
1.860
1.854
1.839
1.821
1.800
1.785
1.766
1.750
1.732
1.717
O
NH
N
O
N
O
Bn
OMe
7w
1H-NMR
1.01
5.04
1.02
1.01
1.00
2.00
1.02
1.02
1.04
3.09
2.00
2.05
2.08
ppm

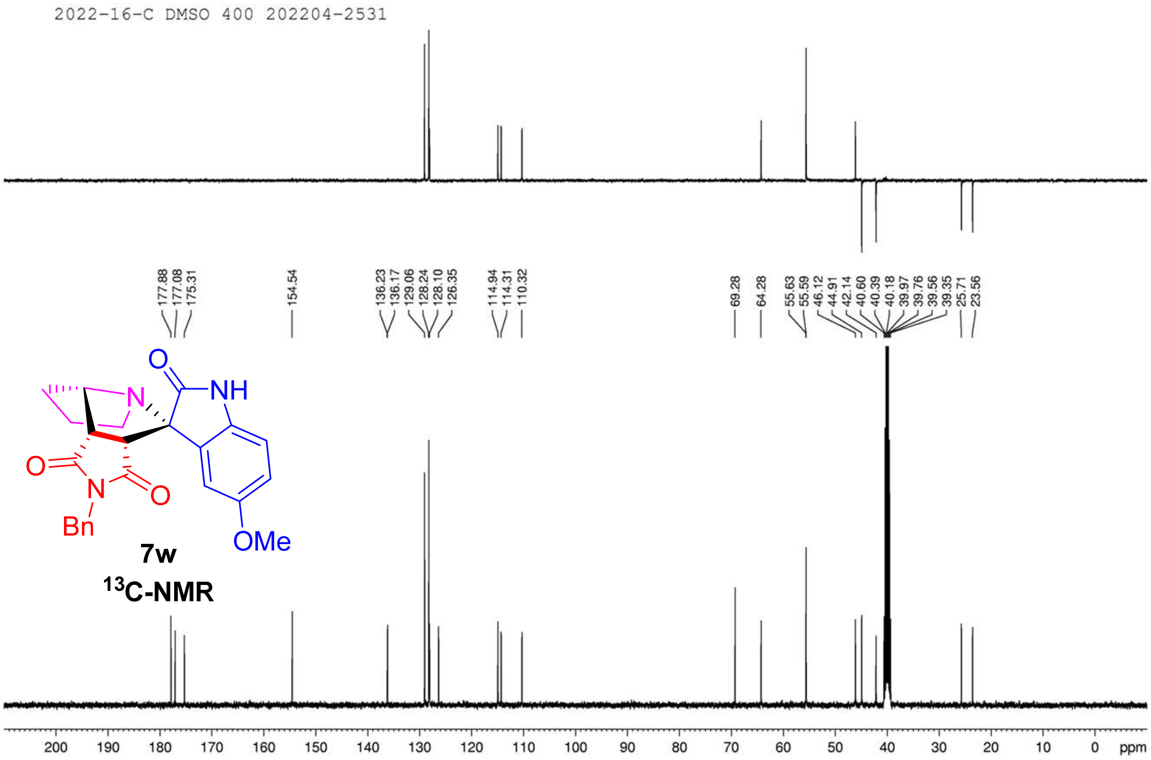
2022-16-C DMSO 400 202204-2531
177.88
177.08
175.31
154.54
136.23
136.17
129.06
128.24
128.10
126.35
114.94
114.31
110.32
69.28
64.28
55.63
55.59
46.12
44.91
42.14
40.60
40.39
40.18
39.97
39.76
39.56
39.35
25.71
23.56
O
NH
N
O
N
O
Bn
OMe
7w
13C-NMR
ppm

## Qualitative Compound Report

| | | | |
|---|---|---|---|
| Data File | 2033368-22-66.d | Sample Name | 2033368-22-66 |
| Sample Type | Sample | Position | P1-C3 |
| Instrument Name | Instrument 1 | User Name | |
| Acq Method | pos-1min.m | Acquired Time | 2/6/2023 5:47:33 PM |
| IRM Calibration Status | Success | DA Method | QG-920.m |
| Comment | | | |
| Sample Group | | Info. | |
| Stream Name | LC 1 | Acquisition SW Version | 6200 series TOF/6500 series Q-TOF B.08.00 (B8058.0) |

**Compound Table**

| Compound Label | RT | Mass | Abund | Formula | Tgt Mass | Diff (ppm) |
|---|---|---|---|---|---|---|
| Cpd 1: C24H23 N3 O4 | 0.123 | 417.1515 | 85241 | C24H23 N3 O4 | 417.1571 | 2.24 |

MS Zoomed Spectrum

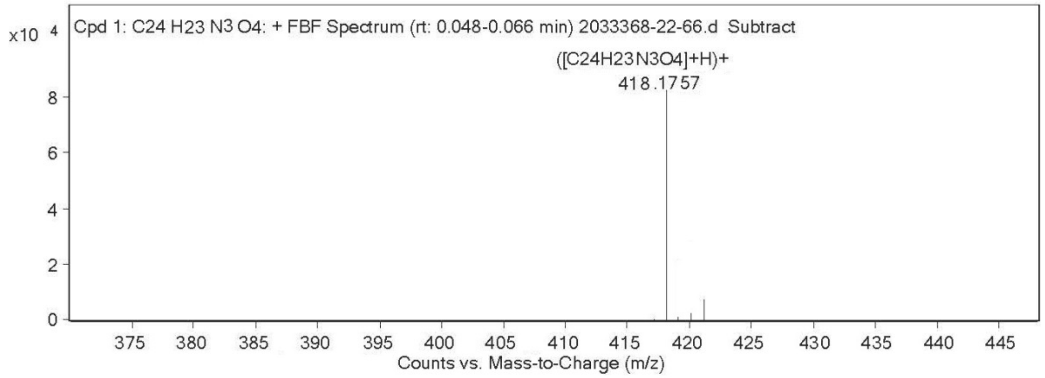


--- End Of Report ---

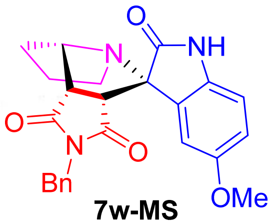


**7w-MS**

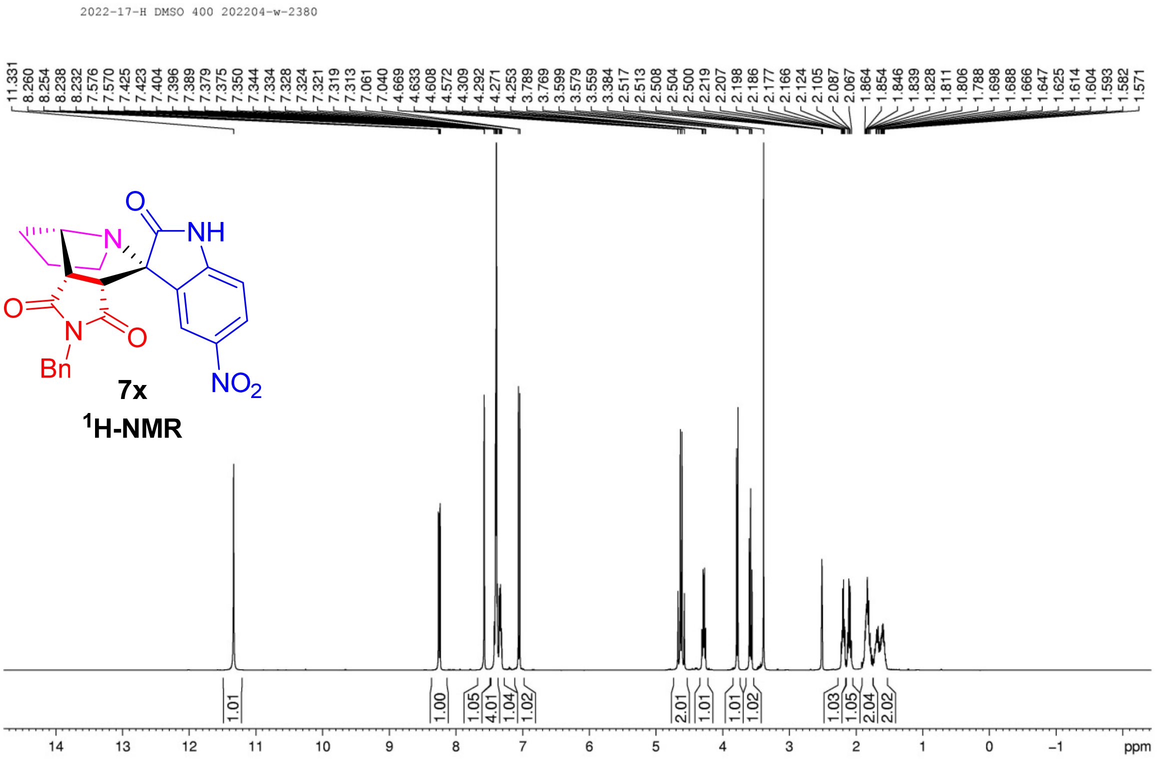
2022-17-H DMSO 400 202204-w-2380
11.331 8.260 8.254 8.238 8.232 7.576 7.570 7.425 7.423 7.404 7.396 7.389 7.379 7.375 7.350 7.344 7.334 7.328 7.324 7.321 7.319 7.313 7.061 7.040 4.669 4.633 4.608 4.572 4.309 4.292 4.271 4.253 3.789 3.769 3.599 3.579 3.559 3.384 2.517 2.513 2.508 2.504 2.500 2.219 2.207 2.198 2.186 2.177 2.166 2.124 2.105 2.087 2.067 1.864 1.854 1.846 1.839 1.828 1.811 1.806 1.788 1.698 1.688 1.666 1.647 1.625 1.614 1.604 1.593 1.582 1.571
O
NH
N
O
N
O
Bn
NO2
7x
1H-NMR
1.01 1.00 1.05 4.01 1.04 1.02 2.01 1.01 1.01 1.02 1.03 1.05 2.04 2.02
14 13 12 11 10 9 8 7 6 5 4 3 2 1 0 –1 ppm

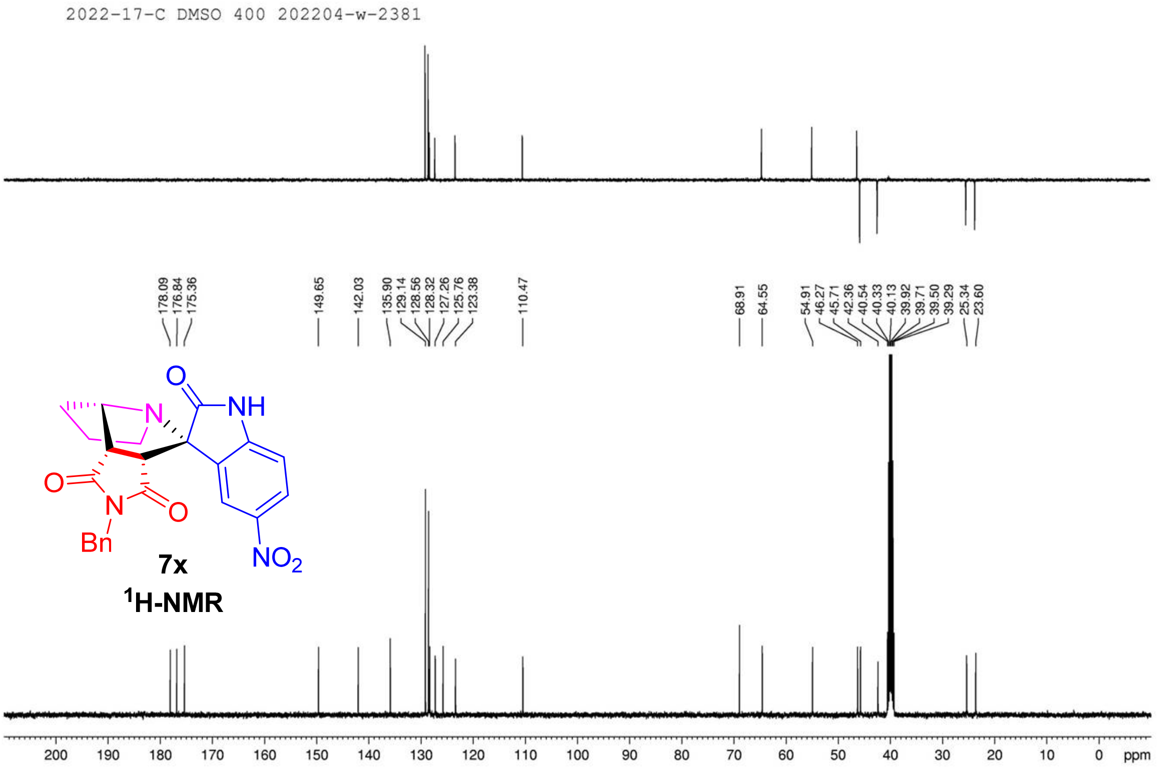
2022-17-C DMSO 400 202204-w-2381
178.09 176.84 175.36 149.65 142.03 135.90 129.14 128.56 128.32 127.26 125.76 123.38 110.47 68.91 64.55 54.91 46.27 45.71 42.36 40.54 40.33 40.13 39.92 39.71 39.50 39.29 25.34 23.60
O
NH
N
O
N
O
Bn
NO2
7x
1H-NMR
200 190 180 170 160 150 140 130 120 110 100 90 80 70 60 50 40 30 20 10 0 ppm

## Qualitative Compound Report

| | | | |
|---|---|---|---|
| Data File | 2033368-22-17-NEG.d | Sample Name | 2033368-22-17 |
| Sample Type | Sample | Position | P1-B1 |
| Instrument Name | Instrument 1 | User Name | |
| Acq Method | neg-1min.m | Acquired Time | 2/8/2023 10:40:33 AM |
| IRM Calibration Status | Success | DA Method | QG-907.m |
| Comment | | | |
| Sample Group | | Info. | |
| Stream Name | LC 1 | Acquisition SW Version | 6200 series TOF/6500 series Q-TOF B.08.00 (B8058.0) |

**Compound Table**

| Compound Label | RT | Mass | Abund | Formula | Tgt Mass | Diff (ppm) |
|---|---|---|---|---|---|---|
| Cpd 1: C23 H20 N4 O5 | 0.106 | 432.1451 | 57311 | C23 H20 N4 O5 | 432.1434 | 3.93 |

MS Zoomed Spectrum

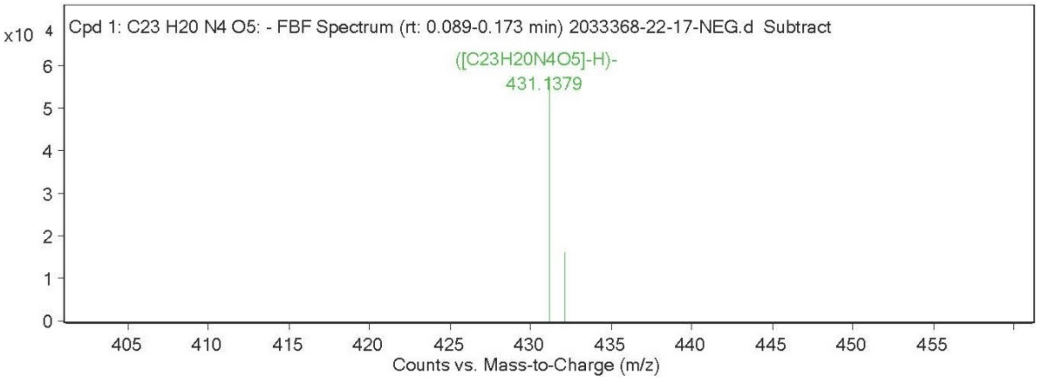


--- End Of Report ---

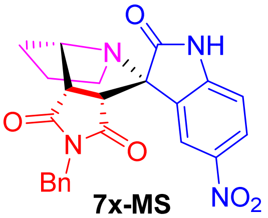


**7x-MS**

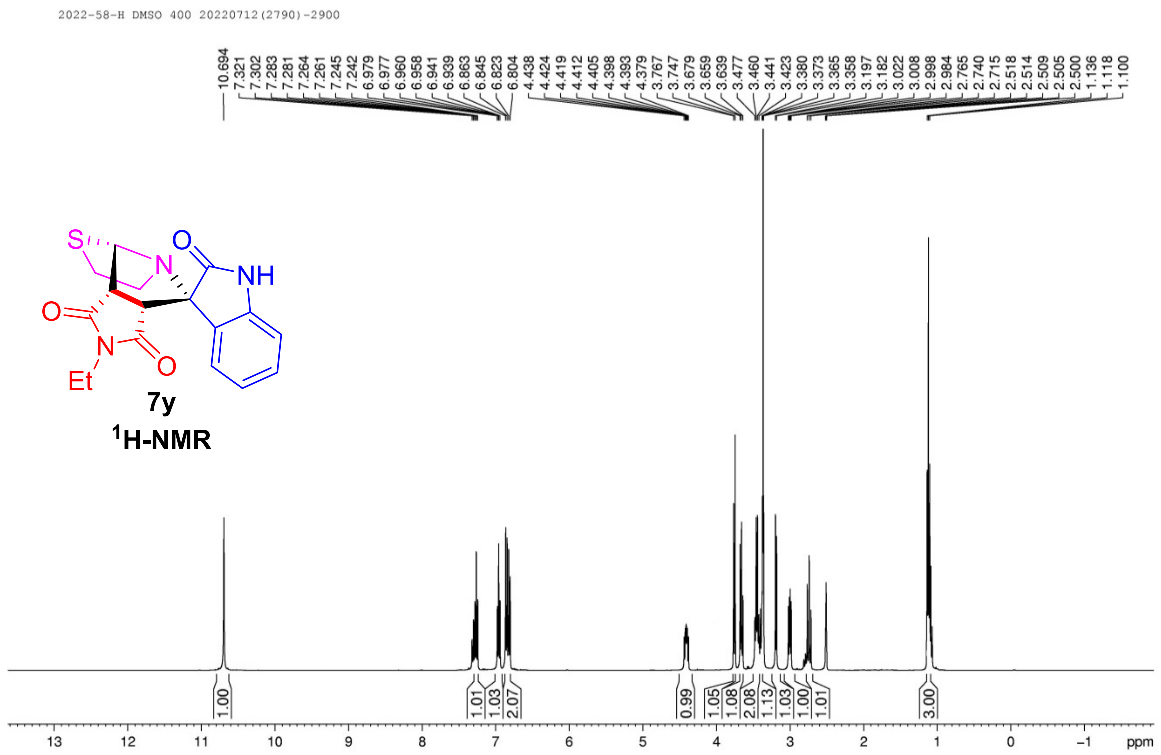
2022-58-H DMSO 400 20220712(2790)-2900
10.694 7.321 7.302 7.283 7.281 7.264 7.261 7.245 7.242 6.979 6.977 6.960 6.958 6.941 6.939 6.863 6.845 6.823 6.804 4.438 4.424 4.419 4.412 4.405 4.398 4.393 4.379 3.767 3.747 3.679 3.659 3.639 3.477 3.460 3.441 3.423 3.380 3.373 3.365 3.358 3.197 3.182 3.022 3.008 2.998 2.984 2.765 2.740 2.715 2.518 2.514 2.509 2.505 2.500 1.136 1.118 1.100
S
N
O
NH
O
N
O
Et
7y
1H-NMR
1.00 1.01 1.03 2.07 0.99 1.05 1.08 2.08 1.13 1.03 1.00 1.01 3.00
13 12 11 10 9 8 7 6 5 4 3 2 1 0 –1 ppm

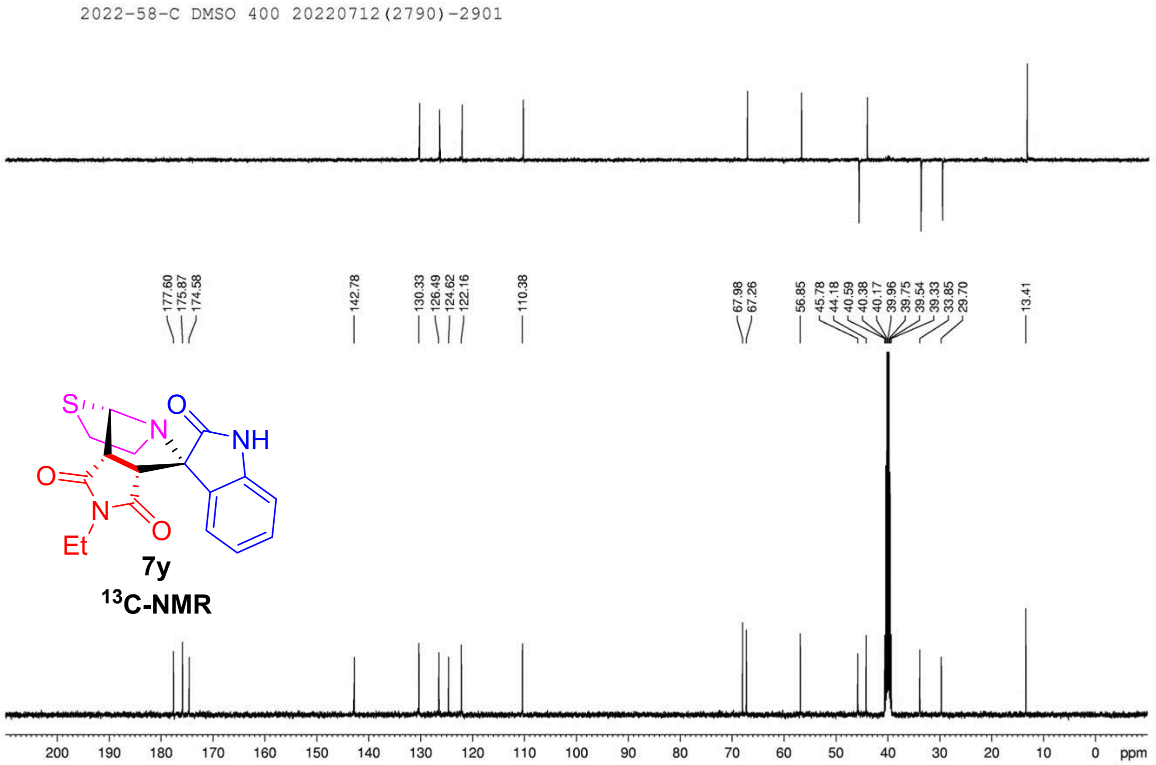
2022-58-C DMSO 400 20220712(2790)-2901
177.60 175.87 174.58 142.78 130.33 126.49 124.62 122.16 110.38 67.98 67.26 56.85 45.78 44.18 40.59 40.38 40.17 39.96 39.75 39.54 39.33 33.85 29.70 13.41
S
N
O
NH
O
N
O
Et
7y
13C-NMR
200 190 180 170 160 150 140 130 120 110 100 90 80 70 60 50 40 30 20 10 0 ppm

## Qualitative Compound Report

| | | | |
|---|---|---|---|
| Data File | 2033368-22-57.d | Sample Name | 2033368-22-57 |
| Sample Type | Sample | Position | P1-C9 |
| Instrument Name | Instrument 1 | User Name | |
| Acq Method | pos-1min.m | Acquired Time | 2/6/2023 5:51:39 PM |
| IRM Calibration Status | Success | DA Method | QG-907.m |
| Comment | | | |
| Sample Group | | Info. | |
| Stream Name | LC 1 | Acquisition SW Version | 6200 series TOF/6500 series Q-TOF B.08.00 (B8058.0) |

**Compound Table**

| Compound Label | RT | Mass | Abund | Formula | Tgt Mass | Diff (ppm) |
|---|---|---|---|---|---|---|
| Cpd 1: C17 H17 N3 O3 S | 0.129 | 343.0997 | 386048 | C17 H17 N3 O3 S | 343.0991 | 1.99 |

MS Zoomed Spectrum

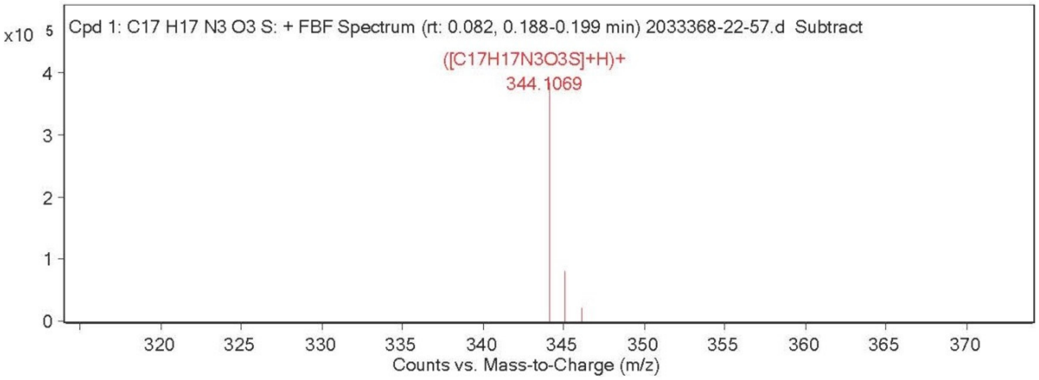


--- End Of Report ---

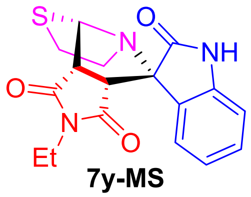


**7y-MS**

## 2. $^{1}$H and $^{13}$C NMR spectra for compounds 8

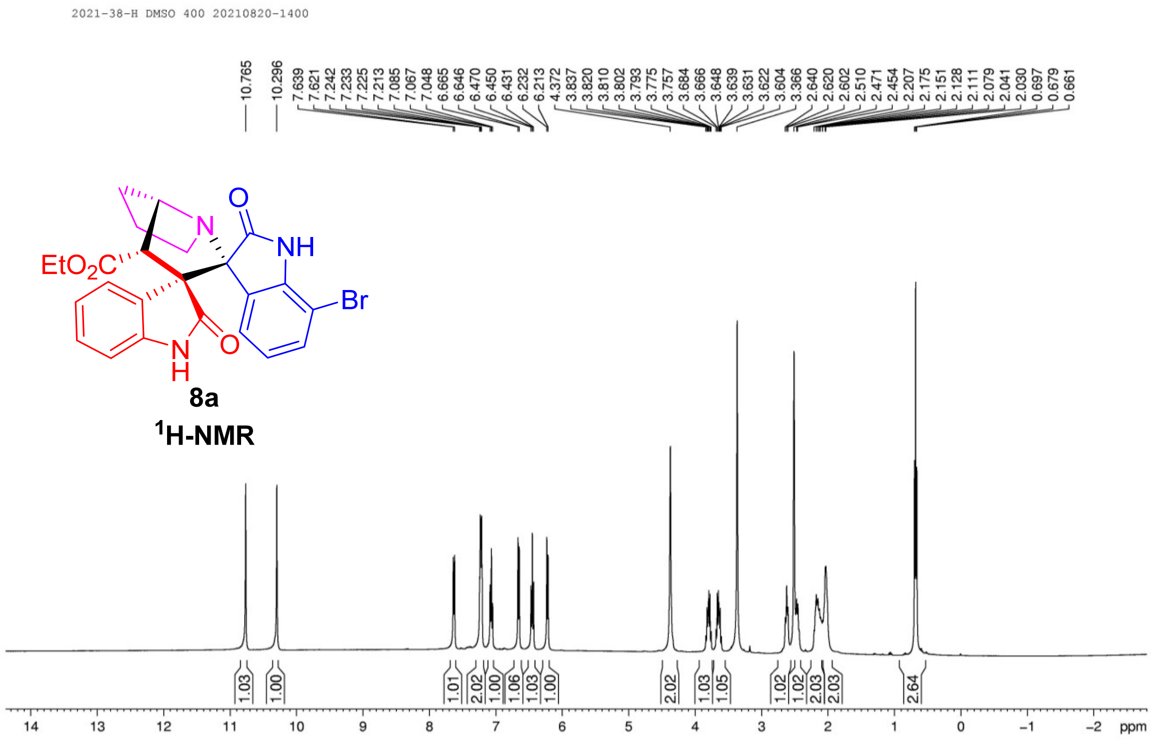


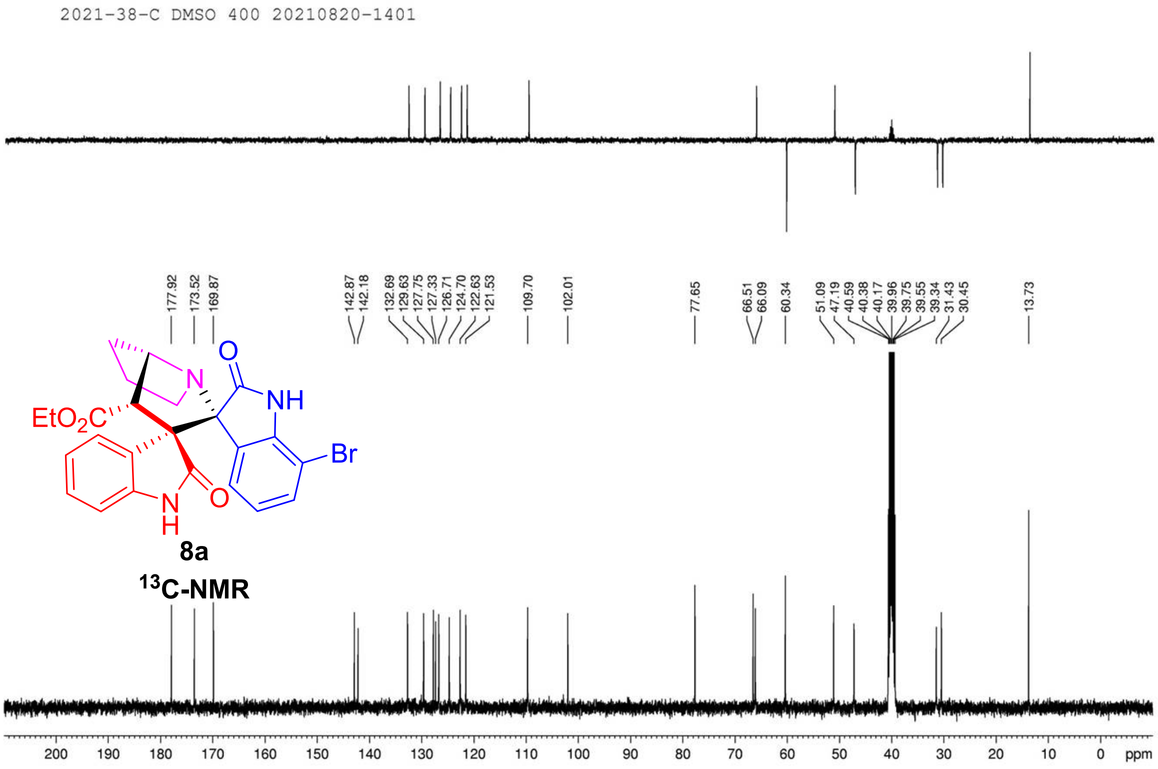

# Qualitative Compound Report

| | | | |
|---|---|---|---|
| Data File | 8036980-22-19.d | Sample Name | 8036980-22-19 |
| Sample Type | Sample | Position | P1-D2 |
| Instrument Name | Instrument 1 | User Name | |
| Acq Method | pos-1min.m | Acquired Time | 8/8/2022 5:40:19 PM |
| IRM Calibration Status | Success | DA Method | default.m |
| Comment | | | |
| Sample Group | | Info. | |
| Stream Name | LC 1 | Acquisition SW Version | 6200 series TOF/6500 series Q-TOF B.08.00 (B8058.0) |

**Compound Table**

| Compound Label | RT | Mass | Abund | Formula | Tgt Mass | Diff (ppm) |
|---|---|---|---|---|---|---|
| Cpd 1: C24H22 Br N3 O4 | 0.203 | 495.0627 | 273827 | C24H22 Br N3 O4 | 495.0632 | 3.03 |

MS Zoomed Spectrum

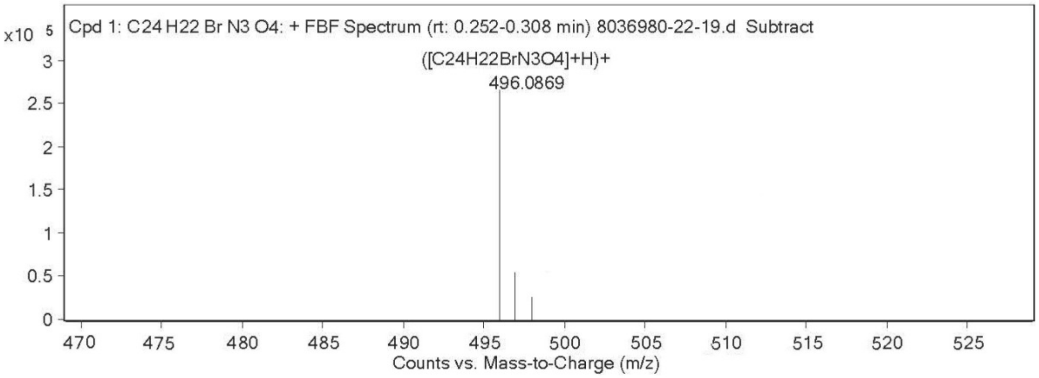


--- End Of Report ---

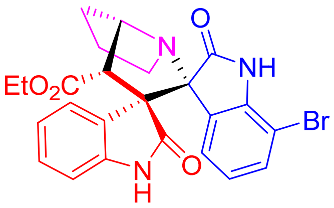


**8a-MS**

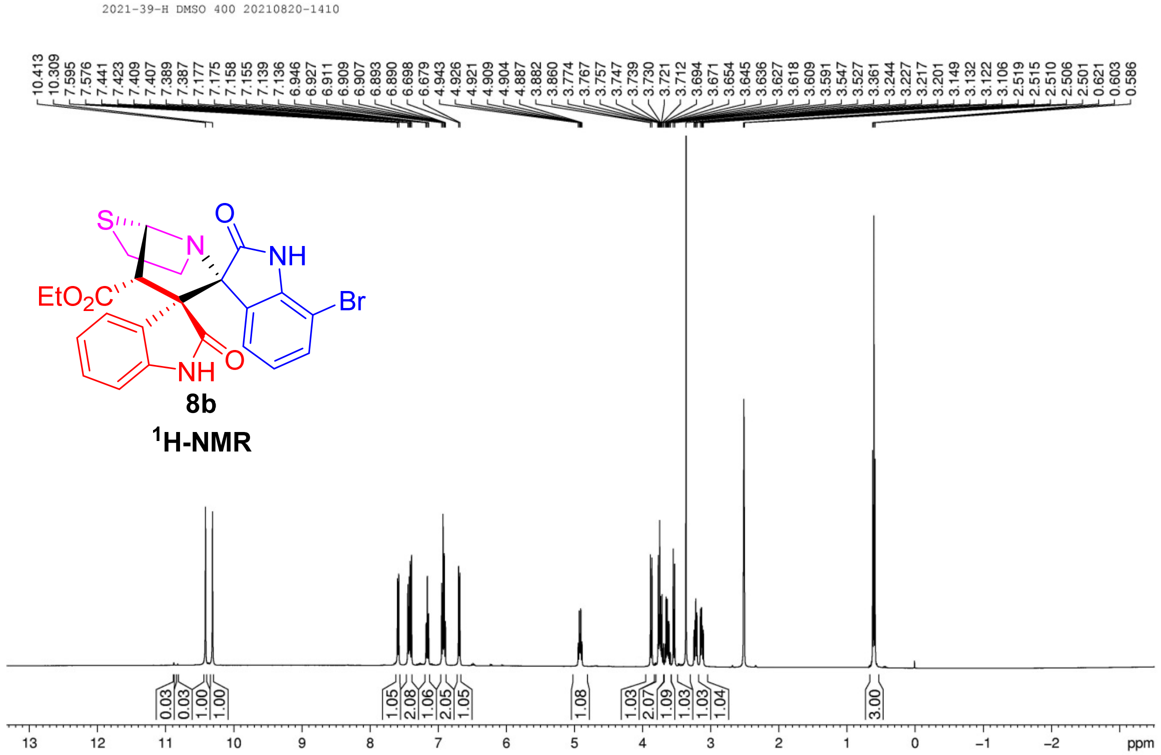
2021-39-H DMSO 400 20210820-1410
8b
1H-NMR
ppm

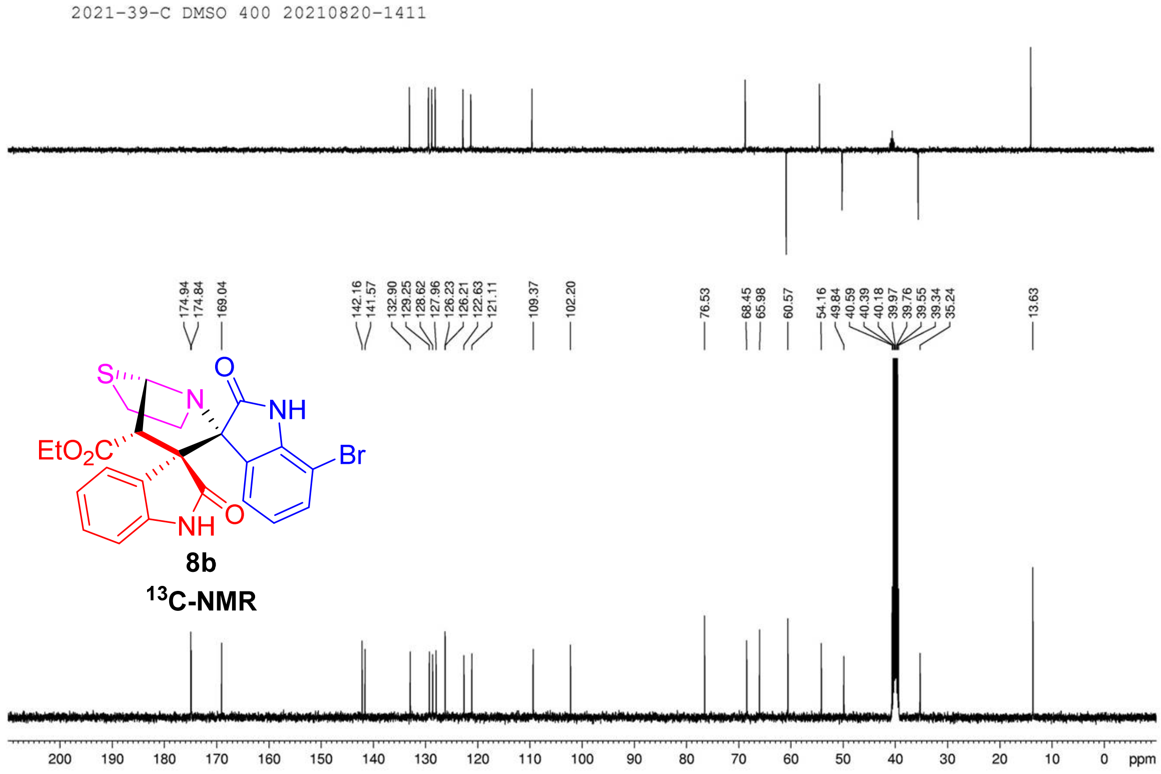
2021-39-C DMSO 400 20210820-1411
8b
13C-NMR
ppm

2

## Qualitative Compound Report

| | | | |
|---|---|---|---|
| Data File | 8036980-22-29.d | Sample Name | 8036980-22-29 |
| Sample Type | Sample | Position | P1-D4 |
| Instrument Name | Instrument 1 | User Name | |
| Acq Method | pos-1min.m | Acquired Time | 8/8/2022 5:49:36 PM |
| IRM Calibration Status | Success | DA Method | default.m |
| Comment | | | |
| Sample Group | | Info. | |
| Stream Name | LC 1 | Acquisition SW Version | 6200 series TOF/6500 series Q-TOF B.08.00 (B8058.0) |

**Compound Table**

| Compound Label | RT | Mass | Abund | Formula | Tgt Mass | Diff (ppm) |
|---|---|---|---|---|---|---|
| Cpd 1: C23H20 Br N3 O4 S | 0.205 | 513.0627 | 273863 | C23H20 Br N3 O4 S | 513.0605 | 3.32 |

MS Zoomed Spectrum

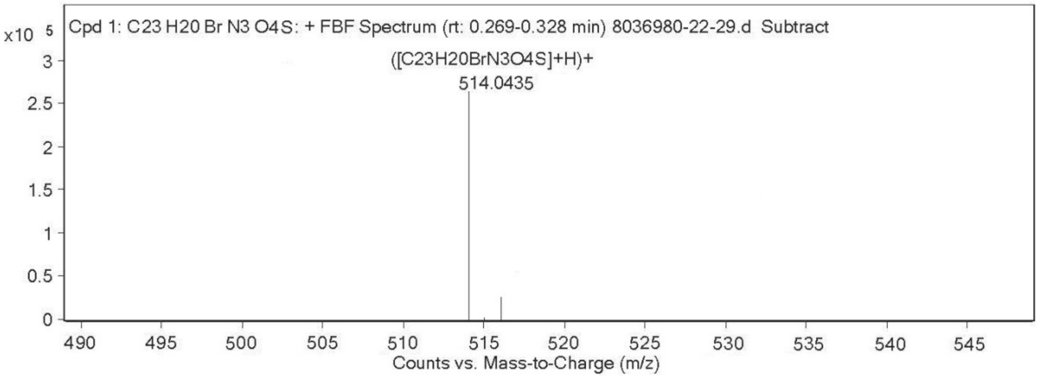


--- End Of Report ---

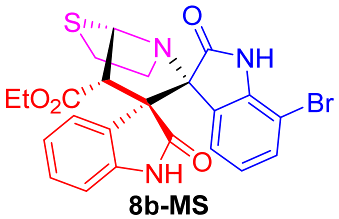


**8b-MS**

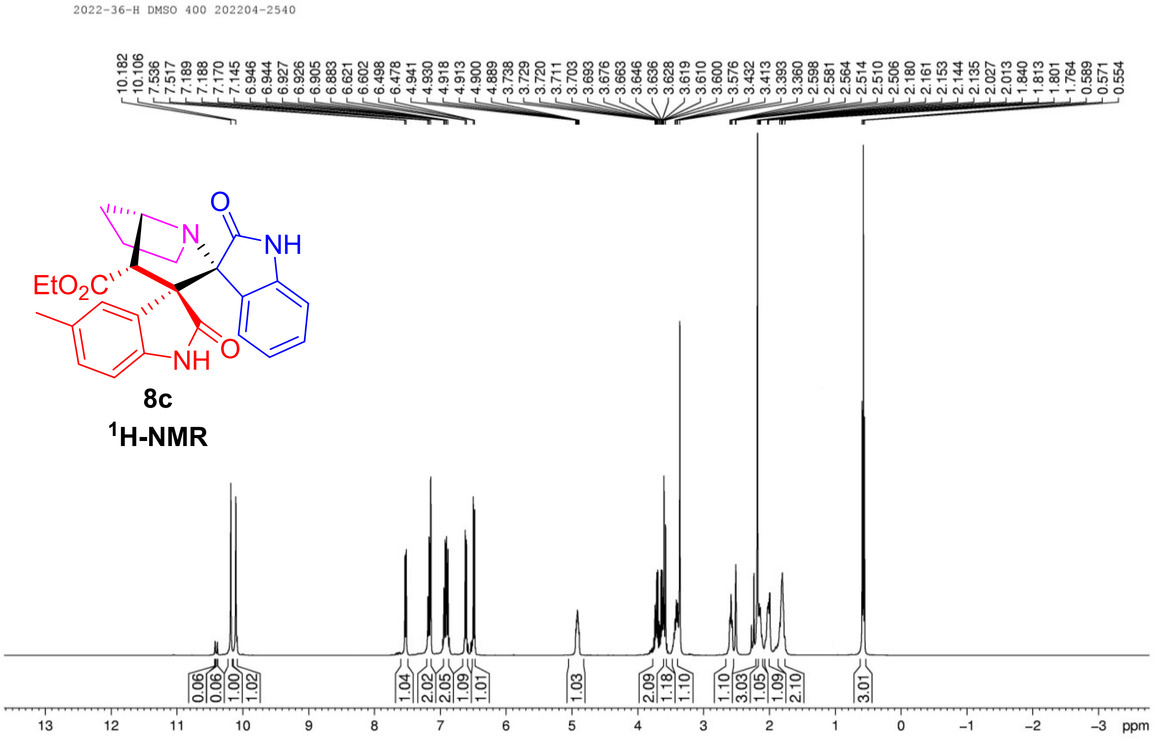
2022-36-H DMSO 400 202204-2540
10.182
10.106
7.536
7.517
7.189
7.188
7.170
7.145
6.946
6.944
6.927
6.926
6.905
6.883
6.621
6.602
6.498
6.478
4.941
4.930
4.918
4.913
4.900
4.889
3.738
3.729
3.720
3.711
3.703
3.693
3.676
3.663
3.646
3.636
3.628
3.619
3.610
3.600
3.576
3.432
3.413
3.393
3.360
2.598
2.581
2.564
2.514
2.510
2.506
2.180
2.161
2.153
2.144
2.135
2.027
2.013
1.840
1.813
1.801
1.764
0.589
0.571
0.554
O
N
NH
EtO2C
NH
O
8c
1H-NMR
0.06
0.06
1.00
1.02
1.04
2.02
2.05
1.09
1.01
1.03
2.09
1.18
1.10
1.10
3.03
1.05
1.09
2.10
3.01
13
12
11
10
9
8
7
6
5
4
3
2
1
0
−1
−2
−3
ppm

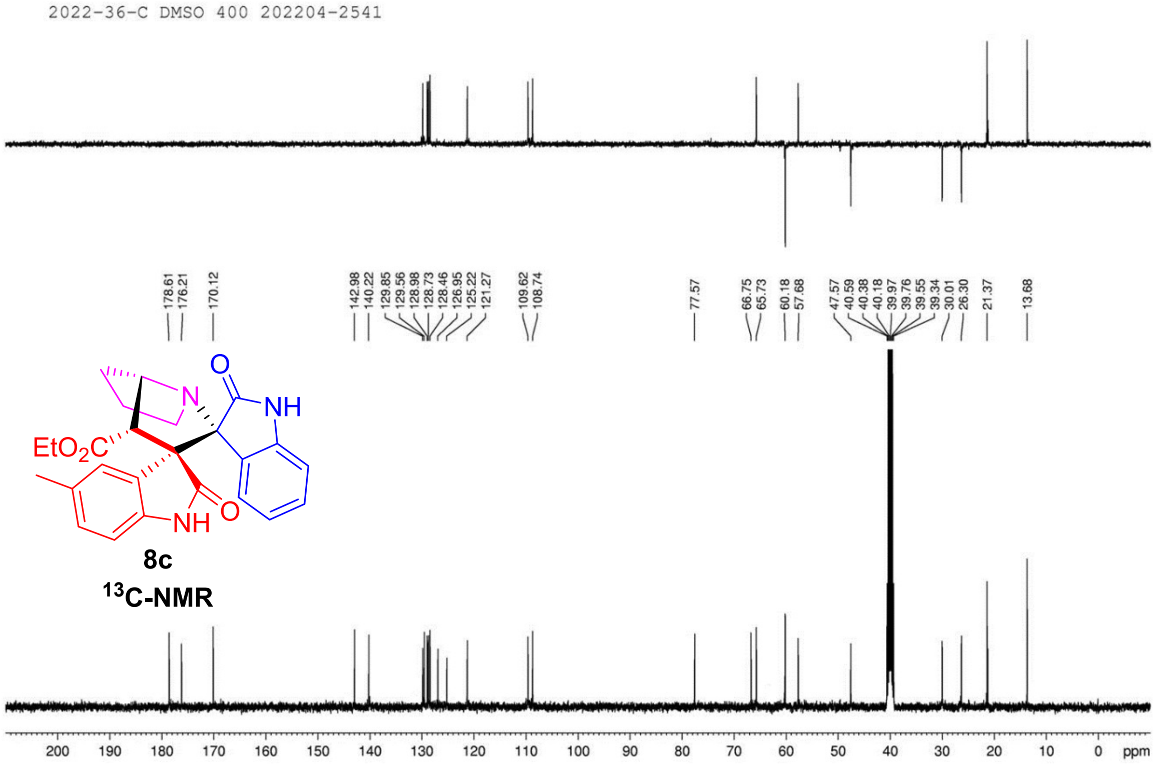
2022-36-C DMSO 400 202204-2541
178.61
176.21
170.12
142.98
140.22
129.85
129.56
128.98
128.73
128.46
126.95
125.22
121.27
109.62
108.74
77.57
66.75
65.73
60.18
57.68
47.57
40.59
40.38
40.18
39.97
39.76
39.55
39.34
30.01
26.30
21.37
13.68
O
N
NH
EtO2C
NH
O
8c
13C-NMR
200
190
180
170
160
150
140
130
120
110
100
90
80
70
60
50
40
30
20
10
0
ppm

# Qualitative Compound Report

| | | | |
|---|---|---|---|
| Data File | 2033368-22-36.d | Sample Name | 2033368-22-36 |
| Sample Type | Sample | Position | P1-B7 |
| Instrument Name | Instrument 1 | User Name | |
| Acq Method | pos-1min.m | Acquired Time | 2/6/2023 5:30:55 PM |
| IRM Calibration Status | Success | DA Method | QG-907.m |
| Comment | | | |
| Sample Group | | Info. | |
| Stream Name | LC 1 | Acquisition SW Version | 6200 series TOF/6500 series Q-TOF B.08.00 (B8058.0) |

**Compound Table**

| Compound Label | RT | Mass | Abund | Formula | Tgt Mass | Diff (ppm) |
|---|---|---|---|---|---|---|
| Cpd 1: C25 H25 N3 O4 | 0.154 | 431.1852 | 575936 | C25 H25 N3 O4 | 431.1845 | 1.52 |

MS Zoomed Spectrum

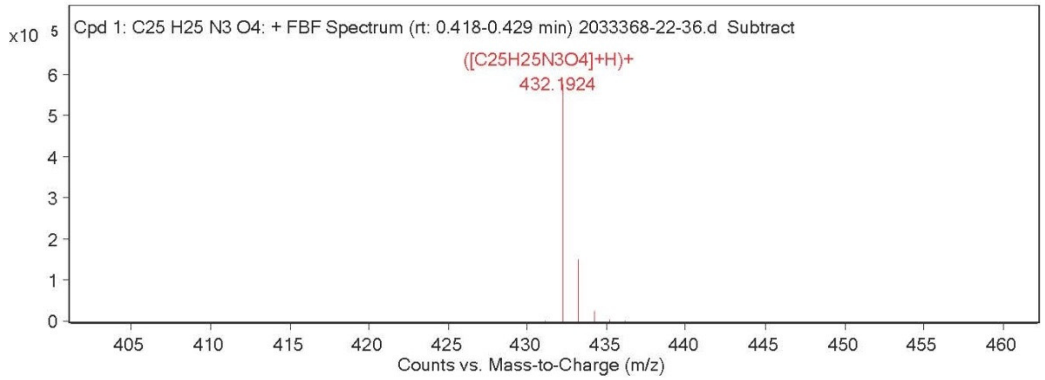


--- End Of Report ---

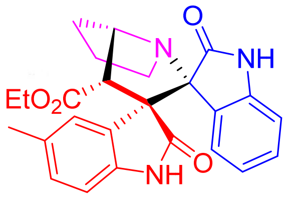


**8c-MS**

2022-37-H DMSO 400 w(-)-2340

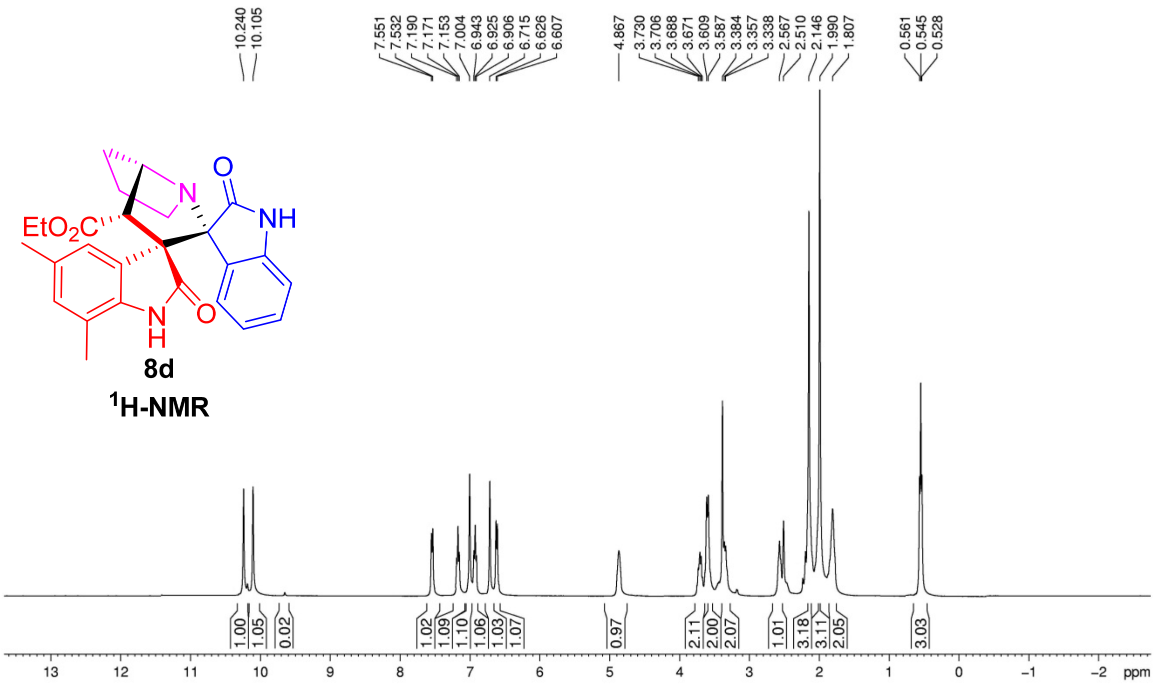


2022-37-C DMSO 400 w(-)-2341

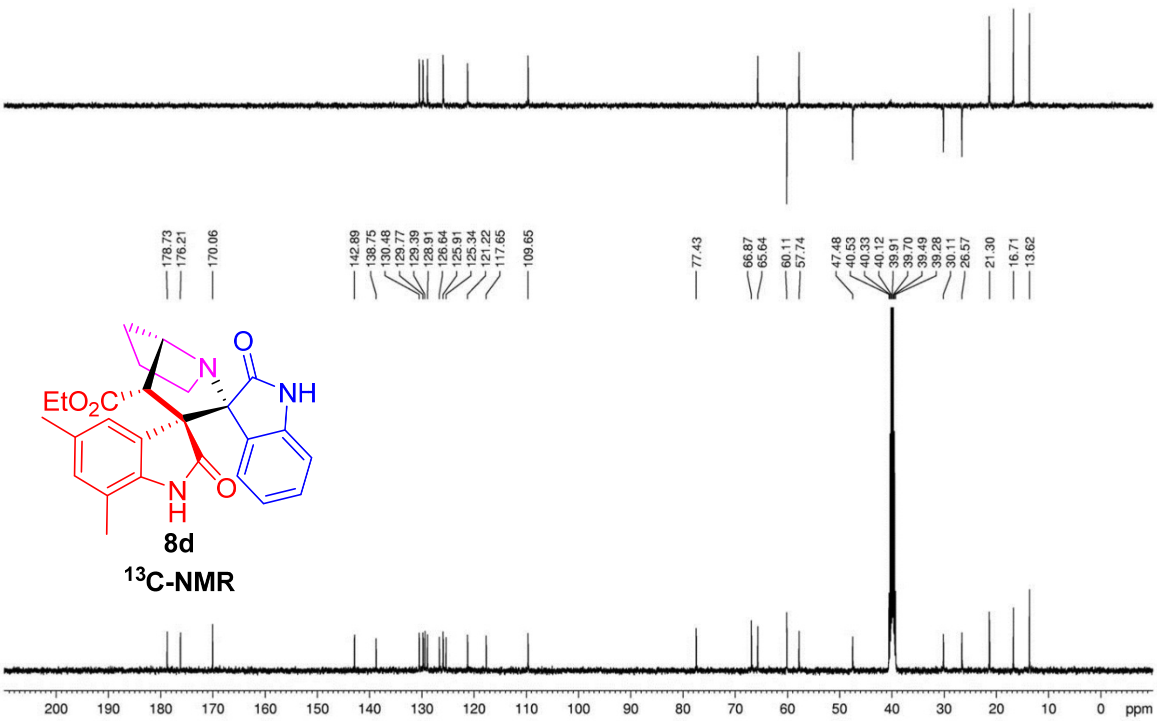

## Qualitative Compound Report

| | | | |
|---|---|---|---|
| Data File | 8036980-22-37.d | Sample Name | 8036980-22-37 |
| Sample Type | Sample | Position | P1-D7 |
| Instrument Name | Instrument 1 | User Name | |
| Acq Method | pos-1min.m | Acquired Time | 8/8/2022 5:43:48 PM |
| IRM Calibration Status | Success | DA Method | default.m |
| Comment | | | |
| Sample Group | | Info. | |
| Stream Name | LC 1 | Acquisition SW Version | 6200 series TOF/6500 series Q-TOF B.08.00 (B8058.0) |

**Compound Table**

| Compound Label | RT | Mass | Abund | Formula | Tgt Mass | Diff (ppm) |
|---|---|---|---|---|---|---|
| Cpd 1: C26 H27 N3 O4 | 0.297 | 445.2021 | 1756304 | C26 H27 N3 O4 | 445.2002 | 4.35 |

MS Zoomed Spectrum

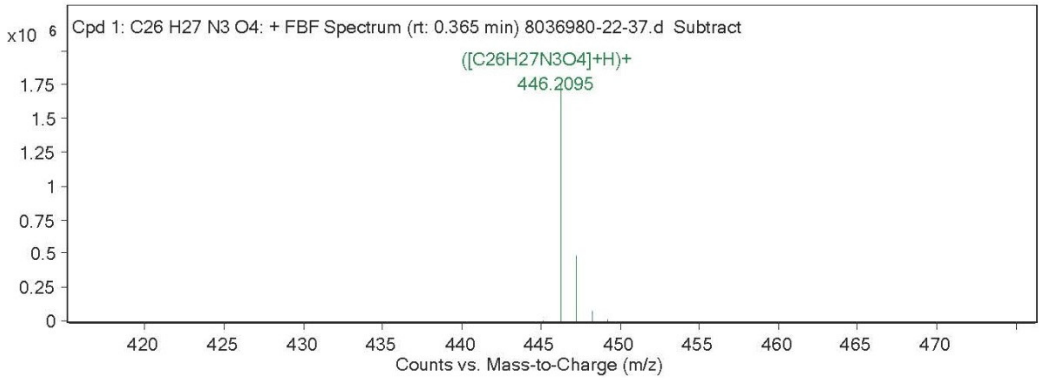


--- End Of Report ---

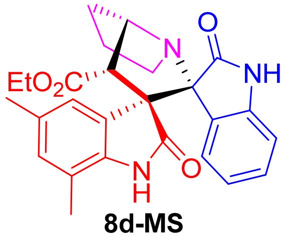


**8d-MS**

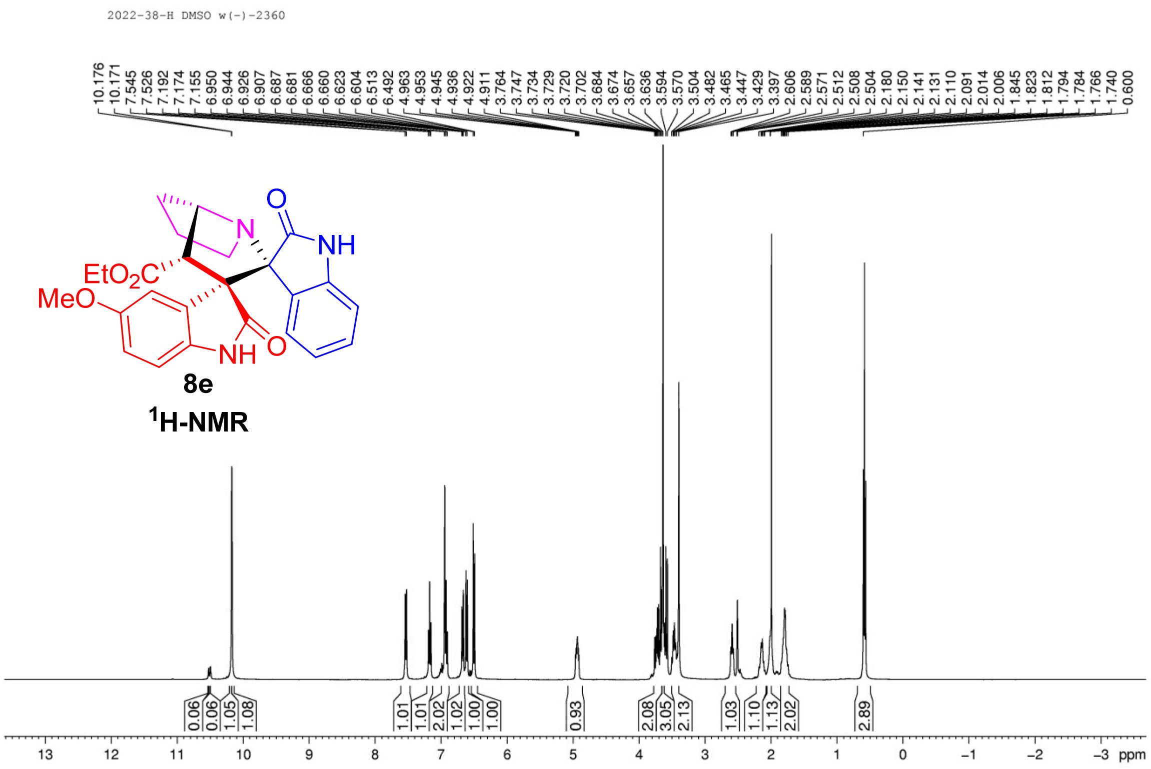
2022-38-H DMSO w(-)-2360
8e
1H-NMR

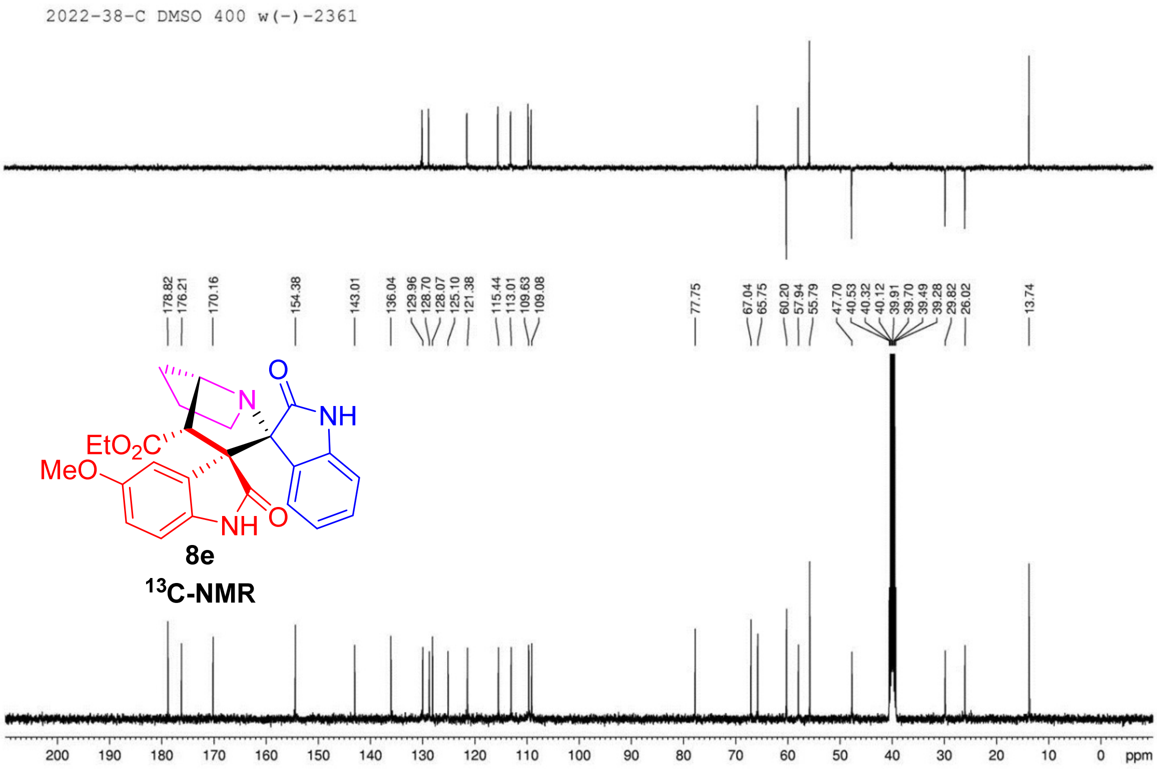
2022-38-C DMSO 400 w(-)-2361
8e
13C-NMR

# Qualitative Compound Report

| | | | |
|---|---|---|---|
| Data File | 8036980-22-38.d | Sample Name | 8036980-22-38 |
| Sample Type | Sample | Position | P1-D6 |
| Instrument Name | Instrument 1 | User Name | |
| Acq Method | pos-1min.m | Acquired Time | 8/8/2022 5:41:57 PM |
| IRM Calibration Status | Success | DA Method | default.m |
| Comment | | | |
| Sample Group | | Info. | |
| Stream Name | LC 1 | Acquisition SW Version | 6200 series TOF/6500 series Q-TOF B.08.00 (B8058.0) |

**Compound Table**

| Compound Label | RT | Mass | Abund | Formula | Tgt Mass | Diff (ppm) |
|---|---|---|---|---|---|---|
| Cpd 1: C25 H25 N3 O5 | 0.253 | 447.1806 | 550043 | C25 H25 N3 O5 | 447.1794 | 2.74 |

MS Zoomed Spectrum

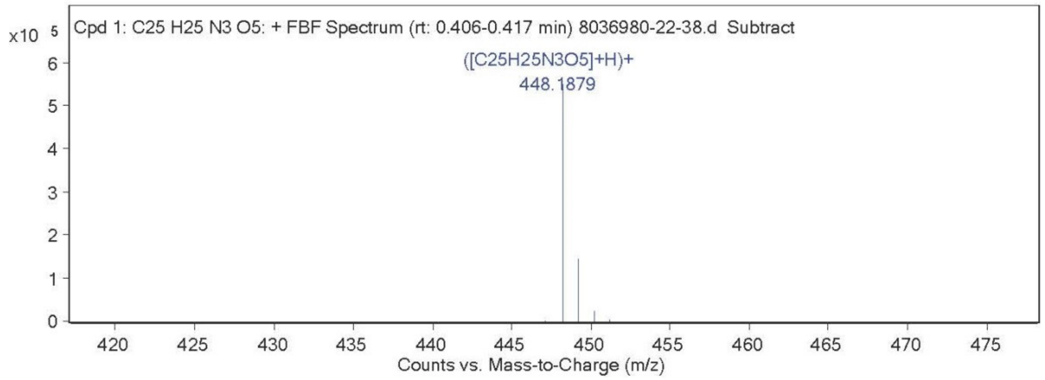


--- End Of Report ---

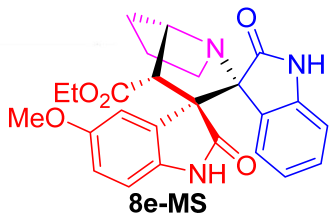


**8e-MS**

# 3. $^{1}H$ and $^{13}C$ NMR spectra for compounds 9

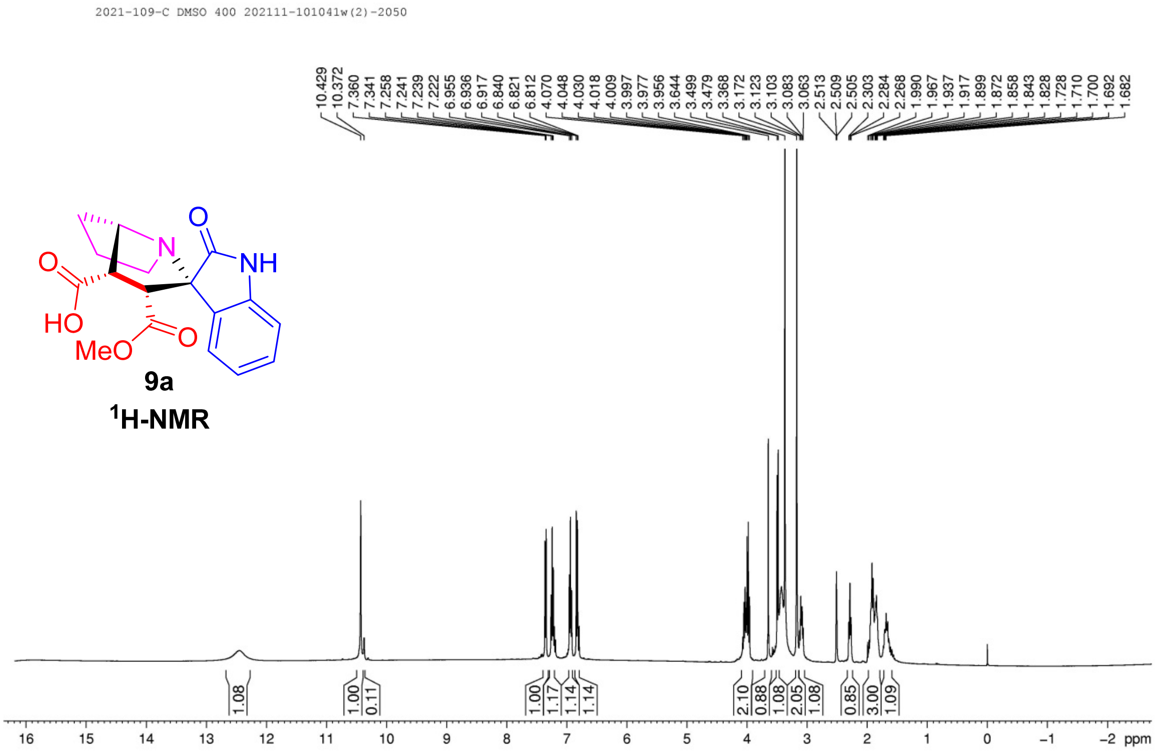


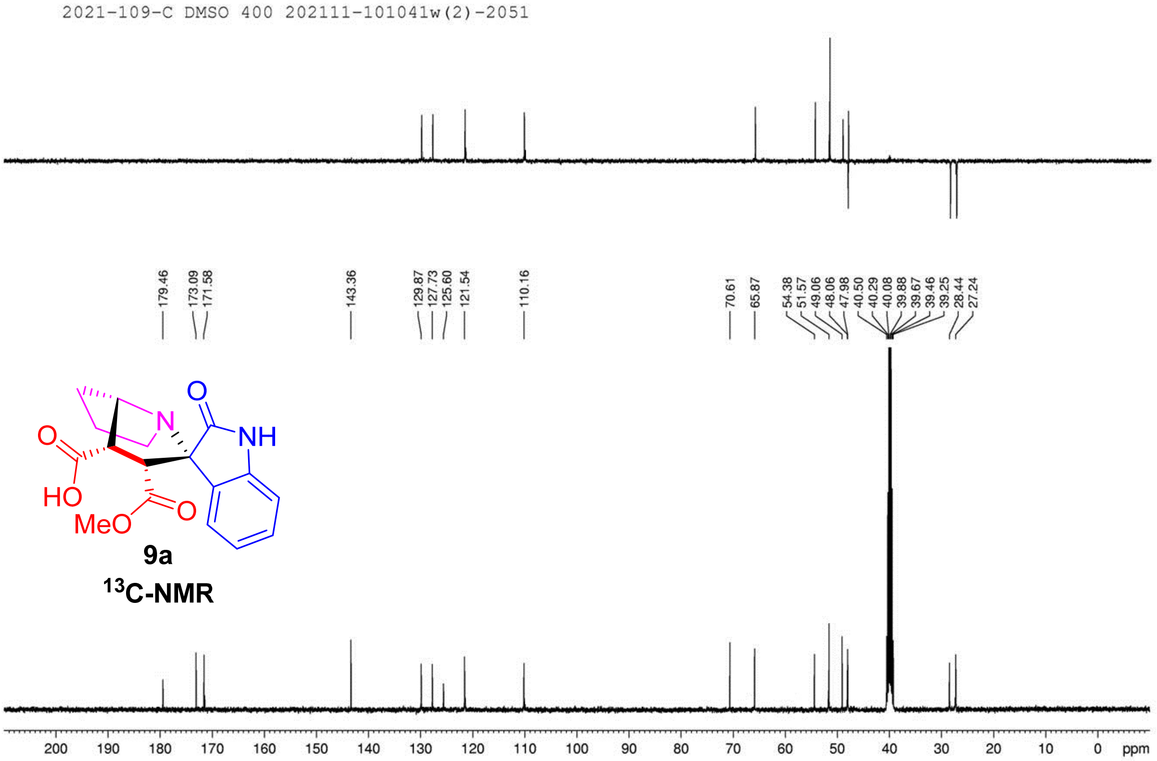

## Qualitative Compound Report

| | | | |
|---|---|---|---|
| **Data File** | 8153503.d | **Sample Name** | 8153503 |
| **Sample Type** | Sample | **Position** | P1-A3 |
| **Instrument Name** | Instrument 1 | **User Name** | |
| **Acq Method** | pos-1min.m | **Acquired Time** | 8/18/2022 10:25:15 PM |
| **IRM Calibration Status** | Success | **DA Method** | default.m |
| **Comment** | | | |
| **Sample Group** | | **Info.** | |
| **Stream Name** | LC 1 | **Acquisition SW Version** | 6200 series TOF/6500 series Q-TOF B.08.00 (B8058.0) |

**Compound Table**

| Compound Label | RT | Mass | Abund | Formula | Tgt Mass | Diff (ppm) |
|---|---|---|---|---|---|---|
| Cpd 1: C17 H18 N2 O5 | 0.321 | 330.1225 | 302325 | C17 H18 N2 O5 | 330.1215 | 3.15 |

MS Zoomed Spectrum

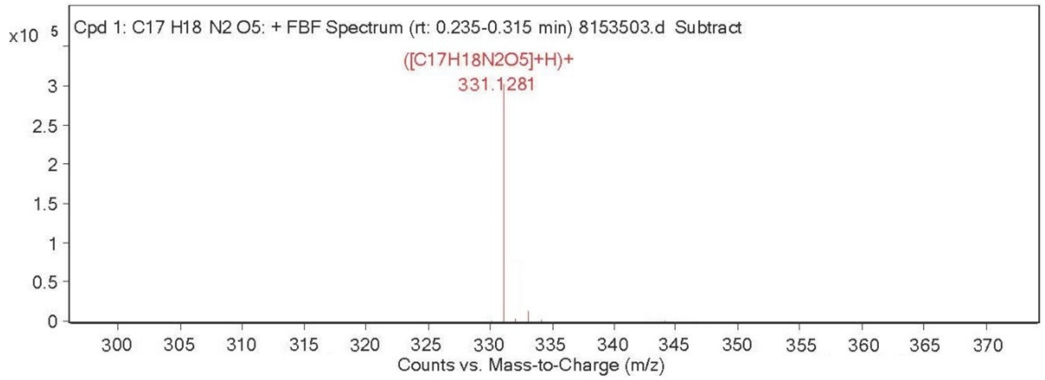


--- End Of Report ---

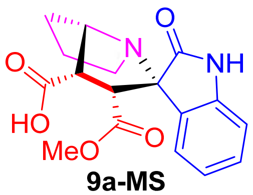


**9a-MS**

2022-56-H DMSO 400 20220712(2790)-2870

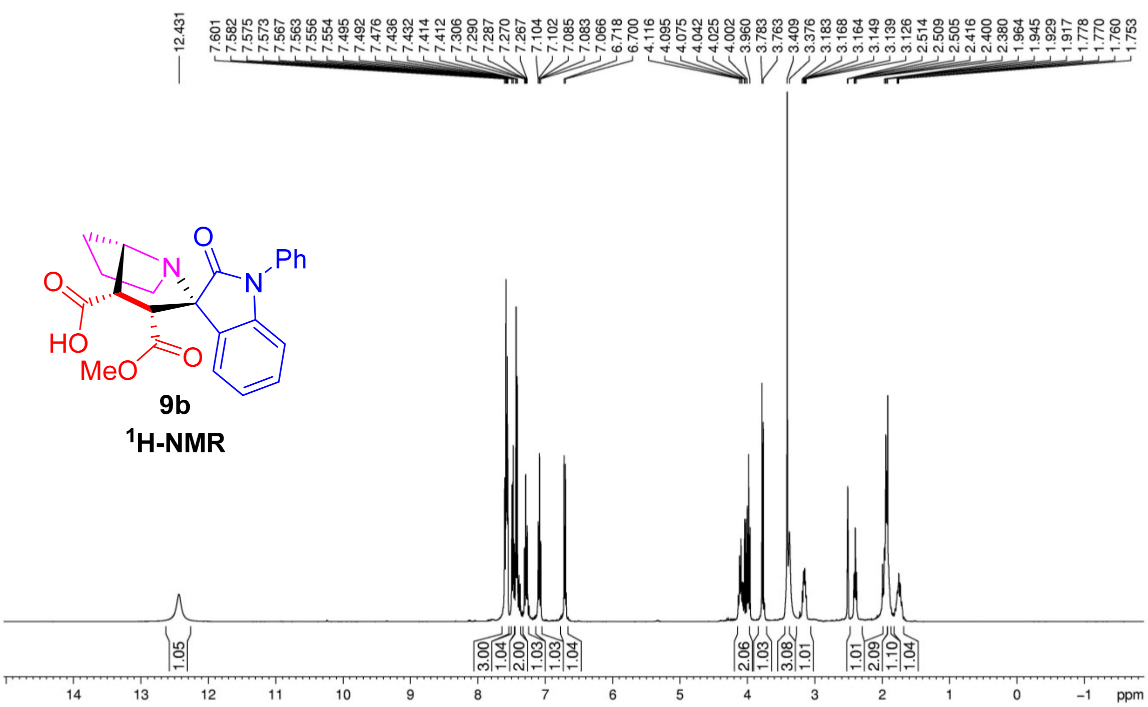


2022-56-C DMSO 400 20220712(2790)-2871

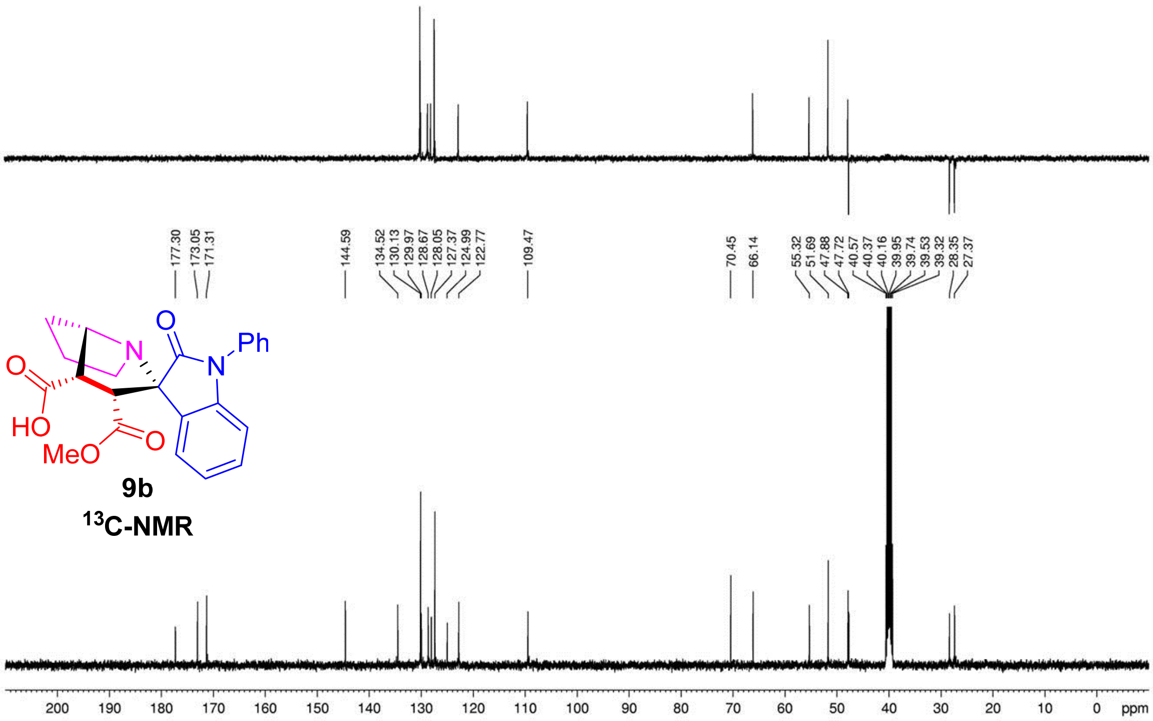

## Qualitative Compound Report

| | | | |
|---|---|---|---|
| Data File | 8153501.d | Sample Name | 8153501 |
| Sample Type | Sample | Position | P1-A5 |
| Instrument Name | Instrument 1 | User Name | |
| Acq Method | pos-1min.m | Acquired Time | 8/18/2022 10:45:42 PM |
| IRM Calibration Status | Success | DA Method | default.m |
| Comment | | | |
| Sample Group | | Info. | |
| Stream Name | LC 1 | Acquisition SW Version | 6200 series TOF/6500 series Q-TOF B.08.00 (B8058.0) |

**Compound Table**

| Compound Label | RT | Mass | Abund | Formula | Tgt Mass | Diff (ppm) |
|---|---|---|---|---|---|---|
| Cpd 1: C23 H22 N2 O5 | 0.151 | 406.1542 | 302015 | C23 H22 N2 O5 | 406.1529 | 3.27 |

MS Zoomed Spectrum

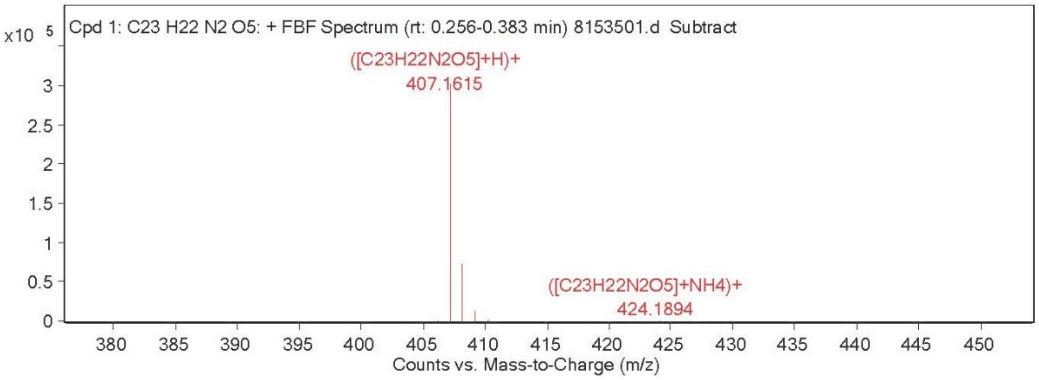


--- End Of Report ---

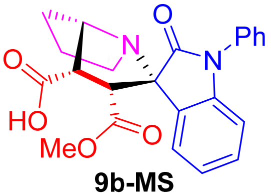


**9b-MS**

# 4. $^1$H and $^{13}$C NMR spectra for compounds 10

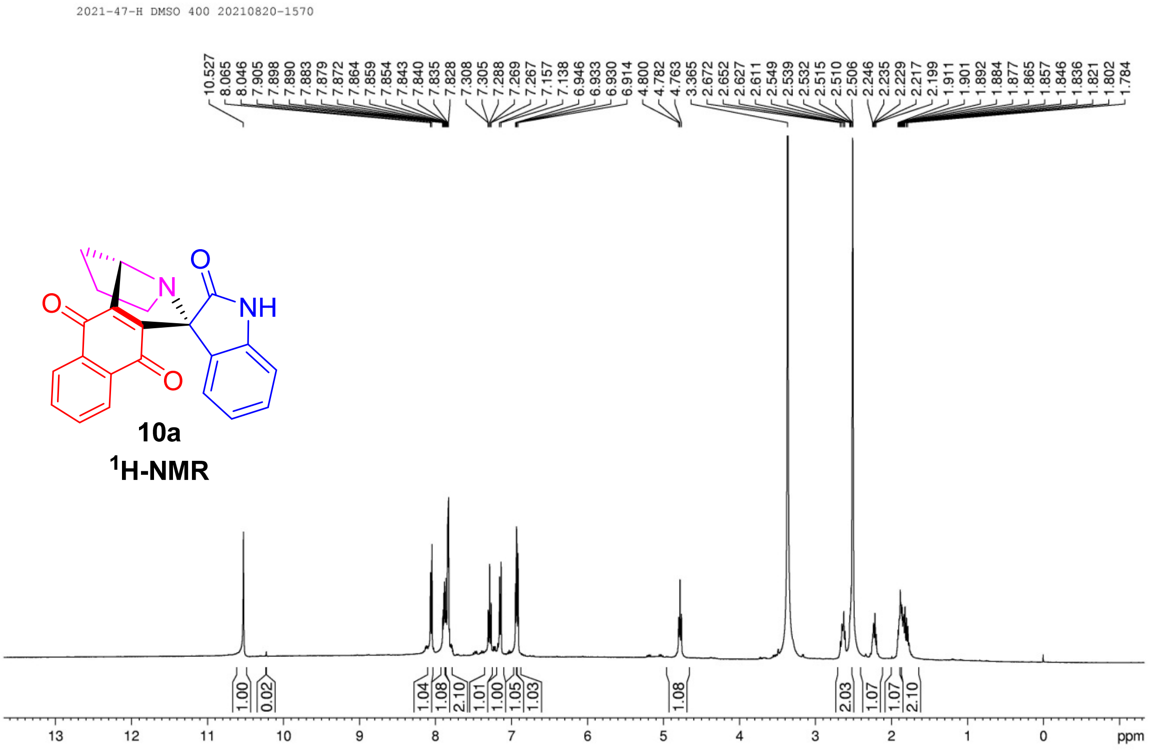


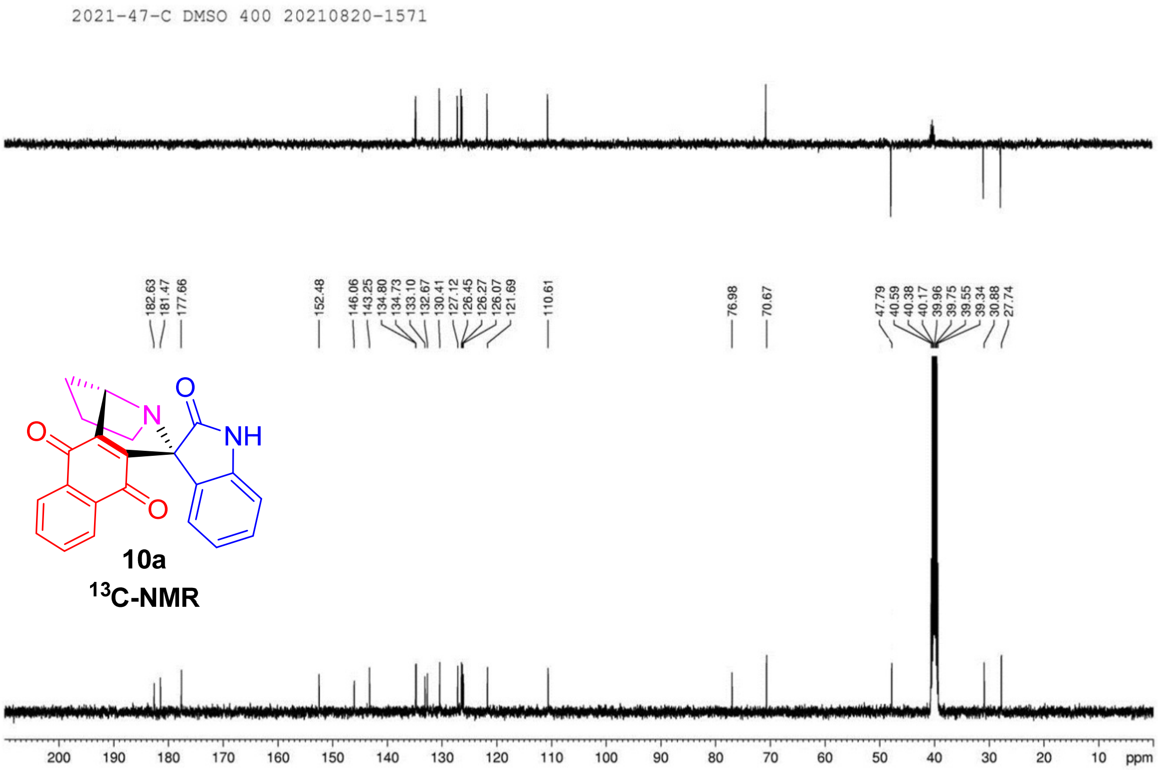

## Qualitative Compound Report

| | | | |
|---|---|---|---|
| **Data File** | 2033368-21-47.d | **Sample Name** | 2033368-21-47 |
| **Sample Type** | Sample | **Position** | P1-B9 |
| **Instrument Name** | Instrument 1 | **User Name** | |
| **Acq Method** | pos-1min.m | **Acquired Time** | 2/6/2023 5:34:41 PM |
| **IRM Calibration Status** | Success | **DA Method** | QG-907.m |
| **Comment** | | | |
| **Sample Group** | | **Info.** | |
| **Stream Name** | LC 1 | **Acquisition SW Version** | 6200 series TOF/6500 series Q-TOF B.08.00 (B8058.0) |

**Compound Table**

| Compound Label | RT | Mass | Abund | Formula | Tgt Mass | Diff (ppm) |
|---|---|---|---|---|---|---|
| Cpd 1: C22 H16 N2 O3 | 0.529 | 356.1176 | 2122562 | C22 H16 N2 O3 | 356.1161 | 4.34 |

MS Zoomed Spectrum

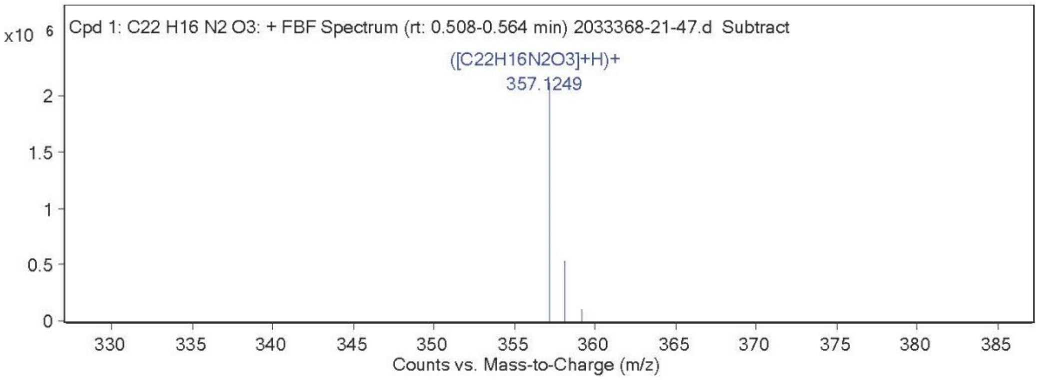


--- End Of Report ---

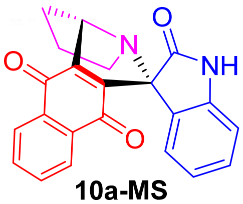


**10a-MS**

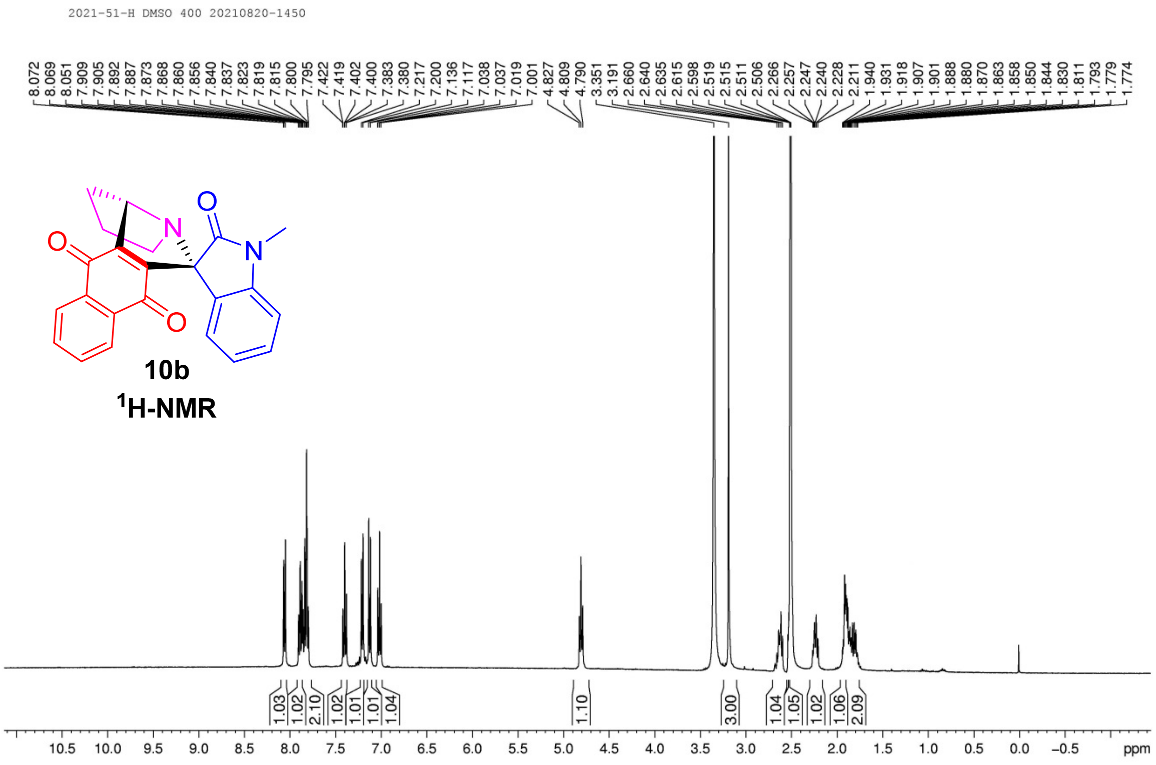
2021-51-H DMSO 400 20210820-1450
10b
1H-NMR
ppm

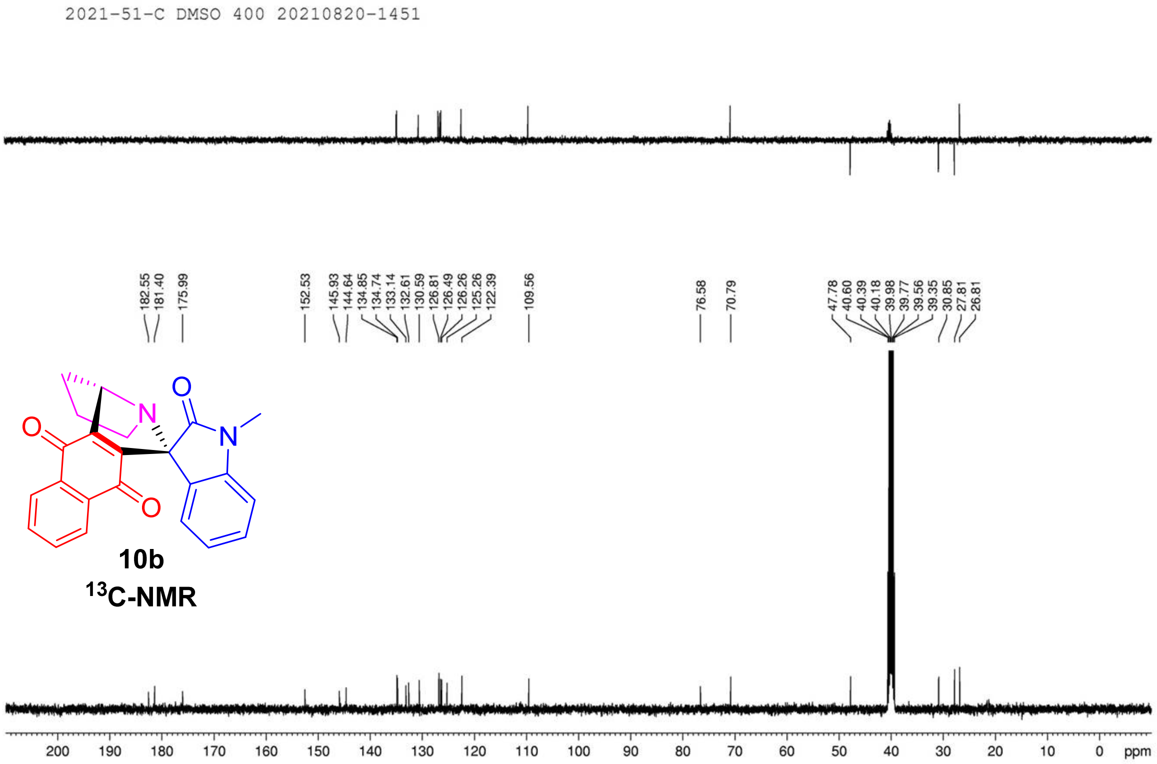
2021-51-C DMSO 400 20210820-1451
10b
13C-NMR
ppm

## Qualitative Compound Report

| | | | |
|---|---|---|---|
| **Data File** | 2033368-22-51.d | **Sample Name** | 2033368-22-51 |
| **Sample Type** | Sample | **Position** | P1-C4 |
| **Instrument Name** | Instrument 1 | **User Name** | |
| **Acq Method** | pos-1min.m | **Acquired Time** | 2/6/2023 5:42:06 PM |
| **IRM Calibration Status** | Success | **DA Method** | QG-907.m |
| **Comment** | | | |
| **Sample Group** | | **Info.** | |
| **Stream Name** | LC 1 | **Acquisition SW Version** | 6200 series TOF/6500 series Q-TOF B.08.00 (B8058.0) |

**Compound Table**

| Compound Label | RT | Mass | Abund | Formula | Tgt Mass | Diff (ppm) |
|---|---|---|---|---|---|---|
| Cpd 1: C23 H18 N2 O3 | 0.447 | 370.1327 | 213394 | C23 H18 N2 O3 | 370.1317 | 2.55 |

MS Zoomed Spectrum

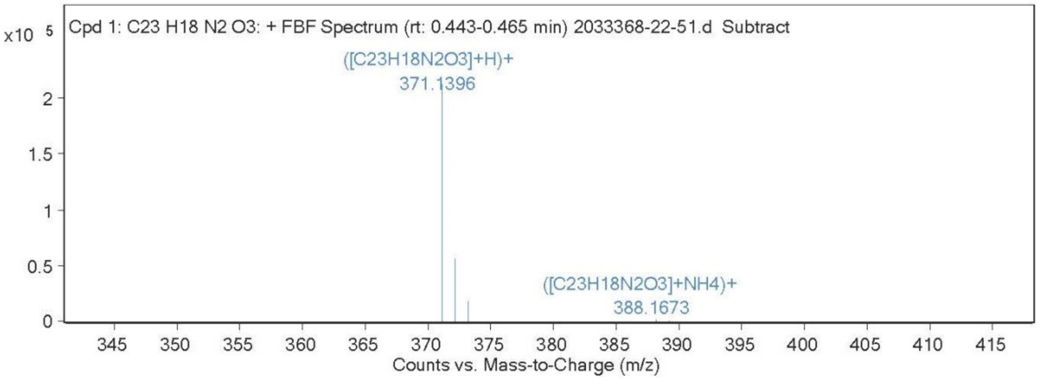


--- End Of Report ---

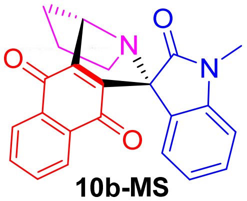

**10b-MS**

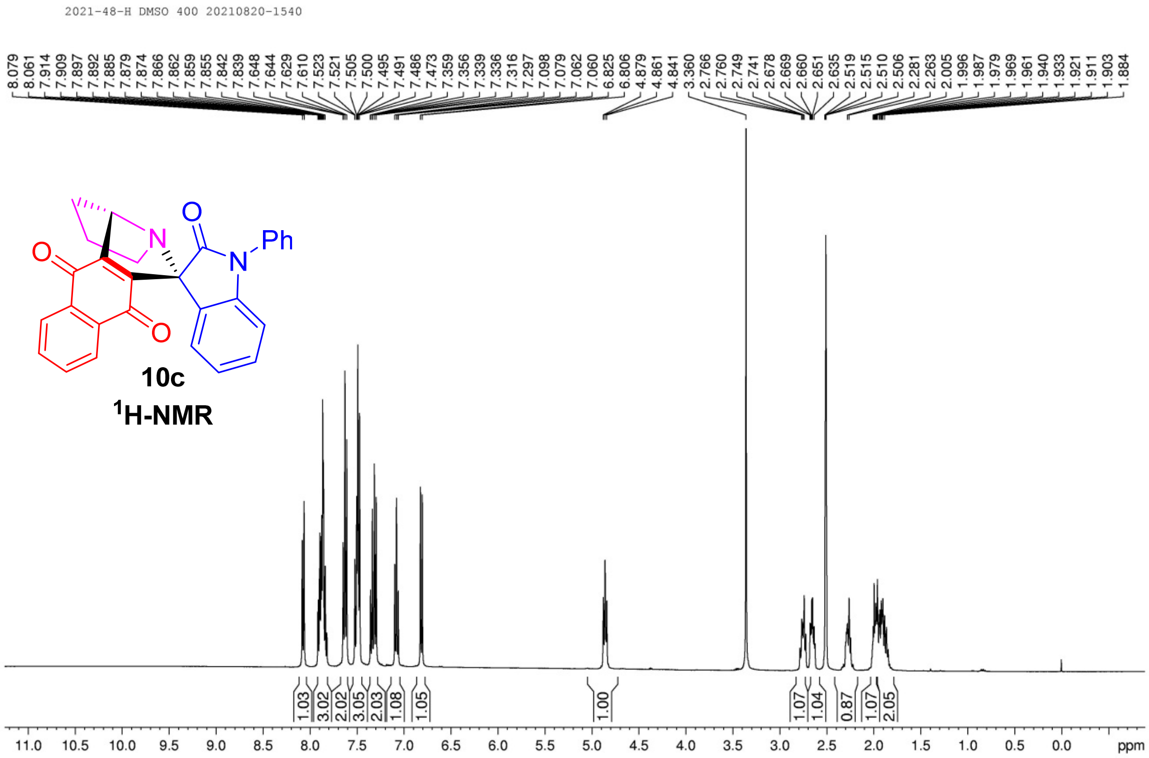

2021-48-H DMSO 400 20210820-1540
8.079 8.061 7.914 7.909 7.897 7.892 7.885 7.879 7.874 7.866 7.862 7.859 7.855 7.842 7.839 7.648 7.644 7.629 7.610 7.523 7.521 7.505 7.500 7.495 7.491 7.486 7.473 7.359 7.356 7.339 7.336 7.316 7.297 7.098 7.079 7.062 7.060 6.825 6.806 4.879 4.861 4.841 3.360 2.766 2.760 2.749 2.741 2.678 2.669 2.660 2.651 2.635 2.519 2.515 2.510 2.506 2.281 2.263 2.005 1.996 1.987 1.979 1.969 1.961 1.940 1.933 1.921 1.911 1.903 1.884
O
N
O
N
Ph
O
10c
1H-NMR
1.03 3.02 2.02 3.05 2.03 1.08 1.05 1.00 1.07 1.04 0.87 1.07 2.05
11.0 10.5 10.0 9.5 9.0 8.5 8.0 7.5 7.0 6.5 6.0 5.5 5.0 4.5 4.0 3.5 3.0 2.5 2.0 1.5 1.0 0.5 0.0 ppm


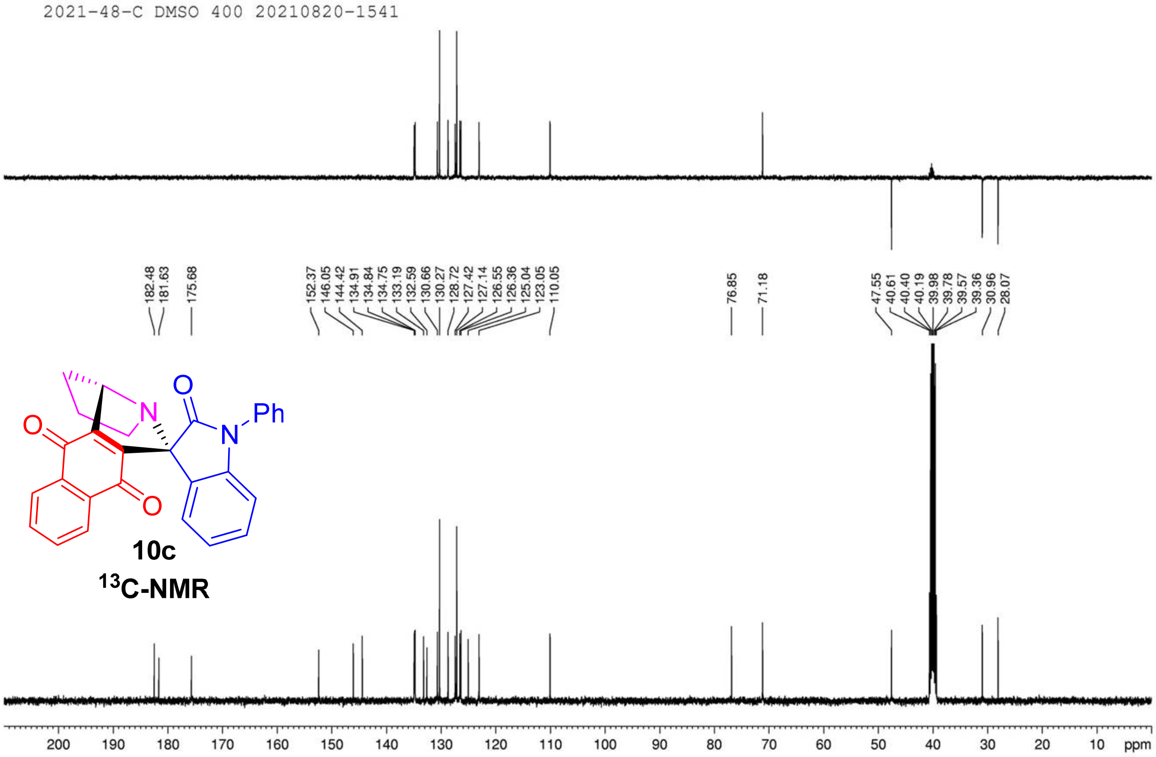

2021-48-C DMSO 400 20210820-1541
182.48 181.63 175.68 152.37 146.05 144.42 134.91 134.84 134.75 133.19 132.59 130.66 130.27 128.72 127.42 127.14 126.55 126.36 125.04 123.05 110.05 76.85 71.18 47.55 40.61 40.40 40.19 39.98 39.78 39.57 39.36 30.96 28.07
O
N
O
N
Ph
O
10c
13C-NMR
200 190 180 170 160 150 140 130 120 110 100 90 80 70 60 50 40 30 20 10 ppm

# Qualitative Compound Report

| | | | |
|---|---|---|---|
| Data File | 7196909-2021-48. d | Sample Name | 7196909-2021-48 |
| Sample Type | Sample | Position | P1-F7 |
| Instrument Name | Instrument 1 | User Name | |
| Acq Method | pos-1min.m | Acquired Time | 2022-07-22 10:22:33 |
| IRM Calibration Status | Success | DA Method | ZNZ-2022. m |
| Comment | | | |
| Sample Group | | Info. | |
| Stream Name | LC 1 | Acquisition SW Version | 6200 series TOF/6500 series Q-TOF B.08.00 (B8058.0) |

**Compound Table**

| Compound Label | RT | Mass | Abund | Formula | Tgt Mass | Diff (ppm) |
|---|---|---|---|---|---|---|
| Cpd 1: C28 H20 N2 O3 | 0. 11 | 432. 1484 | 178300 | C28 H20 N2 O3 | 432. 1474 | 2. 44 |

MS Zoomed Spectrum

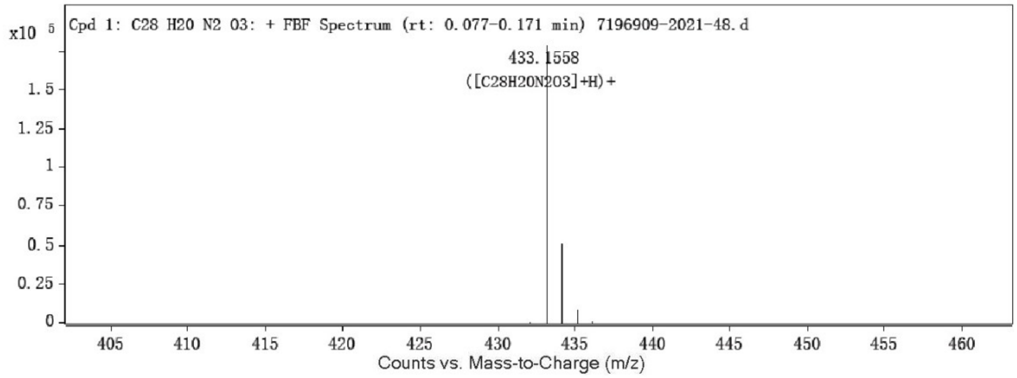


--- End Of Report ---

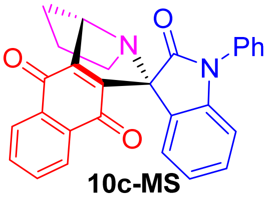


**10c-MS**

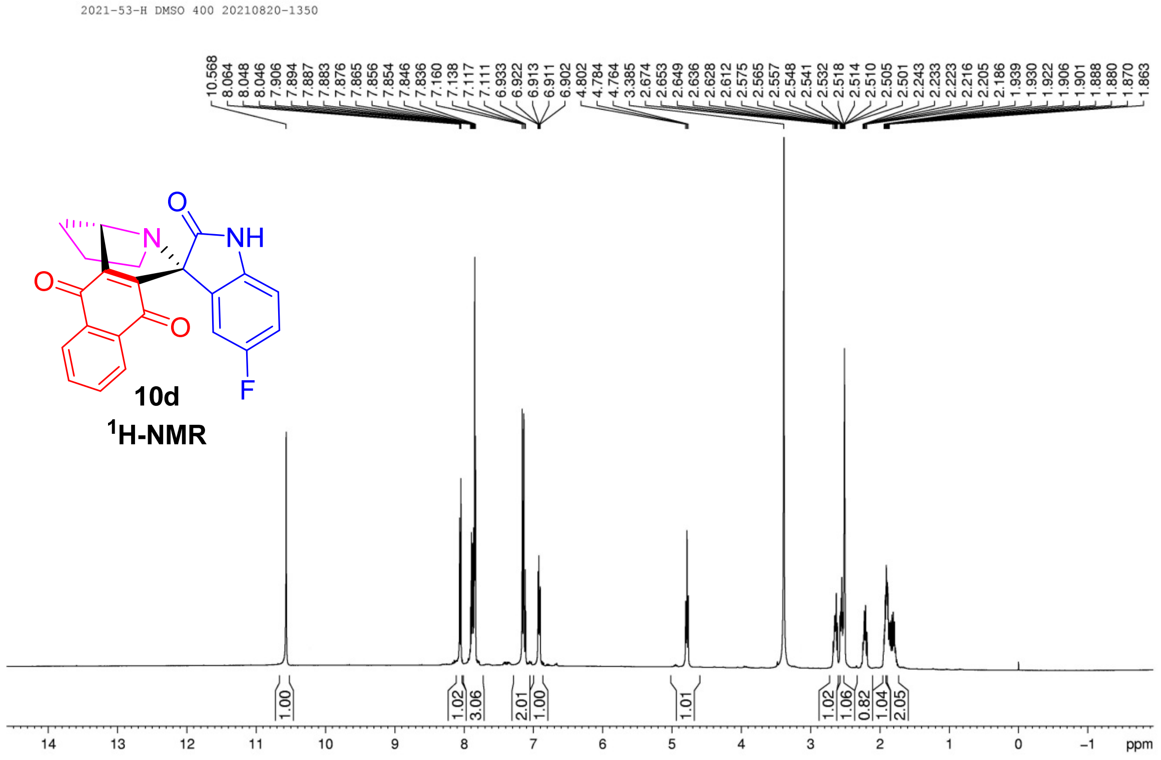
2021-53-H DMSO 400 20210820-1350
10.568
8.064
8.048
8.046
7.906
7.894
7.887
7.883
7.876
7.865
7.856
7.854
7.846
7.836
7.160
7.138
7.117
7.111
6.933
6.922
6.913
6.911
6.902
4.802
4.784
4.764
3.385
2.674
2.653
2.649
2.636
2.628
2.612
2.575
2.565
2.557
2.548
2.541
2.532
2.518
2.514
2.510
2.505
2.501
2.243
2.233
2.223
2.216
2.205
2.186
1.939
1.930
1.922
1.906
1.901
1.888
1.880
1.870
1.863
O
NH
N
O
O
F
10d
¹H-NMR
1.00
1.02
3.06
2.01
1.00
1.01
1.02
1.06
0.82
1.04
2.05
14
13
12
11
10
9
8
7
6
5
4
3
2
1
0
–1
ppm

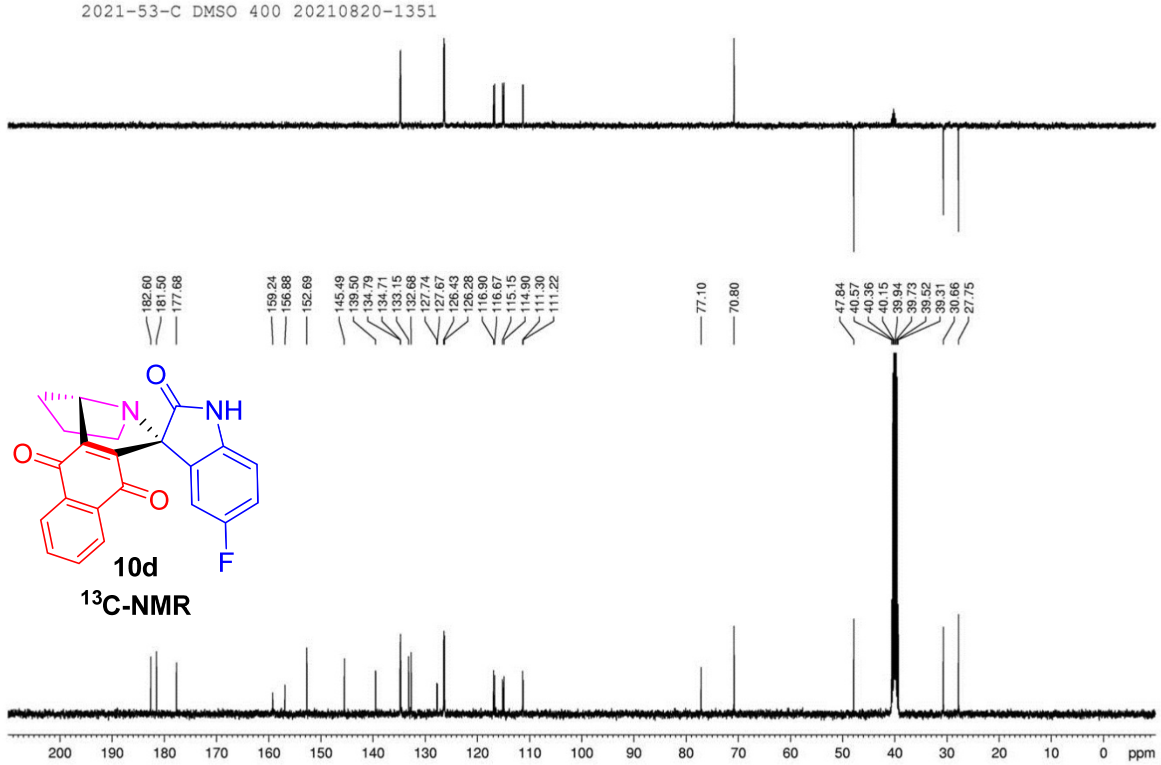
2021-53-C DMSO 400 20210820-1351
182.60
181.50
177.68
159.24
156.88
152.69
145.49
139.50
134.79
134.71
133.15
132.68
127.74
127.67
126.43
126.28
116.90
116.67
115.15
114.90
111.30
111.22
77.10
70.80
47.84
40.57
40.36
40.15
39.94
39.73
39.52
39.31
30.66
27.75
O
NH
N
O
O
F
10d
¹³C-NMR
200
190
180
170
160
150
140
130
120
110
100
90
80
70
60
50
40
30
20
10
0
ppm

## Qualitative Compound Report

| | | | |
|---|---|---|---|
| Data File | 2033368-21-53.d | Sample Name | 2033368-21-53 |
| Sample Type | Sample | Position | P1-C5 |
| Instrument Name | Instrument 1 | User Name | |
| Acq Method | pos-1min.m | Acquired Time | 2/6/2023 5:43:58 PM |
| IRM Calibration Status | Success | DA Method | QG-907.m |
| Comment | | | |
| Sample Group | | Info. | |
| Stream Name | LC 1 | Acquisition SW Version | 6200 series TOF/6500 series Q-TOF B.08.00 (B8058.0) |

**Compound Table**

| Compound Label | RT | Mass | Abund | Formula | Tgt Mass | Diff (ppm) |
|---|---|---|---|---|---|---|
| Cpd 1: C22 H15 F N2 O3 | 0.415 | 374.1071 | 53358 | C22 H15 F N2 O3 | 374.1067 | 1.16 |

MS Zoomed Spectrum

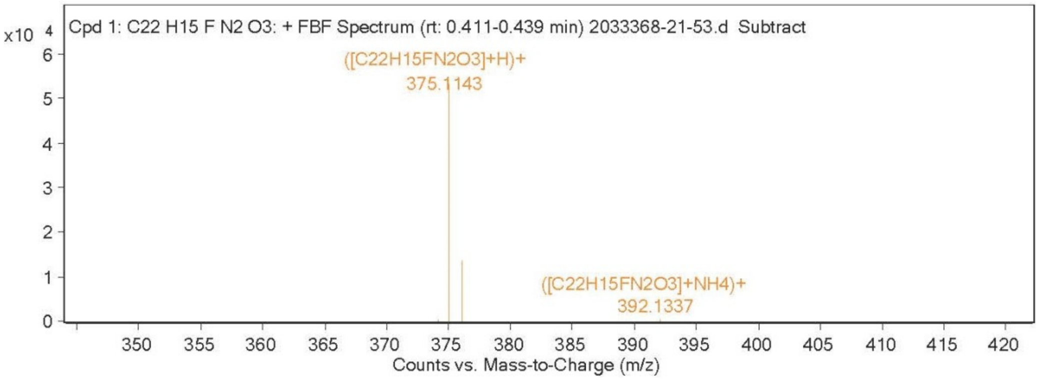


--- End Of Report ---

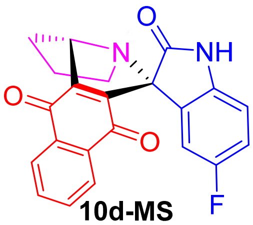

**10d-MS**

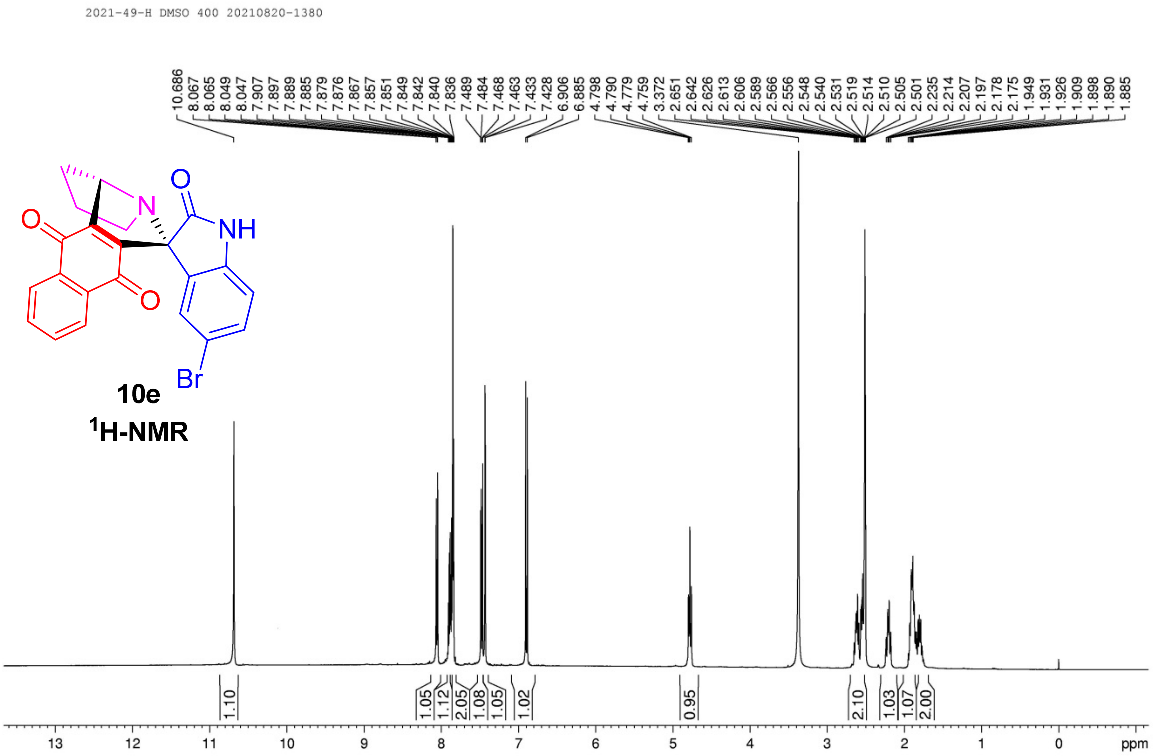
2021-49-H DMSO 400 20210820-1380
10.686 8.067 8.065 8.049 8.047 7.907 7.897 7.889 7.885 7.879 7.876 7.867 7.857 7.851 7.849 7.842 7.840 7.836 7.489 7.484 7.468 7.463 7.433 7.428 6.906 6.885 4.798 4.790 4.779 4.759 3.372 2.651 2.642 2.626 2.613 2.606 2.589 2.566 2.556 2.548 2.540 2.531 2.519 2.514 2.510 2.505 2.501 2.235 2.214 2.207 2.197 2.178 2.175 1.949 1.931 1.926 1.909 1.898 1.890 1.885
O
N
O
NH
O
Br
10e
$^{1}$H-NMR
1.10 1.05 1.12 2.05 1.08 1.05 1.02 0.95 2.10 1.03 1.07 2.00
13 12 11 10 9 8 7 6 5 4 3 2 1 0 ppm

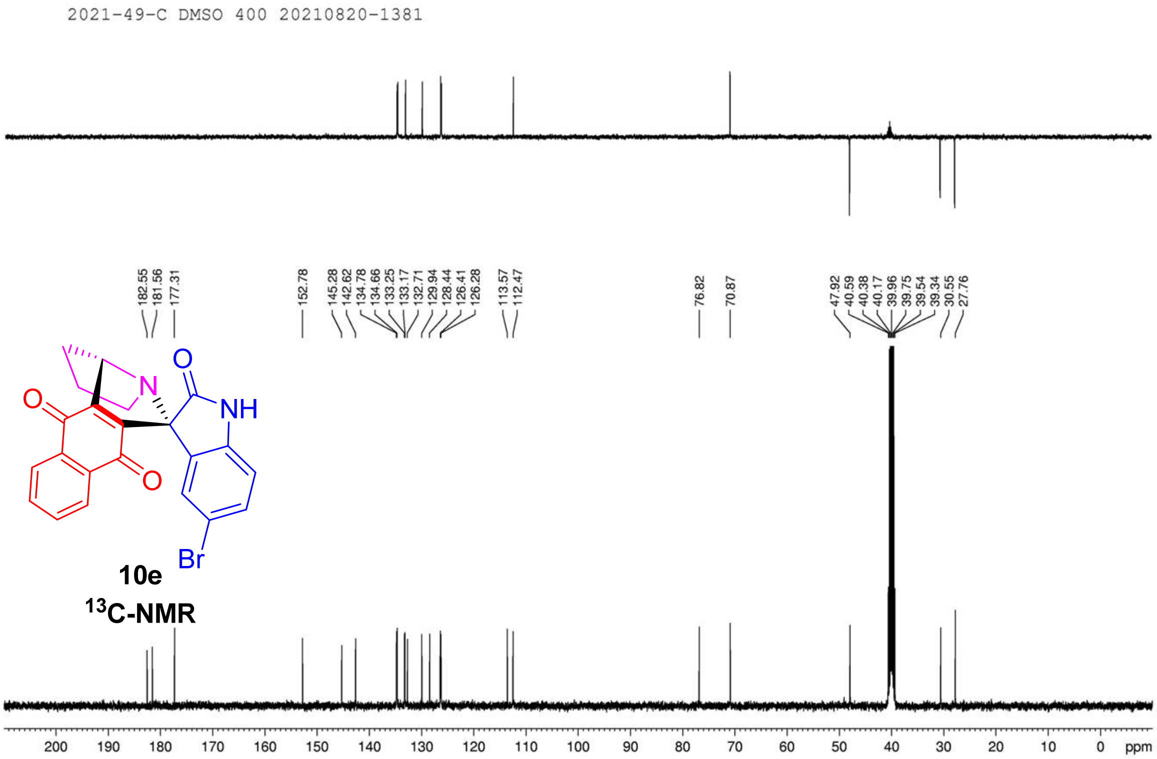
2021-49-C DMSO 400 20210820-1381
182.55 181.56 177.31 152.78 145.28 142.62 134.78 134.66 133.25 133.17 132.71 129.94 128.44 126.41 126.28 113.57 112.47 76.82 70.87 47.92 40.59 40.38 40.17 39.96 39.75 39.54 39.34 30.55 27.76
O
N
O
NH
O
Br
10e
$^{13}$C-NMR
200 190 180 170 160 150 140 130 120 110 100 90 80 70 60 50 40 30 20 10 0 ppm

## Qualitative Compound Report

| | | | |
|---|---|---|---|
| Data File | 2033368-22-35.d | Sample Name | 2033368-22-35 |
| Sample Type | Sample | Position | P1-B6 |
| Instrument Name | Instrument 1 | User Name | |
| Acq Method | pos-1min.m | Acquired Time | 2/6/2023 5:29:03 PM |
| IRM Calibration Status | Success | DA Method | QG-907.m |
| Comment | | | |
| Sample Group | | Info. | |
| Stream Name | LC 1 | Acquisition SW Version | 6200 series TOF/6500 series Q-TOF B.08.00 (B8058.0) |

**Compound Table**

| Compound Label | RT | Mass | Abund | Formula | Tgt Mass | Diff (ppm) |
|---|---|---|---|---|---|---|
| Cpd 1: C22 H15 Br N2 O3 | 0.127 | 434.028 | 194889 | C22 H15 Br N2 O3 | 434.0266 | 3.18 |

MS Zoomed Spectrum

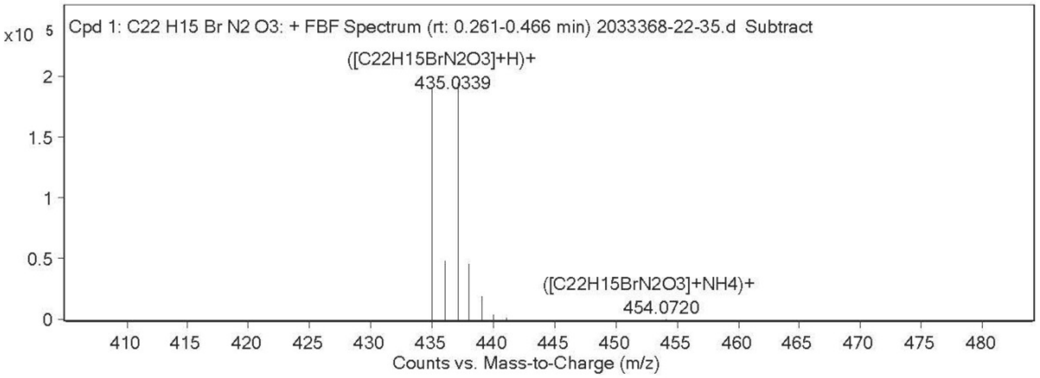


--- End Of Report ---

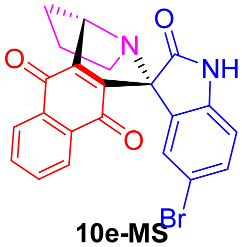


**10e-MS**

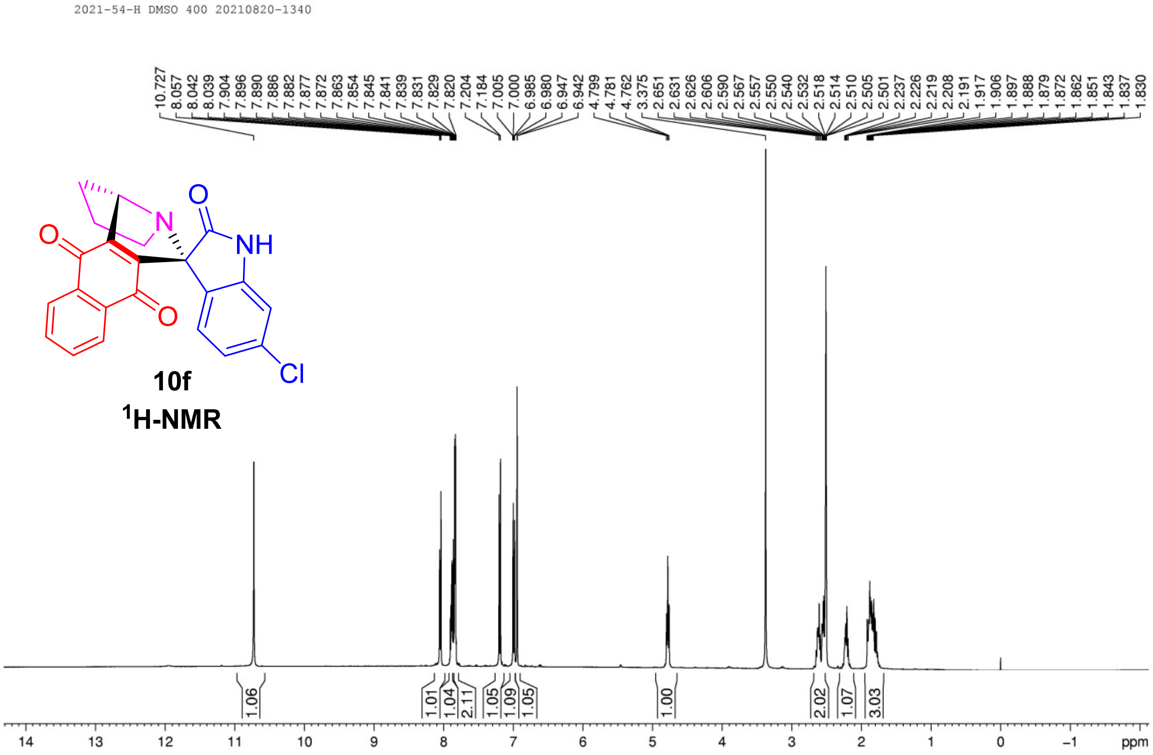

2021-54-H DMSO 400 20210820-1340
10f
1H-NMR
ppm


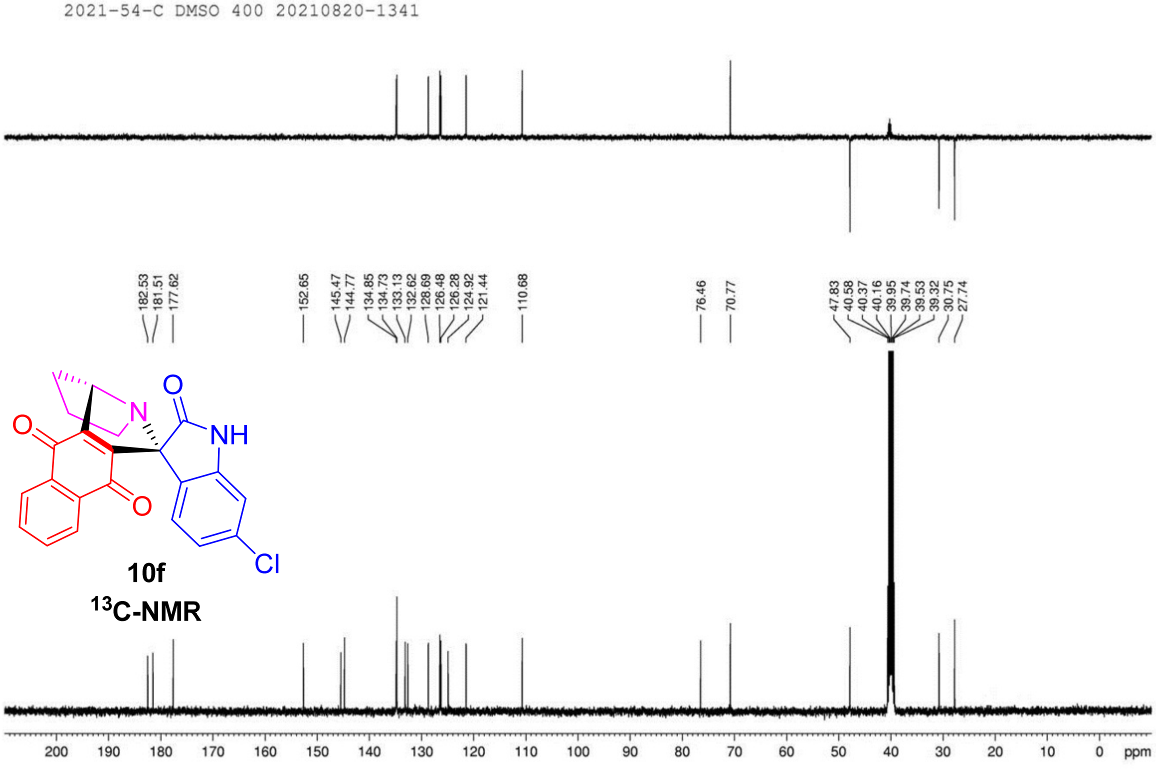

2021-54-C DMSO 400 20210820-1341
10f
13C-NMR
ppm

## Qualitative Compound Report

| | | | |
|---|---|---|---|
| Data File | 2033368-22-54.d | Sample Name | 2033368-22-54 |
| Sample Type | Sample | Position | P1-C7 |
| Instrument Name | Instrument 1 | User Name | |
| Acq Method | pos-1min.m | Acquired Time | 2/6/2023 5:47:51 PM |
| IRM Calibration Status | Success | DA Method | QG-907.m |
| Comment | | | |
| Sample Group | | Info. | |
| Stream Name | LC 1 | Acquisition SW Version | 6200 series TOF/6500 series Q-TOF B.08.00 (B8058.0) |

**Compound Table**

| Compound Label | RT | Mass | Abund | Formula | Tgt Mass | Diff (ppm) |
|---|---|---|---|---|---|---|
| Cpd 1: C22 H15 Cl N2 O3 | 0.112 | 390.0779 | 85267 | C22 H15 Cl N2 O3 | 390.0771 | 2.08 |

MS Zoomed Spectrum

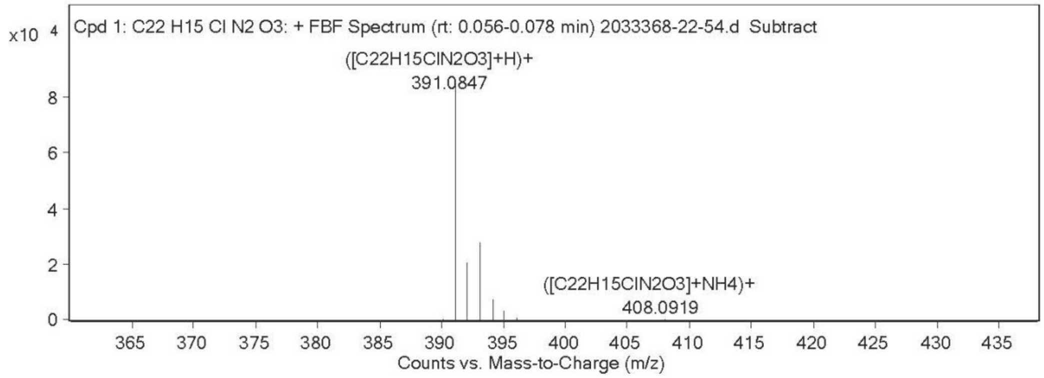


--- End Of Report ---

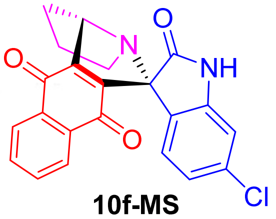


**10f-MS**

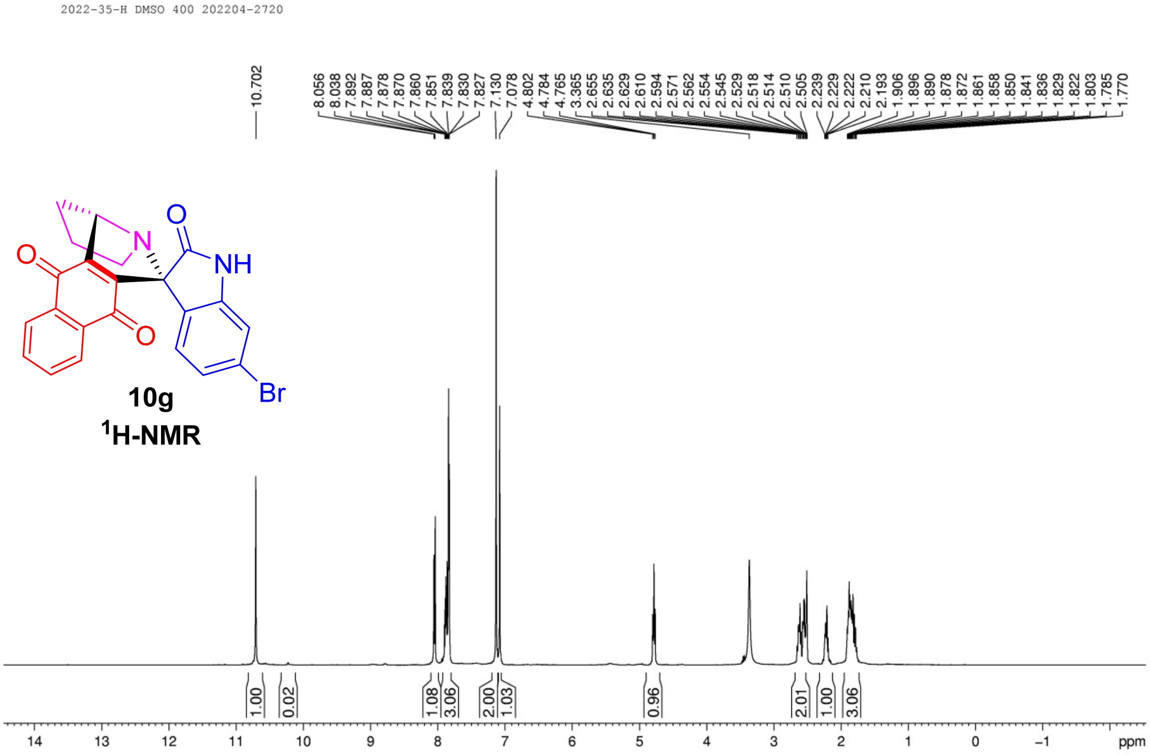
2022-35-H DMSO 400 202204-2720
10g
$^1$H-NMR
ppm

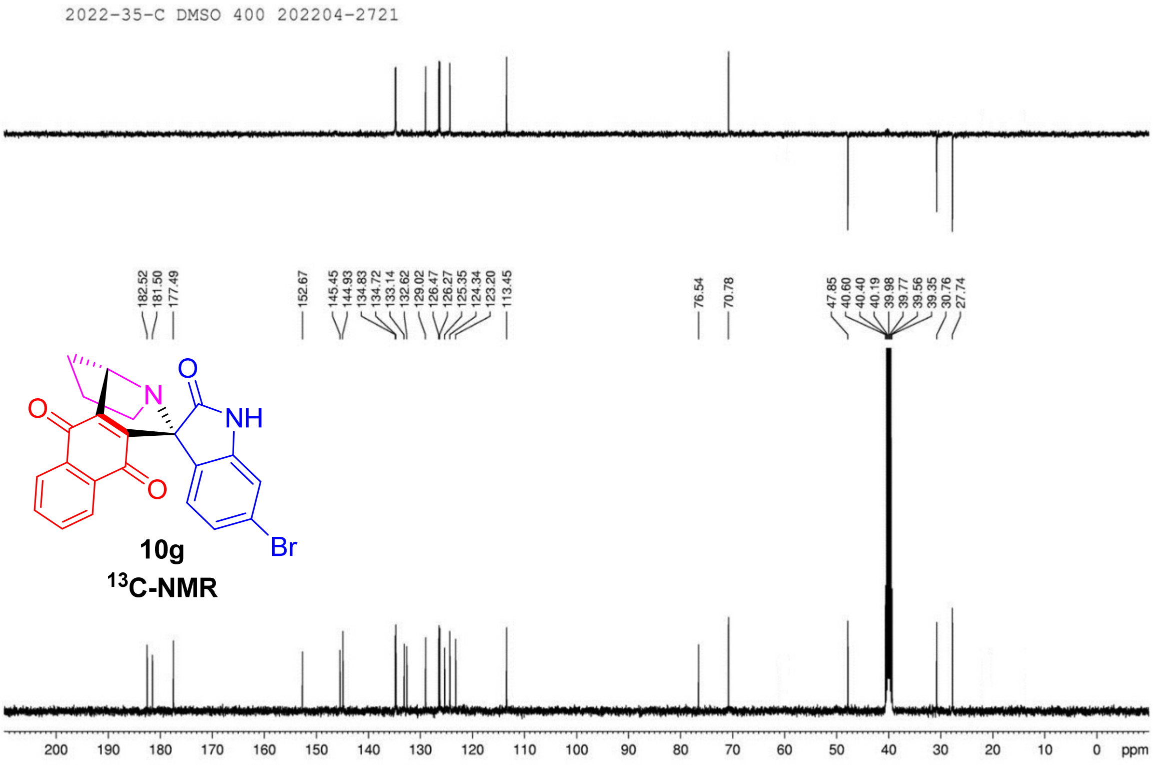
2022-35-C DMSO 400 202204-2721
10g
$^{13}$C-NMR
ppm

## Qualitative Compound Report

| | | | |
|---|---|---|---|
| Data File | 2033368-22-33.d | Sample Name | 2033368-22-33 |
| Sample Type | Sample | Position | P1-B6 |
| Instrument Name | Instrument 1 | User Name | |
| Acq Method | pos-1min.m | Acquired Time | 2/6/2023 5:29:33 PM |
| IRM Calibration Status | Success | DA Method | QG-907.m |
| Comment | | | |
| Sample Group | | Info. | |
| Stream Name | LC 1 | Acquisition SW Version | 6200 series TOF/6500 series Q-TOF B.08.00 (B8058.0) |

**Compound Table**

| Compound Label | RT | Mass | Abund | Formula | Tgt Mass | Diff (ppm) |
|---|---|---|---|---|---|---|
| Cpd 1: C22 H15 Br N2 O3 | 0.127 | 434.028 | 194883 | C22 H15 Br N2 O3 | 434.0266 | 3.17 |

MS Zoomed Spectrum

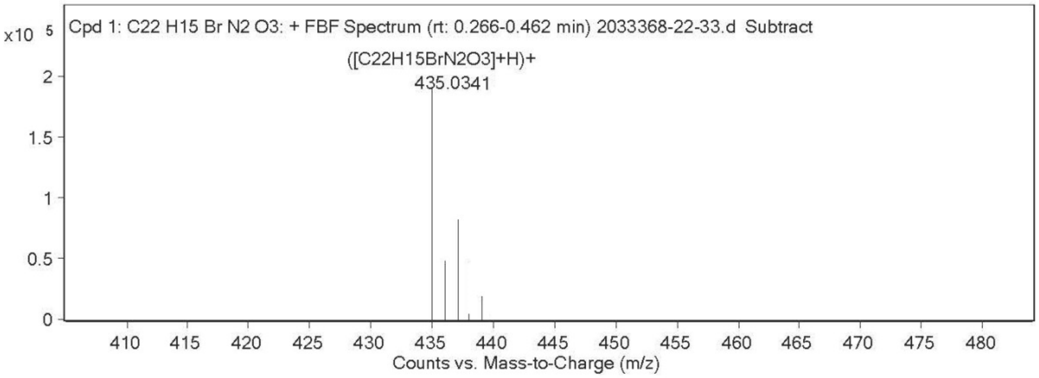


--- End Of Report ---

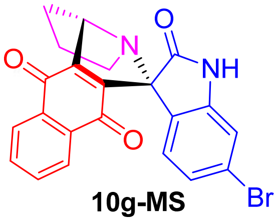


**10g-MS**

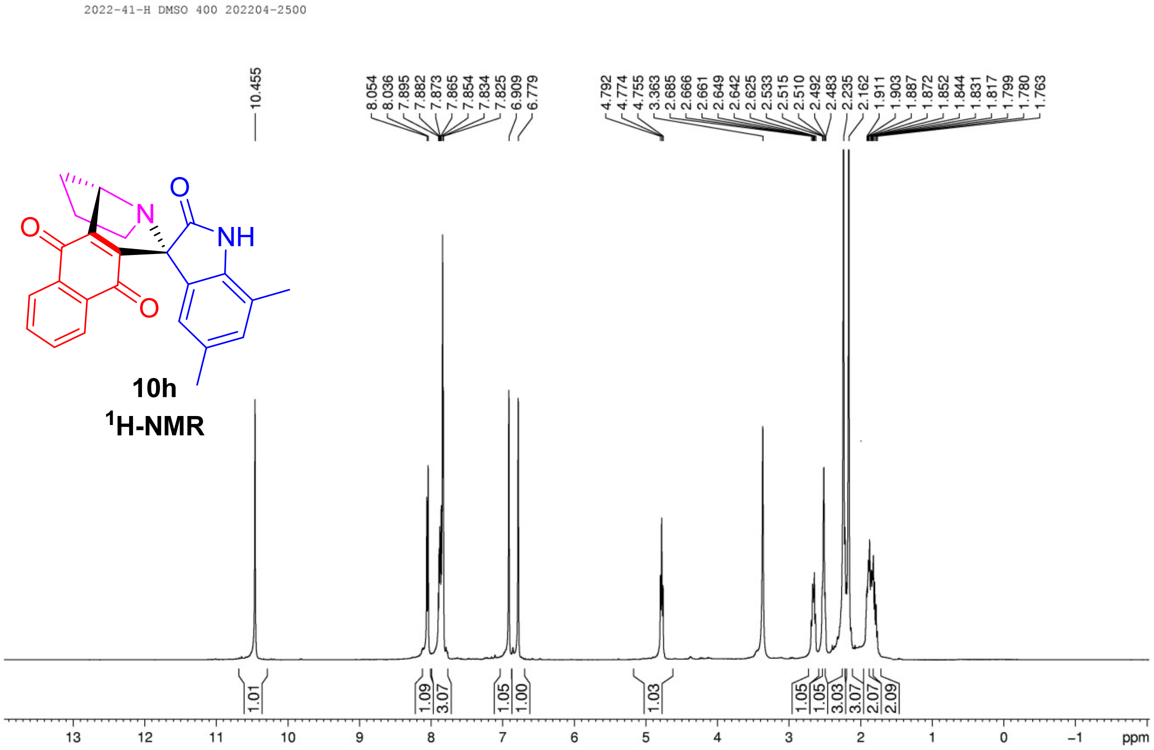


**10h**
**$^{1}$H-NMR**

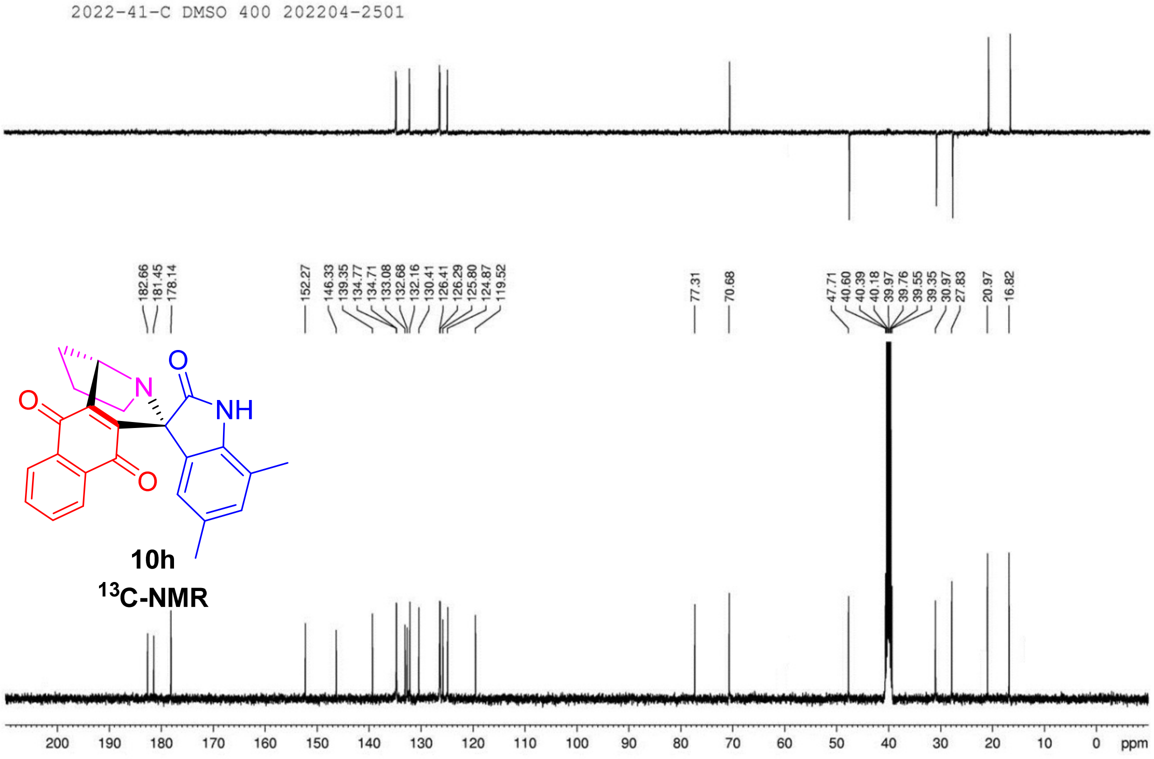


**10h**
**$^{13}$C-NMR**

## Qualitative Compound Report

| | | | |
|---|---|---|---|
| Data File | 2033368-22-41.d | Sample Name | 2033368-22-41 |
| Sample Type | Sample | Position | P1-B8 |
| Instrument Name | Instrument 1 | User Name | |
| Acq Method | pos-1min.m | Acquired Time | 2/6/2023 5:32:48 PM |
| IRM Calibration Status | Success | DA Method | QG-907.m |
| Comment | | | |
| Sample Group | | Info. | |
| Stream Name | LC 1 | Acquisition SW Version | 6200 series TOF/6500 series Q-TOF B.08.00 (B8058.0) |

**Compound Table**

| Compound Label | RT | Mass | Abund | Formula | Tgt Mass | Diff (ppm) |
|---|---|---|---|---|---|---|
| Cpd 1: C24 H20 N2 O3 | 0.503 | 384.1485 | 424849 | C24 H20 N2 O3 | 384.1474 | 2.76 |

MS Zoomed Spectrum

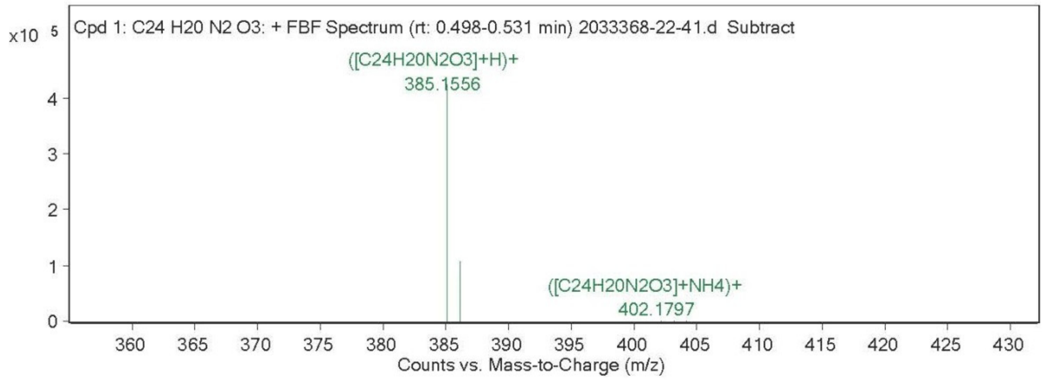


--- End Of Report ---

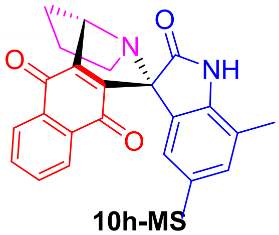


**10h-MS**

# 5. $^{1}$H and $^{13}$C NMR spectra for compounds 17

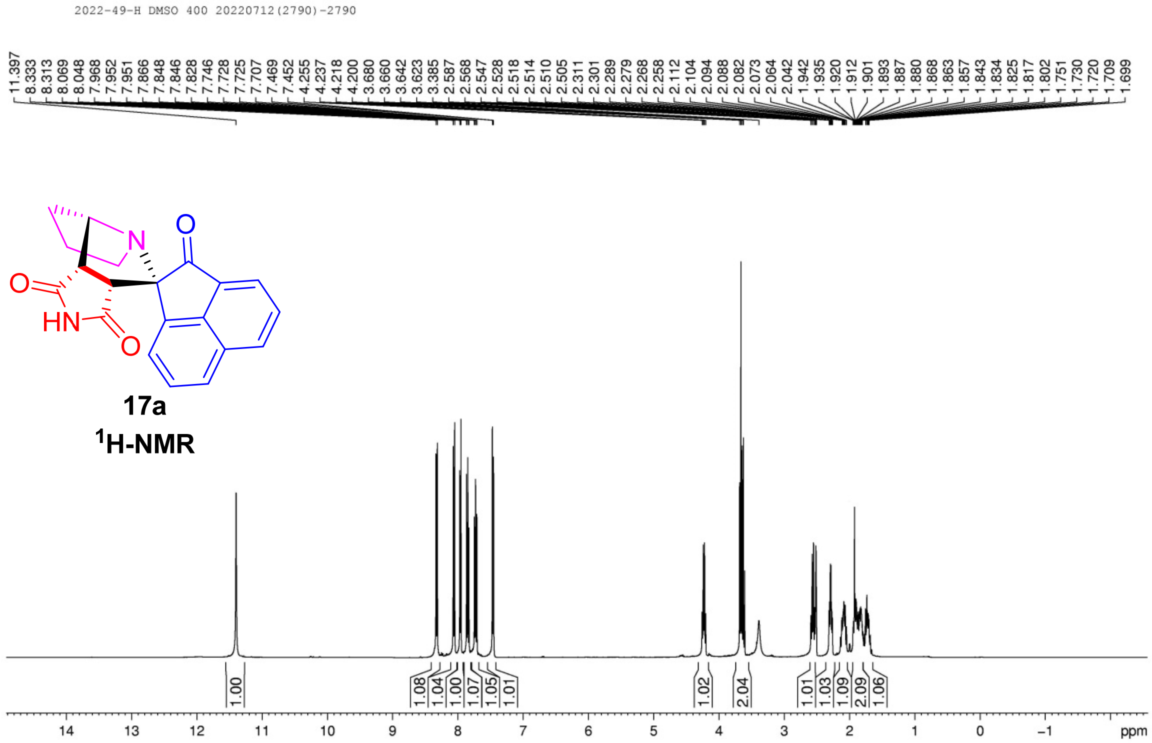


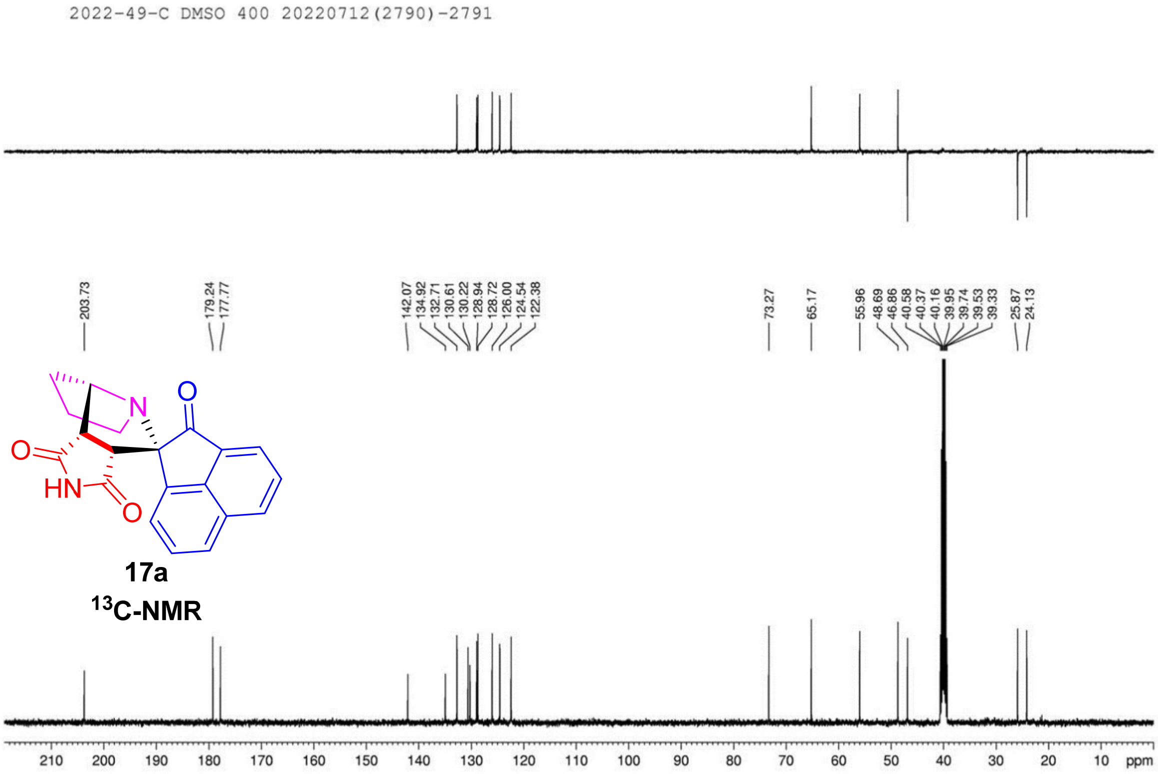

## Qualitative Compound Report

| | | | |
|---|---|---|---|
| Data File | 2033368-22-49.d | Sample Name | 2033368-22-49 |
| Sample Type | Sample | Position | P1-C2 |
| Instrument Name | Instrument 1 | User Name | |
| Acq Method | pos-1min.m | Acquired Time | 2/6/2023 5:38:24 PM |
| IRM Calibration Status | Success | DA Method | QG-907.m |
| Comment | | | |
| Sample Group | | Info. | |
| Stream Name | LC 1 | Acquisition SW Version | 6200 series TOF/6500 series Q-TOF B.08.00 (B8058.0) |

**Compound Table**

| Compound Label | RT | Mass | Abund | Formula | Tgt Mass | Diff (ppm) |
|---|---|---|---|---|---|---|
| Cpd 1: C20 H16 N2 O3 | 0.517 | 332.1167 | 241275 | C20 H16 N2 O3 | 332.1161 | 1.84 |

MS Zoomed Spectrum

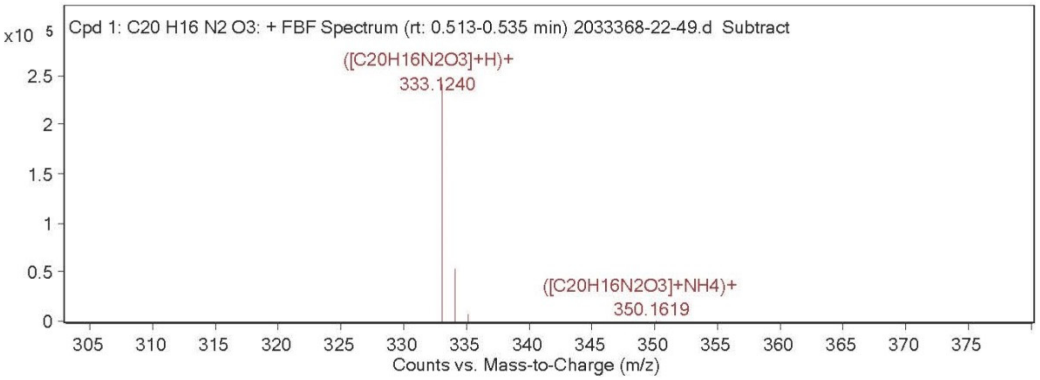


--- End Of Report ---

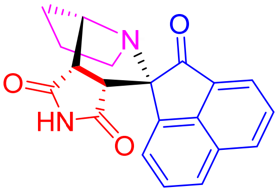


**17a-MS**

2022-50-H DMSO 400 20220712(2790)-2960

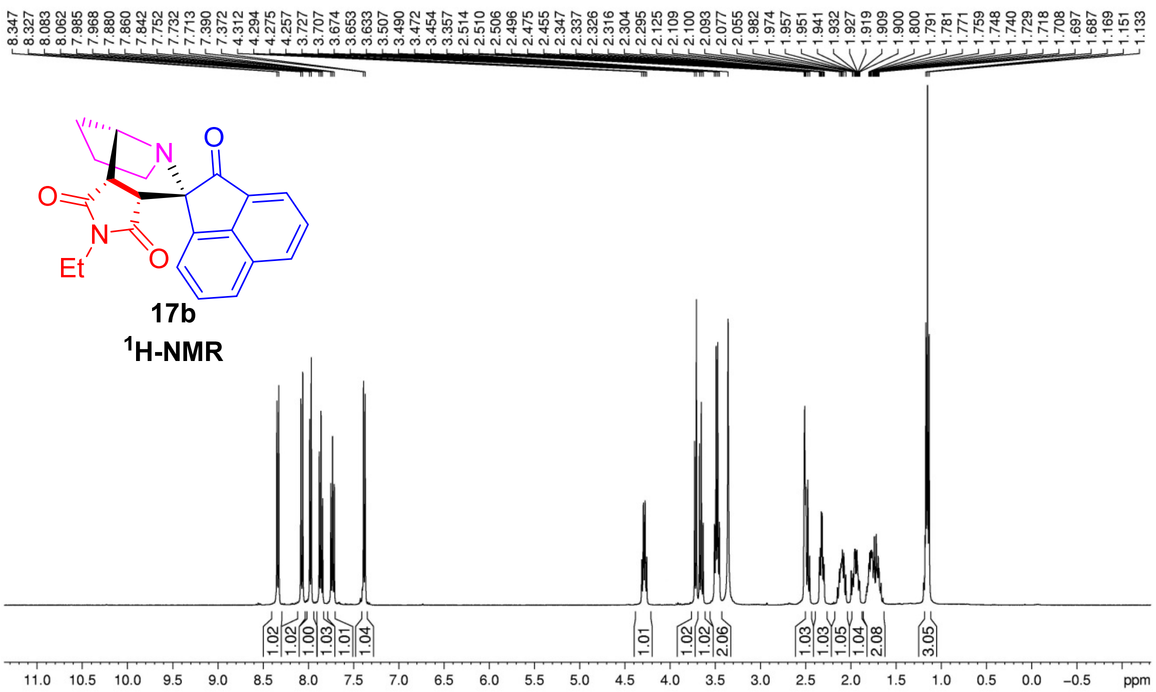


2022-50-C DMSO 400 20220712(2790)-2961

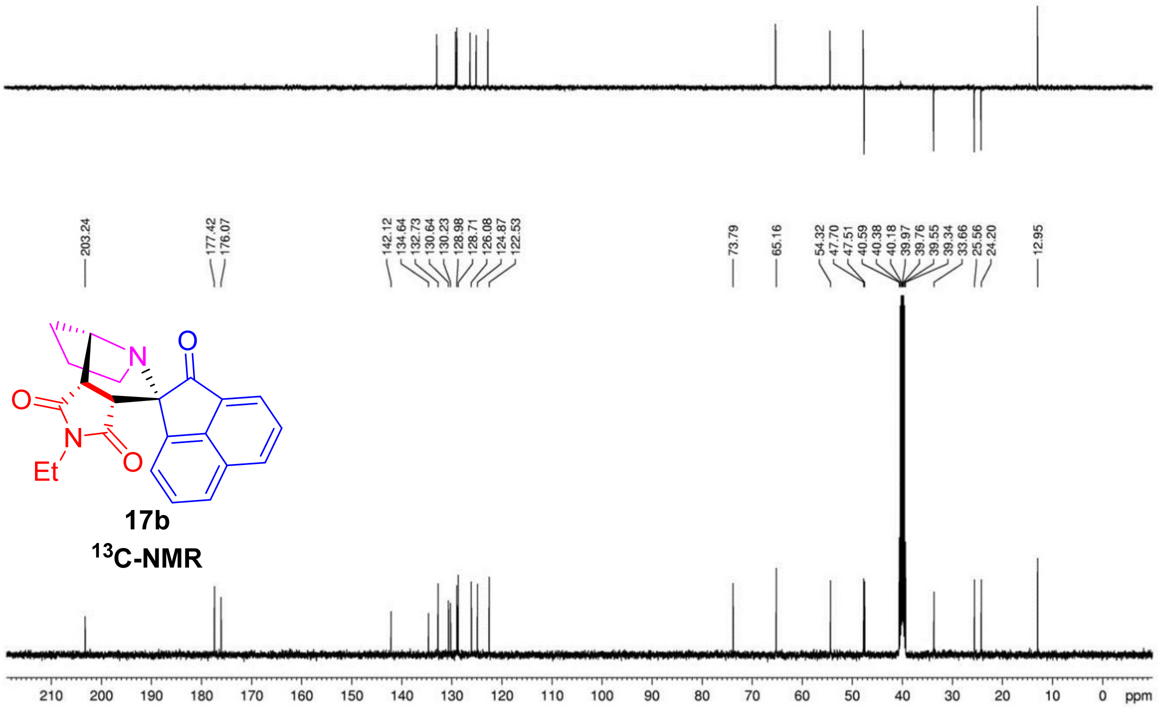

## Qualitative Compound Report

| | | | |
|---|---|---|---|
| Data File | 2033368-22-50.d | Sample Name | 2033368-22-50 |
| Sample Type | Sample | Position | P1-C3 |
| Instrument Name | Instrument 1 | User Name | |
| Acq Method | pos-1min.m | Acquired Time | 2/6/2023 5:40:15 PM |
| IRM Calibration Status | Success | DA Method | QG-907.m |
| Comment | | | |
| Sample Group | | Info. | |
| Stream Name | LC 1 | Acquisition SW Version | 6200 series TOF/6500 series Q-TOF B.08.00 (B8058.0) |

**Compound Table**

| Compound Label | RT | Mass | Abund | Formula | Tgt Mass | Diff (ppm) |
|---|---|---|---|---|---|---|
| Cpd 1: C22 H20 N2 O3 | 0.447 | 360.1479 | 778281 | C22 H20 N2 O3 | 360.1474 | 1.5 |

MS Zoomed Spectrum

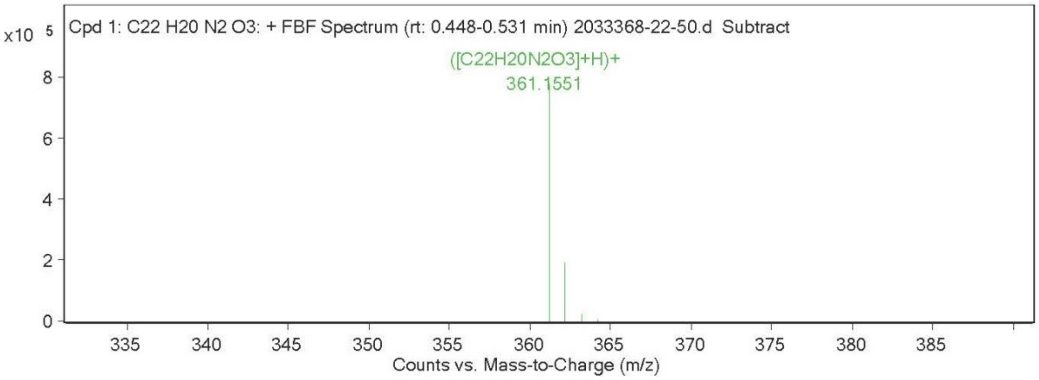


--- End Of Report ---

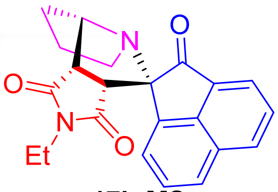


**17b-MS**

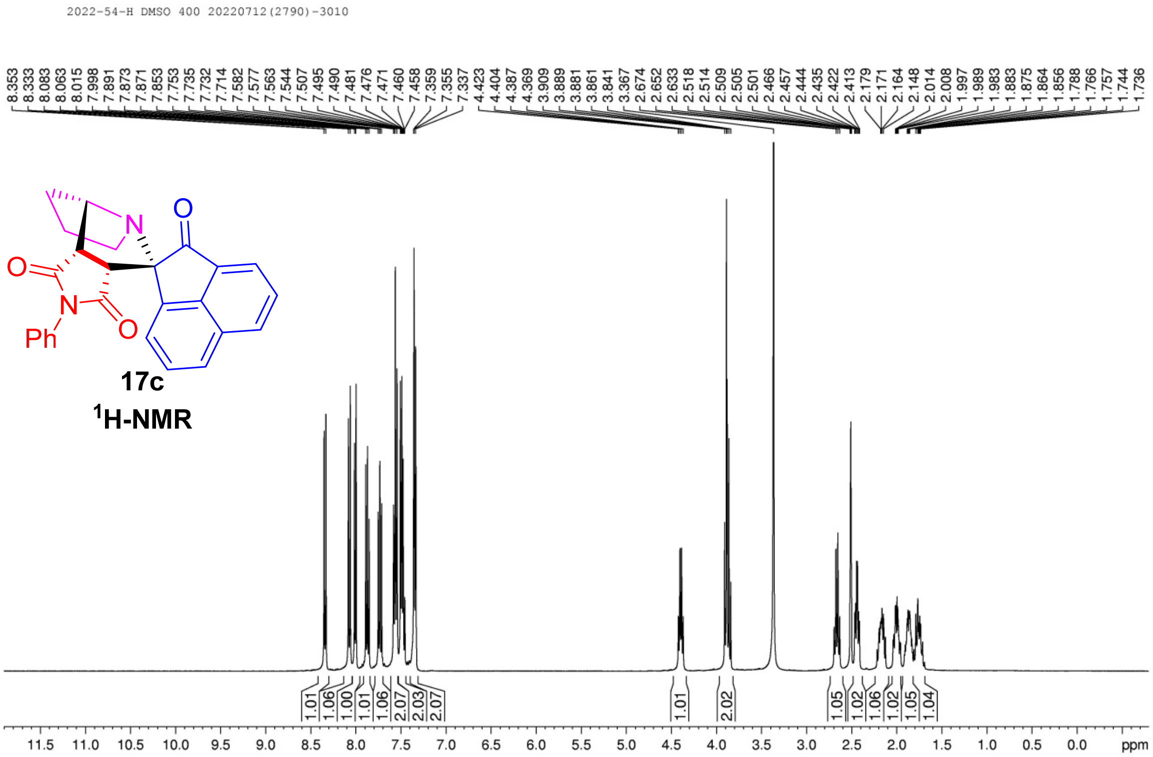
2022-54-H DMSO 400 20220712(2790)-3010
8.353 8.333 8.083 8.063 8.015 7.998 7.891 7.873 7.871 7.853 7.753 7.735 7.732 7.714 7.582 7.577 7.563 7.544 7.507 7.495 7.490 7.481 7.476 7.471 7.460 7.458 7.359 7.355 7.337 4.423 4.404 4.387 4.369 3.909 3.889 3.881 3.861 3.841 3.367 2.674 2.652 2.633 2.518 2.514 2.509 2.505 2.501 2.466 2.457 2.444 2.435 2.422 2.413 2.179 2.171 2.164 2.148 2.014 2.008 1.997 1.989 1.983 1.883 1.875 1.864 1.856 1.788 1.766 1.757 1.744 1.736
17c
1H-NMR
1.01 1.06 1.00 1.01 1.06 2.07 2.03 2.07 1.01 2.02 1.05 1.02 1.06 1.02 1.05 1.04
ppm

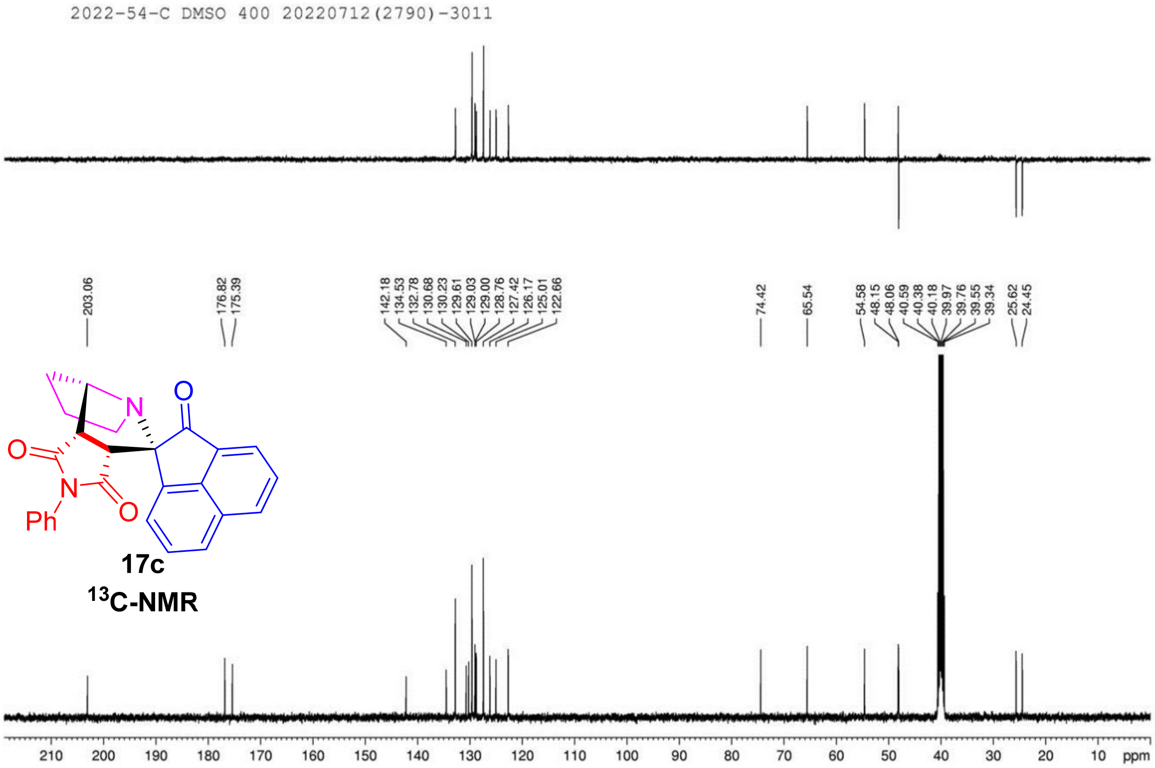
2022-54-C DMSO 400 20220712(2790)-3011
203.06 176.82 175.39 142.18 134.53 132.78 130.68 130.23 129.61 129.03 129.00 128.76 127.42 126.17 125.01 122.66 74.42 65.54 54.58 48.15 48.06 40.59 40.38 40.18 39.97 39.76 39.55 39.34 25.62 24.45
17c
13C-NMR
ppm

## Qualitative Compound Report

| | | | |
|---|---|---|---|
| Data File | 2033368-22-66.d | Sample Name | 2033368-22-66 |
| Sample Type | Sample | Position | P1-C2 |
| Instrument Name | Instrument 1 | User Name | |
| Acq Method | pos-1min.m | Acquired Time | 2/6/2023 5:20:33 PM |
| IRM Calibration Status | Success | DA Method | QG-905.m |
| Comment | | | |
| Sample Group | | Info. | |
| Stream Name | LC 1 | Acquisition SW Version | 6200 series TOF/6500 series Q-TOF B.08.00 (B8058.0) |

**Compound Table**

| Compound Label | RT | Mass | Abund | Formula | Tgt Mass | Diff (ppm) |
|---|---|---|---|---|---|---|
| Cpd 1: C26 H20 N2 O3 | 0.157 | 408.1571 | 85279 | C26 H20 N2 O3 | 408.1552 | 2.24 |

MS Zoomed Spectrum

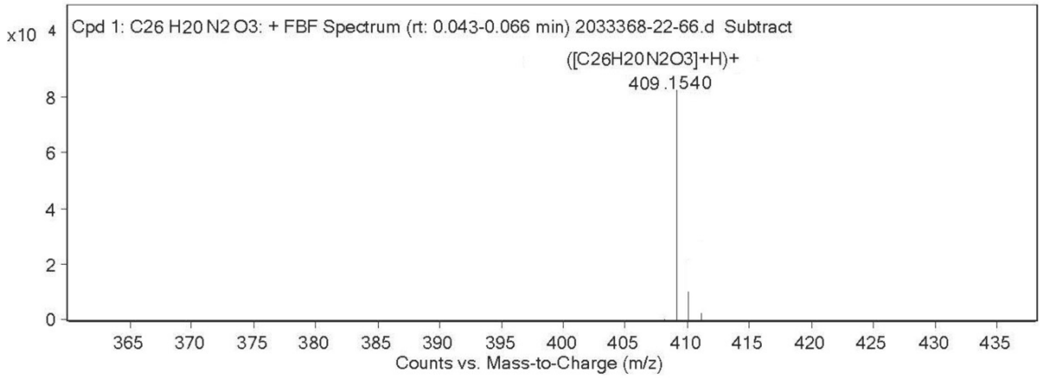


--- End Of Report ---

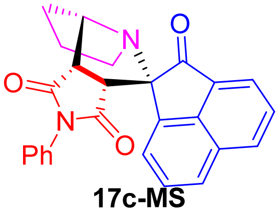


**17c-MS**

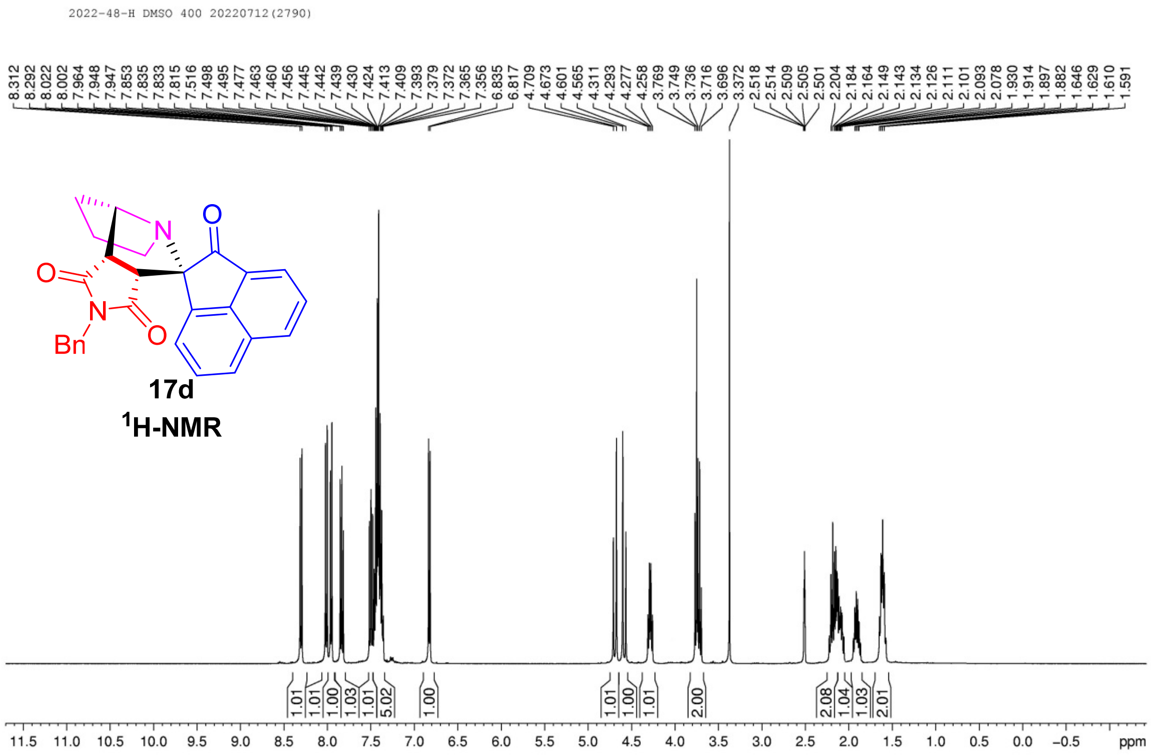
2022-48-H DMSO 400 20220712(2790)
8.312 8.292 8.022 8.002 7.964 7.948 7.947 7.853 7.835 7.833 7.815 7.516 7.498 7.495 7.477 7.463 7.460 7.456 7.445 7.442 7.439 7.430 7.424 7.413 7.409 7.393 7.379 7.372 7.365 7.356 6.835 6.817 4.709 4.673 4.601 4.565 4.311 4.293 4.277 4.258 3.769 3.749 3.736 3.716 3.696 3.372 2.518 2.514 2.509 2.505 2.501 2.204 2.184 2.164 2.149 2.143 2.134 2.126 2.111 2.101 2.093 2.078 1.930 1.914 1.897 1.882 1.646 1.629 1.610 1.591
O
N
O
N
O
Bn
17d
1H-NMR
1.01 1.01 1.00 1.03 1.01 5.02 1.00 1.01 1.00 1.01 2.00 2.08 1.04 1.03 2.01
ppm

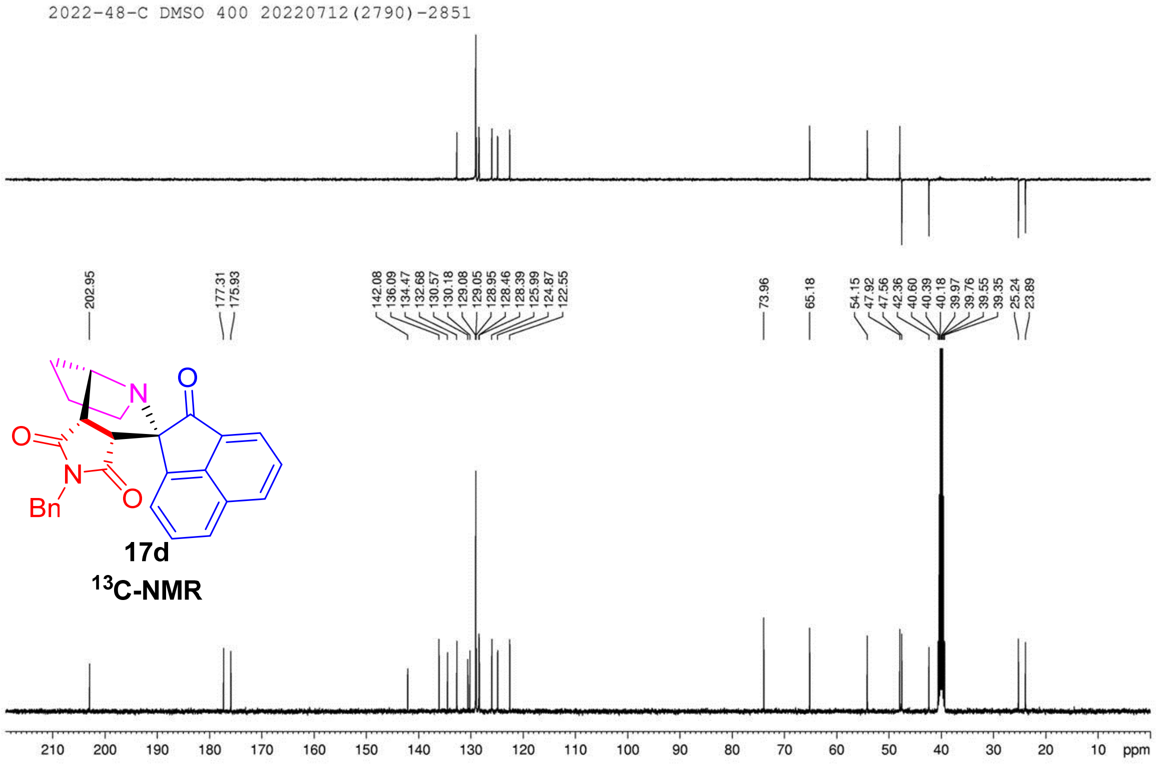
2022-48-C DMSO 400 20220712(2790)-2851
202.95
177.31 175.93
142.08 136.09 134.47 132.68 130.57 130.18 129.08 129.05 128.95 128.46 128.39 125.99 124.87 122.55
73.96
65.18
54.15 47.92 47.56 42.36 40.60 40.39 40.18 39.97 39.76 39.55 39.35 25.24 23.89
O
N
O
N
O
Bn
17d
13C-NMR
ppm

## Qualitative Compound Report

| | | | |
|---|---|---|---|
| Data File | 2033368-22-48.d | Sample Name | 2033368-22-48 |
| Sample Type | Sample | Position | P1-C1 |
| Instrument Name | Instrument 1 | User Name | |
| Acq Method | pos-1min.m | Acquired Time | 2/6/2023 5:36:33 PM |
| IRM Calibration Status | Success | DA Method | QG-907.m |
| Comment | | | |
| Sample Group | | Info. | |
| Stream Name | LC 1 | Acquisition SW Version | 6200 series TOF/6500 series Q-TOF B.08.00 (B8058.0) |

**Compound Table**

| Compound Label | RT | Mass | Abund | Formula | Tgt Mass | Diff (ppm) |
|---|---|---|---|---|---|---|
| Cpd 1: C27 H22 N2 O3 | 0.194 | 422.1641 | 1150205 | C27 H22 N2 O3 | 422.163 | 2.41 |

MS Zoomed Spectrum

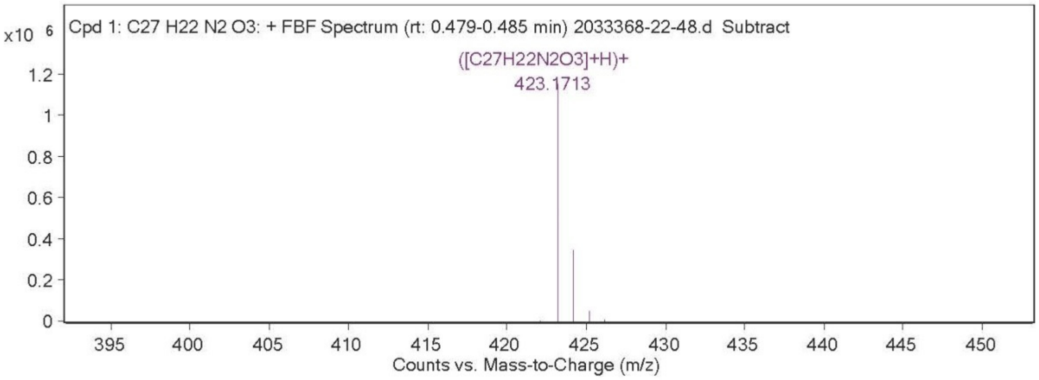


--- End Of Report ---

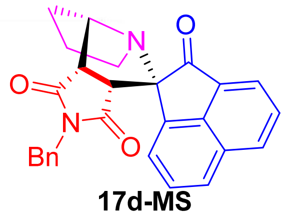


**17d-MS**

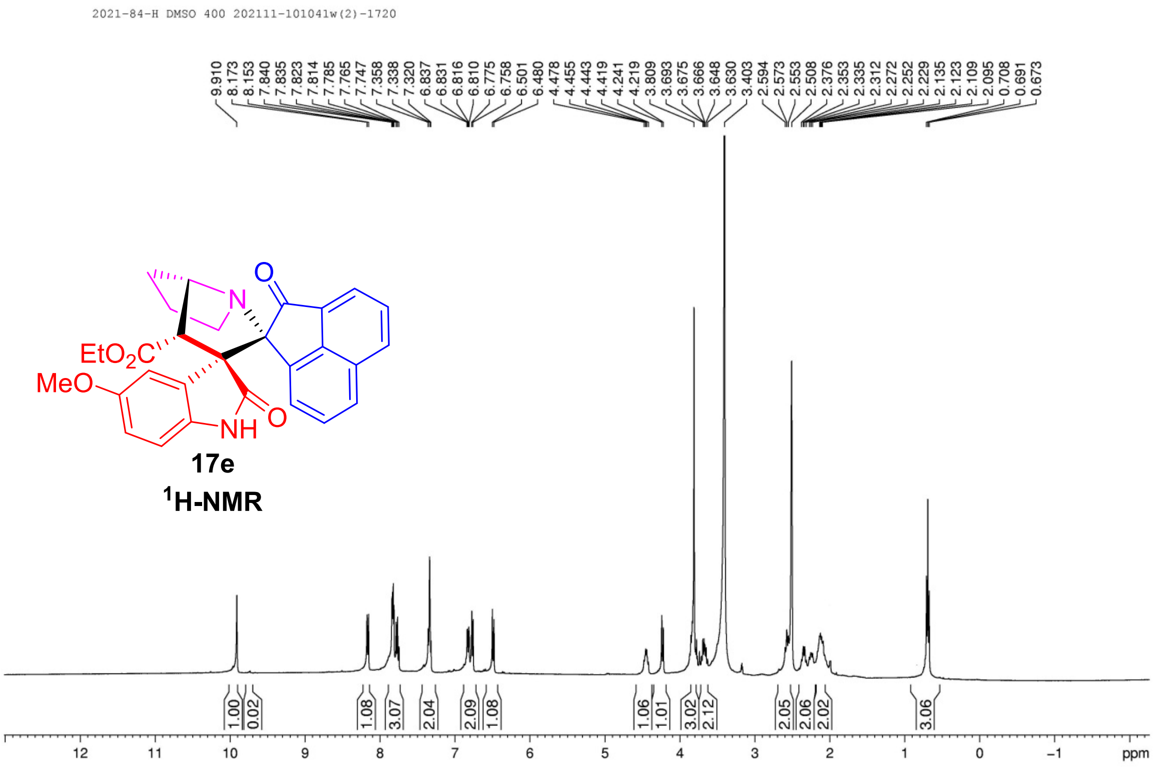
2021-84-H DMSO 400 202111-101041w(2)-1720
17e
1H-NMR

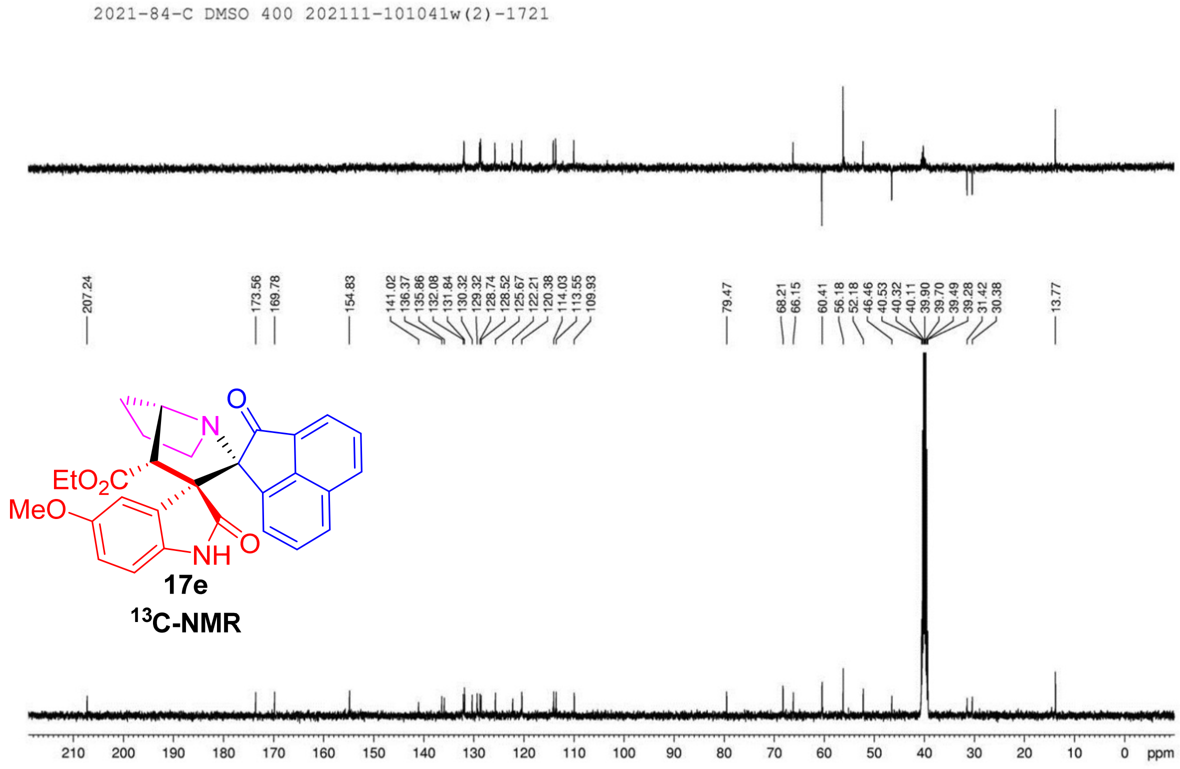
2021-84-C DMSO 400 202111-101041w(2)-1721
17e
13C-NMR

## Qualitative Compound Report

| | | | |
|---|---|---|---|
| Data File | 8036980-22-36.d | Sample Name | 8036980-22-36 |
| Sample Type | Sample | Position | P1-D3 |
| Instrument Name | Instrument 1 | User Name | |
| Acq Method | pos-1min.m | Acquired Time | 8/8/2022 5:40:08 PM |
| IRM Calibration Status | Success | DA Method | default.m |
| Comment | | | |
| Sample Group | | Info. | |
| Stream Name | LC 1 | Acquisition SW Version | 6200 series TOF/6500 series Q-TOF B.08.00 (B8058.0) |

**Compound Table**

| Compound Label | RT | Mass | Abund | Formula | Tgt Mass | Diff (ppm) |
|---|---|---|---|---|---|---|
| Cpd 1: C29H26 N2 O5 | 0.273 | 482.1917 | 273849 | C29H26 N2 O5 | 482.1912 | 3.02 |

MS Zoomed Spectrum

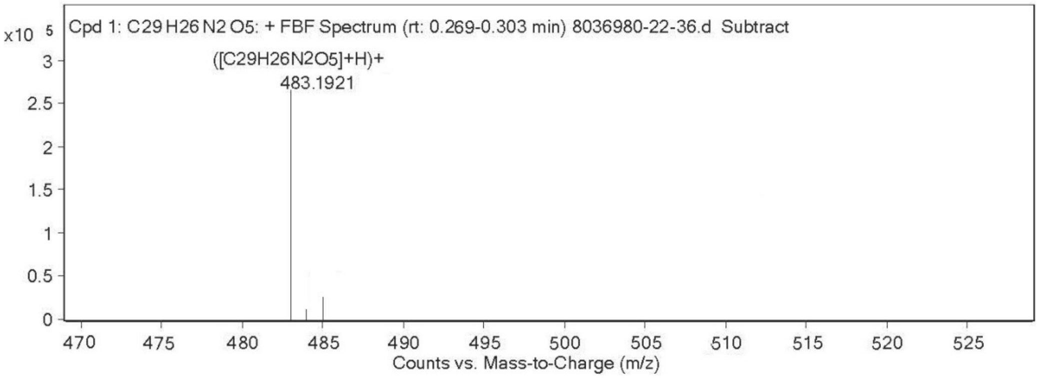


--- End Of Report ---

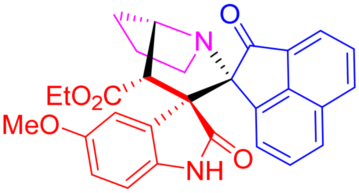


**17e-MS**

## 6. Single crystal X-ray diffraction study data of compound 7a

# checkCIF/PLATON report

Structure factors have been supplied for datablock(s) exp_1883_auto

THIS REPORT IS FOR GUIDANCE ONLY. IF USED AS PART OF A REVIEW PROCEDURE FOR PUBLICATION, IT SHOULD NOT REPLACE THE EXPERTISE OF AN EXPERIENCED CRYSTALLOGRAPHIC REFEREE.

No syntax errors found. CIF dictionary Interpreting this report

## Datablock: exp_1883_auto

---

| | | | |
|---|---|---|---|
| Bond precision: | C-C = 0.0013 A | | Wavelength=0.71073 |
| Cell: | a=7.1626(1) | b=10.5723(2) | c=11.2748(3) |
| | alpha=67.370(2) | beta=76.521(2) | gamma=76.028(2) |
| Temperature: | 100 K | | |

| | Calculated | Reported |
|---|---|---|
| Volume | 755.31(3) | 755.31(3) |
| Space group | P -1 | P -1 |
| Hall group | -P 1 | -P 1 |
| Moiety formula | C18 H19 N3 O3 | C18 H19 N3 O3 |
| Sum formula | C18 H19 N3 O3 | C18 H19 N3 O3 |
| Mr | 325.36 | 325.36 |
| Dx,g cm-3 | 1.431 | 1.431 |
| Z | 2 | 2 |
| Mu (mm-1) | 0.099 | 0.099 |
| F000 | 344.0 | 344.0 |
| F000' | 344.15 | |
| h,k,lmax | 11,17,18 | 11,17,17 |
| Nref | 7069 | 6312 |
| Tmin,Tmax | 0.982,0.990 | 0.734,1.000 |
| Tmin' | 0.980 | |

Correction method= # Reported T Limits: Tmin=0.734 Tmax=1.000
AbsCorr = MULTI-SCAN

Data completeness= 0.893 Theta(max)= 35.800

R(reflections)= 0.0425( 5108) wR2(reflections)= 0.1239( 6312)

S = 1.081 Npar= 218

The following ALERTS were generated. Each ALERT has the format
test-name_ALERT_alert-type_alert-level.
Click on the hyperlinks for more details of the test.

### Alert level G

PLAT007_ALERT_5_G Number of Unrefined Donor-H Atoms ............. 1 Report
PLAT154_ALERT_1_G The s.u.'s on the Cell Angles are Equal ..(Note) 0.002 Degree
PLAT793_ALERT_4_G Model has Chirality at C1 (Centro SPGR) S Verify
PLAT793_ALERT_4_G Model has Chirality at C2 (Centro SPGR) R Verify
PLAT793_ALERT_4_G Model has Chirality at C6 (Centro SPGR) S Verify
PLAT793_ALERT_4_G Model has Chirality at C7 (Centro SPGR) S Verify
PLAT912_ALERT_4_G Missing # of FCF Reflections Above STh/L= 0.600 720 Note
PLAT941_ALERT_3_G Average HKL Measurement Multiplicity ........... 2.6 Low
PLAT978_ALERT_2_G Number C-C Bonds with Positive Residual Density. 17 Info

0 ALERT level A = Most likely a serious problem - resolve or explain
0 ALERT level B = A potentially serious problem, consider carefully
0 ALERT level C = Check. Ensure it is not caused by an omission or oversight
9 ALERT level G = General information/check it is not something unexpected

1 ALERT type 1 CIF construction/syntax error, inconsistent or missing data
1 ALERT type 2 Indicator that the structure model may be wrong or deficient
1 ALERT type 3 Indicator that the structure quality may be low
5 ALERT type 4 Improvement, methodology, query or suggestion
1 ALERT type 5 Informative message, check

It is advisable to attempt to resolve as many as possible of the alerts in all categories. Often the minor alerts point to easily fixed oversights, errors and omissions in your CIF or refinement strategy, so attention to these fine details can be worthwhile. In order to resolve some of the more serious problems it may be necessary to carry out additional measurements or structure refinements. However, the purpose of your study may justify the reported deviations and the more serious of these should normally be commented upon in the discussion or experimental section of a paper or in the "special_details" fields of the CIF. checkCIF was carefully designed to identify outliers and unusual parameters, but every test has its limitations and alerts that are not important in a particular case may appear. Conversely, the absence of alerts does not guarantee there are no aspects of the results needing attention. It is up to the individual to critically assess their own results and, if necessary, seek expert advice.

Publication of your CIF in IUCr journals

A basic structural check has been run on your CIF. These basic checks will be run on all CIFs submitted for publication in IUCr journals (*Acta Crystallographica*, *Journal of Applied Crystallography*, *Journal of Synchrotron Radiation*); however, if you intend to submit to *Acta Crystallographica Section C* or *E* or *IUCrData*, you should make sure that full publication checks are run on the final version of your CIF prior to submission.

Publication of your CIF in other journals

Please refer to the *Notes for Authors* of the relevant journal for any special instructions relating to CIF submission.

PLATON version of 18/05/2022; check.def file version of 17/05/2022

Datablock exp_1883_auto- ellipsoid plot

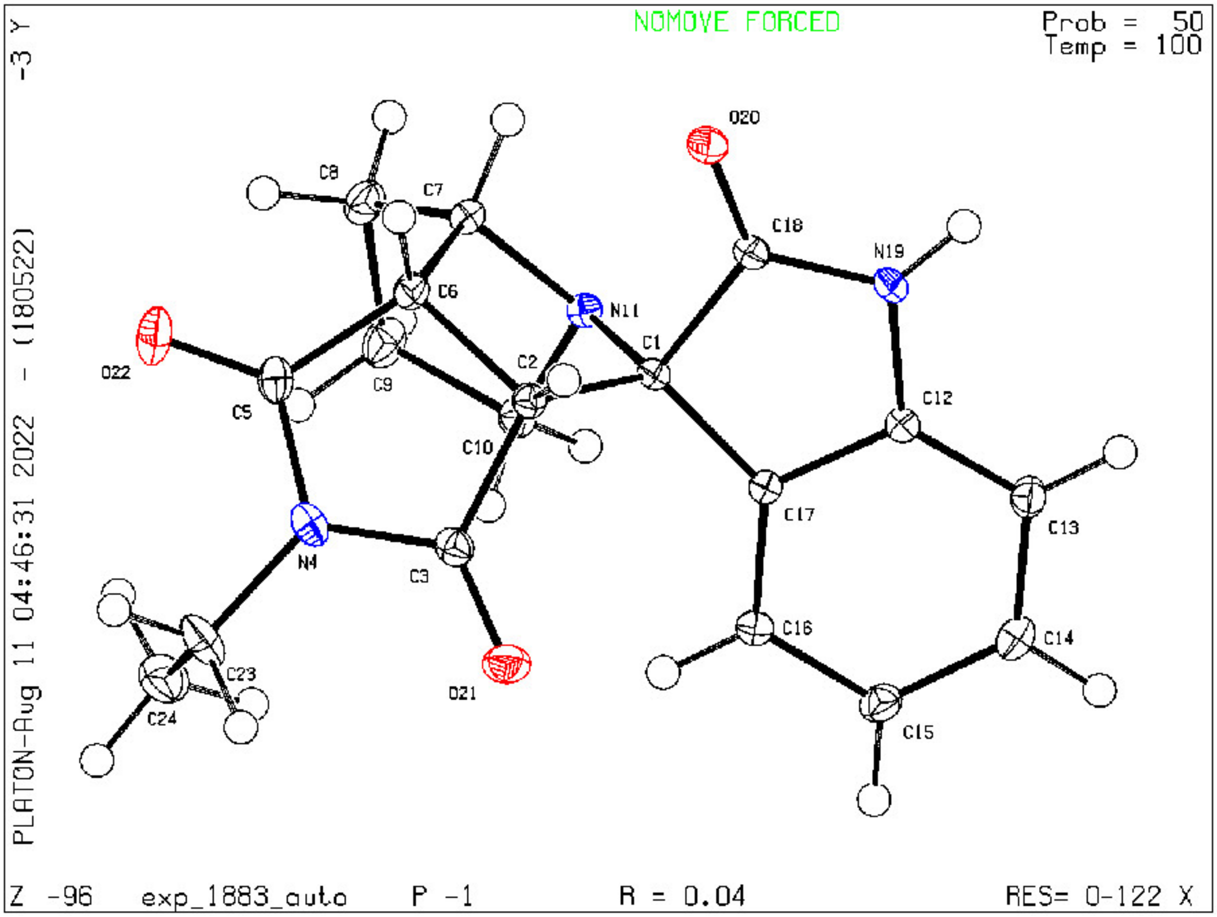

Supplement: Supplementary file 1 [file ijms-24-03771-s001.zip › ijms-2178663-supplementary.pdf]
